# The complex domain architecture of SAMD9 family proteins, predicted STAND-like NTPases, suggests new links to inflammation and apoptosis

**DOI:** 10.1186/s13062-017-0185-2

**Published:** 2017-05-25

**Authors:** Sergei L. Mekhedov, Kira S. Makarova, Eugene V. Koonin

**Affiliations:** National Center for Biotechnology Information, National Library of Medicine, National Institutes of Health, Bethesda, MD 20894 USA

## Abstract

**Abstract:**

We report a comprehensive computational dissection of the domain architecture of the SAMD9 family proteins that are involved in antivirus and antitumor response in humans. We show that the SAMD9 protein family is represented in most animals and also, unexpectedly, in bacteria, in particular actinomycetes. From the N to C terminus, the core SAMD9 family architecture includes DNA/RNA-binding AlbA domain, a variant Sir2-like domain, a STAND-like P-loop NTPase, an array of TPR repeats and an OB-fold domain with predicted RNA-binding properties. Vertebrate SAMD9 family proteins contain the eponymous SAM domain capable of polymerization, whereas some family members from other animals instead contain homotypic adaptor domains of the DEATH superfamily, known as dedicated components of apoptosis networks. Such complex domain architecture is reminiscent of the STAND superfamily NTPases that are involved in various signaling processes, including programmed cell death, in both eukaryotes and prokaryotes. These findings suggest that SAMD9 is a hub of a novel, evolutionarily conserved defense network that remains to be characterized.

**Reviewers:**

This article was reviewed by Igor B. Zhulin and Mensur Dlakic.

**Electronic supplementary material:**

The online version of this article (doi:10.1186/s13062-017-0185-2) contains supplementary material, which is available to authorized users.

## Findings

Innate immunity is a universal attribute of cellular life. Animals, in particular, possess multiple, diverse and elaborate innate immunity mechanisms, and new ones are being continuously discovered [1–3]. The human sterile alpha motif protein 9 (SAMD9) is a ubiquitously expressed cytoplasmic protein with diverse but still poorly characterized functions ranging from stress response to antiviral and antineoplasic activities [4]. Mutations in the SAMD9 gene are associated with normophosphatemic familial tumoral calcinosis [5, 6] as well as the MIRAGE (myelodysplasia, infection, restriction of growth, adrenal hypoplasia, genital phenotypes, and enteropathy) syndrome [7]. The molecular mechanisms of the activities of SAMD9 and its close paralog SAMD9L remain largely obscure. SAMD9 and SAMD9L are significantly down-regulated in various neoplasms including aggressive fibromatosis, breast and colon cancers [8]. Overexpression of SAMD9 protein in tissue culture results in reduction of cell proliferation rate and invasion index, and increasing activity of caspase 3 [8, 9]. In addition, SAMD9 is an innate antiviral host factor and is the target of poxvirus host range factors of the C7L family [10–12] that have been shown to bind SAMD9 via a distinct “molecular claw” [13]. SAMD9 also binds the E6 protein of certain human papillomavirus strains [14]. Additionally, knockdown of SAMD9 enhances the replication of Japanese Encephalitis virus, a positive-strand RNA virus [15]. Furthermore, the SAMD9 gene is tightly regulated by gamma interferon [16]. Together, these findings establish SAMD9 as broad spectrum antiviral and antitumor factor.

Previous analyses of protein domains in the SAMD9 protein family demonstrated the presence of a typical, highly conserved sterile alpha motif (SAM) domain at the N-terminus. This particular subclass of SAM domains has been shown to mediate protein-protein rather than protein-RNA interactions and indeed has been shown to form polymeric complexes [17]. In a recent study on the anti-poxvirus activity of SAMD9, the presence of another domain, a putative P-loop NTPase, has been pointed out [18]. Here we report the results of an exhaustive dissection of the domain architectures of SAMD9 family proteins using sensitive methods of sequence analysis. This analysis reveals a highly complex domain organization of SAMD9 homologs and implies their involvement in an apoptosis-related signaling network.

**Fig. 1:**
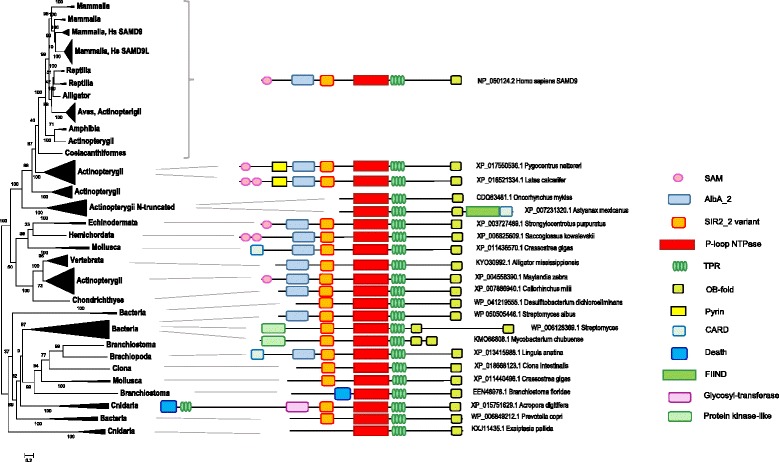
Phylogenetic tree and domain architectures of the SAMD9 family proteins. Domain identities and positions are based on multiple searches with the representative sequences, for which the architectures are shown, as queries to search the CDD, HHpred, Pfam, and SMART databases (see Additional file 1: Table S1) and multiple alignments with human SAMD9 as well as with the sequences within the collapsed branches. The multiple alignment used to build the tree was trimmed down to the conserved core, from the SIR2-like domain through the OB-fold domain (the first upstream copy in the sequences with two OB-fold domains). GenBank identifiers and species names are shown for all domain architectures. Amino acid sequences of the SAMD9 family members were collected from Non-redundant protein sequences database (nr) at the NCBI using BLASTP. Distant homologs containing only a single domain with significant similarity tp SAMD9 proteins were discarded. Sequences were clustered into putative orthologous sets, and highly similar sequences were purged using BLASTClust with –L 0.9 –S 0.9 parameters (https://www.ncbi.nlm.nih.gov/Web/Newsltr/Spring04/blastlab.html). Domain architectures were analyzed by batch CDD search with Expect Value threshold 1.0 at https://www.ncbi.nlm.nih.gov/Structure/bwrpsb/bwrpsb.cgi [33] followed by manual inspection and HHpred [20]. Multiple sequence alignments were constructed using MUSCLE with default parameters [34], and the phylogenetic tree was constructed using FastTree with the WAG evolutionary model and discrete gamma model with 20 rate categories [35]. The numbers at internal branches indicate bootstrap support (percent)

Previous evolutionary analysis of the SAMD9 family has demonstrated an early duplication of this gene in mammalian evolution and the loss of either SAMD9 or SAMD9L in some species but has not explored deep evolutionary conservation of these proteins [4]. We performed exhaustive BLAST searches [19] of the non-redundant protein sequence database at the NCBI starting with mammalian query sequences. Despite earlier claims that members of SAMD9 family could not be detected beyond Vertebrata [4], we identified clear homologs with highly significant sequence similarity to SAMD9 in Echinodermata, Mollusca, Hemichordata, Brachiopoda, Cnidaria, as well as diverse bacteria. It is notable, however, that SAMD9 orthologs are missing in some major metazoan lineages, in particular, nematodes and arthropods. Given the presence of SAMD9 in Cnidaria, a basal metazoan clade, this pattern implies at least one gene loss during metazoan evolution, possibly, at the base of the Ecdysozoan clade. These sequences were used as queries for reciprocal BLAST searches to validate orthology. The resulting set of 575 protein sequences was trimmed by removing highly similar sequences, and the remaining, non-redundant subset of 208 sequences was used for analysis of the domain architecture and phylogeny. All these sequences were used to obtain preliminary data on the domain composition of the SAMD9 family by batch searches of Pfam and CDD databases. A small selection of 27 sequences was used as queries for HHpred, a highly sensitive profile-profile search method [20]. This analysis revealed a common domain architecture of the SAMD9 family proteins. The following domains comprise the core, presumably, ancestral architecture, in the order from the N- to the C-terminus: putative DNA-binding AlbA_2 domain, a domain distantly related to SIR2 family histone deacetylases, a P-loop NTPase domain followed by tandem TPR repeats, and a C-terminal OB-fold domain most similar to that of the cold-shock DNA-binding proteins; the AlbA_2 and Sir2-like domains are lost in some family members (Fig. 1; see Additional file 1 for the statistical significance of the domain detection). The P-loop NTPase domains of SAMD9 and its homologs show the highest similarity to that of the major component of apoptosome, Apaf-1, a member of the superfamily of signal transduction ATPases with numerous domains (STAND) [21] (see Additional file 1). However, the P-loop domains in the SAMD9 family proteins contain a highly unusual amino acid substitution in the P-loop (Walker A motif), namely, a glycine replacing the otherwise conserved lysine residue, resulting in GGT/S signature instead of the canonical GKT/S (see Additional file 2). The invariant lysine of the Walker A motif is directly involved in NTP hydrolysis, and substitution of this residue typically inactivates P-loop NTPases. Therefore it cannot be ruled out that the P-loop containing domain of the SAMD9 family is a NTP-binding protein but not an NTPase. Alternatively, the absence of the lysine in the P-loop might be complemented by a different electrophilic residue, e.g. the upstream histidine of the P-loop that is conserved throughout the SAMD9 family (See Additional file 2). A similar replacement of the canonical P-loop lysine with an upstream lysine residue has been reported for bacteriophage terminases [22]

The SAMD9 family proteins showed marked variability of domain architectures in their N-terminal parts (Fig. 1). The eponymous SAM domain is limited to vertebrates and is missing even in some vertebrate family members. Instead, many proteins from other animals contain DEATH superfamily (CARD, Death, Pyrin and FIIND), and a subfamily of fish proteins contains both SAM and Pyrin domains. Many bacterial members of the SAMD9 family possess a distinct domain architecture, with the AlbaA_2 domain missing and a Ser/Thr protein kinase domain located at the N-terminus (Fig. 1). Each of these domain architectures is conserved in at least a small subfamily of the SAMD9 family, effectively ruling out the possibility that the corresponding protein sequences result from inaccurate, chimeric gene models.

The conserved core of the SAMD9 family was used to build a phylogenetic tree (Fig. 1). The tree confirms the previously reported duplication in mammals [4] and reveals a number of independent duplications in vertebrates, particularly in bony fishes, and beyond the vertebrate lineage. Superposition of the domain architectures over the tree shows that duplications were followed by domain accretions and losses, and hence diversification of domain architectures, and by inference, protein functions.

The complex domain architecture of the SAMD9 family proteins identified by our analysis bears a striking resemblance to the architectures of the STAND superfamily NTPases that are involved in various forms of signal transduction but most prominently, in programmed cell death and antivirus response in animals and plants [21, 23]. A further similarity between the SAMD9 family and the STAND NTPases is their phyletic pattern, i.e. representation in multicellular eukaryotes and bacteria, primarily, those with complex life cycles, such as actinomycetes. A notable difference, however, is the apparent absence of the SAMD9 family proteins in plants. The architectural similarity between SAMD9 and STAND complements the sequence similarity between the NTPase domain of SAMD9 and the nucleotide-binding and oligomerization domain (NOD) of Apaf1, the major component of the animal apoptosome, the key mediator of programmed cell death [24, 25]. Another strong connection to apoptosis and inflammation is the presence, in fish and invertebrate members of the SAMD9 family, of CARD, Death, PYRIN and FIIND domains (Fig. 1; and see Additional file 3). These distantly related domains comprise the DEATH domain superfamily and mediate homotypic interactions that hold together the signaling networks that are involved in apoptosis and inflammation [26–28]. It appears most likely that, in vertebrate SAMD9 family proteins, the SAM domain functionally substitutes for the DEATH superfamily domains. Taken together, these observations strongly suggest that SAMD9 family proteins are hubs of a novel defense network that might function through a distinct form of apoptosis. Furthermore, we predict that these proteins form multisubunit particles architecturally similar to apoptosomes and inflammasomes, and regulate the function of the putative network via NTP binding or hydrolysis.

The presence of two predicted nucleic acid-binding domains, the DNA-binding AlbA_2 and the OB-fold that is likely to bind RNA, along with the SIR2-like domain, in the core architecture of the SAMD9 family (Fig. 1) could provide additional functional clues. Archaeal SIR2 interacts with the major chromatin protein Alba by binding and deacetylating its lysine residues, which stimulates AlbA binding to DNA and turns off gene expression [29, 30]. The presence of AlbaA_2 and SIR2 domains within the same cytoplasmic protein is without precedent and unexpected but the archaeal analogy suggests that AlbaA_2 functions as a cytoplasmic DNA sensor and silencer that is regulated by SIR2 via reversible modification. It has been shown that many AlbA family proteins bind RNA [31] but the functional coupling with the SIR2 domain suggests that the domain in SAMD9 is DNA-binding.

Recently, it has been shown that, in human cell culture, SAMD9 interacts with the Myxoma virus host range proteins M062 and M063 via a portion of the protein that corresponds to the AlbaA_2 domain recognized here [18]. It appears likely that these viral proteins preclude binding of the AlbaA_2 domain of SAMD9 to the viral DNA. In contrast, the OB-fold domain might mediate the function of SAMD9 in the inhibition of RNA virus reproduction [15].

We mapped the known SAMD9 mutations onto the conserved core architecture of the SAMD9 family (see Additional file 2). Of particular interest were the MIRAGE syndrome mutations [7] that cause amino acid substitutions in poorly conserved portions of the SIR2-like and P-loop NTPase domains as well as segments with no recognizable similarity to known domains. Conceivably, even subtle alterations of the SAMD9 activity can have serious phenotypic consequences. In an attempt on a finer mapping of the mutations in SAMD9, we examined the alignment of the mammalian members of the SAMD9 branch proper. These sequences are, on average, about 75% identical, and in this case, 13 of the 14 mutations were in strictly conserved positions indicating that the proteins have not diverged far enough to allow functional inferences (see Additional file 4).

The functions of the bacterial members of the SAMD9 family are harder to predict than those of the animal members but the presence of kinase domains as well as TPR repeats implies roles in signal transduction, in a parallel to the bacterial members of the STAND superfamily [21, 26]. Furthermore, the phyletic pattern of the SAMD9 superfamily suggest the possibility that the respective genes were acquired by animals via horizontal transfer from bacteria, again analogously to the STAND NTPases [21, 26].

In conclusion, the elucidation of the complex domain architecture of the SAMD9 family proteins and the prediction that they mediate antivirus and antitumor response via the formation of a distinct, apoptosome-like particle, NTP-dependent signaling and cytoplasmic DNA sensing opens an entire new direction for experimental study of the predicted novel defense network.

## Reviewers’ comments

### Reviewer 1: Igor B. Zhulin, University of Tennessee

The human SAMD9 protein is expressed in a wide range of tissues and although its function is not fully understood, it is implicated in familial tumoral calcinosis and the MIRAGE syndrome. Thus, understanding its function is critical for understanding these diseases. In this paper, Mekhedov et al. reported evolutionary history, phyletic distribution and complete domain architecture of SAMD9 homologs including representatives of Bacteria. Analysis of domain architecture in the context of available knowledge on individual domains allowed the authors to propose that SAMD9 family proteins mediate antitumor and antivirus responses and to provide testable hypotheses that can drive future experimental work. Overall, computational analysis presented here is sound and all conclusions and suggestions are well justified. Last, but not least, the paper was interesting to read.

The evolutionary history of the SAMD9 history is quite peculiar. In contrast to previous reports, the authors identified SAMD9 homologs outside vertebrates (specifically in several lower metazoan lineages) and in bacteria and suggested that animals acquired the respective genes by horizontal gene transfer. The presence of SAMD9 in Cnidaria, but its absence in several metazoan lineages (e.g. insects, nematodes) suggests that either

SAMD9 acquired by the lower metazoan ancestor was subsequently lost in these lineages or there were several independent acquisitions. While the exact answer might be both difficult to obtain and unnecessary for the purpose of this study, I think it is important to stress that SAMD9 is missing from these lineages. This might become biologically relevant at some point in the future, especially because *Drosophila* and *C. elegans* are models for experimental biology.

Authors’ response: *We agree, it is a good idea to emphasize that SAMD9 is missing in nematodes and insects. This is mentioned in the revised text.*

P-loop NTPase domain and TPR repeats occur in a large number of various proteins in bacteria, not just in those involved in signal transduction.

While the authors were careful in predicting potential function of SAMD9 in bacteria, their suggestion that these proteins play a role in signal transduction (based on the presence of the serine/threonine kinase domain) could be further supported by the fact that the P-loop-NTPase domain (e.g. in sigma 54 transcriptional regulators that are found in bacterial two-component systems) and TPR repeats (found in numerous bacterial signaling systems) are both common to bacterial signal transduction.

Authors’ response: *Yes, there are many P-loop NTPases fused to TPR repeats in bacteria and some archaea that remain largely uncharacterized and quite likely, could be involved in various forms of signal transduction. This was already pointed out in the original manuscript, and the diagnostic presence of the TPR repeats was further emphasized in the revision.*

The only part of the paper, where I think some further digging might be fruitful is the analysis of known disease-causing mutations in human SAMD9 (lines 171-176 and Additional file 2). The authors concluded that some of these mutations occurred in poorly conserved positions. This might as well be true, but to better clarify this point, I suggest including only sequences of reliably identified orthologs of the human SAMD9 (that would be the upper segment of the tree shown on Fig. 1, which includes sequences with exactly the same domain architecture - mammals to fish, marked by a vertical bar, but excluding SAMD9L paralogs). We had recently performed a similar study of the human NPC1 protein and showed that excluding paralogs, such as NPC1L1, and distant homologs substantially improves the fidelity of such analysis [32]. Clearly, even in the dataset that includes all homologs (Additional file 2), it is apparent that some of disease-causing substitutions are “evolutionarily impermissible”, e.g. K764E, D769N, S838L, but for some other cases it might be useful to check whether a change that seems “allowable”, e.g. E457K, might become “impermissible”, when paralogs and distant homologs are removed. As the authors rightfully concluded, these distant homologs might have adopted functions that might be different from that of the human SAMD9…

Authors’ response: *We followed the reviewer’s advice to explore the conservation of amino acids changed in documented SAMD9 mutations. As suggested by the reviewer, we generated a separate multiple alignment for the SAMD9 branch that included 7 mammalian sequences, which are one to one orthologs. Almost all mutations reported for SAMD9 previously fall into positions that are strictly conserved in these proteins (see new Additional file* *4* *). However, so are about 75% of all positions in the alignment, indicating that mammalian sequences have not diverged far enough to make such analysis informative for functional inference.*

### Reviewer 2: Mensur Dlakic, Montana State University

In this manuscript Mekhedov et al. present a comprehensive analysis of SAMD9 family proteins, and show that several domains forming the core of these proteins are more widespread than previously known. This is an important contribution to the field.

The authors describe a series of logical and well-executed inquiries picking apart domain organizations and evolutionary distributions of SAMD9-like proteins. Their results are clear and I agree with the hypotheses they derived for the most part.

One conclusion that could be expanded is that both Alba and OB domains function by binding RNA rather than DNA and RNA, respectively. It is well known that Alba proteins participate in RNA metabolism (reviewed in [31])

Authors’ response: *We have modified the text accordingly and cite the Goyal* et al.*, 2016* [31] *paper. However, considering that archaeal SIR2 interacts with the major chromatin protein Alba by binding and deacetylating its lysine residues, which stimulates AlbA binding to DNA and turns off gene expression, it appears likely that ALBA domain in SAMD9 actually binds DNA as also indicated in the revised text.*

I would much rather see a raw alignment instead of, or at least along with, Additional file 3. I suggest to take this statement out of parentheses and make it a normal follow-up sentence: “(a notable difference, however, is the apparent absence of the SAMD9 family proteins in plants)”

Authors’ response: *Additional file* *3* *is a raw alignment. Not a single residue has been removed. It can be readily seen by opening the respective MS Word file in the Read-Only mode and switch to VIEW-Layout-Paper layout.*

## Additional files

Additional file 1: Table S1. Database search results with the sequences of SAMD9 family members as queries. (DOCX 24 kb)

Additional file 2: Figure S1. Sequence logo of the multiple alignment of 208 amino acid sequences from SAMD9 family including metazoan and bacterial species. (PDF 3644 kb)

Additional file 3: Figure S2. Multiple alignment of the SAMD9 family proteins. (DOCX 318 kb)

Additional file 4: Figure S3. Sequence logo of the multiple alignment of 7 amino acid sequences of mammalian SAMD9 orthologs. (PPTX 1792 kb)

## Abbreviations

NTP: Nucleoside triphosphate
OB: Oligomer-binding domain
SAM: Sterile alpha motif domain
STAND: Signal transduction ATPases with numerous domains
