# Supplementary material for: The complex domain architecture of SAMD9 family proteins, predicted STAND-like NTPases, suggests new links to inflammation and apoptosis
Source: Biol Direct. 2017 May 25;12:13. doi: 10.1186/s13062-017-0185-2 (PMC5445408; doi:10.1186/s13062-017-0185-2)
Supplement: Supplementary file 1 — Database search results with the sequences of SAMD9 family members as queries. (DOCX 24 kb) [file 13062_2017_185_MOESM1_ESM.docx]

**Sequence similarity search results for selected proteins of the SAMD9 family**

| **Query ID and species** | **Method** | **Hit ID and annotation** | **Positions in the query sequence** | **HHpred probablity (Pr)** | **Conclusion/comment** |
| --- | --- | --- | --- | --- | --- |
| NP_060124.2 Homo sapiens | HHpred | SAM_Samd9_Samd9L SAM domain of Samd9/Samd9L subfamily | 12-75 | Pr=99.32 | Family forming hit – SAM protein-protein interaction domain |
| NP_060124.2 Homo sapiens | HHpred | PF04326 AlbA_2: Putative DNA-binding domain | 195-352 | Pr=98.20 | AlbA_2 Putative DNA-binding domain |
| NP_060124.2 Homo sapiens | HHpred | PF13289 SIR2_2: SIR2-like domain | 437-581 | Pr=98.26 | Variant SIR2_2 domain |
| NP_060124.2 Homo sapiens | HHpred | PF00004 AAA: ATPase family associated with various cellular activities (AAA) | 733-837 | Pr=97.68 | Variant AAA ATPase family domain |
| NP_060124.2 Homo sapiens | HHpred | 1z6t_A APAF-1, apoptotic protease activating factor 1; caspase activation, ADP, nucleotide binding, CARD, apoptosis; HET: ADP; 2.21A {Homo sapiens} | 712-998 | Pr=96.79 | Key hit of the core of SAMD9 family |
| NP_060124.2 Homo sapiens | HHpred | 3cam_A Cold-shock domain family protein; cold shock protein, chain SWAP {Neisseria meningitidis MC58} SCOP: b.40.4.5 | 1529-1589 | Pr=91.49 | OB-fold domain |
| XP_007231320.1 Astyanax mexicanus | HHpred | PF00004 AAA: ATPase family associated with various cellular activities (AAA) | 95-198 | Pr=97.69 | Variant AAA ATPase family domain |
| XP_007231320.1 Astyanax mexicanus | HHpred | PF13553 FIIND | 954-1200 | Pr=100.00 | FIIND domain |
| XP_007231320.1 Astyanax mexicanus | HHpred | cdd CARD_ASC_NALP1 | 1221-1305 | Pr=99.58 | CARD domain |
| XP_017550536.1 Pygocentrus nattereri | HHpred | cd09579 SAM_Samd7,11 SAM domain of Samd7,11 subfamily of Polycomb group | 23-87 | Pr=98.29 | Family forming hit – SAM protein-protein interaction domain |
| XP_017550536.1 Pygocentrus nattereri | HHpred | PF02758 PYRIN: PAAD/DAPIN/Pyrin domain | 124-199 | Pr=98.69 | PYRIN domain |
| XP_017550536.1 Pygocentrus nattereri | HHpred | PF13289 SIR2_2: SIR2-like domain | 270-434 | Pr=98.36 | Variant SIR2_2 domain |
| XP_017550536.1 Pygocentrus nattereri | HHpred | PF00004 AAA: ATPase family associated with various cellular activities (AAA) | 559-664 | Pr=97.74 | Variant AAA ATPase family domain |
| CDQ83481.1 Oncorhynchus mykiss | HHpred | PF00004 AAA: ATPase family associated with various cellular activities (AAA) | 106-205 | Pr=98.05 | Variant AAA ATPase family domain |
| CDQ83481.1 Oncorhynchus mykiss | HHpred | PF14559 TPR_19: Tetratricopeptide repeat | 446-510 | Pr=97.13 | TPR repeats |
| XP_003727489.1 Strongylocentrotus purpuratus | HHpred | 5j8y_A Polycomb protein SCM; PRC1 phorc SAM domain polycom response element | 139-200 | Pr=96.91 | Family forming hit – SAM protein-protein interaction domain |
| XP_003727489.1 Strongylocentrotus purpuratus | HHpred | PF13289 SIR2_2: SIR2-like domain | 348-483 | Pr=98.12 | Variant SIR2_2 domain |
| XP_006825609.1 Saccoglossus kowalevskii | HHpred | 3sei_A Caskin-1; SAM domain, protein-protein interaction, signaling protein | 194-345 | Pr=98.09 | Family forming hit – SAM protein-protein interaction domain |
| XP_006825609.1 Saccoglossus kowalevskii | HHpred | PF04326 AlbA_2: Putative DNA-binding domain | 719-877 | Pr=98.06 | AlbA_2 Putative DNA-binding domain |
| XP_006825609.1 Saccoglossus kowalevskii | HHpred | cd01406 SIR2-like Sir2-like | 959-1107 | Pr=98.01 | Variant SIR2_2 domain |
| XP_006825609.1 Saccoglossus kowalevskii | HHpred | PF00004 AAA: ATPase family associated with various cellular activities (AAA) | 1261-1363 | Pr=97.74 | Variant AAA ATPase family domain |
| XP_006825609.1 Saccoglossus kowalevskii | HHpred | PF14559 TPR_19: Tetratricopeptide repeat | 1599-1659 | Pr=96.70 | TPR repeats |
| XP_011436570.1 Crassostrea gigas | HHpred | cd09528 SAM_Samd9_Samd9L SAM domain of Samd9/Samd9L subfamily | 239-197 | Pr=98.60 | Family forming hit – SAM protein-protein interaction domain |
| XP_011436570.1 Crassostrea gigas | HHpred | PF04326 AlbA_2: Putative DNA-binding domain | 380-534 | Pr=97.89 | AlbA_2 Putative DNA-binding domain |
| XP_011436570.1 Crassostrea gigas | HHpred | PF13289 SIR2_2: SIR2-like domain | 619-763 | Pr=98.11 | Variant SIR2_2 domain |
| XP_011436570.1 Crassostrea gigas | HHpred | PF00004 AAA: ATPase family associated with various cellular activities (AAA) | 916-1016 | Pr=97.84 | Variant AAA ATPase family domain |
| KYO30992.1 Alligator mississippiensis | HHpred | PF04326 AlbA_2: Putative DNA-binding domain | 179-340 | Pr=98.11 | AlbA_2 Putative DNA-binding domain |
| KYO30992.1 Alligator mississippiensis | HHpred | PF13289 SIR2_2: SIR2-like domain | 422-586 | Pr=98.72 | Variant SIR2_2 domain |
| KYO30992.1 Alligator mississippiensis | HHpred | PF00004 AAA: ATPase family associated with various cellular activities (AAA) | 710-815 | Pr=97.71 | Variant AAA ATPase family domain |
| KYO30992.1 Alligator mississippiensis | HHpred | PF14559 TPR_19: Tetratricopeptide repeat | 1039-1102 | Pr=96.73 | TPR repeats |
| XP_004558390.1 Maylandia zebra | HHpred | cd09528 SAM_Samd9_Samd9L SAM domain of Samd9/Samd9L subfamily | 19-64 | Pr=99.29 | Family forming hit – SAM protein-protein interaction domain |
| XP_004558390.1 Maylandia zebra | HHpred | PF04326 AlbA_2: Putative DNA-binding domain | 186-344 | Pr=98.06 | AlbA_2 Putative DNA-binding domain |
| XP_004558390.1 Maylandia zebra | HHpred | cd01406 SIR2-like Sir2-like | 416-567 | Pr=98.39 | Variant SIR2_2 domain |
| XP_004558390.1 Maylandia zebra | HHpred | PF00004 AAA: ATPase family associated with various cellular activities (AAA) | 715-818 | Pr=97.78 | Variant AAA ATPase family domain |
| XP_007886940.1 Callorhinchus milii | HHpred | PF04326 AlbA_2: Putative DNA-binding domain | 52-121 | Pr=98.23 | AlbA_2 Putative DNA-binding domain |
| XP_007886940.1 Callorhinchus milii | HHpred | cd01406 SIR2-like Sir2-like | 273-434 | Pr=98.45 | Variant SIR2_2 domain |
| XP_007886940.1 Callorhinchus milii | HHpred | PF00004 AAA: ATPase family associated with various cellular activities (AAA) | 568-673 | Pr=97.88 | Variant AAA ATPase family domain |
| XP_007886940.1 Callorhinchus milii | HHpred | PF14559 TPR_19: Tetratricopeptide repeat | 881-944 | Pr=96.99 | TPR repeats |
| XP_007886940.1 Callorhinchus milii | HHpred | 3cam_A Cold-shock domain family protein | 1297-1354 | Pr=89.79 | OB-fold domain |
| WP_041219555.1 Desulfitobacterium dichloroeliminans | HHpred | PF13289 SIR2_2: SIR2-like domain | 206-349 | Pr=99.36 | Variant SIR2_2 domain |
| WP_041219555.1 Desulfitobacterium dichloroeliminans | HHpred | PF00004 AAA: ATPase family associated with various cellular activities (AAA) | 460-549 | Pr=98.10 | Variant AAA ATPase family domain |
| KMO66808.1 Mycobacterium chubuense | HHpred | 1nd4_A Aminoglycoside 3'-phosphotransferase; protein kinase, ATPase | 37-280 | Pr=97.87 | Protein kinase-like domain |
| KMO66808.1 Mycobacterium chubuense | HHpred | PF00004 AAA: ATPase family associated with various cellular activities (AAA) | 698-786 | Pr=97.81 | Variant AAA ATPase family domain |
| XP_013415988.1 Lingula anatina | HHpred | PF00619 CARD: Caspase recruitment domain | 78-162 | Pr=97.22 | CARD domain |
| XP_013415988.1 Lingula anatina | HHpred | PF04326 AlbA_2: Putative DNA-binding domain | 451-588 | Pr=96.68 | AlbA_2 Putative DNA-binding domain |
| XP_013415988.1 Lingula anatina | HHpred | PF13289 SIR2_2: SIR2-like domain | 662-811 | Pr=98.61 | Variant SIR2_2 domain |
| XP_013415988.1 Lingula anatina | HHpred | 1z6t_A APAF-1, apoptotic protease activating factor 1 | 913-1188 | Pr=97.27 | Key hit of the core of SAMD9 family |
| XP_013415988.1 Lingula anatina | HHpred | PF14559 TPR_19: Tetratricopeptide repeat | 1268-1362 | Pr=95.58 | TPR repeats |
| XP_013415988.1 Lingula anatina | HHpred | pfam00313 CSD 'Cold-shock' DNA-binding domain | 1709-1778 | Pr=94.42 | OB-fold domain |
| XP_018668123.1 Ciona intestinalis | HHpred | PF13289 SIR2_2: SIR2-like domain | 215-372 | Pr=99.20 | Variant SIR2_2 domain |
| XP_018668123.1 Ciona intestinalis | HHpred | PF00004 AAA: ATPase family associated with various cellular activities (AAA) | 489-578 | Pr=97.74 | Variant AAA ATPase family domain |
| XP_011440496.1 Crassostrea gigas | HHpred | [PF13289](http://pfam.xfam.org/family?acc=PF13289) SIR2_2: SIR2-like domain | 505-665 | Pr=98.59 | Variant SIR2_2 domain |
| XP_011440496.1 Crassostrea gigas | HHpred | [PF00004](http://pfam.xfam.org/family?acc=PF00004) AAA: ATPase family associated with various cellular activities (AAA) | 785-877 | Pr=97.72 | Variant AAA ATPase family domain |
| XP_011440496.1 Crassostrea gigas | HHpred | PF14559 TPR_19: Tetratricopeptide repeat | 1130-1214 | Pr=98.21 | TPR repeats |
| XP_011440496.1 Crassostrea gigas | HHpred | pfam00313 CSD 'Cold-shock' DNA-binding domain | 1556-1625 | Pr=95.30 | OB-fold domain |
| EEN48978.1 Branchiostoma floridae | HHpred | cd08319 Death_RAIDD Death domain of RIP-associated ICH-1 homologous protein with a death domain | 251-333 | Pr=98.59 | Death domain |
| EEN48978.1 Branchiostoma floridae | HHpred | PF00004 AAA: ATPase family associated with various cellular activities (AAA) | 416-510 | Pr=97.75 | Variant AAA ATPase family domain |
| EEN48978.1 Branchiostoma floridae | HHpred | 3cv0_A Peroxisome targeting signal 1 receptor PEX5; TPR motifs | 758-1011 | Pr=97.76 | TPR repeats |
| XP_015751629.1 Acropora digitifera | HHpred | 4m59_A Chloroplast pentatricopeptide repeat protein 10; pentatricopeptide repeats, superhelical, RNA binding  protein RNA binding protein-RNA complex; 2.46A {Zea mays} | 11-394 | Pr=99.91 | Uncharacerised proline rich repeat, not shown in the architecture |
| XP_015751629.1 Acropora digitifera | HHpred | 3c48_A Predicted glycosyltransferases | 425-804 | Pr=99.73 | Glycosyltransferase domain is present in close homologs from other Cnidaria species |
| XP_015751629.1 Acropora digitifera | HHpred | PF13289 SIR2_2: SIR2-like domain | 859-1011 | Pr=99.62 | Variant SIR2_2 domain |
| XP_015751629.1 Acropora digitifera | HHpred | 1z6t_A APAF-1, apoptotic protease activating factor 1; caspase activation, ADP, nucleotide binding, CARD, apoptosis; HET: ADP; 2.21A {Homo sapiens} | 1101-1388 | Pr=98.96 | Key hit of the core of SAMD9 family |
| XP_015751629.1 Acropora digitifera | HHpred | 3cv0_A Peroxisome targeting signal 1 receptor PEX5; TPR motifs, TPR protein, peroxin 5, PEX5, PTS1 binding domain, protein-peptide complex, receptor; 2.00A {Trypanosoma brucei} | 1451-1766 | Pr=99.88 | TPR repeats |
| WP_006849212.1 Prevotella copri | HHpred | PF13289 SIR2_2: SIR2-like domain | 255-403 | Pr=99.29 | Variant SIR2_2 domain |
| WP_006849212.1 Prevotella copri | HHpred | PF13173 AAA_14: AAA domain | 504-628 | Pr=97.84 | Variant AAA ATPase family domain |
| KXJ11435.1 Exaiptasia pallida | HHpred | PF00004 AAA: ATPase family associated with various cellular activities (AAA) | 801-902 | Pr=97.51 | Variant AAA ATPase family domain |
| KXJ11435.1 Exaiptasia pallida | HHpred | 4r7s_A Tetratricopeptide repeat protein | 1129-1415 | Pr=96.93 | TPR repeats |
| KXJ11435.1 Exaiptasia pallida | HHpred | pfam00313 CSD 'Cold-shock' DNA-binding domain | 1552-1628 | Pr=97.98 | OB-fold domain |
| WP_006128369.1 Streptomyces | HHpred | PRK05231 homoserine kinase | 32-398 | Probab=99.84 | Protein kinase-like domain |
| WP_006128369.1 Streptomyces | HHpred | 2a5y_B CED-4; apoptosis; HET: ATP; 2.60A {Caenorhabditis elegans} SCOP: a.4.5.80 a.77.1.3 c.37.1.20 | 716-995 | Probab=99.16 | Variant AAA ATPase family domain |
| WP_006128369.1 Streptomyces | HHpred | 4r7s_A Tetratricopeptide repeat protein | 1053-1351 | Probab=99.11 | TPR repeats |
| WP_006128369.1 Streptomyces | HHpred | 1c9o_A CSPB, cold-shock protein; beta barrel, homodimer, transcription; 1.17A {Bacillus caldolyticus} SCOP: b.40.4.5 | 1438-1485 | Probab=49.67 | Diverged OB-fold domain |
| WP_006128369.1 Streptomyces | HHpred | 2lxj_A CSPL, cold shock-like protein CSPLA; nucleic acids, transcription; NMR {Listeria monocytogenes} SCOP: b.40.4.5 | 1646-1704 | Probab=62.41 | Diverged OB-fold domain |
|  |  |  |  |  |  |

HHpred – defalt parameters with HHblits, against pdb70_12Feb17, cdd_04Jul16, pfam_30.0, and smart_04Jul16 databases
