# Supplementary material for: The complex domain architecture of SAMD9 family proteins, predicted STAND-like NTPases, suggests new links to inflammation and apoptosis
Source: Biol Direct. 2017 May 25;12:13. doi: 10.1186/s13062-017-0185-2 (PMC5445408; doi:10.1186/s13062-017-0185-2)
Supplement: Supplementary file 2 — Sequence logo of the multiple alignment of 208 amino acid sequences from SAMD9 family including metazoan and bacterial species. (PDF 3644 kb) [file 13062_2017_185_MOESM2_ESM.pdf]

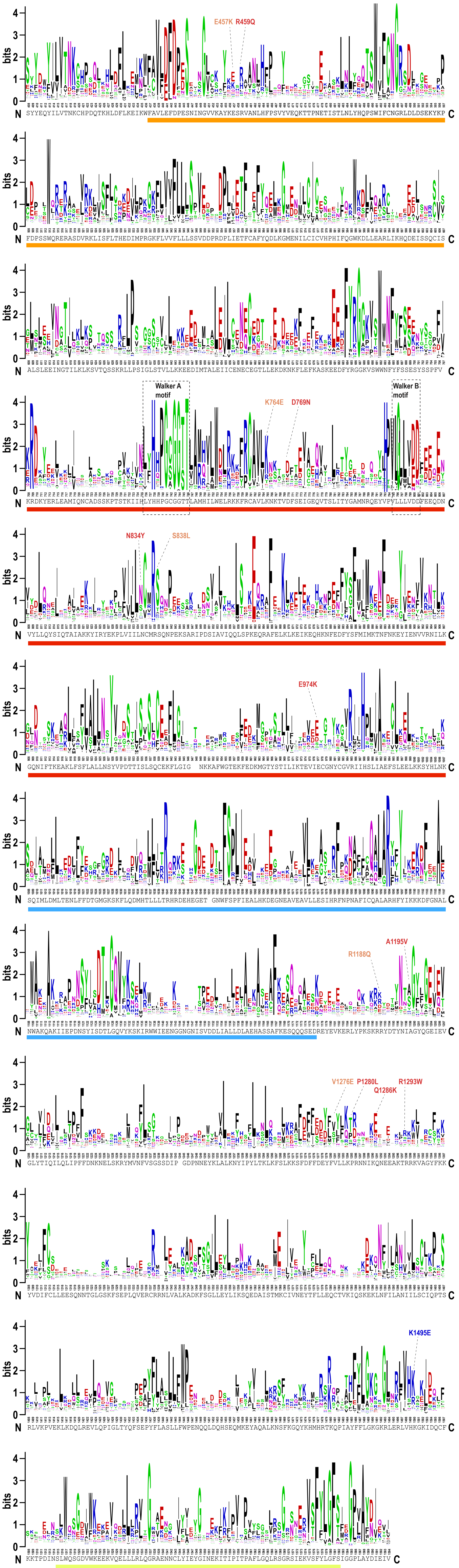
E457K
R459Q
bits
Walker A motif
Walker B motif
K764E
D769N
N834Y
S838L
E974K
R1188Q
A1195V
V1276E
P1280L
Q1286K
R1293W
K1495E

To focus on the conservation of human proteins and to avoid signals from amino acids corresponding to inserts that are absent in human SAMD9 and/or SAMD9-like, only positions represented in these two proteins were kept in the alignment. Numbers correspond to the coordinates of the alignment of human SAMD9 and SAMD9-like starting from position 408 in SAMD9. Beneath the logo the SAMD9 sequence is shown. Colored bars beneath the sequence correspond to detected or predicted domains: orange - variant SIR2_2 domain, red - P-loop NTPase, blue - TPR repeats, yellow - OB-fold. Positions of 14 amino acid changes reported for SAMD9 are: E457K, K764E, S838L, R1188Q, V1276E from ExAC database (PubMed: 27182967); R459Q, D769N, N834Y, E974K, A1195V, P1280L, Q1286K, R1293W from MIRAGE syndrome patients (PubMed: 27182967) and K1495E from normophosphatemic familial tumoral calcinosis patient (PubMed: 16960814). Positions of the Walker A and B motifs are indicated.
