# Supplementary material for: The complex domain architecture of SAMD9 family proteins, predicted STAND-like NTPases, suggests new links to inflammation and apoptosis
Source: Biol Direct. 2017 May 25;12:13. doi: 10.1186/s13062-017-0185-2 (PMC5445408; doi:10.1186/s13062-017-0185-2)
Supplement: Supplementary file 3 — Multiple alignment of the SAMD9 family proteins. (DOCX 318 kb) [file 13062_2017_185_MOESM3_ESM.docx]

* 20 * 40 * 60 * 80 * 100 * 120

Homsa_NP_060124.2 : ------------------------------------------------------------------------------------------------------------------------- : -

Homsa_NP_689916.2 : ------------------------------------------------------------------------------------------------------------------------- : -

fish_XP_015241820.1 : ------------------------------------------------------------------------------------------------------------------------- : -

fish_XP_018543893.1 : ------------------------------------------------------------------------------------------------------------------------- : -

fish_CDQ83481.1 : ------------------------------------------------------------------------------------------------------------------------- : -

fish_CDQ89582.1 : ------------------------------------------------------------------------------------------------------------------------- : -

fish_XP_005809248.1 : ------------------------------------------------------------------------------------------------------------------------- : -

fish_XP_007231320.1 : ------------------------------------------------------------------------------------------------------------------------- : -

fish_XP_007254667.1 : ------------------------------------------------------------------------------------------------------------------------- : -

fish_XP_008277954.1 : ------------------------------------------------------------------------------------------------------------------------- : -

fish_XP_010746900.1 : ------------------------------------------------------------------------------------------------------------------------- : -

fish_XP_012685053.1 : ------------------------------------------------------------------------------------------------------------------------- : -

fish_XP_013123332.1 : ------------------------------------------------------------------------------------------------------------------------- : -

fish_XP_014011897.1 : ------------------------------------------------------------------------------------------------------------------------- : -

fish_XP_014011904.1 : ------------------------------------------------------------------------------------------------------------------------- : -

fish_XP_014264868.1 : ------------------------------------------------------------------------------------------------------------------------- : -

fish_XP_014264875.1 : ------------------------------------------------------------------------------------------------------------------------- : -

fish_XP_014854842.1 : ------------------------------------------------------------------------------------------------------------------------- : -

fish_XP_017578465.1 : ------------------------------------------------------------------------------------------------------------------------- : -

fish_XP_012713132.1 : ------------------------------------------------------------------------------------------------------------------------- : -

fish_XP_010787190.1 : ------------------------------------------------------------------------------------------------------------------------- : -

fish_XP_017319342.1 : ------------------------------------------------------------------------------------------------------------------------- : -

Alligator_KYO30992.1 : ------------------------------------------------------------------------------------------------------------------------- : -

Alligator_XP_006025954.1 : ------------------------------------------------------------------------------------------------------------------------- : -

amphibia_XP_018413082.1 : ------------------------------------------------------------------------------------------------------------------------- : -

bact_AGA70736.1 : ------------------------------------------------------------------------------------------------------------------------- : -

bact_CUJ86040.1 : ------------------------------------------------------------------------------------------------------------------------- : -

bact_ESW58583.1 : ------------------------------------------------------------------------------------------------------------------------- : -

bact_GAC67715.1 : ------------------------------------------------------------------------------------------------------------------------- : -

bact_KMO66808.1 : ------------------------------------------------------------------------------------------------------------------------- : -

bact_KUO79116.1 : ------------------------------------------------------------------------------------------------------------------------- : -

bact_KYG06363.1 : ------------------------------------------------------------------------------------------------------------------------- : -

bact_ODA70488.1 : ------------------------------------------------------------------------------------------------------------------------- : -

bact_OGP49286.1 : ------------------------------------------------------------------------------------------------------------------------- : -

bact_SCF05533.1 : ------------------------------------------------------------------------------------------------------------------------- : -

bact_SCX17622.1 : ------------------------------------------------------------------------------------------------------------------------- : -

bact_SFJ80976.1 : ------------------------------------------------------------------------------------------------------------------------- : -

bact_WP_006128369.1 : ----------------------------------------------------------------------------------------------------------------------MMS : 3

bact_WP_006365791.1 : ------------------------------------------------------------------------------------------------------------------------- : -

bact_WP_006849212.1 : ------------------------------------------------------------------------------------------------------------------------- : -

bact_WP_007897046.1 : ------------------------------------------------------------------------------------------------------------------------- : -

bact_WP_030194308.1 : ------------------------------------------------------------------------------------------------------------------------- : -

bact_WP_030198971.1 : ------------------------------------------------------------------------------------------------------------------------- : -

bact_WP_030898614.1 : ------------------------------------------------------------------------------------------------------------------------- : -

bact_WP_041219555.1 : ------------------------------------------------------------------------------------------------------------------------- : -

bact_WP_050505446.1 : ------------------------------------------------------------------------------------------------------------------------- : -

bact_WP_055538636.1 : ------------------------------------------------------------------------------------------------------------------------- : -

bact_WP_062134708.1 : ------------------------------------------------------------------------------------------------------------------------- : -

bact_WP_062799339.1 : ------------------------------------------------------------------------------------------------------------------------- : -

bact_WP_066463897.1 : ------------------------------------------------------------------------------------------------------------------------- : -

bird_XP_002194798.1 : ------------------------------------------------------------------------------------------------------------------------- : -

bird_XP_005010984.1 : ------------------------------------------------------------------------------------------------------------------------- : -

bird_XP_005505023.1 : ------------------------------------------------------------------------------------------------------------------------- : -

bird_XP_008492486.1 : ------------------------------------------------------------------------------------------------------------------------- : -

bird_XP_008628214.1 : ------------------------------------------------------------------------------------------------------------------------- : -

bird_XP_009068034.1 : ------------------------------------------------------------------------------------------------------------------------- : -

bird_XP_009080343.1 : ------------------------------------------------------------------------------------------------------------------------- : -

bird_XP_009486154.1 : ------------------------------------------------------------------------------------------------------------------------- : -

bird_XP_009640122.1 : ------------------------------------------------------------------------------------------------------------------------- : -

bird_XP_009672623.1 : ------------------------------------------------------------------------------------------------------------------------- : -

bird_XP_009695665.1 : MKLIKDHEVYRGGEEACKTALSAAAGNAEHATHIASASRAVLRPSVTEGEEPLVFHDPATLSSSLLTLDHLKESREHYRNSHSAHTPARTTEPAGWQSLEPLLPSPPLETSLEGRIWPRHW : 121

bird_XP_009886214.1 : ------------------------------------------------------------------------------------------------------------------------- : -

bird_XP_009934438.1 : ------------------------------------------------------------------------------------------------------------------------- : -

bird_XP_009999877.1 : ------------------------------------------------------------------------------------------------------------------------- : -

bird_XP_010119033.1 : ------------------------------------------------------------------------------------------------------------------------- : -

bird_XP_010144931.1 : ------------------------------------------------------------------------------------------------------------------------- : -

bird_XP_010222910.1 : ------------------------------------------------------------------------------------------------------------------------- : -

bird_XP_013160560.1 : ------------------------------------------------------------------------------------------------------------------------- : -

bird_XP_014795808.1 : ------------------------------------------------------------------------------------------------------------------------- : -

bird_XP_015156587.1 : ------------------------------------------------------------------------------------------------------------------------- : -

bird_XP_015709021.1 : ------------------------------------------------------------------------------------------------------------------------- : -

brachiop_XP_013415988.1 : ---------------------------------------------------------------------------------------------------------------------MADQ : 4

Cioin_XP_018668123.1 : ------------------------------------------------------------------------------------------------------------------------- : -

Cnidaria_EDO35430.1 : ------------------------------------------------------------------------------------------------------------------------- : -

Cnidaria_KXJ09690.1 : ------------------------------------------------------------------------------------------------------------------------- : -

Cnidaria_KXJ11435.1 : ------------------------------------------------------------------------------------------------------------------------- : -

Cnidaria_KXJ17955.1 : ------------------------------------------------------------------------------------------------------------------------- : -

Cnidaria_KXJ23466.1 : ------------------------------------------------------------------------------------------------------------------------- : -

Cnidaria_XP_015751626.1 : ------------------------------------------------------------------------------------------------------------------------- : -

Cnidaria_XP_015751629.1 : ------------------------------------------------------------------------------------------------------------------------- : -

Cnidaria_XP_015758975.1 : -----------------------------------------------------------------------------------------------------MHICSFDVLLGPVLFFLQRK : 20

Cnidaria_XP_015766106.1 : ------------------------------------------------------------------------------------------------------------------------- : -

Cnidaria_XP_015766118.1 : ------------------------------------------------------------------------------------------------------------------------- : -

Cnidaria_XP_015766124.1 : ------------------------------------------------------------------------------------------------------------------------- : -

coelacanth_XP_006000513.2 : ------------------------------------------------------------------------------------------------------------------------- : -

fish_CDQ87765.1 : --------MVIKPLSNNHDTIQITSSYEKLKFREPECWCGDRMSDTTVSPEGAESGKSEHIRQWLHSSPTAPQEYNEDVAGSSLLCPGKQGLVAQEERAESPSCLSMKSDHSMGQPINFHR : 113

fish_CDQ92295.1 : ------------------------------------------------------------------------------------------------------------------------- : -

fish_KKF13953.1 : ------------------------------------------------------------------------------------------------------------------------- : -

fish_XP_004085798.2 : -------------------------------------------------------------------------------------------------------------------MLKCLC : 6

fish_XP_004552746.3 : ------------------------------------------------------------------------------------------------------------------------- : -

fish_XP_004558390.1 : ------------------------------------------------------------------------------------------------------------------------- : -

fish_XP_004576250.1 : ------------------------------------------------------------------------------------------------------------------------- : -

fish_XP_005161888.1 : ------------------------------------------------------------------------------------------------------------------------- : -

fish_XP_005478813.2 : ------------------------------------------------------------------------------------------------------------------------- : -

fish_XP_005806405.2 : ------------------------------------------------------------------------------------------------------------------------- : -

fish_XP_006625674.1 : ------------------------------------------------------------------------------------------------------------------------- : -

fish_XP_006635547.1 : ------------------------------------------------------------------------------------------------------------------------- : -

fish_XP_006635548.1 : ------------------------------------------------------------------------------------------------------------------------- : -

fish_XP_006791552.1 : ------------------------------------------------------------------------------------------------------------------------- : -

fish_XP_006791631.1 : ------------------------------------------------------------------------------------------------------------------------- : -

fish_XP_007241837.1 : ------------------------------------------------------------------------------------------------------------------------- : -

fish_XP_007242299.1 : ------------------------------------------------------------------------------------------------------------------------- : -

fish_XP_007557414.1 : ------------------------------------------------------------------------------------------------------------------------- : -

fish_XP_008284284.1 : ------------------------------------------------------------------------------------------------------------------------- : -

fish_XP_008284762.1 : ------------------------------------------------------------------------------------------------------------------------- : -

fish_XP_008285761.1 : ------------------------------------------------------------------------------------------------------------------------- : -

fish_XP_008336142.1 : ------------------------------------------------------------------------------------------------------------------------- : -

fish_XP_008417602.1 : ------------------------------------------------------------------------------------------------------------------------- : -

fish_XP_009510531.1 : ------------------------------------------------------------------------------------------------------------------------- : -

fish_XP_010752845.1 : ------------------------------------------------------------------------------------------------------------------------- : -

fish_XP_010755274.1 : ------------------------------------------------------------------------------------------------------------------------- : -

fish_XP_010767524.1 : ------------------------------------------------------------------------------------------------------------------------- : -

fish_XP_010781233.1 : ----------------------------------------------------------------------------------------------------------------------MIR : 3

fish_XP_010791966.1 : ------------------------------------------------------------------------------------------------------------------------- : -

fish_XP_012687177.1 : ------------------------------------------------------------------------------------------------------------------------- : -

fish_XP_012708385.1 : ------------------------------------------------------------------------------------------------------------------------- : -

fish_XP_012713253.1 : ------------------------------------------------------------------------------------------------------------------------- : -

fish_XP_012727798.1 : ------------------------------------------------------------------------------------------------------------------------- : -

fish_XP_012990558.1 : ------------------------------------------------------------------------------------------------------------------------- : -

fish_XP_013126906.1 : ------------------------------------------------------------------------------------------------------------------------- : -

fish_XP_013796981.1 : ------------------------------------------------------------------------------------------------------------------------- : -

fish_XP_014005049.1 : ------------------------------------------------------------------------------------------------------------------------- : -

fish_XP_014049585.1 : ------------------------------------------------------------------------------------------------------------------------- : -

fish_XP_014325953.1 : ------------------------------------------------------------------------------------------------------------------------- : -

fish_XP_015195770.1 : ------------------------------------------------------------------------------------------------------------------------- : -

fish_XP_015197335.1 : ------------------------------------------------------------------------------------------------------------------------- : -

fish_XP_015232478.1 : ------------------------------------------------------------------------------------------------------------------------- : -

fish_XP_015252023.1 : ------------------------------------------------------------------------------------------------------------------------- : -

fish_XP_015264177.1 : ------------------------------------------------------------------------------------------------------------------------- : -

fish_XP_015801149.1 : ------------------------------------------------------------------------------------------------------------------------- : -

fish_XP_015810486.1 : ------------------------------------------------------------------------------------------------------------------------- : -

fish_XP_016115105.1 : ------------------------------------------------------------------------------------------------------------------------- : -

fish_XP_016117315.1 : ------------------------------------------------------------------------------------------------------------------------- : -

fish_XP_016395637.1 : ------------------------------------------------------------------------------------------------------------------------- : -

fish_XP_017207716.1 : ------------------------------------------------------------------------------------------------------------------------- : -

fish_XP_017277259.1 : ------------------------------------------------------------------------------------------------------------------------- : -

fish_XP_017277301.1 : ------------------------------------------------------------------------------------------------------------------------- : -

fish_XP_017325460.1 : --------------------------------------------------------------------------------------------------------------------MAETR : 5

fish_XP_017346953.1 : ------------------------------------------------------------------------------------------------------------------------- : -

fish_XP_017550536.1 : ------------------------------------------------------------------------------------------------------------------------- : -

fish_XP_017550824.1 : ------------------------------------------------------------------------------------------------------------------------- : -

fish_XP_017554653.1 : ------------------------------------------------------------------------------------------------------------------------- : -

fish_XP_017569575.1 : ------------------------------------------------------------------------------------------------------------------------- : -

fish_XP_018413083.1 : ------------------------------------------------------------------------------------------------------------------------- : -

fish_XP_018429299.1 : ------------------------------------------------------------------------------------------------------------------------- : -

fish_XP_018521334.1 : ------------------------------------------------------------------------------------------------------------------------- : -

fish_XP_018526467.1 : ------------------------------------------------------------------------------------------------------------------------- : -

fish_XP_018541041.1 : -----------------------------------------------------------------------------------------------------------------------MD : 2

fish_XP_018558293.1 : ------------------------------------------------------------------------------------------------------------------------- : -

fish_XP_018595329.1 : ------------------------------------------------------------------------------------------------------------------------- : -

fish_XP_018611696.1 : ------------------------------------------------------------------------------------------------------------------------- : -

fish_XP_018956725.1 : ------------------------------------------------------------------------------------------------------------------------- : -

frog_XP_018091031.1 : ------------------------------------------------------------------------------------------------------------------------- : -

Hemichordata_XP_006825609.1 : --------------------------------MAVHNVLTYPPHDPMERSLWCQQQLAERRRCILSNQHITIVNKIKQSVRRRRRYPANNAQIHDTQRGNIRKKIIIKKSKVEMNTSKRPD : 89

lance_EEN48978.1 : ------------------------------------------------------------------------------------------------------------------------- : -

lance_EEN55742.1 : ------------------------------------------------------------------------------------------------------------------------- : -

mammal_XP_001378512.1 : ------------------------------------------------------------------------------------------------------------------------- : -

mammal_XP_003771993.1 : ------------------------------------------------------------------------------------------------------------------------- : -

mammal_XP_003771994.1 : ------------------------------------------------------------------------------------------------------------------------- : -

mammal_XP_003782717.1 : ------------------------------------------------------------------------------------------------------------------------- : -

mammal_XP_003982796.1 : ------------------------------------------------------------------------------------------------------------------------- : -

mammal_XP_004389713.1 : ------------------------------------------------------------------------------------------------------------------------- : -

mammal_XP_004582357.1 : ------------------------------------------------------------------------------------------------------------------------- : -

mammal_XP_004582358.1 : ------------------------------------------------------------------------------------------------------------------------- : -

mammal_XP_004622892.1 : ------------------------------------------------------------------------------------------------------------------------- : -

mammal_XP_004646313.1 : ------------------------------------------------------------------------------------------------------------------------- : -

mammal_XP_004702866.1 : ------------------------------------------------------------------------------------------------------------------------- : -

mammal_XP_005340592.1 : ------------------------------------------------------------------------------------------------------------------------- : -

mammal_XP_005388084.1 : ------------------------------------------------------------------------------------------------------------------------- : -

mammal_XP_006163552.1 : ------------------------------------------------------------------------------------------------------------------------- : -

mammal_XP_006779273.1 : ------------------------------------------------------------------------------------------------------------------------- : -

mammal_XP_006834357.1 : ------------------------------------------------------------------------------------------------------------------------- : -

mammal_XP_006891533.1 : ------------------------------------------------------------------------------------------------------------------------- : -

mammal_XP_007522555.1 : ------------------------------------------------------------------------------------------------------------------------- : -

mammal_XP_007938002.1 : ------------------------------------------------------------------------------------------------------------------------- : -

mammal_XP_008062458.1 : ------------------------------------------------------------------------------------------------------------------------- : -

mammal_XP_008259950.1 : ------------------------------------------------------------------------------------------------------------------------- : -

mammal_XP_008524371.1 : ------------------------------------------------------------------------------------------------------------------------- : -

mammal_XP_008820771.1 : ------------------------------------------------------------------------------------------------------------------------- : -

mammal_XP_010601767.1 : ------------------------------------------------------------------------------------------------------------------------- : -

mammal_XP_011853657.1 : ------------------------------------------------------------------------------------------------------------------------- : -

mammal_XP_012791283.1 : ------------------------------------------------------------------------------------------------------------------------- : -

mammal_XP_012881542.1 : ------------------------------------------------------------------------------------------------------------------------- : -

mammal_XP_012934202.1 : ------------------------------------------------------------------------------------------------------------------------- : -

mammal_XP_013013639.1 : ------------------------------------------------------------------------------------------------------------------------- : -

mammal_XP_016016798.1 : ------------------------------------------------------------------------------------------------------------------------- : -

mammal_XP_016281899.1 : ------------------------------------------------------------------------------------------------------------------------- : -

mammal_XP_017199853.1 : ------------------------------------------------------------------------------------------------------------------------- : -

mammal_XP_017508280.1 : ------------------------------------------------------------------------------------------------------------------------- : -

mollusk_EKC42356.1 : ------------------------------------------------------------------------------------------------------------------------- : -

mollusk_XP_011436570.1 : ----------------------------------------------------------------------------------------------------------------MNPEQKHKI : 9

mollusk_XP_011440496.1 : ------------------------------------------------------------------------------------------------------------------------- : -

mollusk_XP_013061423.1 : ---------------------------------------------------------------------------------------------------------------MNAHDWEKLQ : 10

mollusk_XP_013061425.1 : ------------------------------------------------------------------------------------------------------------------------- : -

Musmu_NP_034286.2 : ------------------------------------------------------------------------------------------------------------------------- : -

rept_XP_008110733.2 : ------------------------------------------------------------------------------------------------------------------------- : -

rept_XP_008112638.1 : ------------------------------------------------------------------------------------------------------------------------- : -

rept_XP_015264172.1 : -------------------------------------------------------------------------------------------------------------------MLFTFC : 6

shark_XP_007886940.1 : ------------------------------------------------------------------------------------------------------------------------- : -

Strpu_XP_003727489.1 : ------------------------------------------------------------------------------------------------------------------------- : -

Strpu_XP_011667260.1 : ------------------------------------------------------------------------------------------------------------------------- : -

turtle_XP_006139744.1 : ------------------------------------------------------------------------------------------------------------------------- : -

turtle_XP_007060369.1 : ------------------------------------------------------------------------------------------------------------------------- : -

turtle_XP_014430084.1 : ------------------------------------------------------------------------------------------------------------------------- : -

Xenla_OCT63184.1 : ------------------------------------------------------------------------------------------------------------------------- : -

Xenla_XP_018122800.1 : ------------------------------------------------------------------------------------------------------------------------- : -

Xentr_XP_002943568.3 : ------------------------------------------------------------------------------------------------------------------------- : -

Xentr_XP_012813408.1 : ------------------------------------------------------------------------------------------------------------------------- : -

* 140 * 160 * 180 * 200 * 220 * 240

Homsa_NP_060124.2 : ------------------------MAKQL----------------------------------------------------------------------NLP-ENTDDWTKEDVNQ--WL- : 23

Homsa_NP_689916.2 : ------------------------MSKQV----------------------------------------------------------------------SLP-EMIKDWTKEHVKK--WV- : 23

fish_XP_015241820.1 : ------------------------------------------------------------------------------------------------------------------------- : -

fish_XP_018543893.1 : ------------------------------------------------------------------------------------------------------------------------- : -

fish_CDQ83481.1 : ------------------------------------------------------------------------------------------------------------------------- : -

fish_CDQ89582.1 : ------------------------------------------------------------------------------------------------------------------------- : -

fish_XP_005809248.1 : ------------------------------------------------------------------------------------------------------------------------- : -

fish_XP_007231320.1 : ------------------------------------------------------------------------------------------------------------------------- : -

fish_XP_007254667.1 : ------------------------------------------------------------------------------------------------------------------------- : -

fish_XP_008277954.1 : ------------------------------------------------------------------------------------------------------------------------- : -

fish_XP_010746900.1 : ------------------------------------------------------------------------------------------------------------------------- : -

fish_XP_012685053.1 : ------------------------------------------------------------------------------------------------------------------------- : -

fish_XP_013123332.1 : ------------------------------------------------------------------------------------------------------------------------- : -

fish_XP_014011897.1 : ------------------------------------------------------------------------------------------------------------------------- : -

fish_XP_014011904.1 : ------------------------------------------------------------------------------------------------------------------------- : -

fish_XP_014264868.1 : ------------------------------------------------------------------------------------------------------------------------- : -

fish_XP_014264875.1 : ------------------------------------------------------------------------------------------------------------------------- : -

fish_XP_014854842.1 : ------------------------------------------------------------------------------------------------------------------------- : -

fish_XP_017578465.1 : ------------------------------------------------------------------------------------------------------------------------- : -

fish_XP_012713132.1 : ------------------------------------------------------------------------------------------------------------------------- : -

fish_XP_010787190.1 : ------------------------------------------------------------------------------------------------------------------------- : -

fish_XP_017319342.1 : -----------------MDYSGEKMEELH----------------------------------------------------------------------STP---TEKWTEDMVSV--W-- : 27

Alligator_KYO30992.1 : -----------------------MKGGQI----------------------------------------------------------------------HIL-TRKRNELLEHLHK--NTG : 25

Alligator_XP_006025954.1 : ---MPKR---------KKKNPNTKESKQL----------------------------------------------------------------------DLS-QNFEQWTKEQVSQ--WV- : 35

amphibia_XP_018413082.1 : -------------------MTELKMETPK----------------------------------------------------------------------SMP-SNIEEWTRDHVKL--WV- : 28

bact_AGA70736.1 : ------------------------------------------------------------------------------------------------------------------------- : -

bact_CUJ86040.1 : -------------------------------------------------------------------------------------------------------MQSQVFREKVPRI----- : 13

bact_ESW58583.1 : ------------------------MANTP-------------------------------------------------------------------------------------------- : 5

bact_GAC67715.1 : --------------MSERGCLLLQGELAA----------------------------------------------------------------------GAEIRLVKPLTGGRSGARVWL- : 36

bact_KMO66808.1 : ---------------MSQDGGQTATQDAS----------------------------------------------------------------------DTPTADAMELIRSHFAG----- : 31

bact_KUO79116.1 : ------------------------MSTLE----------------------------------------------------------------------NNVYSFLIEYFRTGNRN----- : 22

bact_KYG06363.1 : -------------------------------------------------------------------------------------------------------MSNQGWPTEVTEL----- : 13

bact_ODA70488.1 : -----------------------MNRDSA----------------------------------------------------------------------KSDGGPGRDVLESGLAL----- : 23

bact_OGP49286.1 : ----------------------MGDLDKK----------------------------------------------------------------------RVTFLHISDFHFKTDHETAFDQ : 29

bact_SCF05533.1 : -----------------------MKEELR----------------------------------------------------------------------LRD-PRHQELAEAALRE--WLP : 25

bact_SCX17622.1 : ------------------------------------------------------------------------------------------------------------------------- : -

bact_SFJ80976.1 : -----------------------MDSELR----------------------------------------------------------------------LGD-PRHQNVAEEALRL--WLP : 25

bact_WP_006128369.1 : RADDLETGSGTTDERCSLGHVGGNVDGDP----------------------------------------------------------------------GLADEAQRNAVVEALKD--WLP : 52

bact_WP_006365791.1 : ------------------------------------------------------------------------------------------------------------MKEFELRQ----- : 8

bact_WP_006849212.1 : --------------------MENIQEQLK----------------------------------------------------------------------EQYNMSIEFYNKGDYEHYFFHT : 31

bact_WP_007897046.1 : ------------------------------------------------------------------------------------------------------------------------- : -

bact_WP_030194308.1 : ------------MENYSAENDTGSGQDLL----------------------------------------------------------------------ESGLALLRNHLPGHASV----- : 34

bact_WP_030198971.1 : --------------------MTRLFEQDG----------------------------------------------------------------------------TGTVDQDEVDRRNRAA : 25

bact_WP_030898614.1 : ------MPEVPAEKNELALTEVLRQAEVG----------------------------------------------------------------------EAVASELEKWQQAHDTR--IVF : 43

bact_WP_041219555.1 : ------------------------MGRST-------------------------------------------------------------------------------------------- : 5

bact_WP_050505446.1 : ------------------MDISGNSGDIQ----------------------------------------------------------------------PGTYVLRVGPEEEDLSE----- : 28

bact_WP_055538636.1 : ------------------MTRLFEQDGTG----------------------------------------------------------------------TVD-EEEVKW-RERVAQ--IV- : 28

bact_WP_062134708.1 : ------------------------------------------------------------------------------------------------------------------------- : -

bact_WP_062799339.1 : ------------------------------------------------------------------------------------------------------------------------- : -

bact_WP_066463897.1 : ------------------------MTNVN----------------------------------------------------------------------ILKMGRIAELNEVKTSY----- : 22

bird_XP_002194798.1 : -------------------------MDYK----------------------------------------------------------------------RLP---VSEWVEDHVKC--W-- : 19

bird_XP_005010984.1 : -----------------MENANTENKEKL----------------------------------------------------------------------HFY-QHAETWTKEEVKQ--FL- : 30

bird_XP_005505023.1 : -----------------MENTNTEKNEKS----------------------------------------------------------------------HSY-QPIEEWTKEEVKH--WA- : 30

bird_XP_008492486.1 : -----------------MENLSTEKHEKS----------------------------------------------------------------------YLC-KPLKQWTKEEVKQ--WA- : 30

bird_XP_008628214.1 : -----------------MEKATTKKNEES----------------------------------------------------------------------YSY-QHIEQWTKEQVKQ--WA- : 30

bird_XP_009068034.1 : ---------------------------YK----------------------------------------------------------------------TLP---VNEWDENHVKC--W-- : 17

bird_XP_009080343.1 : -----------------MEKATTEQNENS----------------------------------------------------------------------WSY-QHIEQWTKEEVKQ--WA- : 30

bird_XP_009486154.1 : -----------------MENANTENNEKS----------------------------------------------------------------------HSD-QYFEQWTKEEVKQ--WA- : 30

bird_XP_009640122.1 : -----------------MENANTEKNEKS----------------------------------------------------------------------QSY-QHFEQWTKEEVKQ--WA- : 30

bird_XP_009672623.1 : -----------------MENTNSEQNAKS----------------------------------------------------------------------QLY-QNIDDWTKEQVRQ--WA- : 30

bird_XP_009695665.1 : RRRLADRQALQGFWSLTAQFPSSKAMDYR----------------------------------------------------------------------TLP---VNKWDENHVQC--W-- : 165

bird_XP_009886214.1 : -----------------MENANTEKNEKS----------------------------------------------------------------------HSH-QHIEQLTKEEVKQ--WV- : 30

bird_XP_009934438.1 : -----------------MENANTGKNEKS----------------------------------------------------------------------HSY-QHIEKWTKEEVKQ--WA- : 30

bird_XP_009999877.1 : -----------------MENANTEINEKS----------------------------------------------------------------------YSY-QDLEQWTKEEVKQ--WA- : 30

bird_XP_010119033.1 : -----------------MENANTEKKEK------------------------------------------------------------------------AF-QHIEQYTKEEVKK--WA- : 28

bird_XP_010144931.1 : -----------------MENENTEKEEKS----------------------------------------------------------------------QSS-RPIKLWTKEEVKK--WA- : 30

bird_XP_010222910.1 : -----------------MENNNTEENEKS----------------------------------------------------------------------KLY-QNVDNWTKEQVRQ--WV- : 30

bird_XP_013160560.1 : -------------MLKSMENSNTEENEKS----------------------------------------------------------------------YSY-QHIEEWTKEEVKQ--WA- : 34

bird_XP_014795808.1 : -----------------MDNANTEKYEKT----------------------------------------------------------------------HSY-QHMEQLTKEEVKQ--WA- : 30

bird_XP_015156587.1 : ----------------------NTEKKRE----------------------------------------------------------------------LHVYQHIEKWTKEEVKQ--FL- : 26

bird_XP_015709021.1 : ----------MQNILKRMEEANSEKKEKL----------------------------------------------------------------------HLY-QHTEKWTKEEVKQ--FL- : 37

brachiop_XP_013415988.1 : QIPHKANAQRKTGLGGGAKGGKKRKGSRN----------------------------------------------------------------------RLPSLSDNDSSASSSST----- : 50

Cioin_XP_018668123.1 : --------------------------------------------------------------------------------------------------MNTPPHHVNCFFINAEST----- : 18

Cnidaria_EDO35430.1 : ----------------MASGFSEEGENFR----------------------------------------------------------------------QQLLEIHNGLGKEELEKLIFLC : 35

Cnidaria_KXJ09690.1 : ---------MDSDKSDLLEKEDKEASQLA----------------------------------------------------------------------AKSGKKKKKKKKKKTST--TIE : 40

Cnidaria_KXJ11435.1 : -----------MDSDKSDCLEKEDKEESQ----------------------------------------------------------------------HAP-KSGKKKKKKKKKK--STT : 37

Cnidaria_KXJ17955.1 : -----------------MSQSDEQENHFE----------------------------------------------------------------------RKLSEIAFALDEDDLDQ-LLFG : 33

Cnidaria_KXJ23466.1 : ------------------MAESRHAKIQS----------------------------------------------------------------------TKP-PNSRRREQFRSKL--SLI : 30

Cnidaria_XP_015751626.1 : --------------MESRGEFRLKLTEVA----------------------------------------------------------------------SELDVEKLNRLKYLCSD--TIP : 35

Cnidaria_XP_015751629.1 : -------------MESRGEFRLKLTEVAS----------------------------------------------------------------------ELD-VEKLNRLKYLCSD--TIP : 35

Cnidaria_XP_015758975.1 : FKDAQEDCDSVLLLNPHHTKARYRLAESLDGQGKIAEAFTQVTSLQRIYPENLAIVQLESSLKRKIEDSKQEAMEKHREGQECFRKSNFRAAIFNFSEGKLLLPPLDMYAEEMKQFCWWLA : 141

Cnidaria_XP_015766106.1 : ----------------------------------------------------------------------------------------------------------------------MCI : 3

Cnidaria_XP_015766118.1 : ----------------------MATKEAA----------------------------------------------------------------------ASAGGRNSERKEENTGDGLSLS : 29

Cnidaria_XP_015766124.1 : -----------------MCHSRFLCQLLS----------------------------------------------------------------------TLP-LRHRELCDNCGRR----- : 28

coelacanth_XP_006000513.2 : -----------------------------------------------------------------------------------------------------------------------M- : 1

fish_CDQ87765.1 : GHGTRDKREGAESPTSSCVSMRSDRSMDPPINYKREFLNYKRQEGADSPTPSYLSMKSDQSMGPSSRMMTSDTTQLSIGGVHATMHTEHHGMNRVSNLREFP-QRIEDWTKEHVKE--WL- : 230

fish_CDQ92295.1 : ------------------MTCSVYKMEWN----------------------------------------------------------------------KLP---CGEWTESHVSS--W-- : 26

fish_KKF13953.1 : -----------------------MADKLD----------------------------------------------------------------------QSP----KSWTESQVST--W-- : 20

fish_XP_004085798.2 : FIYRNMNPTATASIMKDDGLQTKYEKSDL----------------------------------------------------------------------ELP-LRIEDWTKDHVKA--WL- : 53

fish_XP_004552746.3 : -----------------------MNQSTT----------------------------------------------------------------------RKP-PLIEKWTTEDVHQ--WL- : 24

fish_XP_004558390.1 : --------------MCDKTRKEIMADTPQ----------------------------------------------------------------------QSP----KNWTESEVST--W-- : 29

fish_XP_004576250.1 : --MDSNYKKKSTLSSCMSLKSNRSKSPPP------------------------------------DLSSGETMKDRNTVPPGATLTLSGQWEKTADCDLRLP-PLIEDWTREHVKD--WL- : 79

fish_XP_005161888.1 : ------------------------MEKPE----------------------------------------------------------------------DSP---METWTESMVSN--W-- : 20

fish_XP_005478813.2 : -----------------------MNQSTT----------------------------------------------------------------------RKP-PLIEKWTTEDVHQ--WL- : 24

fish_XP_005806405.2 : -----------MAKLSSKMDCNSADLEWH----------------------------------------------------------------------KLP---FSSWTESHVSS--W-- : 33

fish_XP_006625674.1 : ------------------------MSSLS----------------------------------------------------------------------DLP-DRIEDWSREDVHR--WL- : 23

fish_XP_006635547.1 : ------------------------MSPPP----------------------------------------------------------------------PSP--KCLQLMSSAALD--WSG : 23

fish_XP_006635548.1 : -------------------------MEDQ----------------------------------------------------------------------TLP---RDKWDENHVSS--W-- : 19

fish_XP_006791552.1 : -----------------------MAKIPQ----------------------------------------------------------------------QLP--SASGWTESEVST--W-- : 22

fish_XP_006791631.1 : ------------------MESNSSEMDWR----------------------------------------------------------------------ELS---YANWTESHVSS--W-- : 26

fish_XP_007241837.1 : ------------------------MEELH----------------------------------------------------------------------NIP---IENWTETMVGT--W-- : 20

fish_XP_007242299.1 : -------------MEEHETHFDTAMSHKK----------------------------------------------------------------------EFP-LRVEEWTKEQVHQ--WL- : 34

fish_XP_007557414.1 : -----------------------MAEDLE----------------------------------------------------------------------KSP----TNWTESDVSA--W-- : 20

fish_XP_008284284.1 : -----------------MAEQSEMKGAEE----------------------------------------------------------------------DLP-PHIKDWSKHHVRE--WA- : 30

fish_XP_008284762.1 : -------------------------MEWQ----------------------------------------------------------------------ELS---WDRWTESHVSS--W-- : 19

fish_XP_008285761.1 : -----------------------MADRPE----------------------------------------------------------------------QSP----KNWSESDVSN--W-- : 20

fish_XP_008336142.1 : ------------------------MSQST--------------------------------------------------------------------------PNIKKWTTEDVHQ--WL- : 20

fish_XP_008417602.1 : -----------------MDPERKQSSSPSLSMMTDHSKEDRVNFTVDERNYQSSVVDEMKVRPSDPTDPSLKSYRSMSPPPDFSNAIGGLLTKTDDHNLTLP-PIIEDWTKDQVKD--WL- : 100

fish_XP_009510531.1 : -----------------MENTNAEKKEKS----------------------------------------------------------------------HSY-QHIGQWTKEEVKQ--WA- : 30

fish_XP_010752845.1 : ------------------------MKRST----------------------------------------------------------------------TKP-PPFEKWTKEDVHR--WL- : 23

fish_XP_010755274.1 : -----------------MADQGEMKNQEE----------------------------------------------------------------------ELP-PDIKDWSKDQVKE--WA- : 30

fish_XP_010767524.1 : -----------------------MTTRPS------------------------------------------------------------------------P-QLLEKWTEQDVHL--WL- : 22

fish_XP_010781233.1 : KNKGLVIYFCEEETNTAMADRGEIKSQEE----------------------------------------------------------------------DLP-PDIKDWSKHQVKA--WT- : 50

fish_XP_010791966.1 : ------------------------------------------------------------------------------------------------------------------------- : -

fish_XP_012687177.1 : ----------------MTVGKKDFSVKHR----------------------------------------------------------------------DVVEIEARSASEKDMTV----- : 30

fish_XP_012708385.1 : -----------------------MAEQPH----------------------------------------------------------------------QSP----RQWTESEVSD--W-- : 20

fish_XP_012713253.1 : -------------------MAHEGQFKEE----------------------------------------------------------------------DLP-SDINEWSEHHVRE--WV- : 28

fish_XP_012727798.1 : -------------------------MEWH----------------------------------------------------------------------KLP---FSSWTESHVSS--W-- : 19

fish_XP_012990558.1 : ---------------------------------------------------------------------------------------------------MDSAILMRKWTKEEVHN--WL- : 19

fish_XP_013126906.1 : -----------------MADQGEMKFQEE----------------------------------------------------------------------DLS-SDIQDWSKHQVRQ--WV- : 30

fish_XP_013796981.1 : -----------------MENTNTEENEKS----------------------------------------------------------------------KLY-QNVDNWTKEQVRQ--WA- : 30

fish_XP_014005049.1 : -----------------------MKTEPN-------------------------------------------------------------------------------------------- : 6

fish_XP_014049585.1 : -----------------------MAEEPD----------------------------------------------------------------------ELP---VEKWTDSNVSS--W-- : 21

fish_XP_014325953.1 : ---------------------------------------------------------------------------------------------------MSQ-SRVEEWTTADVHH--WL- : 18

fish_XP_015195770.1 : -----MEPPGAAEANLEEVKQSEALSSQP----------------------------------------------------------------------SLP-KAIKEWDKHHVKQ--WI- : 42

fish_XP_015197335.1 : ------------------------MSLSA----------------------------------------------------------------------DLP-EKIEDWTKDHVYK--WL- : 23

fish_XP_015232478.1 : ------------------------------------------------------------------------------------------------------------------------- : -

fish_XP_015252023.1 : -------------------------MKEE----------------------------------------------------------------------DLC-PEMKDWTKHQVKE--WV- : 22

fish_XP_015264177.1 : ------------------------MSDYG----------------------------------------------------------------------QLP---VTEWNESHVKS--W-- : 20

fish_XP_015801149.1 : -----------------------MADKLG----------------------------------------------------------------------QSP----ENWTESEVSF--W-- : 20

fish_XP_015810486.1 : -------------------MADQGDIKGE----------------------------------------------------------------------DLP-CDLKDWSKHDVRF--WV- : 28

fish_XP_016115105.1 : -----------MEASRHSEKKKDQTAVSS----------------------------------------------------------------------ERP-VDINDWNKDHVRQ--W-- : 35

fish_XP_016117315.1 : ------------------------MSQKT----------------------------------------------------------------------TLP-HLVEKWTKEHVHY--WL- : 23

fish_XP_016395637.1 : ------------------------MEKPE----------------------------------------------------------------------DPP---LETWTESMVSN--W-- : 20

fish_XP_017207716.1 : ------------------------MNQKT----------------------------------------------------------------------TLP-CHVKEWTKEQVHY--WL- : 23

fish_XP_017277259.1 : ------------------MDCSSSEMDWH----------------------------------------------------------------------ELS---YNHWTESHVSS--W-- : 26

fish_XP_017277301.1 : -----------------------MADKPE----------------------------------------------------------------------QSP----QSWSESEVSV--W-- : 20

fish_XP_017325460.1 : AVEEVHVNLRSHFQVSILPNNRREISHST----------------------------------------------------------------------ELP-AEIKDWDKEHVRD--WI- : 52

fish_XP_017346953.1 : ------------------------MEDLY----------------------------------------------------------------------STP---VEKWTESMVSN--W-- : 20

fish_XP_017550536.1 : -------------MATSSGSSSQNAAAMS----------------------------------------------------------------------DYKHHPIAKWTKEHVHA--WL- : 35

fish_XP_017550824.1 : -------------MANILEDNGSEVAHST----------------------------------------------------------------------ELP-AEIKDWDKNHVRD--WI- : 34

fish_XP_017554653.1 : ------------------------MSHKI----------------------------------------------------------------------KLP-PKVGEWTKEHVQQ--WL- : 23

fish_XP_017569575.1 : ------------------------MEELS----------------------------------------------------------------------NTP---VEKWTESMVST--W-- : 20

fish_XP_018413083.1 : ------------------------MDKPF----------------------------------------------------------------------DLP-PNITDWTKEHVRY--WV- : 23

fish_XP_018429299.1 : ---MVGVTIGFLVSSLTKALRSRVTSSWK----------------------------------------------------------------------NYKSVPLDDWTEDHVKA--W-- : 44

fish_XP_018521334.1 : ------------------------MSQPT----------------------------------------------------------------------PEPQLSIEKWTTEDVHQ--WL- : 24

fish_XP_018526467.1 : -----------------------MADKFE----------------------------------------------------------------------QSP----ENWTESQVST--W-- : 20

fish_XP_018541041.1 : AKKKKKASSSSRLSMMSDLSREAPPDFSNEEKNPPCAETDRMDADHEKRPTSPVCMSVKSDESMSLRVTFNSGEKEARNVMQVAKQSPAVGSQANTCDIKLP-PMIEDWTKEHVKD--WL- : 119

fish_XP_018558293.1 : --------MYEEGIKAVMTDQGKMMSQEE----------------------------------------------------------------------DLS-PDIKDWSKHQVRE--WA- : 39

fish_XP_018595329.1 : ------------------------MSSMT----------------------------------------------------------------------DLP-ADIEAWTKEDVRQ--WL- : 23

fish_XP_018611696.1 : ---------------------MFDQQEHE----------------------------------------------------------------------NLP---VEKWTESHVSS--W-- : 23

fish_XP_018956725.1 : ---------------------MATASQHT----------------------------------------------------------------------ELP-LRVEEWTREQVYY--WL- : 26

frog_XP_018091031.1 : ------------------------MEEYR----------------------------------------------------------------------KLP---LDDWTEEHVRD--W-- : 20

Hemichordata_XP_006825609.1 : KIQVFLYSDFSGPIRFVGMKTDQVSSIIKFISMTFNLPASKIRLATGTNFQLCEVLSLDDHGIDADTNIYVTIEESGLKGGADSEDAIKKKTDESKGDDDASNDNIHRWRKEDVRH--WL- : 207

lance_EEN48978.1 : ---------------------------------------------------------------------------------------------------------MRHLDKEPTRS----- : 11

lance_EEN55742.1 : ---------------------------------------------------------------------------------------------------MLTWLFNKDYFRTGAVA----- : 17

mammal_XP_001378512.1 : ------------------------MAEQT----------------------------------------------------------------------NLP-ENPDDWTVEDVCQ--WI- : 23

mammal_XP_003771993.1 : ------------------------MDEQL----------------------------------------------------------------------TLP-KNTDDWTIEDVYQ--WV- : 23

mammal_XP_003771994.1 : ------------------------MTDPT----------------------------------------------------------------------P----SIDDWTKEHVKQ--WL- : 20

mammal_XP_003782717.1 : ------------------------MAKQL----------------------------------------------------------------------NLP-ENTDDWTKEDVNQ--WL- : 23

mammal_XP_003982796.1 : ------------------------MSEKV----------------------------------------------------------------------ELP-EVTNNWTKEHVKQ--WV- : 23

mammal_XP_004389713.1 : ------------------------MSEQV----------------------------------------------------------------------TLP-EMTQDWTKEHVKK--WV- : 23

mammal_XP_004582357.1 : -------------------------MAQL----------------------------------------------------------------------KLP-ENTEDWTKEDVNK--WL- : 22

mammal_XP_004582358.1 : ------------------------MNGQT----------------------------------------------------------------------TLP-EMTEEWTKEHVKK--WV- : 23

mammal_XP_004622892.1 : ------------------------MSEHT----------------------------------------------------------------------NLP-EMTKDWTKEDVKQ--WV- : 23

mammal_XP_004646313.1 : ------------------------MNEQV----------------------------------------------------------------------TLP-EMIKNWTKEDVTK--WV- : 23

mammal_XP_004702866.1 : ------------------------MSEQV----------------------------------------------------------------------PLP-EKIQDWTKEHVEK--WV- : 23

mammal_XP_005340592.1 : ------------------------MNEQE----------------------------------------------------------------------TLP-EIIKDWTKEHVKK--WV- : 23

mammal_XP_005388084.1 : ------------------------MNEQV----------------------------------------------------------------------ILP-EKIKDWTKEHVKK--WV- : 23

mammal_XP_006163552.1 : ------------------------MSEQI----------------------------------------------------------------------TLP-KMIKDWTKEHVKK--WV- : 23

mammal_XP_006779273.1 : ------------------------MSEQV----------------------------------------------------------------------NLP-EMTKDWNKEHVKQ--WV- : 23

mammal_XP_006834357.1 : ------------------------MSDQV----------------------------------------------------------------------TIP-ELTRDWTKEHVKK--WV- : 23

mammal_XP_006891533.1 : ------------------------MSEQI----------------------------------------------------------------------ILP-EVTQDWTKEHVKK--WV- : 23

mammal_XP_007522555.1 : ------------------------MDEQV----------------------------------------------------------------------NLP-EMTQDWTKEHVKQ--WV- : 23

mammal_XP_007938002.1 : ------------------------MSQQE----------------------------------------------------------------------TLP-EMTQDWTKEHVKK--WV- : 23

mammal_XP_008062458.1 : ------------------------MAKQL----------------------------------------------------------------------DFA-ENTDDWTKENVNQ--WL- : 23

mammal_XP_008259950.1 : ------------------------MNEQV----------------------------------------------------------------------TLP-ELVKDWTKEHVKK--WV- : 23

mammal_XP_008524371.1 : ------------------------MNEQA----------------------------------------------------------------------NLP-EMTKDWTKEHVKQ--WV- : 23

mammal_XP_008820771.1 : ------------------------MNEQV----------------------------------------------------------------------TLP-KKTKDWTKEHVKK--WI- : 23

mammal_XP_010601767.1 : ------------------------MHEQV----------------------------------------------------------------------TLP-KMIKDWTKEHVKK--WV- : 23

mammal_XP_011853657.1 : ------------------------MSKQV----------------------------------------------------------------------SLP-EIIKDWTKEHVKK--WV- : 23

mammal_XP_012791283.1 : ------------------------MAAQP----------------------------------------------------------------------NLS-KNPDDWTKEDVNI--WL- : 23

mammal_XP_012881542.1 : ------------------------MNEQV----------------------------------------------------------------------NLP-ERIKDWTKEQVKK--WV- : 23

mammal_XP_012934202.1 : ------------------MEEGITMAEQL----------------------------------------------------------------------NLP-ENTDEWTKEDVNR--WL- : 29

mammal_XP_013013639.1 : ------------------------MNEQG----------------------------------------------------------------------TLP-EMIKDWTKDHVKK--WI- : 23

mammal_XP_016016798.1 : ------------------------MSEQV----------------------------------------------------------------------NLP-KMTNDWTKEHVKR--WV- : 23

mammal_XP_016281899.1 : -----------------------MADPIP---------------------------------------------------------------------------KVDDWTKEYVKQ--WV- : 20

mammal_XP_017199853.1 : ------------------------MAEQP----------------------------------------------------------------------ELP-ENTDDWTKEDVNR--W-- : 22

mammal_XP_017508280.1 : ---------------MKIRKVPALMSEQV----------------------------------------------------------------------NLP-EMTKDWTKEHVKQ--WV- : 32

mollusk_EKC42356.1 : ---------------------------------------------------------------------------------------------------MDP---SRNWAQDVLRC----- : 14

mollusk_XP_011436570.1 : QTNFSCLLENLLVGEIADNLFSQCVIEHD----------------------------------------------------------------------DLQRVHAEVTDKDKARC--LIN : 58

mollusk_XP_011440496.1 : --------MASKGSKSKGKSRSKKRQPAP----------------------------------------------------------------------STFNINIEKFIDIQQSS--GIV : 41

mollusk_XP_013061423.1 : RNMEDLLGINPVYPIIIHLYENDVIELSE----------------------------------------------------------------------KVAIEKIKVYDRQMKKLLKILK : 61

mollusk_XP_013061425.1 : ----------------MIFVIISFISGES----------------------------------------------------------------------VET-KEVKDMSSSELTA--YLA : 32

Musmu_NP_034286.2 : ------------------------MSGQV----------------------------------------------------------------------TQP-KLIKDWTKEHVRK--WV- : 23

rept_XP_008110733.2 : MMYSLKGNGDPG----EGTSKSYKQEHSL----------------------------------------------------------------------ALN-DKIENWTKEHVKQ--WV- : 43

rept_XP_008112638.1 : -------------------------MDYT----------------------------------------------------------------------DLP---LDEWNESHVKY--W-- : 19

rept_XP_015264172.1 : FFFSSAKFNNFGFFFLSAKNKSIKKNDQQ----------------------------------------------------------------------NLP-EKIEDWTKEHVKQ--WV- : 53

shark_XP_007886940.1 : ------------------------MPNPP-------------------------------------------------------------------------------------------- : 5

Strpu_XP_003727489.1 : ---------------------------------------------------------------------------------------------------------------MEVDA--Y-- : 6

Strpu_XP_011667260.1 : --------------------------------------------------------------------------------------------------------EVAIWLKSNVHL--SD- : 14

turtle_XP_006139744.1 : ---MGKK---------RAKVKNLEKLERP----------------------------------------------------------------------NLS-SNIDEWTKEQVKQ--WV- : 35

turtle_XP_007060369.1 : ---MGKK---------RAKVKNLEKSEQP----------------------------------------------------------------------NLP-PNIDDWTKEQVKQ--WV- : 35

turtle_XP_014430084.1 : -------------------------MDYK----------------------------------------------------------------------TLP---VAMWNEYHVSA--W-- : 19

Xenla_OCT63184.1 : -------------------------MDYR----------------------------------------------------------------------SIP---VKEWTEGDVGE--W-- : 19

Xenla_XP_018122800.1 : ------------------------MDKPL----------------------------------------------------------------------DLP-LNVDTWTKDHVKK--WV- : 23

Xentr_XP_002943568.3 : ----MKEGQIQLLISKRDEILLGQKSTEP----------------------------------------------------------------------GSPSQYTQASSSHQEPQALADP : 47

Xentr_XP_012813408.1 : -------------------------MDSQ----------------------------------------------------------------------LLP---LDDWTESHVRD--W-- : 19

* 260 * 280 * 300 * 320 * 340 * 360

Homsa_NP_060124.2 : ----ESHKIDQKH---------------------------------------------REILTEQDVNG------AVLKWLKKEHLVDM-------------------------------- : 57

Homsa_NP_689916.2 : ---NEDLKINEQY---------------------------------------------GQILLSEEVTG------LVLQELTEKDLVEM-------------------------------- : 58

fish_XP_015241820.1 : ------------------------------------------------------------------------------------------------------------------------- : -

fish_XP_018543893.1 : ------------------------------------------------------------------------------------------------------------------------- : -

fish_CDQ83481.1 : ------------------------------------------------------------------------------------------------------------------------- : -

fish_CDQ89582.1 : ------------------------------------------------------------------------------------------------------------------------- : -

fish_XP_005809248.1 : ------------------------------------------------------------------------------------------------------------------------- : -

fish_XP_007231320.1 : ------------------------------------------------------------------------------------------------------------------------- : -

fish_XP_007254667.1 : ------------------------------------------------------------------------------------------------------------------------- : -

fish_XP_008277954.1 : ------------------------------------------------------------------------------------------------------------------------- : -

fish_XP_010746900.1 : ------------------------------------------------------------------------------------------------------------------------- : -

fish_XP_012685053.1 : ------------------------------------------------------------------------------------------------------------------------- : -

fish_XP_013123332.1 : ------------------------------------------------------------------------------------------------------------------------- : -

fish_XP_014011897.1 : ------------------------------------------------------------------------------------------------------------------------- : -

fish_XP_014011904.1 : ------------------------------------------------------------------------------------------------------------------------- : -

fish_XP_014264868.1 : ------------------------------------------------------------------------------------------------------------------------- : -

fish_XP_014264875.1 : ------------------------------------------------------------------------------------------------------------------------- : -

fish_XP_014854842.1 : ------------------------------------------------------------------------------------------------------------------------- : -

fish_XP_017578465.1 : ------------------------------------------------------------------------------------------------------------------------- : -

fish_XP_012713132.1 : ------------------------------------------------------------------------------------------------------------------------- : -

fish_XP_010787190.1 : ------------------------------------------------------------------------------------------------------------------------- : -

fish_XP_017319342.1 : ---LASIGVKENY---------------------------------------------IKTLHEQEVDG------QVLLKITEEFLKQET------------------------------- : 63

Alligator_KYO30992.1 : LDLALANDSSKKA---------------------------------------------SEALVCESTNN------AGKKKGKSRNSA---------------------------------- : 61

Alligator_XP_006025954.1 : ---VEDLKIDPKH---------------------------------------------GEILLNQDVTG------CTLRALTKQALIDM-------------------------------- : 70

amphibia_XP_018413082.1 : ---TKQLKLSLSV---------------------------------------------GDILYNQEVTG------EVLKVLTKKDLVEM-------------------------------- : 63

bact_AGA70736.1 : ---MNSNYMDKNK---------------------------------------------IVFYYNQSVE----------------------------------------------------- : 20

bact_CUJ86040.1 : ---VAKVRHGEVY------------------------------------------------VLLLEIGG--------SSRIKWKPLRNTL------------------------------- : 44

bact_ESW58583.1 : ------------------------------------------------------------------------------------------------------------------------- : -

bact_GAC67715.1 : ---CDIIEPASGS---------------------------------------------------ASVDG------QYVLKVSSGPLIT--------------------------------- : 64

bact_KMO66808.1 : ----------------------------------------------------------ATVELIRPLSGGKSGAQVLLIDLTDATQR---------------------------------- : 60

bact_KUO79116.1 : ----------------------------------------------------------AIEIVSQNSDG------KSGALVYSLHVKHATPKN---------------------------- : 51

bact_KYG06363.1 : ---ISKFSQETGA---------------------------------------------AITIREPLLQG------KSGAFVAIVDCKGQH------------------------------- : 49

bact_ODA70488.1 : ---LRSHLPRAS----------------------------------------------SVEIVKPLAGG------YTDARVMLCDIAQA-------------------------------- : 57

bact_OGP49286.1 : NIVLKSLIQE------------------------------------------------IKKICDDKEWR------PELVLCTGDMAFSG-------------------------------- : 64

bact_SCF05533.1 : AAKILFAPMAPGRS--------------------------------------------GVELVRIDVSG------ETADRAPGSYVL---------------------------------- : 62

bact_SCX17622.1 : ---------------------------------------------------------------------------MGLVQINFSGLLAS-------------------------------- : 14

bact_SFJ80976.1 : SAAFLFEPMSPGRS--------------------------------------------GAELTGVDISG------QTGDIQPGTYVL---------------------------------- : 62

bact_WP_006128369.1 : DANLIYRAMPQGRS--------------------------------------------EASLLGVDVSG------RTGTTFKPGFYVV--------------------------------- : 90

bact_WP_006365791.1 : ------------------------------------------------------------------------------------------------------------------------- : -

bact_WP_006849212.1 : RKSIELIGKFLIYD--------------------------------------------TMKIKGEEEMA------SKIIAGDSSFDLDF-------------------------------- : 70

bact_WP_007897046.1 : ------------------------------------------------------------------------------------------------------------------------- : -

bact_WP_030194308.1 : ---EIVKALAGGYTDARVML--------------------------------------CDIGNKPGRTG--------DPVLNGQYILKA-------------------------------- : 74

bact_WP_030198971.1 : EIVERELGAPVKI-----------------------------------------------TYLDDFADG------FTGARVTRCDLM---------------------------------- : 59

bact_WP_030898614.1 : DRWLTPGKSRAVLA--------------------------------------------AVALTGPVFNG------KVVMKVCPPGTTTGR------------------------------- : 83

bact_WP_041219555.1 : ---LNSNYMDKNK---------------------------------------------IVFYYNQSVE----------------------------------------------------- : 25

bact_WP_050505446.1 : ----------------------------------------------------------ANELAAHQLIR------DADPAFAERHIPRLLRIDQR-------------------------- : 59

bact_WP_055538636.1 : ---ERELGMPVKI-----------------------------------------------TYLDGFADG------FTAARVTRCDLM---------------------------------- : 59

bact_WP_062134708.1 : ----------------------------------------------------------------MSTSG---------------------------------------------------- : 5

bact_WP_062799339.1 : ---MEIDGVD------------------------------------------------VLLLCAVQVEW------ECLKSLLVDATPDAR------------------------------- : 33

bact_WP_066463897.1 : ---KDSKNIIDCY--------------------------------------------------------------NQSILFYKDHLFYLDSHS---------------------------- : 50

bird_XP_002194798.1 : ---LESTGIKKEY---------------------------------------------VDKLHEEEVTG------PALMELDESFLKD-I------------------------------- : 54

bird_XP_005010984.1 : ---TEILKIDQKY---------------------------------------------AEILYDQDVTG------STLKLLTKADLVEM-------------------------------- : 65

bird_XP_005505023.1 : ---TEVVKIDQKY---------------------------------------------AEILFNQEVTG------FTLKLMTKADFVDM-------------------------------- : 65

bird_XP_008492486.1 : ---IEVVKIDHQY---------------------------------------------AEILFNQKVTG------CSLKEITKADLVAM-------------------------------- : 65

bird_XP_008628214.1 : ---TEVAKIDQEH---------------------------------------------AEILFRQAVTG------FALKRMTKADLVEM-------------------------------- : 65

bird_XP_009068034.1 : ---LEATGIKKEY---------------------------------------------VEKLCAEEVTG------PALMALDEPFLKR-M------------------------------- : 52

bird_XP_009080343.1 : ---IEVVKIDQEY---------------------------------------------AEILFNQAVTG------FTLKQMTKTDFLKM-------------------------------- : 65

bird_XP_009486154.1 : ---TEVVKIDQKH---------------------------------------------AEILFNQDVTG------FTLKLMTKTDLVEM-------------------------------- : 65

bird_XP_009640122.1 : ---TEVVKIDQKY---------------------------------------------AEILFNEDVTG------FTLKLITKTDFVEM-------------------------------- : 65

bird_XP_009672623.1 : ---IEELKIDEKY---------------------------------------------AEILFNQDVTG------SILRLLNKRDLVDM-------------------------------- : 65

bird_XP_009695665.1 : ---LESTGIKKEY---------------------------------------------VEKLYAEEVTG------PALMELDESFLKG-I------------------------------- : 200

bird_XP_009886214.1 : ---TEVAKIDQKY---------------------------------------------AEILFNQDVTG------FALKLMTKADFVQM-------------------------------- : 65

bird_XP_009934438.1 : ---NEVVKIDQKY---------------------------------------------AEILFDQDVTG------FTLKLMTKADIVEM-------------------------------- : 65

bird_XP_009999877.1 : ---TEVVKIDQKY---------------------------------------------AEILFKEDVRG------VSLKLVTKNDLLEM-------------------------------- : 65

bird_XP_010119033.1 : ---TEVVKIDQKY---------------------------------------------AEILFNEDVTG------FTLKLMTKNDFLEM-------------------------------- : 63

bird_XP_010144931.1 : ---TDVVKIDRKH---------------------------------------------AETLFNQEVTG------FTLKLMSKADLVEM-------------------------------- : 65

bird_XP_010222910.1 : ---VEEVKIDKKH---------------------------------------------AETLFNQDVTG------AVLRLLTKSDLVEM-------------------------------- : 65

bird_XP_013160560.1 : ---IEVVKIDQKY---------------------------------------------AEVLFNQDVTG------PTLKLVSKADFVEM-------------------------------- : 69

bird_XP_014795808.1 : ---TEVVKIDQKY---------------------------------------------AEILFNQDVTG------STLKLMNKADFVQM-------------------------------- : 65

bird_XP_015156587.1 : ---IEDVNIDQKY---------------------------------------------AEILYSQDVTG------STLKLLTKADLVDM-------------------------------- : 61

bird_XP_015709021.1 : ---IEDVNIDQKY---------------------------------------------AEILYSQDVTG------SSLKLLTKADLVDM-------------------------------- : 72

brachiop_XP_013415988.1 : ---ATIVACSKQS---------------------------------------------ERQPKPRARSGMRVELERLLQKYSKNIIRNANNEQLCKALLEKGVIDEKDVKEV--------- : 114

Cioin_XP_018668123.1 : ---ISSSGLEDA----------------------------------------------DISFFSTNIEN----------DFSQKILQ---------------------------------- : 46

Cnidaria_EDO35430.1 : ENCTPGLSSLNITSGLGL----------------------------------------FRELQNCNKIG------PGNVQFLKWRLYAIK------------------------------- : 79

Cnidaria_KXJ09690.1 : EGHSTSNEVSELCAGSIQP---------------------------------------ASQITPEEISE------SNVLHSEEINFLSPS------------------------------- : 85

Cnidaria_KXJ11435.1 : SFEESTLGNSESDP--------------------------------------------ASQRCTENVLS------CCQITPEEKSELNVSHSDENSSLLPSKS------------------ : 90

Cnidaria_KXJ17955.1 : CKDIIPDGIRDAIQN-------------------------------------------VKQLFEELKKR------GKLNEAKQEFLIDLLRSIGHEELACDLESSVDFPLDERTLEQIYNL : 105

Cnidaria_KXJ23466.1 : ACQITTEELDK-----------------------------------------------LKFLSEDIPDG------EREKIKTAEMFFEKL------------------------------- : 67

Cnidaria_XP_015751626.1 : AGDLEKIKTPEQL---------------------------------------------ILELEQRKKIE------PNRLKFLIDCLDGVGRKDLAADLKSYERKRTE-------------- : 91

Cnidaria_XP_015751629.1 : AGDLEKIKTPEQL---------------------------------------------ILELEQRKKIE------PNRLKFLIDCLDGVGRKDLAADLKSYERKRTE-------------- : 91

Cnidaria_XP_015758975.1 : ECHLRNVQAGENNLELALRQCEEADRLVPEDVNILHTKMKVLERMNRHKECYAVCNKLIQSHFKESEAK------KTLEKLLPKIFMEDEVLATEKHVKLELPRSPDFALAKKSKKGRATK : 256

Cnidaria_XP_015766106.1 : FKLIYGVGLTRLR--------------------------------------------------------------KLFMEINPSW------------------------------------ : 26

Cnidaria_XP_015766118.1 : GLAFVTQQPETS----------------------------------------------SAQGGEEQVQP------GGMVKTSKSAAKRK-------------------------------- : 66

Cnidaria_XP_015766124.1 : ---SEGILISASC---------------------------------------------DSSASLRIVHG------DLRKDASESQVHDQLTIP---------------------------- : 67

coelacanth_XP_006000513.2 : ---IEELNLDPVY---------------------------------------------ANILQEEEVSG------ARLIRFEKHNLLQI-------------------------------- : 36

fish_CDQ87765.1 : ---ISSLKLPEV----------------------------------------------ATKLYEQDVSG------ASLVCIEKQDLTDF-------------------------------- : 264

fish_CDQ92295.1 : ---LESIGVKEKY---------------------------------------------ILKLYEEEVTG------PALIELQEKYLRDTI------------------------------- : 62

fish_KKF13953.1 : ---LRSIGVKEQY---------------------------------------------IEKLYEEEVDG------QILLALDENYLKTKI------------------------------- : 56

fish_XP_004085798.2 : ---VCKLRVSEKV---------------------------------------------AQNLYDQELSG------ACLTSFEKDDLLDL-------------------------------- : 88

fish_XP_004552746.3 : ---TTEVKVQQSC---------------------------------------------ADRFTEEEVSG------ACLVSFNKTDILDF-------------------------------- : 59

fish_XP_004558390.1 : ---LRSIGVKEQY---------------------------------------------IEKLHEEEVNG------QILLTLNEEFLKTKI------------------------------- : 65

fish_XP_004576250.1 : ---VITLRVPNHV---------------------------------------------AQSLYEQEFSG------ACLASFEKQDLLDL-------------------------------- : 114

fish_XP_005161888.1 : ---LKSIGIKEAY---------------------------------------------IEKLHEEEVNG------QVLSKVSEEFLKKET------------------------------- : 56

fish_XP_005478813.2 : ---MAEVKVQQSC---------------------------------------------ADRFTEEEVSG------ACLVSFNKTDILDF-------------------------------- : 59

fish_XP_005806405.2 : ---LKSIGIKENY---------------------------------------------ILKLNEEEVTG------SVLTTLQRDYLSKTI------------------------------- : 69

fish_XP_006625674.1 : ---VGVLNVDSRW---------------------------------------------ADKFYEEEVSG------LELVYFQKQDLLDL-------------------------------- : 58

fish_XP_006635547.1 : QTRLLVLALQQDWTGPA-----------------------ERSHVRLHGAVPGDPGGHLRVLWTASKAG------Q---GVTEAWPETAVEMSRFLLGGLPGKSAAFVLIGSPSGSPVETS : 112

fish_XP_006635548.1 : ---LKSIGVKDEY---------------------------------------------IKKLHEAEVTG------PVLKGLTRDFLKNET------------------------------- : 55

fish_XP_006791552.1 : ---LRSIGIKEHY---------------------------------------------IEKLYEEEVDG------QILLALDEDFLKTKI------------------------------- : 58

fish_XP_006791631.1 : ---LRSIGIKEVH---------------------------------------------IIKMEEEEVTG------PILTTLHQDFLRKTI------------------------------- : 62

fish_XP_007241837.1 : ---LQSIGVKEQY---------------------------------------------IKKLHEEEVDG------RILLQITEDYLRKET------------------------------- : 56

fish_XP_007242299.1 : ---SEELKVNQTW---------------------------------------------ADRLYEEDVSG------KELICYKAKDLQEL-------------------------------- : 69

fish_XP_007557414.1 : ---LRSIGVKEQY---------------------------------------------IKKLHEEEVDG------QTLLALTEDFLKTEI------------------------------- : 56

fish_XP_008284284.1 : ---RKLNGVGETV---------------------------------------------AGKLFQQDICG------PSLLLLNRTDLSEI-------------------------------- : 65

fish_XP_008284762.1 : ---LRFIGTEEAH---------------------------------------------IKTLEEEKVTG------PDLATLHREFFSNTV------------------------------- : 55

fish_XP_008285761.1 : ---LRSIGVKQQY---------------------------------------------VEKLYEEEVDG------QILLTLNEDFLKSKI------------------------------- : 56

fish_XP_008336142.1 : ---IGEVKVQLSC---------------------------------------------ADRFSEEDVSG------DCLYSFEKEDILDL-------------------------------- : 55

fish_XP_008417602.1 : ---VIKLRVSKEL---------------------------------------------AQKLYDQELTG------ACLTSYEKEDLLEL-------------------------------- : 135

fish_XP_009510531.1 : ---TEVIKIDQKY---------------------------------------------AEILFKQAVTG------SSLKLATKADFVDM-------------------------------- : 65

fish_XP_010752845.1 : ---VTEVKVHQTC---------------------------------------------ADKFSEEEVLG------DYLVAFEKRDILDL-------------------------------- : 58

fish_XP_010755274.1 : ---LKLDGVDDHI---------------------------------------------AEILFQQDING------PSLLLLEIEDFRGM-------------------------------- : 65

fish_XP_010767524.1 : ---MTEVKVHETL---------------------------------------------ADKFFEEEVSG------SSLDVFGKTDILDL-------------------------------- : 57

fish_XP_010781233.1 : ---LTLDDVDDGV---------------------------------------------AEILFEQDING------PSLLLLDREDLKQM-------------------------------- : 85

fish_XP_010791966.1 : ------------------------------------------------------------------------------------------------------------------------- : -

fish_XP_012687177.1 : ----------------------------------------------------------SDSWTENEKSS------ESPAHVNLHKSSDA-------------------------------- : 55

fish_XP_012708385.1 : ---LRSIGVKETY---------------------------------------------IQKLYEEEVDG------QCLLALDEHFLVTKV------------------------------- : 56

fish_XP_012713253.1 : ---RRLKKVDASV---------------------------------------------ADVLFKQDISG------PSLLLLDMQDLINM-------------------------------- : 63

fish_XP_012727798.1 : ---LESIGIKEKY---------------------------------------------ILKLKEEEVTG------KVLTSLHRDYLSKTI------------------------------- : 55

fish_XP_012990558.1 : ---TTQVNINQIY---------------------------------------------ADELLEEEVSG------EDLDYFQKKDLLDL-------------------------------- : 54

fish_XP_013126906.1 : ---LQLDRVDDKV---------------------------------------------AEILFKEDING------ESLLLLDTTDLTKI-------------------------------- : 65

fish_XP_013796981.1 : ---IEEVKIDKEY---------------------------------------------AEILFNQDVTG------STLRLLTKKDLVDM-------------------------------- : 65

fish_XP_014005049.1 : ---TEEPRIVE-------------------------------------------------------------------------------------------------------------- : 14

fish_XP_014049585.1 : ---LRTIGVKEQY---------------------------------------------IKKLYEEEVDG------SILLVLTEDYLRKEI------------------------------- : 57

fish_XP_014325953.1 : ---RTEVKVSTAC---------------------------------------------ADIFIQEEVSG------EYLVSFRKLDILDL-------------------------------- : 53

fish_XP_015195770.1 : ---LQEPDLDDSC---------------------------------------------AQILYDQDING------PSLLLLDRSDLLNM-------------------------------- : 77

fish_XP_015197335.1 : ---ISSLGVSKVY---------------------------------------------ADQLYHEDVSG------SDLVFFEIEDLTKL-------------------------------- : 58

fish_XP_015232478.1 : ------------------------------------------------------------------------------------------------------------------------- : -

fish_XP_015252023.1 : ---LQLENVEESA---------------------------------------------ADILLQQDING------ASLLLLDTQDLTKM-------------------------------- : 57

fish_XP_015264177.1 : ---LTSIGIKEVY---------------------------------------------AEKLYEEEVTG------PVLKVLDEPFLKRI-------------------------------- : 55

fish_XP_015801149.1 : ---LQSIGVKEQY---------------------------------------------IKKLYEEEVDG------QTLLTLTVDFLKTEI------------------------------- : 56

fish_XP_015810486.1 : ---LTLGGVDGSV---------------------------------------------ADKLFQEDING------PSLKLLTKSDLSEM-------------------------------- : 63

fish_XP_016115105.1 : ---ILEIKVDEED---------------------------------------------AEIFYNQRING------ASLLLLEETDLSSIP------------------------------- : 71

fish_XP_016117315.1 : ---TEVVKVDKKY---------------------------------------------ADKLYDEEVSG------EALVCYQPSDLHYL-------------------------------- : 58

fish_XP_016395637.1 : ---LTSIGVKETY---------------------------------------------IKKLNEEEVDG------RILCELSEEYLEKKI------------------------------- : 56

fish_XP_017207716.1 : ---TEVVRSDKKY---------------------------------------------VEKLYEEDVDG------KALICYKSDDLHYL-------------------------------- : 58

fish_XP_017277259.1 : ---LKSIGIKEPY---------------------------------------------IIRVKEEEVTG------SVLTTLQREYLSNTI------------------------------- : 62

fish_XP_017277301.1 : ---LKSIGVKEQY---------------------------------------------IKKLYEEEVDG------QTLLALNEDFLKTEI------------------------------- : 56

fish_XP_017325460.1 : ---LTLTDVDNEN---------------------------------------------AKILYEQDING------PSLLLLETSDIKDI-------------------------------- : 87

fish_XP_017346953.1 : ---LTSIGVKENY---------------------------------------------IKKLNEEEVDG------QILLELTEEFLKKET------------------------------- : 56

fish_XP_017550536.1 : ---CKTVQVKQNY---------------------------------------------ADMFVKQEISG------ECLLCFEKQDIVDM-------------------------------- : 70

fish_XP_017550824.1 : ---LSFSDVDDEY---------------------------------------------ANILFDQEITG------SSLLLLEKAELIDL-------------------------------- : 69

fish_XP_017554653.1 : ---VEELKIDQTW---------------------------------------------ADKLYEEEVSG------KELICFKTRDLREL-------------------------------- : 58

fish_XP_017569575.1 : ---LRSIGVKEQY---------------------------------------------VKKLHEEEVDG------RILLEITEDFLKKET------------------------------- : 56

fish_XP_018413083.1 : ---TDCLQIEQDE---------------------------------------------ADILYTQKVNG------KALNVLSKEDFQNM-------------------------------- : 58

fish_XP_018429299.1 : ---LHSIRLKPEY---------------------------------------------VDKLYAEEVTX------PVLKEITDNFLKSI-------------------------------- : 79

fish_XP_018521334.1 : ---MTEVKVHQSC---------------------------------------------ADRFIEEEVLG------EYLVLFKKKDILDL-------------------------------- : 59

fish_XP_018526467.1 : ---LRSIGVKEQY---------------------------------------------IKKLFEEEVDG------QILLTLDEDFLKAKI------------------------------- : 56

fish_XP_018541041.1 : ---IIRLRVPQQI---------------------------------------------AQNLYDQELSG------SCLVSYEKQDLLEL-------------------------------- : 154

fish_XP_018558293.1 : ---LKVDGVDNIV---------------------------------------------AEILFKQDISG------HALLLLDTTDLRKM-------------------------------- : 74

fish_XP_018595329.1 : ---LDVLKVPKEH---------------------------------------------ADRLYDEDTSG------ASLVCFEKQDLLDI-------------------------------- : 58

fish_XP_018611696.1 : ---LKSIGVKEHY---------------------------------------------IQKLHEEEVNG------QILLELTEDYLRKET------------------------------- : 59

fish_XP_018956725.1 : ---TEVIKVDKKH---------------------------------------------ADKLYEEEVSG------EELVCYQPKHLQEL-------------------------------- : 61

frog_XP_018091031.1 : ---LCSIRLKKEY---------------------------------------------TDKLFEEEVTG------PALREIKHEFLKT-I------------------------------- : 55

Hemichordata_XP_006825609.1 : ---INTVRMQSKY---------------------------------------------ANMFYEEEVDG------ETLITYKEKDLLEDFKIKDGPLTKLLGYRDNLQIKVPPITFQDLPA : 274

lance_EEN48978.1 : ----------------------------------------------------------ILLTNLPRVAG------SCQVSQLNAEICAALTEA---------------------------- : 40

lance_EEN55742.1 : ---IFSFSVSEKH-----------------------------------------------RVPGAKCES--------DLMEQEGSVEETA------------------------------- : 49

mammal_XP_001378512.1 : ---TEKLKINSKY---------------------------------------------TEILKKEEVNG------KSLKVSDKNDLVDM-------------------------------- : 58

mammal_XP_003771993.1 : ---TGKLKLNPEY---------------------------------------------GEILKKEEVNG------MSLKRSAKQDLIDM-------------------------------- : 58

mammal_XP_003771994.1 : ---TEELKIDEKY---------------------------------------------VQILFSQAVTG------LVLNILTKNDLLEM-------------------------------- : 55

mammal_XP_003782717.1 : ----ESHKVHQKH---------------------------------------------REILTSQDVDG------RILKWLKKKDLIDM-------------------------------- : 57

mammal_XP_003982796.1 : ---TKDLKIDEKY---------------------------------------------GQILIDEEVTG------LVLQELTEMDLREM-------------------------------- : 58

mammal_XP_004389713.1 : ---TEDLKIDEKY---------------------------------------------GQILLREEVTG------LVLQVLTEQDLRDM-------------------------------- : 58

mammal_XP_004582357.1 : ----ESHKIDKKH---------------------------------------------RDTLMAQDVNG------AVLRFLKKTHLVDM-------------------------------- : 56

mammal_XP_004582358.1 : ---TEDLKIDEKY---------------------------------------------GEILFKEEVTG------LVLEQLTEEDLREM-------------------------------- : 58

mammal_XP_004622892.1 : ---TEVLQIDEEY---------------------------------------------GQILLNEKVSG------LALQEITEEDLREM-------------------------------- : 58

mammal_XP_004646313.1 : ---TEELKIDKKY---------------------------------------------GQILFNAEVTG------LVLEELTEEDLEKM-------------------------------- : 58

mammal_XP_004702866.1 : ---TEALKIDDKY---------------------------------------------GHILLREEVTG------LVLQELTEKDLRDM-------------------------------- : 58

mammal_XP_005340592.1 : ---TEDLKIDEKY---------------------------------------------GQILLNEEVSG------LVLQELTEKDLKEM-------------------------------- : 58

mammal_XP_005388084.1 : ---TEELKIDEKY---------------------------------------------GQILLNEEVTG------LVLQELTEKDLKEM-------------------------------- : 58

mammal_XP_006163552.1 : ---TEDLKVDEKY---------------------------------------------GQILYREEVSG------LVLQELTEKDLIDM-------------------------------- : 58

mammal_XP_006779273.1 : ---TEDLKIDEKY---------------------------------------------GQILFNEEVTG------LVLQELTEKDLRDM-------------------------------- : 58

mammal_XP_006834357.1 : ---TEDLKVDEKY---------------------------------------------GQILLNEEVTG------LVLQELTEKDLRDM-------------------------------- : 58

mammal_XP_006891533.1 : ---TEDLKLDEKY---------------------------------------------GQILLREDVTG------LVLQELTEEDLRDM-------------------------------- : 58

mammal_XP_007522555.1 : ---TNDLQIDEKY---------------------------------------------GQILLCEEVTG------RVLQVLTEKDLIEM-------------------------------- : 58

mammal_XP_007938002.1 : ---TEDLKIDEKY---------------------------------------------GQILLSEEVTG------LVLKELTEKDLRDM-------------------------------- : 58

mammal_XP_008062458.1 : ----ESHKFNXKH---------------------------------------------REILTAQDMDG------ETLKXLKKNYLVDT-------------------------------- : 57

mammal_XP_008259950.1 : ---TEDLKVDEKY---------------------------------------------GQILLNEEVTG------LVLKELTEDDLKEM-------------------------------- : 58

mammal_XP_008524371.1 : ---TKDLKIGEKY---------------------------------------------GQILLNEEVTG------LVLQELTEKDLIEM-------------------------------- : 58

mammal_XP_008820771.1 : ---TEDLKIDEKY---------------------------------------------GQILLSEEVTG------PVLQELTKEDLRDM-------------------------------- : 58

mammal_XP_010601767.1 : ---TEELKIDEKY---------------------------------------------GQILLNEEVTG------LVLQELTKKDLKEM-------------------------------- : 58

mammal_XP_011853657.1 : ---TDDLKINEQY---------------------------------------------GQILLSEEVTG------LVLQELTEKDLIEM-------------------------------- : 58

mammal_XP_012791283.1 : ----EEHNIDKKY---------------------------------------------REILVAQDVNG------DVLKLLTKKHLVDM-------------------------------- : 57

mammal_XP_012881542.1 : ---TEDLKIDEKY---------------------------------------------GQILFNEEVTG------LVLQELTEKDLKDM-------------------------------- : 58

mammal_XP_012934202.1 : ----ESHKIDQKH---------------------------------------------REILMAQDVSG------AILKWLNKSHLVDM-------------------------------- : 63

mammal_XP_013013639.1 : ---TEELKIDEKY---------------------------------------------GQILFNEEVTG------LVLQELTEKDLKEM-------------------------------- : 58

mammal_XP_016016798.1 : ---TEDLQIDEKY---------------------------------------------GQILLKEEVTG------LVLQELTEKDLRDM-------------------------------- : 58

mammal_XP_016281899.1 : ---TEELRIDERY---------------------------------------------GEILLSQEVTG------LVLNILTEKDLIDM-------------------------------- : 55

mammal_XP_017199853.1 : ---LESHKIHQKH---------------------------------------------RDILVAQDVSG------AVLKFLKKSDLIEM-------------------------------- : 57

mammal_XP_017508280.1 : ---TKDLKIDEKY---------------------------------------------GQILLNEEVTG------LVLQELTEKDLIEM-------------------------------- : 67

mollusk_EKC42356.1 : --HLCETPVPPMYCDIC-----------------------------------------HIHLCKACVGE------HLSDELKEHKVVSFK------------------------------- : 55

mollusk_XP_011436570.1 : ILYTKENSYDPFIRELRSIHTYGYL---------------------------------ADQLNDTDVEKELEQEKENWMKYLSDALEERYRFVKEKSILENEILMEIKYVFW--------- : 137

mollusk_XP_011440496.1 : VGDQNVVHMEEN----------------------------------------------AKRLIEDPKEF-RLSRNEVAIGFVNAILCPD-------------------------------- : 83

mollusk_XP_013061423.1 : GKPEKYKGFRHFVDALKIDP--------------------------------------VNKLIADKITGTESKSDTSLKNKTNPDMLNETVRKILLSSLT--------------------- : 123

mollusk_XP_013061425.1 : SR-LGKDGPNNVN---------------------------------------------LEFLQKQDVTG------KVFLSMTEEDFDKLLPEA---------------------------- : 73

Musmu_NP_034286.2 : ---TEDLNIVEKY---------------------------------------------AQILFKEEVTG------MVLQELTEEDLREM-------------------------------- : 58

rept_XP_008110733.2 : ---ISELKLEEKH---------------------------------------------GNILLHQDVTG------SILKILTKEDLLAM-------------------------------- : 78

rept_XP_008112638.1 : ---LESIGIKKVY---------------------------------------------VEKLCEEEVTG------PALKILDDKFLKGM-------------------------------- : 54

rept_XP_015264172.1 : ---TDELKLDEKH---------------------------------------------GNTLLQQDVTG------TSLKLLQMQDLIAM-------------------------------- : 88

shark_XP_007886940.1 : ---KSTKGTSRHY------------------------------------------------------------------------------------------------------------ : 15

Strpu_XP_003727489.1 : ----------------------------------------------------------IEPIDEPMITG------EDEAGAGEADP----------------------------------- : 28

Strpu_XP_011667260.1 : ----------------------------------------------------------ELVSKFEGVDG------FTLVRCSKADLREDFEL----------------------------- : 42

turtle_XP_006139744.1 : ---TEELKIDQKY---------------------------------------------GEILVSQDVTG------SALKLFTLKDLKDI-------------------------------- : 70

turtle_XP_007060369.1 : ---TEELKIDQKY---------------------------------------------GEILFSQDVTG------SNLKLFTTEHLTAI-------------------------------- : 70

turtle_XP_014430084.1 : ---LESIGIKEEY---------------------------------------------VKKLREVEVTG------PVLRKINESFLKNI-------------------------------- : 54

Xenla_OCT63184.1 : ---LRSVHVKENY---------------------------------------------IEKLLEEEVNG------EALAELDATFLRQNL------------------------------- : 55

Xenla_XP_018122800.1 : ---TQCLNIGMDE---------------------------------------------GDILYKENVTG------QVLNILCKRDFVDM-------------------------------- : 58

Xentr_XP_002943568.3 : SSSLKGTTICTAV---------------------------------------------VQTPLADPCST------PEPPLVSQTPLAEPCS------------------------------ : 87

Xentr_XP_012813408.1 : ---LHSIRIKEEY---------------------------------------------VAKMFEEEVTG------PVLKKITEKYLKK-L------------------------------- : 54

* 380 * 400 * 420 * 440 * 460 * 480

Homsa_NP_060124.2 : -------GI--------------------------------------------------------------THGPAIQIEEL-----------FKELR--------------------KTA : 78

Homsa_NP_689916.2 : -------GL--------------------------------------------------------------PWGPALLIKRS-----------YNKLN--------------------SKS : 79

fish_XP_015241820.1 : ------------------------------------------------------------------------------------------------------------------------- : -

fish_XP_018543893.1 : ------------------------------------------------------------------------------------------------------------------------- : -

fish_CDQ83481.1 : ------------------------------------------------------------------------------------------------------------------------- : -

fish_CDQ89582.1 : ------------------------------------------------------------------------------------------------------------------------- : -

fish_XP_005809248.1 : ------------------------------------------------------------------------------------------------------------------------- : -

fish_XP_007231320.1 : ------------------------------------------------------------------------------------------------------------------------- : -

fish_XP_007254667.1 : ------------------------------------------------------------------------------------------------------------------------- : -

fish_XP_008277954.1 : ------------------------------------------------------------------------------------------------------------------------- : -

fish_XP_010746900.1 : ------------------------------------------------------------------------------------------------------------------------- : -

fish_XP_012685053.1 : ------------------------------------------------------------------------------------------------------------------------- : -

fish_XP_013123332.1 : ------------------------------------------------------------------------------------------------------------------------- : -

fish_XP_014011897.1 : ------------------------------------------------------------------------------------------------------------------------- : -

fish_XP_014011904.1 : ------------------------------------------------------------------------------------------------------------------------- : -

fish_XP_014264868.1 : ------------------------------------------------------------------------------------------------------------------------- : -

fish_XP_014264875.1 : ------------------------------------------------------------------------------------------------------------------------- : -

fish_XP_014854842.1 : ------------------------------------------------------------------------------------------------------------------------- : -

fish_XP_017578465.1 : ------------------------------------------------------------------------------------------------------------------------- : -

fish_XP_012713132.1 : ------------------------------------------------------------------------------------------------------------------------- : -

fish_XP_010787190.1 : ------------------------------------------------------------------------------------------------------------------------- : -

fish_XP_017319342.1 : -------GM--------------------------------------------------------------KSGPAHLIIES-----------RNEWL--------------------K-- : 82

Alligator_KYO30992.1 : -----------------------------------------------------------------------PRTTTNIQTRE--------------------------------------- : 72

Alligator_XP_006025954.1 : -------GI--------------------------------------------------------------THGPALQIIYA-----------LKQ-M--------------------SIL : 90

amphibia_XP_018413082.1 : -------GI--------------------------------------------------------------NHGPAVWIAHH-----------LKKLN--------------------E-- : 82

bact_AGA70736.1 : ------------------------------------------------------------------------------------------------------------------------- : -

bact_CUJ86040.1 : -------GLF-------------------------------------------------------------GSGYARLIELL--------------------------------------- : 58

bact_ESW58583.1 : -----------------------------------------------------------------------RDHTAFIVIGV--------------------------------------- : 16

bact_GAC67715.1 : -----------------------------------------------------------------------TGSASDLALGL--------------------------------------- : 75

bact_KMO66808.1 : -----------------------------------------------------------------------RDGQYVAKVQA--------------------------------------- : 71

bact_KUO79116.1 : -----------------------------------------------------------------------REGYYILKLSD-----------TRSIW----------------------- : 67

bact_KYG06363.1 : -----------------------------------------------------------------------RDGVYILKVTP--------------------------------------- : 60

bact_ODA70488.1 : -----------------------------------------------------------------------PLGDGDGVLNGQYILKAGFSTRRPQAD----------------------- : 84

bact_OGP49286.1 : -----------------------------------------------------------------------NFKEYLKAIDF-----------LNELA----------------------- : 80

bact_SCF05533.1 : -----------------------------------------------------------------------RVGPAADNLVQ--------------------------------------- : 73

bact_SCX17622.1 : ------------------------------------------------------------------------------------------------------------------------- : -

bact_SFJ80976.1 : -----------------------------------------------------------------------RVGPAEDGLTE-----------ANELA----------------------A : 79

bact_WP_006128369.1 : -----------------------------------------------------------------------RVGPNDDGLIQ-----------RNELA----------------------- : 106

bact_WP_006365791.1 : ------------------------------------------------------------------------------------------------------------------------- : -

bact_WP_006849212.1 : -----------------------------------------------------------------------RNKVCNLVQ----------------------------------------- : 79

bact_WP_007897046.1 : -----------------------------------------------------------------------MVSSKRLLIHN--------------------------------------- : 11

bact_WP_030194308.1 : -------GF--------------------------------------------------------------STGRSQAAAHH--------------------------------------- : 87

bact_WP_030198971.1 : -----------------------------------------------------------------------GDGRARLEGQY--------------------------------------- : 70

bact_WP_030898614.1 : -----------------------------------------------------------------------EPGRHAKALEE--------------------------------------- : 94

bact_WP_041219555.1 : ------------------------------------------------------------------------------------------------------------------------- : -

bact_WP_050505446.1 : -----------------------------------------------------------------------RSGDTRLVTSL--------------------------------------- : 70

bact_WP_055538636.1 : -----------------------------------------------------------------------GDGRARLEGQY-------------------------------------IL : 72

bact_WP_062134708.1 : ------------------------------------------------------------------------------------------------------------------------- : -

bact_WP_062799339.1 : -----------------------------------------------------------------------HSDPAIRGRIE--------------------------------------- : 44

bact_WP_066463897.1 : -----------------------------------------------------------------------YSGAKQQLIPA--------------------------------------- : 61

bird_XP_002194798.1 : -------GM--------------------------------------------------------------KKGQIQILIHK-----------RNELL--------------------RLQ : 75

bird_XP_005010984.1 : -------GI--------------------------------------------------------------PHGPAIQIIHF-----------LKK-H--------------------GIL : 85

bird_XP_005505023.1 : -------GI--------------------------------------------------------------PHGPALQIMHF-----------REK-H--------------------DIV : 85

bird_XP_008492486.1 : -------GI--------------------------------------------------------------PYGTALQIMYF-----------LKQ-H--------------------DIL : 85

bird_XP_008628214.1 : -------GM--------------------------------------------------------------PHGPAFQITCV-----------LKE-L--------------------DIL : 85

bird_XP_009068034.1 : -------GM--------------------------------------------------------------KGGQIQILICK-----------RREIP--------------------QPR : 73

bird_XP_009080343.1 : -------GI--------------------------------------------------------------PHEPALQIMYF-----------LKE-H--------------------GVI : 85

bird_XP_009486154.1 : -------GI--------------------------------------------------------------PHGPALHIMHF-----------LEKHH----------------VLAKGSN : 90

bird_XP_009640122.1 : -------GI--------------------------------------------------------------PHGPALQIMHF-----------LKEHG----------------ILAKGSN : 90

bird_XP_009672623.1 : -------GL--------------------------------------------------------------TYGPAFQIIHV-----------LRQ-N--------------------NIL : 85

bird_XP_009695665.1 : -------GM--------------------------------------------------------------KGGQIHMLIRK-----------RNELL--------------------QLQ : 221

bird_XP_009886214.1 : -------GI--------------------------------------------------------------PLGPALQITYF-----------LKEHD----------------ILAKGSN : 90

bird_XP_009934438.1 : -------GI--------------------------------------------------------------PHGPALQIMHF-----------LKEHD----------------ILAKGSN : 90

bird_XP_009999877.1 : -------GI--------------------------------------------------------------PHEPALQILHF-----------LKQ-H--------------------GIL : 85

bird_XP_010119033.1 : -------GI--------------------------------------------------------------PHGPTLEIMHF-----------LKE-H--------------------YNL : 83

bird_XP_010144931.1 : -------GI--------------------------------------------------------------PQGPAFQIMYF-----------LKE-H--------------------DVL : 85

bird_XP_010222910.1 : -------GI--------------------------------------------------------------THGPALRIIHV-----------LRQ-N--------------------NIL : 85

bird_XP_013160560.1 : -------GI--------------------------------------------------------------PHGPALQIIYF-----------LKE-R--------------------GIL : 89

bird_XP_014795808.1 : -------GI--------------------------------------------------------------PFGPTIRIMHF-----------LKEHD----------------NLAKGSN : 90

bird_XP_015156587.1 : -------SI--------------------------------------------------------------PHGPAVQIIHF-----------LKKHG----------------ILTESSD : 86

bird_XP_015709021.1 : -------SI--------------------------------------------------------------PHGPAVQIIHF-----------LKK-H--------------------GIV : 92

brachiop_XP_013415988.1 : -----------------------------------------------------------------------EYSPEATCMEK-----------NEVLLTLLTQKNDQAFNALCEALRQSGL : 153

Cioin_XP_018668123.1 : -----------------------------------------------------------------------ELKKEYTVIDQ--------------------------------------- : 57

Cnidaria_EDO35430.1 : -----------------------------------------------------------------------RNDLASKLADK-----------KETFKDSSVQINQSDQQVNSPIQETTPQ : 118

Cnidaria_KXJ09690.1 : -----------------------------------------------------------------------KSEAKSSEEAI-----------KDELERVYPDHDAITNLHEKSFISNQSS : 124

Cnidaria_KXJ11435.1 : -----------------------------------------------------------------------EETPTKEIKNA-----------FERVTSTDSQDKSNPTRETLTKDKRKKR : 129

Cnidaria_KXJ17955.1 : KRKGKAAFKEK------------------------------------------------------------KFSKACQIYEEAIELISPRNLNREAVKERRHLRLKLIRSLICKISQNKQE : 166

Cnidaria_KXJ23466.1 : -----------------------------------------------------------------------QKLKKLSLDDKTVLVNLLRQIGRDDLASELESWKCLNENAKEIDKLTKKS : 117

Cnidaria_XP_015751626.1 : -------GSAEVPSELSPLQGSIEKAEYYNNRGKVEFQESRFENACQLYTSALKCLPSNLEKDDQRNKYLCNRAACYLKLRK---FKDAQEDCDSVLLRNPRDTKARFRLAQSLDGQGKTA : 202

Cnidaria_XP_015751629.1 : -------GSAEVPSELSPLQGSIEKAEYYNNRGKVEFQESRFENACQLYTSALKCLPSNLEKDDQRNKYLCNRAACYLKLRK---FKDAQEDCDSVLLRNPRDTKARFRLAQSLDGQGKTA : 202

Cnidaria_XP_015758975.1 : G----------------------------------------------------------------------SNGEKEKETNEGRLEGGQPPSVDDNLEPIYSNTTDTSSSLPSARDAVAEE : 307

Cnidaria_XP_015766106.1 : -----------------------------------------------------------------------SNQPSDAAAFD--------------------------------------- : 37

Cnidaria_XP_015766118.1 : -----------------------------------------------------------------------KKKRSKKMKNR--------------------------------DITAGNA : 84

Cnidaria_XP_015766124.1 : -----------------------------------------------------------------------LSGLATGMDEQ--------------------------------------- : 78

coelacanth_XP_006000513.2 : -------GI--------------------------------------------------------------KAGPTVMML----------------------------------------- : 47

fish_CDQ87765.1 : -------GV--------------------------------------------------------------KFGPAIQIIKN-------VEILRNDLESVGRSTRSPESDPFRRQERFRFR : 309

fish_CDQ92295.1 : -------EM--------------------------------------------------------------KGGQIQLMLHK-----------RDELL--------------------KPV : 83

fish_KKF13953.1 : -------CM--------------------------------------------------------------KSGPAHLILQK-----------RDELS--------------------NSK : 77

fish_XP_004085798.2 : -------GV--------------------------------------------------------------PRAPALQILRE-----------TEKFR----------------------- : 106

fish_XP_004552746.3 : -------GI--------------------------------------------------------------KHGPAVKITAY-----------LESLK----------------------- : 77

fish_XP_004558390.1 : -------YM--------------------------------------------------------------KSGPAHLIIQR-----------RDELI--------------------NSQ : 86

fish_XP_004576250.1 : -------GV--------------------------------------------------------------PPAPAIQIIKQ-----------VKKLRKHSETFNLSARLSESYYTDEFVE : 155

fish_XP_005161888.1 : -------GM--------------------------------------------------------------KAGPALLIIKE-----------RNEIL--------------------KNS : 77

fish_XP_005478813.2 : -------GI--------------------------------------------------------------KHGPAVKITAY-----------LESLK--------------------KAC : 80

fish_XP_005806405.2 : -------GM--------------------------------------------------------------KSGQIEHLLKK-----------RDDLL--------------------KTE : 90

fish_XP_006625674.1 : -------HI--------------------------------------------------------------KHGPAVRIINN-----------LEELKKAKQPSQDQKYLEEHEKASQPPQ : 99

fish_XP_006635547.1 : PRPPEPRGL--------------------------------------------------------------QDALALPCREQ-----------SRVWT--------------------RTR : 140

fish_XP_006635548.1 : -------GM--------------------------------------------------------------KPGQIQLLLSE-----------RDKLL--------------------GST : 76

fish_XP_006791552.1 : -------CM--------------------------------------------------------------KSGPAHLIIQK-----------RDELI--------------------NSQ : 79

fish_XP_006791631.1 : -------GM--------------------------------------------------------------KSGQIEHLLKK-----------RDELL--------------------IPQ : 83

fish_XP_007241837.1 : -------GM--------------------------------------------------------------KSGPALLIITK-----------RNELV--------------------KSV : 77

fish_XP_007242299.1 : -------GI--------------------------------------------------------------KHGPAVRIINT-----------LEALVQTHKNEPVLDIQKDTPQTQSEPS : 110

fish_XP_007557414.1 : -------CM--------------------------------------------------------------KSGPAHLIIQK-----------RDALL--------------------NS- : 76

fish_XP_008284284.1 : -------NV--------------------------------------------------------------TFGPAKLLIHA-----------RDELV----------------------- : 83

fish_XP_008284762.1 : -------GM--------------------------------------------------------------NSVQIEHLLRK-----------REELL--------------------KSG : 76

fish_XP_008285761.1 : -------CM--------------------------------------------------------------KLGPAHLIIQK-----------RDELL--------------------KLQ : 77

fish_XP_008336142.1 : -------GI--------------------------------------------------------------KHGPAVKISRY-----------LRCLK----------------------- : 73

fish_XP_008417602.1 : -------GV--------------------------------------------------------------PPAPAIQILRQ-----------IKKFK----------------------- : 153

fish_XP_009510531.1 : -------GI--------------------------------------------------------------PHGPALEIIDF-----------LKKHD----------------ILAQGSN : 90

fish_XP_010752845.1 : -------GI--------------------------------------------------------------KHGPAVKITSS-----------LKSLK----------------------- : 76

fish_XP_010755274.1 : -------GV--------------------------------------------------------------TFGPAKLIKHA--------------------------------------- : 78

fish_XP_010767524.1 : -------GI--------------------------------------------------------------KHGPAVKITSY-----------LESLK----------------------- : 75

fish_XP_010781233.1 : -------GV--------------------------------------------------------------TFGPAKLFIHA-----------RHELV----------------------- : 103

fish_XP_010791966.1 : --------M--------------------------------------------------------------KSGPAHLIIQK-----------RNELI--------------------NSK : 20

fish_XP_012687177.1 : -----------------------------------------------------------------------KADVKPKIKQR--------------------------------------- : 66

fish_XP_012708385.1 : -------SM--------------------------------------------------------------KSGPANLIIKR-----------RDELI--------------------NFT : 77

fish_XP_012713253.1 : -------GV--------------------------------------------------------------TFGPAKLVLHA-----------REELM----------------------- : 81

fish_XP_012727798.1 : -------GM--------------------------------------------------------------KSGQIEHLLKK-----------RDDIM--------------------KTD : 76

fish_XP_012990558.1 : -------KI--------------------------------------------------------------KHGPAVKMMSM-----------LEDLR----------------------- : 72

fish_XP_013126906.1 : -------GV--------------------------------------------------------------TFGPAKLLIRA-----------RDEVV----------------------- : 83

fish_XP_013796981.1 : -------DI--------------------------------------------------------------THGPALQIIHV-----------LRQ-N--------------------NIL : 85

fish_XP_014005049.1 : -----------------------------------------------------------------------PPGKTVTRKKE--------------------------------------- : 25

fish_XP_014049585.1 : -------GM--------------------------------------------------------------KSGPALLIIRK-----------RNELV--------------------DTK : 78

fish_XP_014325953.1 : -------GI--------------------------------------------------------------KHGPAVKIASH-----------LKSLT----------------------- : 71

fish_XP_015195770.1 : -------GI--------------------------------------------------------------KYGPAKLIVHK-----------KDELV--------------------RLK : 98

fish_XP_015197335.1 : -------GI--------------------------------------------------------------KYGPAVKIVKN-----------VRQLKV---------------------- : 77

fish_XP_015232478.1 : ----------------------------------------------------------------------------MRIVHA-----------RDELI--------------------NFK : 14

fish_XP_015252023.1 : -------GL--------------------------------------------------------------TFGPAKLIIHA-----------RDEVI----------------------- : 75

fish_XP_015264177.1 : -------GM--------------------------------------------------------------KGAQIYLLMSE-----------RKQLGESNTRPDSTQVVHDKVNMSNSKK : 96

fish_XP_015801149.1 : -------CM--------------------------------------------------------------KSGPAHLIIQK-----------RDALL--------------------NPL : 77

fish_XP_015810486.1 : -------GV--------------------------------------------------------------NFGPAKIIIHA--------------------------------------- : 76

fish_XP_016115105.1 : -------NL--------------------------------------------------------------SLNAKKLIIHN-----------KDLLK----------------------- : 89

fish_XP_016117315.1 : -------GI--------------------------------------------------------------KHGPAVMISNR-----------LKELKQPYGSDSASPEHSQTQQKEDGLD : 99

fish_XP_016395637.1 : -------GL--------------------------------------------------------------KSGPALLIIKK-----------RDELV---------------------NS : 76

fish_XP_017207716.1 : -------GI--------------------------------------------------------------KHGPAVIIINQ-----------LKEYRRIHEPHFASPEHSPTQQEEDRLE : 99

fish_XP_017277259.1 : -------GM--------------------------------------------------------------KSGQIEHLLKK-----------RDDLL--------------------KAE : 83

fish_XP_017277301.1 : -------CM--------------------------------------------------------------KAGPAHLIIQN-----------RDALL--------------------SSL : 77

fish_XP_017325460.1 : -------GI--------------------------------------------------------------KLGPAKLIIHH-----------RDELK----------------------- : 105

fish_XP_017346953.1 : -------GM--------------------------------------------------------------KSGPALLIIKK-----------RNELV--------------------NTL : 77

fish_XP_017550536.1 : -------GI--------------------------------------------------------------EYGPAVKIWSQ-----------LKK------------------------- : 86

fish_XP_017550824.1 : -------NI--------------------------------------------------------------KLGPAKLIIHR-----------RDELI----------------------- : 87

fish_XP_017554653.1 : -------GI--------------------------------------------------------------KHGPAVRIINT-----------LETLIKSHENVSDSEMPTDISQTQSPSE : 99

fish_XP_017569575.1 : -------GM--------------------------------------------------------------KSGYVHLIITK-----------RNELI--------------------NAS : 77

fish_XP_018413083.1 : -------KI--------------------------------------------------------------SYGPAAIIIHE-----------RTQMT--------------------KDR : 79

fish_XP_018429299.1 : -------GM--------------------------------------------------------------KQGQIQLLMQK-----------RNELL----------------------- : 97

fish_XP_018521334.1 : -------GI--------------------------------------------------------------KCGPAVKITSY-----------LERLK----------------------- : 77

fish_XP_018526467.1 : -------CM--------------------------------------------------------------KSGPAHLVIKK-----------RDELL--------------------DFQ : 77

fish_XP_018541041.1 : -------GV--------------------------------------------------------------PLAPAIQIIRQ-----------VEKFKRHSETFEPSMRTHMNYYELSKGE : 195

fish_XP_018558293.1 : -------NV--------------------------------------------------------------TFGPAKLIIHA-----------RDEVM----------------------- : 92

fish_XP_018595329.1 : -------GV--------------------------------------------------------------KHGPAIQIIQNVRKLTGSSERPKSQPKKEKTPQRVKLNSSAEVNKGVGTS : 110

fish_XP_018611696.1 : -------GM--------------------------------------------------------------KSGPALLIIRK-----------RNELI--------------------NSQ : 80

fish_XP_018956725.1 : -------GI--------------------------------------------------------------KHGPAVKIITR-----------LEMLKKEQQELPDNSYTQHTEASVDTTV : 102

frog_XP_018091031.1 : -------GM--------------------------------------------------------------KEGQIQLLISK-----------RDKLLLEQKSTEPGSPSQNTQASSSHQE : 96

Hemichordata_XP_006825609.1 : NLEEWTPNHVELWAKSINLDDKYIQVFKDEEYDGDALRFLTPEKIQRIFSM--------------------RKGPMRKLLYRIYAIADTLVDGRKQLTSESQMGYSTSAMSQGPRKFPHTS : 375

lance_EEN48978.1 : -----------------------------------------------------------------------TLGKVINLRYS--------------------------------------- : 51

lance_EEN55742.1 : -----------------------------------------------------------------------KGGMAAAVGNT--------------------------------------- : 60

mammal_XP_001378512.1 : -------GI--------------------------------------------------------------KHGPALQIINS-----------FKELN----------------KSSECPK : 83

mammal_XP_003771993.1 : -------GL--------------------------------------------------------------KHGPALQIINM-----------FKELN------------------SECFK : 81

mammal_XP_003771994.1 : -------GI--------------------------------------------------------------PHGPALLIMRT-----------IEKMK--------------------NIP : 76

mammal_XP_003782717.1 : -------GI--------------------------------------------------------------THGPAIQIEEL-----------FKELL--------------------RTS : 78

mammal_XP_003982796.1 : -------GL--------------------------------------------------------------PRGPALLIKRT-----------YNRLNNRYPESNNQDSGQLDHTKPSLQE : 99

mammal_XP_004389713.1 : -------GL--------------------------------------------------------------PQGPVLLIKRG-----------YNKLN--------------------NSS : 79

mammal_XP_004582357.1 : -------GI--------------------------------------------------------------THGPAIQIEEL-----------FKEL------------------------ : 73

mammal_XP_004582358.1 : -------GL--------------------------------------------------------------PRGPALLIRRM-----------YNKLH--------------------NST : 79

mammal_XP_004622892.1 : -------GL--------------------------------------------------------------PRGPALLIKRT-----------YNKLN--------------------NIS : 79

mammal_XP_004646313.1 : -------GL--------------------------------------------------------------PPGPALLIKRT-----------YDKLK--------------------TSF : 79

mammal_XP_004702866.1 : -------GL--------------------------------------------------------------PKGPALLIKRA-----------FNKLN--------------------NSS : 79

mammal_XP_005340592.1 : -------GL--------------------------------------------------------------PRGPALLIKRT-----------YNKLI--------------------NSC : 79

mammal_XP_005388084.1 : -------GL--------------------------------------------------------------PRGPALLIKRM-----------YNKLN--------------------TSF : 79

mammal_XP_006163552.1 : -------GL--------------------------------------------------------------PRGPALLIKRM-----------YSKLN--------------------NTS : 79

mammal_XP_006779273.1 : -------GL--------------------------------------------------------------PRGPALLIKRM-----------YDRLNNIFPENHNQDSEQLEHIKASKKH : 99

mammal_XP_006834357.1 : -------GL--------------------------------------------------------------PRGPALLIKRG-----------YNKLN--------------------TSS : 79

mammal_XP_006891533.1 : -------GL--------------------------------------------------------------PRGPALLIKRG-----------YNKLN--------------------NSR : 79

mammal_XP_007522555.1 : -------GL--------------------------------------------------------------PRGPALLIKRK-----------FKSLN--------------------NLS : 79

mammal_XP_007938002.1 : -------GL--------------------------------------------------------------PRGPALLIKRG-----------YNKLN--------------------NSS : 79

mammal_XP_008062458.1 : -------GI--------------------------------------------------------------THGPAIRTEEL-----------SKELQ--------------------KTS : 78

mammal_XP_008259950.1 : -------GL--------------------------------------------------------------PRGPALLIKRA-----------CNKLL--------------------NSS : 79

mammal_XP_008524371.1 : -------GL--------------------------------------------------------------PRGPAILIKRA-----------YNRLN--------------------NSS : 79

mammal_XP_008820771.1 : -------GL--------------------------------------------------------------PRGPALLIERA-----------YNKLN--------------------THS : 79

mammal_XP_010601767.1 : -------GL--------------------------------------------------------------PRGPALLIKRM-----------YNKLN--------------------TSL : 79

mammal_XP_011853657.1 : -------GL--------------------------------------------------------------PRGPALLIKRS-----------YNKLN--------------------SKS : 79

mammal_XP_012791283.1 : -------GV--------------------------------------------------------------THGPAILIENL-----------VKKLQ--------------------KTS : 78

mammal_XP_012881542.1 : -------GI--------------------------------------------------------------PRGPSLLIKRT-----------YNKLS--------------------NSS : 79

mammal_XP_012934202.1 : -------GI--------------------------------------------------------------THGPAIQIEGL-----------FKELQ--------------------KTS : 84

mammal_XP_013013639.1 : -------GL--------------------------------------------------------------PRGPALLIKRM-----------YNKLN--------------------TSF : 79

mammal_XP_016016798.1 : -------GL--------------------------------------------------------------PRGPALLIKRM-----------YGKLNNSFPENNNQGSGQLDHTNPSKSE : 99

mammal_XP_016281899.1 : -------GI--------------------------------------------------------------PNGPALLIMRT-----------YKKLR--------------------NIS : 76

mammal_XP_017199853.1 : -------GI--------------------------------------------------------------THGPAVQIEGL-----------FRELQ--------------------KTS : 78

mammal_XP_017508280.1 : -------GL--------------------------------------------------------------PRGPALLIKRT-----------YDRLY--------------------NSA : 88

mollusk_EKC42356.1 : -----------------------------------------------------------------------KRGSTTRCPNH--------------------------------------- : 66

mollusk_XP_011436570.1 : -----------------------------------------------------------------------HDGKEYSEQEVKTSLNECIKMLFPQAIRKKVKRMNKKLWSYCNIDRKEPD : 187

mollusk_XP_011440496.1 : -----------------------------------------------------------------------GSGPVKGIPLL--------------------------------------- : 94

mollusk_XP_013061423.1 : -------CI--------------------------------------------------------------PKETIPVSEVRELLKAKSVFSGQDEWSNEKLIEFVKETFTRVEVTKVKRP : 175

mollusk_XP_013061425.1 : -----------------------------------------------------------------------TFGARRSLSML-----------VAELT----------------------- : 89

Musmu_NP_034286.2 : -------GL--------------------------------------------------------------PRGPALLIKRM-----------YNKL---------------------ISS : 78

rept_XP_008110733.2 : -------GM--------------------------------------------------------------PHGPAIKIMHA-----------VKG-N--------------------GDP : 98

rept_XP_008112638.1 : -------GM--------------------------------------------------------------KQGQIHILIQK-----------RDKLLQGNIG------YQDKIGGVTALD : 89

rept_XP_015264172.1 : -------GI--------------------------------------------------------------TYGPAIQIMHA-----------LKG-K--------------------DDP : 108

shark_XP_007886940.1 : ------------------------------------------------------------------------------------------------------------------------- : -

Strpu_XP_003727489.1 : -----------------------------------------------------------------------TIQRLVAELHN-----------FEKWR----------------------- : 44

Strpu_XP_011667260.1 : -----------------------------------------------------------------------SAGYCKSLMLR-----------REHYLAKERMDMLTTPKSALSDSRDGNY : 81

turtle_XP_006139744.1 : -------GI--------------------------------------------------------------THGPALQIMDA-----------LK--C--------------------NFL : 89

turtle_XP_007060369.1 : -------GI--------------------------------------------------------------TYGPALQILNA-----------LK--F--------------------NFL : 89

turtle_XP_014430084.1 : -------GM--------------------------------------------------------------KEGQIYVLICE-----------RDELLEYLSKKTSAEQAENPTFKATEIL : 95

Xenla_OCT63184.1 : -------QM--------------------------------------------------------------KEGQIQLLIKK-----------RDSLL----------------------- : 73

Xenla_XP_018122800.1 : -------GI--------------------------------------------------------------TIGTACIIMHR-----------LKTMS--------------------T-- : 77

Xentr_XP_002943568.3 : -----------------------------------------------------------------------TPGPPLVPQTP-----------LAEPCSTLGPPLVPQTPLADPCSTPEPP : 126

Xentr_XP_012813408.1 : -------GM--------------------------------------------------------------KAGQIHLLLVS-----------RDELCVQQSS------------------ : 77

* 500 * 520 * 540 * 560 * 580 * 600

Homsa_NP_060124.2 : I--------------------------------------------------EDS---IQTSKM---GKP-----------------SKN----AP-------------------------- : 96

Homsa_NP_689916.2 : P--------------------------------------------------ESD---NHDPGQLDNSKP-----------------SKT----EH-------------------------- : 100

fish_XP_015241820.1 : ------------------------------------------------------------------------------------------------------------------------- : -

fish_XP_018543893.1 : ------------------------------------------------------------------------------------------------------------------------- : -

fish_CDQ83481.1 : ------------------------------------------------------------------------------------------------------------------------- : -

fish_CDQ89582.1 : ------------------------------------------------------------------------------------------------------------------------- : -

fish_XP_005809248.1 : ------------------------------------------------------------------------------------------------------------------------- : -

fish_XP_007231320.1 : ------------------------------------------------------------------------------------------------------------------------- : -

fish_XP_007254667.1 : ------------------------------------------------------------------------------------------------------------------------- : -

fish_XP_008277954.1 : ------------------------------------------------------------------------------------------------------------------------- : -

fish_XP_010746900.1 : ------------------------------------------------------------------------------------------------------------------------- : -

fish_XP_012685053.1 : ------------------------------------------------------------------------------------------------------------------------- : -

fish_XP_013123332.1 : ------------------------------------------------------------------------------------------------------------------------- : -

fish_XP_014011897.1 : ------------------------------------------------------------------------------------------------------------------------- : -

fish_XP_014011904.1 : ------------------------------------------------------------------------------------------------------------------------- : -

fish_XP_014264868.1 : ------------------------------------------------------------------------------------------------------------------------- : -

fish_XP_014264875.1 : ------------------------------------------------------------------------------------------------------------------------- : -

fish_XP_014854842.1 : ------------------------------------------------------------------------------------------------------------------------- : -

fish_XP_017578465.1 : ------------------------------------------------------------------------------------------------------------------------- : -

fish_XP_012713132.1 : ------------------------------------------------------------------------------------------------------------------------- : -

fish_XP_010787190.1 : ------------------------------------------------------------------------------------------------------------------------- : -

fish_XP_017319342.1 : --------------------------------------------------------KVQKQ--QNPRE----------------------------------------------------- : 92

Alligator_KYO30992.1 : -----------------------------------------------------------GVGDLGTIS----------------------------------------------------- : 81

Alligator_XP_006025954.1 : T--------------------------------------------------EDP---SQASGQEDAKKP-----------------IGEKYLIEK-------------------------- : 115

amphibia_XP_018413082.1 : -----------------------------------------------------T---CKDQGQQSNLGV------------------------NK-------------------------- : 97

bact_AGA70736.1 : ------------------------------------------------------------------------------------------------------------------------- : -

bact_CUJ86040.1 : ------------------------------------------------------------------------------------------------------------------------- : -

bact_ESW58583.1 : ------------------------------------------------------------------------------------------------------------------------- : -

bact_GAC67715.1 : -----------------------------------------------------------GTYAETHVPR---------------------------------------------------- : 85

bact_KMO66808.1 : -----------------------------------------------------------ARTAHGNPN----------------------------------------------------- : 80

bact_KUO79116.1 : ------------------------------------------------------------------------------------------------------------------------- : -

bact_KYG06363.1 : ------------------------------------------------------------------------------------------------------------------------- : -

bact_ODA70488.1 : -----------------------------------------------------------AHNAFVQGLE---------------------------------------------------- : 94

bact_OGP49286.1 : ------------------------------------------------------------------------------------------------------------------------- : -

bact_SCF05533.1 : -----------------------------------------------------------PNERAAHRLIRQ-------------------------------------------------- : 85

bact_SCX17622.1 : ------------------------------------------------------------------------------------------------------------------------- : -

bact_SFJ80976.1 : H----------------------------------------------------------QRIRDRA------------------------------------------------------- : 87

bact_WP_006128369.1 : ------------------------------------------------------------------------------------------------------------------------- : -

bact_WP_006365791.1 : ------------------------------------------------------------------------------------------------------------------------- : -

bact_WP_006849212.1 : ------------------------------------------------------------------------------------------------------------------------- : -

bact_WP_007897046.1 : ------------------------------------------------------------------------------------------------------------------------- : -

bact_WP_030194308.1 : ------------------------------------------------------------------------------------------------------------------------- : -

bact_WP_030198971.1 : -----------------------------------------------------------ILKVSDDPN----------------------------------------------------- : 79

bact_WP_030898614.1 : ------------------------------------------------------------------------------------------------------------------------- : -

bact_WP_041219555.1 : ------------------------------------------------------------------------------------------------------------------------- : -

bact_WP_050505446.1 : --------------------------------------------------------HQLAGGSLSQYT----------------------------------------------------- : 82

bact_WP_055538636.1 : K----------------------------------------------------------VSDDPD-------------------------------------------------------- : 79

bact_WP_062134708.1 : ------------------------------------------------------------------------------------------------------------------------- : -

bact_WP_062799339.1 : ------------------------------------------------------------------------------------------------------------------------- : -

bact_WP_066463897.1 : ------------------------------------------------------------------------------------------------------------------------- : -

bird_XP_002194798.1 : D---------------------------------------------------------NAQQAEDSSS----------------------------------------------------- : 86

bird_XP_005010984.1 : T--------------------------------------------------ESS---NQAEGQEGSEQS-----------------IGG----E--------------------------- : 105

bird_XP_005505023.1 : A--------------------------------------------------KGS---NQAVEQEGTEQS-----------------LDG----EG-------------------------- : 106

bird_XP_008492486.1 : P--------------------------------------------------KVS---NQTMEQEDTAEC-----------------LDG----EG-------------------------- : 106

bird_XP_008628214.1 : G--------------------------------------------------KGS---NQAVEQEGTEES-----------------FDG----GG-------------------------- : 106

bird_XP_009068034.1 : D---------------------------------------------------------NAQQAKDSSS----------------------------------------------------- : 84

bird_XP_009080343.1 : D--------------------------------------------------KGS---DQAVEQEDTQQS-----------------LDG----EG-------------------------- : 106

bird_XP_009486154.1 : Q----------------------------------------------------------AAEQEGTEQSLDGEG----------------------------------------------- : 106

bird_XP_009640122.1 : Q----------------------------------------------------------AVGQEGTEQSLDG------------------------------------------------- : 104

bird_XP_009672623.1 : P--------------------------------------------------ESS---NQAVGQEDTKHG---------------------------------------------------- : 101

bird_XP_009695665.1 : E---------------------------------------------------------NAQHTKHSSS----------------------------------------------------- : 232

bird_XP_009886214.1 : Q----------------------------------------------------------ALDQESTGQSFDGEG----------------------------------------------- : 106

bird_XP_009934438.1 : E----------------------------------------------------------AVEQEGTEQSIGGE------------------------------------------------ : 105

bird_XP_009999877.1 : A--------------------------------------------------EGS---NQAVEEEGSEQN-----------------LTG----E--------------------------- : 105

bird_XP_010119033.1 : A--------------------------------------------------TGA---KQAVEQENSKQS-----------------LDG----EG-------------------------- : 104

bird_XP_010144931.1 : A--------------------------------------------------KGS---NQAVEQEDTEQS-----------------LNG----EG-------------------------- : 106

bird_XP_010222910.1 : P--------------------------------------------------ESS---NQAVGQEDTKHD-----------------PDG----NG-------------------------- : 106

bird_XP_013160560.1 : P--------------------------------------------------KGS---NQAVEQEGTEQS-----------------LDG----EG-------------------------- : 110

bird_XP_014795808.1 : Q----------------------------------------------------------AVDQEGTEQCLDGEG----------------------------------------------- : 106

bird_XP_015156587.1 : Q----------------------------------------------------------AMGQEGNEQCLDG------------------------------------------------- : 100

bird_XP_015709021.1 : A--------------------------------------------------ESS---DPAMGQEGSEQC-----------------LDG----K--------------------------- : 112

brachiop_XP_013415988.1 : A------------------------------------------------------------------------------------------------------------------------ : 154

Cioin_XP_018668123.1 : ------------------------------------------------------------------------------------------------------------------------- : -

Cnidaria_EDO35430.1 : K------------------------------------------------------------------------------------------------------------------------ : 119

Cnidaria_KXJ09690.1 : E----------------------------------------------------------STAKRKKNKKKDSL------------------------------------------------ : 139

Cnidaria_KXJ11435.1 : N----------------------------------------------------------KKSNPEAKE----------------------------------------------------- : 139

Cnidaria_KXJ17955.1 : N----------------------------------------------------------EIDHDQYKRKLHEAKTACLELLNYDPEDFKAKFLLSKCLVGLEQYDDAFKELTILLKIQPKN : 229

Cnidaria_KXJ23466.1 : RVFQ-------------------------------------------------------KEGKFCSESEIYGKILELCSNDEDGKLSRCKFLKKR-------------------------- : 157

Cnidaria_XP_015751626.1 : E----------------------------------------------------------AFTEVTSLLCINPESVAIVHLESSLRGKIEDSKQKALEKQRQGKEYFEKSNFRAAILKFSEG : 265

Cnidaria_XP_015751629.1 : E----------------------------------------------------------AFTEVTSLLCINPESVAIVHLESSLRGKIEDSKQKALEKQRQGKEYFEKSNFRAAILKFSEG : 265

Cnidaria_XP_015758975.1 : D----------------------------------------------------------AGSSSTSGEEVTQGGLNCSGTMQRRNIPSSKPELGVGLVGNDVLVKT--------------- : 355

Cnidaria_XP_015766106.1 : ------------------------------------------------------------------------------------------------------------------------- : -

Cnidaria_XP_015766118.1 : K----------------------------------------------------------GNRSQSPPA----------------------------------------------------- : 94

Cnidaria_XP_015766124.1 : ------------------------------------------------------------------------------------------------------------------------- : -

coelacanth_XP_006000513.2 : ---------------------------------------------------------------------------------------------RE-------------------------- : 49

fish_CDQ87765.1 : A----------------------------------------------------------IPEARMGSHEETL------------------------------------------------- : 323

fish_CDQ92295.1 : P---------------------------------------------------------QKTKAVSSTQ----------------------------------------------------- : 94

fish_KKF13953.1 : Q-------------------------------------------------------KSQVK--KK-------------------------------------------------------- : 85

fish_XP_004085798.2 : --------------------------------------------------------RHSDTLVSSVGT----------------------------------------------------- : 118

fish_XP_004552746.3 : ------------------------------------------------------------------------------------------------------------------------- : -

fish_XP_004558390.1 : Q-------------------------------------------------------KCQEK--KKTTG----------------------------------------------------- : 97

fish_XP_004576250.1 : T------------------------------------------------------------------------------------------------------------------------ : 156

fish_XP_005161888.1 : Q-------------------------------------------------------KAQSK--PKQPS----------------------------------------------------- : 88

fish_XP_005478813.2 : K----------------------------------------------------------HQSEFPEYVE---------------------------------------------------- : 91

fish_XP_005806405.2 : Q---------------------------------------------------------SKTNTKTVTS----------------------------------------------------- : 101

fish_XP_006625674.1 : VQKNLKELQNAKEPLQEQKNLEELDKAKQPSQVQKNLGELQNAKQPSQEQKNLEEHQKAKQPSQEQENLEELEKAKQLSQNLKVLQNAKQ------------------------------- : 189

fish_XP_006635547.1 : E--------------------------------------------------YRNRRAPVDG--ERERG----------------------------------------------------- : 156

fish_XP_006635548.1 : E---------------------------------------------------------SNKPEKKQTG----------------------------------------------------- : 87

fish_XP_006791552.1 : Q-------------------------------------------------------KCQEK--KKTTG----------------------------------------------------- : 90

fish_XP_006791631.1 : Q---------------------------------------------------------QKIK-KSDLS----------------------------------------------------- : 93

fish_XP_007241837.1 : H-------------------------------------------------------KGQRQ--HDKAV----------------------------------------------------- : 88

fish_XP_007242299.1 : G----------------------------------------------------------SSTTQCTESRHDTSVNTALPKTEQPQQKSKKKKQKSHKKGLGN------------------- : 154

fish_XP_007557414.1 : ----------------------------------------------------------QKN--KQVTR----------------------------------------------------- : 84

fish_XP_008284284.1 : ------------------------------------------------------------------------------------------------------------------------- : -

fish_XP_008284762.1 : Q------------------------------------------------------------------------------------------------------------------------ : 77

fish_XP_008285761.1 : Q-------------------------------------------------------QCQEK--KKPSG----------------------------------------------------- : 88

fish_XP_008336142.1 : ------------------------------------------------------------------------------------------------------------------------- : -

fish_XP_008417602.1 : --------------------------------------------------------KQSETFVPATPV----------------------------------------------------- : 165

fish_XP_009510531.1 : Q----------------------------------------------------------AVEQEGTEQSLD-EG----------------------------------------------- : 105

fish_XP_010752845.1 : ------------------------------------------------------------------------------------------------------------------------- : -

fish_XP_010755274.1 : ------------------------------------------------------------------------------------------------------------------------- : -

fish_XP_010767524.1 : ------------------------------------------------------------------------------------------------------------------------- : -

fish_XP_010781233.1 : ------------------------------------------------------------------------------------------------------------------------- : -

fish_XP_010791966.1 : Q-------------------------------------------------------KPQEK--IKPNP----------------------------------------------------- : 31

fish_XP_012687177.1 : ------------------------------------------------------------------------------------------------------------------------- : -

fish_XP_012708385.1 : Q-------------------------------------------------------KPPEK--NTSSS----------------------------------------------------- : 88

fish_XP_012713253.1 : ------------------------------------------------------------------------------------------------------------------------- : -

fish_XP_012727798.1 : Q---------------------------------------------------------SKTKTQTETS----------------------------------------------------- : 87

fish_XP_012990558.1 : ------------------------------------------------------------------------------------------------------------------------- : -

fish_XP_013126906.1 : ------------------------------------------------------------------------------------------------------------------------- : -

fish_XP_013796981.1 : P--------------------------------------------------ESS---NQAVGQEDAKHG-----------------LDG----KG-------------------------- : 106

fish_XP_014005049.1 : ------------------------------------------------------------------------------------------------------------------------- : -

fish_XP_014049585.1 : Q-------------------------------------------------------RTQGK--EKTQS----------------------------------------------------- : 89

fish_XP_014325953.1 : ------------------------------------------------------------------------------------------------------------------------- : -

fish_XP_015195770.1 : K--------------------------------------------------EQP----STQGI---------------------------------------------------------- : 107

fish_XP_015197335.1 : -----------------------------------------------------------SPGRILKFQT---------------------------------------------------- : 87

fish_XP_015232478.1 : R--------------------------------------------------VEP---TCSRNQ---------------------------------------------------------- : 24

fish_XP_015252023.1 : -----------------------------------------------------------NLKSKKPTGSSS-------------------------------------------------- : 87

fish_XP_015264177.1 : T----------------------------------------------------------KTGGENTKH----------------------------------------------------- : 106

fish_XP_015801149.1 : Q-------------------------------------------------------NNQGN--KKPTG----------------------------------------------------- : 88

fish_XP_015810486.1 : ------------------------------------------------------------------------------------------------------------------------- : -

fish_XP_016115105.1 : ------------------------------------------------------------------------------------------------------------------------- : -

fish_XP_016117315.1 : T----------------------------------------------------------TEEIQDLSTDQTQISKYTAKKSKTKSSKKGK------------------------------- : 131

fish_XP_016395637.1 : Q-------------------------------------------------------KARSKHLHKQTS----------------------------------------------------- : 89

fish_XP_017207716.1 : K--------------------------------------------------------PEEIQDLSSDQMPRHTMK---------------------------------------------- : 118

fish_XP_017277259.1 : Q---------------------------------------------------------CKIKNKTGSS----------------------------------------------------- : 94

fish_XP_017277301.1 : Q-------------------------------------------------------KCQGN--KKSTS----------------------------------------------------- : 88

fish_XP_017325460.1 : -----------------------------------------------------------AHQLKNSGR----------------------------------------------------- : 114

fish_XP_017346953.1 : Q-------------------------------------------------------KAQKQ--KNNTP----------------------------------------------------- : 88

fish_XP_017550536.1 : ------------------------------------------------------------------------------------------------------------------------- : -

fish_XP_017550824.1 : ------------------------------------------------------------------------------------------------------------------------- : -

fish_XP_017554653.1 : P-------------------------------------------------------PGCSRTQDTEGKSDTLLNDDMSVSVT--------------------------------------- : 126

fish_XP_017569575.1 : R-------------------------------------------------------EVQRQ--TDTTH----------------------------------------------------- : 88

fish_XP_018413083.1 : K--------------------------------------------------QGP---ANPDRKGVDGRK------------------------DP-------------------------- : 97

fish_XP_018429299.1 : --------------------------------------------------------REQKQSEDNSSRPPPLNE----------------------------------------------- : 115

fish_XP_018521334.1 : ------------------------------------------------------------------------------------------------------------------------- : -

fish_XP_018526467.1 : K-------------------------------------------------------KSQEQ--KKPTS----------------------------------------------------- : 88

fish_XP_018541041.1 : L------------------------------------------------------------------------------------------------------------------------ : 196

fish_XP_018558293.1 : ------------------------------------------------------------------------------------------------------------------------- : -

fish_XP_018595329.1 : H----------------------------------------------------------TNIQKEHVVPISEE------------------------------------------------ : 125

fish_XP_018611696.1 : Q--------------------------------------------------EQKPPKVQKK--QQSGP----------------------------------------------------- : 96

fish_XP_018956725.1 : E----------------------------------------------------NKDSISTDQTQIPKN----------------------------------------------------- : 118

frog_XP_018091031.1 : L--------------------------------------------------IARGKASVQTPTDSSSNLKLTTTIPTDVVQVPSTLASS-------------------------------- : 135

Hemichordata_XP_006825609.1 : S----------------------------------------------DKDTKSAINKAQPQTFNDVKTKEKQFTYAEKHHDPDVLQCIELLNRQLELDLYKKADVSEYYPCTLQLISGVFG : 450

lance_EEN48978.1 : ------------------------------------------------------------------------------------------------------------------------- : -

lance_EEN55742.1 : ----------------------------------------------------------ADDGTTNCKS----------------------------------------------------- : 70

mammal_XP_001378512.1 : Q----------------------------------------------------------TSEEQKDKQT---------------------------------------------------- : 94

mammal_XP_003771993.1 : Q----------------------------------------------------------TLGEKDGQKT---------------------------------------------------- : 92

mammal_XP_003771994.1 : P--------------------------------------------------KNP---VPTSEKQDSSKI-----------------SRK----EK-------------------------- : 97

mammal_XP_003782717.1 : S--------------------------------------------------EDS---SQTNKK---GKG-----------------SKI----VP-------------------------- : 96

mammal_XP_003982796.1 : H----------------------------------------------------------PKKPQQTKK----------------------------------------------------- : 109

mammal_XP_004389713.1 : P--------------------------------------------------ESH---NQNSGQLDHTKT-----------------SKK----EQ-------------------------- : 100

mammal_XP_004582357.1 : --------------------------------------------------------------Q---KKG-----------------NKY----AH-------------------------- : 82

mammal_XP_004582358.1 : P--------------------------------------------------EGD---KQDSEKSDRTKS-----------------SKK----EQ-------------------------- : 100

mammal_XP_004622892.1 : P--------------------------------------------------ESN---SPNSRPLDSTNV-----------------SKK----KS-------------------------- : 100

mammal_XP_004646313.1 : P--------------------------------------------------ESD---NPDSRQFDDTES-----------------SKK----RR-------------------------- : 100

mammal_XP_004702866.1 : S--------------------------------------------------ESH---NQESGQFDHTKP-----------------SKK----EH-------------------------- : 100

mammal_XP_005340592.1 : P--------------------------------------------------KND---NQDSAQSDLTKP-----------------SKK----EQ-------------------------- : 100

mammal_XP_005388084.1 : P--------------------------------------------------ESD---NPDSRQSDNTKS-----------------SKK----EH-------------------------- : 100

mammal_XP_006163552.1 : P--------------------------------------------------ESD---NQDSGQLDYTKP-----------------SIK----EH-------------------------- : 100

mammal_XP_006779273.1 : Q----------------------------------------------------------KKPQQTQKE----------------------------------------------------- : 109

mammal_XP_006834357.1 : L--------------------------------------------------ENH---NQDSGQFDQTKK-----------------SKK----KQ-------------------------- : 100

mammal_XP_006891533.1 : F--------------------------------------------------ESD---NHDSGKFDPTTT-----------------PKK----EH-------------------------- : 100

mammal_XP_007522555.1 : S--------------------------------------------------KNS---NQNSGQLDHAEP-----------------SKK----EH-------------------------- : 100

mammal_XP_007938002.1 : P--------------------------------------------------ESH---NEDSGQLDHPKS-----------------SKK----RY-------------------------- : 100

mammal_XP_008062458.1 : F--------------------------------------------------KDS---VQKPRR---KQG----------------GTMN----SP-------------------------- : 97

mammal_XP_008259950.1 : P--------------------------------------------------ESD---NQDSGKLDNIKP-----------------SKK----KQ-------------------------- : 100

mammal_XP_008524371.1 : P----------------------------------------------------------ESNNQDSGQLDHTKSSKEEH------------------------------------------ : 100

mammal_XP_008820771.1 : P--------------------------------------------------ESG---NQDSRLLDNEKS-----------------STK----E--------------------------- : 99

mammal_XP_010601767.1 : P--------------------------------------------------ESD---NPDSKQLVNTKS-----------------TEK----EH-------------------------- : 100

mammal_XP_011853657.1 : P--------------------------------------------------ESD---NHDPGQLDHSKP-----------------SKR----EH-------------------------- : 100

mammal_XP_012791283.1 : S--------------------------------------------------ENS---IQTREK---KTN-----------------NKN----IP-------------------------- : 96

mammal_XP_012881542.1 : L--------------------------------------------------ESN---NPNSRQFNDTKA-----------------SKK----EL-------------------------- : 100

mammal_XP_012934202.1 : S--------------------------------------------------EDP---IQTSKR---EKS-----------------SKN----IP-------------------------- : 102

mammal_XP_013013639.1 : P--------------------------------------------------ESD---NPNSRQVHDTKS-----------------SKK----EH-------------------------- : 100

mammal_XP_016016798.1 : H----------------------------------------------------------AKKPQQTKK----------------------------------------------------- : 109

mammal_XP_016281899.1 : P----------------------------------------------------------EKPVQASGKQER-------------------------------------------------- : 89

mammal_XP_017199853.1 : S------------------------------------------------------------------------------------------------------------------------ : 79

mammal_XP_017508280.1 : L--------------------------------------------------DSN---HQGLGQLDHTKP-----------------SKK----EH-------------------------- : 109

mollusk_EKC42356.1 : ------------------------------------------------------------------------------------------------------------------------- : -

mollusk_XP_011436570.1 : T----------------------------------------------------------SDSDDDSNTDNDSVSVKGVLIPANDELN---------------------------------- : 216

mollusk_XP_011440496.1 : ------------------------------------------------------------------------------------------------------------------------- : -

mollusk_XP_013061423.1 : R----------------------------------------------------------KRTAITCFVNIGPIF----------------------------------------------- : 191

mollusk_XP_013061425.1 : -----------------------------------------------------------HKTDSYPAF----------------------------------------------------- : 98

Musmu_NP_034286.2 : P--------------------------------------------------ESH---NQDSRELNDKKL-----------------STK----E--------------------------- : 98

rept_XP_008110733.2 : A--------------------------------------------------KRS---TMVSRQEHSTEI-----------------FDGKLSNES-------------------------- : 123

rept_XP_008112638.1 : H------------------------------------------------------------------------------------------------------------------------ : 90

rept_XP_015264172.1 : G--------------------------------------------------EGT---SKVPGKEPSIET-----------------LSANSFMEN-------------------------- : 133

shark_XP_007886940.1 : ------------------------------------------------------------------------------------------------------------------------- : -

Strpu_XP_003727489.1 : ------------------------------------------------------------------------------------------------------------------------- : -

Strpu_XP_011667260.1 : K----------------------------------------------------------VSAMESVNTPSPVTVE---------------------------------------------- : 98

turtle_XP_006139744.1 : V--------------------------------------------------QSR---AQLVRQEDSEKT-----------------LDKKYLTEK-------------------------- : 114

turtle_XP_007060369.1 : V--------------------------------------------------EST---AQTVRQEGRKKT-----------------FDEKYLMEK-------------------------- : 114

turtle_XP_014430084.1 : S----------------------------------------------------------HPGSTTHAQKKKVKAKNSNIPAMTNV------------------------------------ : 122

Xenla_OCT63184.1 : ------------------------------------------------------------------------------------------------------------------------- : -

Xenla_XP_018122800.1 : -----------------------------------------------------P---CIGNKTASDSSM------------------------KS-------------------------- : 92

Xentr_XP_002943568.3 : L----------------------------------------------------------APQIPLAEPCSTPGPPLVPQTPLAEPCSTPRPPL---------------------------- : 161

Xentr_XP_012813408.1 : ----------------------------------------------------------TTAEQKKLLS----------------------------------------------------- : 87

* 620 * 640 * 660 * 680 * 700 * 720

Homsa_NP_060124.2 : ---------------------------------------------------KDQTVSQKERRETSKQKQK--------------------------------------------------- : 115

Homsa_NP_689916.2 : ---------------------------------------------------QKNPKHTKKEEENSMSSNI--------------------------------------------------- : 119

fish_XP_015241820.1 : ------------------------------------------------------------------------------------------------------------------------- : -

fish_XP_018543893.1 : ------------------------------------------------------------------------------------------------------------------------- : -

fish_CDQ83481.1 : ------------------------------------------------------------------------------------------------------------------------- : -

fish_CDQ89582.1 : ------------------------------------------------------------------------------------------------------------------------- : -

fish_XP_005809248.1 : ------------------------------------------------------------------------------------------------------------------------- : -

fish_XP_007231320.1 : ------------------------------------------------------------------------------------------------------------------------- : -

fish_XP_007254667.1 : ------------------------------------------------------------------------------------------------------------------------- : -

fish_XP_008277954.1 : ------------------------------------------------------------------------------------------------------------------------- : -

fish_XP_010746900.1 : ------------------------------------------------------------------------------------------------------------------------- : -

fish_XP_012685053.1 : ------------------------------------------------------------------------------------------------------------------------- : -

fish_XP_013123332.1 : ------------------------------------------------------------------------------------------------------------------------- : -

fish_XP_014011897.1 : ------------------------------------------------------------------------------------------------------------------------- : -

fish_XP_014011904.1 : ------------------------------------------------------------------------------------------------------------------------- : -

fish_XP_014264868.1 : ------------------------------------------------------------------------------------------------------------------------- : -

fish_XP_014264875.1 : ------------------------------------------------------------------------------------------------------------------------- : -

fish_XP_014854842.1 : ------------------------------------------------------------------------------------------------------------------------- : -

fish_XP_017578465.1 : ------------------------------------------------------------------------------------------------------------------------- : -

fish_XP_012713132.1 : ------------------------------------------------------------------------------------------------------------------------- : -

fish_XP_010787190.1 : ------------------------------------------------------------------------------------------------------------------------- : -

fish_XP_017319342.1 : ----------------------------------------------------EDQMKQDAE------------------------------------------------------------ : 101

Alligator_KYO30992.1 : ----------------------------------------------------VTQLEDASNSQVTHPSGA--------------------------------------------------- : 99

Alligator_XP_006025954.1 : ---------------------------------------------------KDEKIMEKNR--KGDSKPV--------------------------------------------------- : 132

amphibia_XP_018413082.1 : ---------------------------------------------------KGTETKQNKSKSKQ-------------------------------------------------------- : 111

bact_AGA70736.1 : ------------------------------------------------------------------------------------------------------------------------- : -

bact_CUJ86040.1 : ---------------------------------------------------WDGVAIHSAEDTPNSLFRS--------------------------------------------------- : 77

bact_ESW58583.1 : ------------------------------------------------------------------------------------------------------------------------- : -

bact_GAC67715.1 : ---------------------------------------------------MLRSATSDDLSVELYDLAGH-------------------------------------------------- : 105

bact_KMO66808.1 : ----------------------------------------------------ERFRTLGAYTLGHTPEIP--------------------------------------------------- : 98

bact_KUO79116.1 : ---------------------------------------------------YDSSDNEATKSSRAEQHAN--------------------------------------------------- : 86

bact_KYG06363.1 : ----------------------------------------VSTEYADEYARHAQAVDIGAFSKSVPPLVG--------------------------------------------------- : 90

bact_ODA70488.1 : ---------------------------------------------------GYGRTRVPQLVQSLQESG---------------------------------------------------- : 112

bact_OGP49286.1 : -----------------------------------------------------KTTLLSPQRFFII------------------------------------------------------- : 93

bact_SCF05533.1 : ---------------------------------------------------RDEQFAERHLPRLLEIYKQ--------------------------------------------------- : 104

bact_SCX17622.1 : ----------------------------------------------------ANQVNYRENSVLVDSFNA--------------------------------------------------- : 32

bact_SFJ80976.1 : ---------------------------------------------------PDFAEHHIPRLVRLHQRTA--------------------------------------------------- : 106

bact_WP_006128369.1 : ----------------------------------------------------AHNLLREAAPDFAENHIP--------------------------------------------------- : 124

bact_WP_006365791.1 : ------------------------------------------------------------------------------------------------------------------------- : -

bact_WP_006849212.1 : ------------------------------------------------------------------------------------------------------------------------- : -

bact_WP_007897046.1 : ------------------------------------------------------------------------------------------------------------------------- : -

bact_WP_030194308.1 : -----------------------------------------------AFAGGLGEFGRTRLPQLVRSLQV--------------------------------------------------- : 110

bact_WP_030198971.1 : ---------------------------------------------------DRQNEAHRLFVERLSPFAS--------------------------------------------------- : 98

bact_WP_030898614.1 : --------------------------------------------------APEGFAERHLVRQPVEPVSV--------------------------------------------------- : 114

bact_WP_041219555.1 : ------------------------------------------------------------------------------------------------------------------------- : -

bact_WP_050505446.1 : ----------------------------------------------------SPRSQSTSLLRGAQAISR--------------------------------------------------- : 100

bact_WP_055538636.1 : ---------------------------------------------------DRQNEAHRLFTERLTPFAD--------------------------------------------------- : 98

bact_WP_062134708.1 : ------------------------------------------------------------------------------------------------------------------------- : -

bact_WP_062799339.1 : ---------------------------------------------------VDGRSLSSALVEVGMGL----------------------------------------------------- : 61

bact_WP_066463897.1 : ------------------------------------------------------------------------------------------------------------------------- : -

bird_XP_002194798.1 : ----------------------------------------------------KKPNRTDAQKGPARDSNA--------------------------------------------------- : 104

bird_XP_005010984.1 : ---------------------------------------------------DDEEIVKNECKKKYRSFNS--------------------------------------------------- : 124

bird_XP_005505023.1 : ---------------------------------------------------EDGDIAKTECKKQHGSFNS--------------------------------------------------- : 125

bird_XP_008492486.1 : ---------------------------------------------------EDREIEKKESKKKHDLFNS--------------------------------------------------- : 125

bird_XP_008628214.1 : ---------------------------------------------------KDGEMAKKECKKNCESLNS--------------------------------------------------- : 125

bird_XP_009068034.1 : ----------------------------------------------------KTPDRTDVQTDPVGASNS--------------------------------------------------- : 102

bird_XP_009080343.1 : ---------------------------------------------------KDGGLGKEECKKKCESFNS--------------------------------------------------- : 125

bird_XP_009486154.1 : ---------------------------------------------------EDGEIAKKECKKKYGSFNSSI------------------------------------------------- : 127

bird_XP_009640122.1 : ---------------------------------------------------EDGEIAKKECKKKDESFNSSM------------------------------------------------- : 125

bird_XP_009672623.1 : ----------------------------------------------------------XXXXXXYCSFNA--------------------------------------------------- : 113

bird_XP_009695665.1 : ----------------------------------------------------KAPNRTDAQTDPVRPRNA--------------------------------------------------- : 250

bird_XP_009886214.1 : ---------------------------------------------------EDGEIAKKECKKTHGSLTSST------------------------------------------------- : 127

bird_XP_009934438.1 : ---------------------------------------------------EDGETAKKD-----GPFNSSI------------------------------------------------- : 121

bird_XP_009999877.1 : ---------------------------------------------------VDGEIAKKDCKKKYGSFNS--------------------------------------------------- : 124

bird_XP_010119033.1 : ---------------------------------------------------EDGEIAKKKWKNTCGSFNS--------------------------------------------------- : 123

bird_XP_010144931.1 : ---------------------------------------------------EDGE------------FNS--------------------------------------------------- : 113

bird_XP_010222910.1 : ---------------------------------------------------EDKETAKQNSNK---------------------------------------------------------- : 118

bird_XP_013160560.1 : ---------------------------------------------------EDGEIAKKECEKEYGSSNC--------------------------------------------------- : 129

bird_XP_014795808.1 : ---------------------------------------------------EDGEIAKKLCKKKSGSFTSGI------------------------------------------------- : 127

bird_XP_015156587.1 : ---------------------------------------------------KEDGKIAKECKKYGSLNSN--------------------------------------------------- : 119

bird_XP_015709021.1 : ---------------------------------------------------VDGEIAKKEC-KEYRSSNS--------------------------------------------------- : 130

brachiop_XP_013415988.1 : ---------------------------------------------------DTGQQMEEESASIPMVWSPPEN------------------------------------------------ : 176

Cioin_XP_018668123.1 : ------------------------------------------------------------------------------------------------------------------------- : -

Cnidaria_EDO35430.1 : ---------------------------------------------------ILDEDIIDELLSQESPPMRILL------------------------------------------------ : 141

Cnidaria_KXJ09690.1 : ---------------------------------------------------ESKQVLSEEKESKLKIPKDSEKLIKSDSGSEK-------------------------------------- : 171

Cnidaria_KXJ11435.1 : ---------------------------------------------------VRQVLLAEKYSESKVPEEG--------------------------------------------------- : 158

Cnidaria_KXJ17955.1 : ---------------------------------------------------PDVLILYEEMSQQLQIKDDTHVRGVPFP------------------------------------------ : 257

Cnidaria_KXJ23466.1 : ---------------------------------------------------AASLIAQEKFEDALKDCRN--------------------------------------------------- : 176

Cnidaria_XP_015751626.1 : KLLLPQLHMYADEMKQFCWWLAECHLRKGHAEEKNLEAALRQCEEADRLVPDDVNVLYTKMKVLERMNRHKECYAVCNKLIQSNFKESEAVKKTLERLLPKIVMEDEALATKEQGKPELPG : 386

Cnidaria_XP_015751629.1 : KLLLPQLHMYADEMKQFCWWLAECHLRKGHAEEKNLEAALRQCEEADRLVPDDVNVLYTKMKVLERMNRHKECYAVCNKLIQSNFKESEAVKKTLERLLPKIVMEDEALATKEQGKPELPG : 386

Cnidaria_XP_015758975.1 : ---------------------------------------------------SERDPLEAETESYLSQVQD--------------------------------------------------- : 374

Cnidaria_XP_015766106.1 : ----------------------------------------------------KGTMHLKKDEEAV-------------------------------------------------------- : 50

Cnidaria_XP_015766118.1 : ----------------------------------------------------DEDAHLVEGRSSTEKPSI--------------------------------------------------- : 112

Cnidaria_XP_015766124.1 : ---------------------------------------------------NEIESVEEQIKQEDDQEEP--------------------------------------------------- : 97

coelacanth_XP_006000513.2 : ---------------------------------------------------RDGYQKRKAN------------------------------------------------------------ : 59

fish_CDQ87765.1 : ---------------------------------------------------DSDSVKSESVAPSLSSSWTSV------------------------------------------------- : 344

fish_CDQ92295.1 : ----------------------------------------------------RDTSHVESSAVFTQESKD--------------------------------------------------- : 112

fish_KKF13953.1 : -------------------------------------------------------IEVEQKNKNQKSVQH--------------------------------------------------- : 100

fish_XP_004085798.2 : ---------------------------------------------------PHSDYEVKKAQDMSGRLNE--------------------------------------------------- : 137

fish_XP_004552746.3 : ----------------------------------------------------KECKHQSEFPKYVEKWTK--------------------------------------------------- : 95

fish_XP_004558390.1 : ----------------------------------------------------SKRTEFEEK-RSQKLVQS--------------------------------------------------- : 114

fish_XP_004576250.1 : ----------------------------------------------------KTVSSDSGIQSESSVFVE--------------------------------------------------- : 174

fish_XP_005161888.1 : ----------------------------------------------------GKNENGVSK------------------------------------------------------------ : 97

fish_XP_005478813.2 : ---------------------------------------------------KWTKEQVSQWLVQHLNIYS--------------------------------------------------- : 110

fish_XP_005806405.2 : ----------------------------------------------------SNTKHDKKVNVFV-------------------------------------------------------- : 114

fish_XP_006625674.1 : ---------------------------------------------------PSQEQNNLEEHQKAKQPSQNLKELQNAKQPSEKQKNLEELQNAKQPSQEQNNLQKHQKAKRRKAGKQKNQ : 259

fish_XP_006635547.1 : ----------------------------------------------------GLWNPNRNLKGAARPASR--------------------------------------------------- : 174

fish_XP_006635548.1 : ----------------------------------------------------FPERSET--QRVSADHQK--------------------------------------------------- : 103

fish_XP_006791552.1 : ----------------------------------------------------SKRTEFEEK-KSKKSVQC--------------------------------------------------- : 107

fish_XP_006791631.1 : ----------------------------------------------------GKSRDGKKETVEDVGQET--------------------------------------------------- : 111

fish_XP_007241837.1 : ----------------------------------------------------HERTVQKSGQKRASSINN--------------------------------------------------- : 106

fish_XP_007242299.1 : ---------------------------------------------------PDTSTNSENIGSTNEEPST--------------------------------------------------- : 173

fish_XP_007557414.1 : ----------------------------------------------------GKKSELERK----KSVEG--------------------------------------------------- : 98

fish_XP_008284284.1 : ----------------------------------------------------------KIMKEEPTSSAN--------------------------------------------------- : 95

fish_XP_008284762.1 : --------------------------------------------------------------------EP--------------------------------------------------- : 79

fish_XP_008285761.1 : ----------------------------------------------------GKTTELEQK-SSQKPVPA--------------------------------------------------- : 105

fish_XP_008336142.1 : ----------------------------------------------------EGTNYQAQFPAYVAQWSK--------------------------------------------------- : 91

fish_XP_008417602.1 : ---------------------------------------------------PERELNLRDNAEMSQKLDE--------------------------------------------------- : 184

fish_XP_009510531.1 : ---------------------------------------------------EDGEIAKKECKKKYQSLYSSI------------------------------------------------- : 126

fish_XP_010752845.1 : ----------------------------------------------------EGSVLESEFPAYVENWTK--------------------------------------------------- : 94

fish_XP_010755274.1 : ---------------------------------------------------RDGKVKFKKEEPTSS------------------------------------------------------- : 93

fish_XP_010767524.1 : ----------------------------------------------------KGPRPEPQFPAYVENWTK--------------------------------------------------- : 93

fish_XP_010781233.1 : -------------------------------------------------------KFKKEEPTNATN------------------------------------------------------ : 115

fish_XP_010791966.1 : ----------------------------------------------------GKNTEDKQN-IHQKAVQ---------------------------------------------------- : 47

fish_XP_012687177.1 : ----------------------------------------------------DGPSTTKHLP----------------------------------------------------------- : 76

fish_XP_012708385.1 : ----------------------------------------------------DQTNKKRNLGNKSDLPK---------------------------------------------------- : 105

fish_XP_012713253.1 : -------------------------------------------------------NLKSKTPTSSSN------------------------------------------------------ : 93

fish_XP_012727798.1 : ----------------------------------------------------GKMREAK--NVVV-------------------------------------------------------- : 98

fish_XP_012990558.1 : ----------------------------------------------------SGSQPKSVFPTYINRWTK--------------------------------------------------- : 90

fish_XP_013126906.1 : -------------------------------------------------------KFKKEEPVGSRN------------------------------------------------------ : 95

fish_XP_013796981.1 : ---------------------------------------------------EDKEIAKKECNKKYRSFNP--------------------------------------------------- : 125

fish_XP_014005049.1 : ------------------------------------------------------------------------------------------------------------------------- : -

fish_XP_014049585.1 : ----------------------------------------------------SNNSEKGSKKKQGSAPQT--------------------------------------------------- : 107

fish_XP_014325953.1 : ----------------------------------------------------EASAHRSECPAYVEAWT---------------------------------------------------- : 88

fish_XP_015195770.1 : ------------------------------------------------------------------------------------------------------------------------- : -

fish_XP_015197335.1 : ---------------------------------------------------EEDQDASKTIQTSMQTASPHMFQQCS-------------------------------------------- : 113

fish_XP_015232478.1 : ------------------------------------------------------------------------------------------------------------------------- : -

fish_XP_015252023.1 : ------------------------------------------------------------------------------------------------------------------------- : -

fish_XP_015264177.1 : ---------------------------------------------------KQPGLLTEERKGVAEETKC--------------------------------------------------- : 125

fish_XP_015801149.1 : ----------------------------------------------------DKNSESEQK-NIQRSTQS--------------------------------------------------- : 105

fish_XP_015810486.1 : ----------------------------------------------------RDEVMTLKREEPMSSPN---------------------------------------------------- : 93

fish_XP_016115105.1 : ----------------------------------------------------QNTQQQNDMVTQSCSL----------------------------------------------------- : 105

fish_XP_016117315.1 : ---------------------------------------------------SKKSQKLNVNKDSMDMITS--------------------------------------------------- : 150

fish_XP_016395637.1 : ----------------------------------------------------GKNDHEASA------------------------------------------------------------ : 98

fish_XP_017207716.1 : ---------------------------------------------------SKGKSNQKMNSNKCRTTNT--------------------------------------------------- : 137

fish_XP_017277259.1 : ----------------------------------------------------SKKSEDKKDNVFVLQEEP--------------------------------------------------- : 112

fish_XP_017277301.1 : ----------------------------------------------------AKSSESEPK-NNQRSAQG--------------------------------------------------- : 105

fish_XP_017325460.1 : ------------------------------------------------------------------------------------------------------------------------- : -

fish_XP_017346953.1 : ----------------------------------------------------ERTAE-----NDDQGATS--------------------------------------------------- : 101

fish_XP_017550536.1 : ----------------------------------------------------QKALSDTEADTSTKTVRP--------------------------------------------------- : 104

fish_XP_017550824.1 : ----------------------------------------------------------KRKAQQSVSHGN--------------------------------------------------- : 99

fish_XP_017554653.1 : ---------------------------------------------------QSRQKAKKSNQECLPNPDICKNLEAKNPNKASSSNITEAKVEKTLCDMAASKPTEISTVQ---------- : 186

fish_XP_017569575.1 : ----------------------------------------------------EKTVQKRDDKNNDQKPAS--------------------------------------------------- : 106

fish_XP_018413083.1 : ---------------------------------------------------TNKPTTNKNEKQKRADA----------------------------------------------------- : 114

fish_XP_018429299.1 : ---------------------------------------------------PSRSNPEDAQHSCKTLTKA--------------------------------------------------- : 134

fish_XP_018521334.1 : ----------------------------------------------------DGSQHESQFPAYVENWTK--------------------------------------------------- : 95

fish_XP_018526467.1 : ----------------------------------------------------CINNKLEQN-SDQKLVQC--------------------------------------------------- : 105

fish_XP_018541041.1 : ----------------------------------------------------WKSAEMQIVSGNMDESME--------------------------------------------------- : 214

fish_XP_018558293.1 : -------------------------------------------------------KLRKEGASSLTSSTN--------------------------------------------------- : 107

fish_XP_018595329.1 : ---------------------------------------------------SNTPLELNTVPTKHRDKHE--------------------------------------------------- : 144

fish_XP_018611696.1 : ----------------------------------------------------TRESEGNDVKEEVSHLDT--------------------------------------------------- : 114

fish_XP_018956725.1 : ---------------------------------------------------AKKSKRKSQKKENTDLKSA--------------------------------------------------- : 137

frog_XP_018091031.1 : ----------------------------------------------------SNQELIEKGKASVQPPTDSSSILKVTSTI-----------------------------------PTDVV : 169

Hemichordata_XP_006825609.1 : KAANLLERQFCFLTLKKGQKELTSQETDLLWKQIQLNVKQFIRLLPANKQVIFDVNIETKGEGVARASDKQQFLLRDKGLAACPMLRKNLKSITMHTQLVLLVDSKMLTPTTLRYGTYLDI : 571

lance_EEN48978.1 : ------------------------------------------------------------------------------------------------------------------------- : -

lance_EEN55742.1 : ----------------------------------------------------GNANGAEDTKASLSGTRT--------------------------------------------------- : 88

mammal_XP_001378512.1 : ---------------------------------------------------EVQWVTQEKNRKRRRHHTD--------------------------------------------------- : 113

mammal_XP_003771993.1 : ---------------------------------------------------EDQCVTQDKKRKGKRNQTD--------------------------------------------------- : 111

mammal_XP_003771994.1 : ---------------------------------------------------RKRGKVKTENTEEAILTTN--------------------------------------------------- : 116

mammal_XP_003782717.1 : ---------------------------------------------------TNQTLMQKESRE----KQK--------------------------------------------------- : 111

mammal_XP_003982796.1 : ---------------------------------------------------EDEKSELPNFNHDLREIRD--------------------------------------------------- : 128

mammal_XP_004389713.1 : ---------------------------------------------------RKKPQQTKKEE--LISSNI--------------------------------------------------- : 117

mammal_XP_004582357.1 : ---------------------------------------------------KKQELVQKENRDTSKQNQQ--------------------------------------------------- : 101

mammal_XP_004582358.1 : ---------------------------------------------------QKKPKQKRKEEEAPTPSSS--------------------------------------------------- : 119

mammal_XP_004622892.1 : ---------------------------------------------------KKNSQNTKEEK--LVSSNS--------------------------------------------------- : 117

mammal_XP_004646313.1 : ---------------------------------------------------KKKPKPTQKESQELMSSDI--------------------------------------------------- : 119

mammal_XP_004702866.1 : ---------------------------------------------------TKKSQETKKKK--SVSSDI--------------------------------------------------- : 117

mammal_XP_005340592.1 : ---------------------------------------------------QKKPKQTKKEEEK-MSSGI--------------------------------------------------- : 118

mammal_XP_005388084.1 : ---------------------------------------------------QKNPKLTKKEETELMSSHI--------------------------------------------------- : 119

mammal_XP_006163552.1 : ---------------------------------------------------KKVPKQTKKEEEKSISSNM--------------------------------------------------- : 119

mammal_XP_006779273.1 : ----------------------------------------------------EEQSMPSNIDHNLREIRD--------------------------------------------------- : 127

mammal_XP_006834357.1 : ---------------------------------------------------KKTPEETKKEE--PISSNI--------------------------------------------------- : 117

mammal_XP_006891533.1 : ---------------------------------------------------QKKPKQSKKEK--PISSKT--------------------------------------------------- : 117

mammal_XP_007522555.1 : ---------------------------------------------------TKKTQTTE-----NTLSNN--------------------------------------------------- : 114

mammal_XP_007938002.1 : ---------------------------------------------------QDKPQQTEKEK--SVSSDI--------------------------------------------------- : 117

mammal_XP_008062458.1 : ---------------------------------------------------EEPTLMQKESSELSKQKQK--------------------------------------------------- : 116

mammal_XP_008259950.1 : ---------------------------------------------------QKKPKQKSKEEGTSMSLNI--------------------------------------------------- : 119

mammal_XP_008524371.1 : ---------------------------------------------------KEKPQQTKKEKEKSTSSNIDHNL----------------------------------------------- : 123

mammal_XP_008820771.1 : ---------------------------------------------------QKKPKQIKNQEEKPMSLNT--------------------------------------------------- : 118

mammal_XP_010601767.1 : ---------------------------------------------------QKKPKQTKKEEKESMSSNT--------------------------------------------------- : 119

mammal_XP_011853657.1 : ---------------------------------------------------QKDPKQTKKEEENSMSSNI--------------------------------------------------- : 119

mammal_XP_012791283.1 : ---------------------------------------------------EVQTLSQSENGEKSKKKQK--------------------------------------------------- : 115

mammal_XP_012881542.1 : ---------------------------------------------------QKKSKQTKNEE-KSVPSNI--------------------------------------------------- : 118

mammal_XP_012934202.1 : ---------------------------------------------------TKQSLMQKEGRETSKQKQK--------------------------------------------------- : 121

mammal_XP_013013639.1 : ---------------------------------------------------QKKPNL----EKELMPSSI--------------------------------------------------- : 115

mammal_XP_016016798.1 : ---------------------------------------------------DEQNSVSSNIAHDFSETTD--------------------------------------------------- : 128

mammal_XP_016281899.1 : ---------------------------------------------------LKTPGKGKGTKVKLETENT--------------------------------------------------- : 108

mammal_XP_017199853.1 : ----------------------------------------------------KDPIRTSEQKKGSKNVSN--------------------------------------------------- : 97

mammal_XP_017508280.1 : ---------------------------------------------------QKKSQQTKKEEEKAMSSNI--------------------------------------------------- : 128

mollusk_EKC42356.1 : --------------------------------------------------------SSKQCELYCQNCDI--------------------------------------------------- : 80

mollusk_XP_011436570.1 : ---------------------------------------------------PSEDKPSRKFLNDLETSEMKICLASYFEEHGLEERFLEVFDLEKIDGKVFVDCDMDDFNRLF-------- : 278

mollusk_XP_011440496.1 : ----------------------------------------------------DLQQRVENQINLLQPGKH--------------------------------------------------- : 112

mollusk_XP_013061423.1 : ---------------------------------------------------ESVQSPPEVKPESHEEPKE--------------------------------------------------- : 210

mollusk_XP_013061425.1 : ------------------------------------------------------------------------------------------------------------------------- : -

Musmu_NP_034286.2 : ---------------------------------------------------QQ--TKTKNEEENSVSSNS--------------------------------------------------- : 115

rept_XP_008110733.2 : ---------------------------------------------------KDGRLIEANNEMKSDAASK--------------------------------------------------- : 142

rept_XP_008112638.1 : ------------------------------------------------NKTHSESKYPKESDSSTEEKKG--------------------------------------------------- : 112

rept_XP_015264172.1 : ---------------------------------------------------RDGGKMEESARGKSKSTNA--------------------------------------------------- : 152

shark_XP_007886940.1 : ------------------------------------------------------------------------------------------------------------------------- : -

Strpu_XP_003727489.1 : ---------------------------------------------------QNGELMYHELESLFAQLGY--------------------------------------------------- : 63

Strpu_XP_011667260.1 : ---------------------------------------------------PAPPPATSADADEPVLQGKSMQ------------------------------------------------ : 120

turtle_XP_006139744.1 : ---------------------------------------------------EDGQSAKQDEKDKGKSKTG--------------------------------------------------- : 133

turtle_XP_007060369.1 : ---------------------------------------------------KDGGSAKQDQKNKGKSKSA--------------------------------------------------- : 133

turtle_XP_014430084.1 : ---------------------------------------------------PAEKRESSTKTNQITGPKN--------------------------------------------------- : 141

Xenla_OCT63184.1 : ------------------------------------------------------------------------------------------------------------------------- : -

Xenla_XP_018122800.1 : ---------------------------------------------------EEKKKTKNQKKEEDIDL----------------------------------------------------- : 109

Xentr_XP_002943568.3 : ---------------------------------------------------VPQTPLAEPCSTPRPPLVPQTPLADPCSTPGPPLVPQTPLADPCSTPGPPLVPQTPLADPCSTPGPPLVP : 231

Xentr_XP_012813408.1 : ----------------------------------------------------GNNDKHPSGKDKETVERP--------------------------------------------------- : 105

* 740 * 760 * 780 * 800 * 820 * 840

Homsa_NP_060124.2 : --------------------------GKEN------P------------------------------------------------------------------------------------ : 120

Homsa_NP_689916.2 : --------------------------DYDP------R------------------------------------------------------------------------------------ : 124

fish_XP_015241820.1 : ------------------------------------------------------------------------------------------------------------------------- : -

fish_XP_018543893.1 : ------------------------------------------------------------------------------------------------------------------------- : -

fish_CDQ83481.1 : ------------------------------------------------------------------------------------------------------------------------- : -

fish_CDQ89582.1 : ------------------------------------------------------------------------------------------------------------------------- : -

fish_XP_005809248.1 : ------------------------------------------------------------------------------------------------------------------------- : -

fish_XP_007231320.1 : ------------------------------------------------------------------------------------------------------------------------- : -

fish_XP_007254667.1 : ------------------------------------------------------------------------------------------------------------------------- : -

fish_XP_008277954.1 : ------------------------------------------------------------------------------------------------------------------------- : -

fish_XP_010746900.1 : ------------------------------------------------------------------------------------------------------------------------- : -

fish_XP_012685053.1 : ------------------------------------------------------------------------------------------------------------------------- : -

fish_XP_013123332.1 : ------------------------------------------------------------------------------------------------------------------------- : -

fish_XP_014011897.1 : ------------------------------------------------------------------------------------------------------------------------- : -

fish_XP_014011904.1 : ------------------------------------------------------------------------------------------------------------------------- : -

fish_XP_014264868.1 : ------------------------------------------------------------------------------------------------------------------------- : -

fish_XP_014264875.1 : ------------------------------------------------------------------------------------------------------------------------- : -

fish_XP_014854842.1 : ------------------------------------------------------------------------------------------------------------------------- : -

fish_XP_017578465.1 : ------------------------------------------------------------------------------------------------------------------------- : -

fish_XP_012713132.1 : ------------------------------------------------------------------------------------------------------------------------- : -

fish_XP_010787190.1 : ------------------------------------------------------------------------------------------------------------------------- : -

fish_XP_017319342.1 : ------------------------------------------------------------------------------------------------------------------------- : -

Alligator_KYO30992.1 : ------------------------------------------------------------------------------------------------------------------------- : -

Alligator_XP_006025954.1 : --------------------------DSSTTQECKIS------------------------------------------------------------------------------------ : 143

amphibia_XP_018413082.1 : ------------------------------------S------------------------------------------------------------------------------------ : 112

bact_AGA70736.1 : ------------------------------------------------------------------------------------------------------------------------- : -

bact_CUJ86040.1 : ------------------------------------------------------------------------------------------------------------------------- : -

bact_ESW58583.1 : ------------------------------------------------------------------------------------------------------------------------- : -

bact_GAC67715.1 : ------------------------------------------------------------------------------------------------------------------------- : -

bact_KMO66808.1 : ------------------------------------------------------------------------------------------------------------------------- : -

bact_KUO79116.1 : ------------------------------------------------------------------------------------------------------------------------- : -

bact_KYG06363.1 : ------------------------------------------------------------------------------------------------------------------------- : -

bact_ODA70488.1 : ------------------------------------------------------------------------------------------------------------------------- : -

bact_OGP49286.1 : ------------------------------------------------------------------------------------------------------------------------- : -

bact_SCF05533.1 : ------------------------------------------------------------------------------------------------------------------------- : -

bact_SCX17622.1 : ------------------------------------------------------------------------------------------------------------------------- : -

bact_SFJ80976.1 : ------------------------------------------------------------------------------------------------------------------------- : -

bact_WP_006128369.1 : ------------------------------------------------------------------------------------------------------------------------- : -

bact_WP_006365791.1 : ------------------------------------------------------------------------------------------------------------------------- : -

bact_WP_006849212.1 : ------------------------------------------------------------------------------------------------------------------------- : -

bact_WP_007897046.1 : ------------------------------------------------------------------------------------------------------------------------- : -

bact_WP_030194308.1 : ------------------------------------------------------------------------------------------------------------------------- : -

bact_WP_030198971.1 : ------------------------------------------------------------------------------------------------------------------------- : -

bact_WP_030898614.1 : ------------------------------------------------------------------------------------------------------------------------- : -

bact_WP_041219555.1 : ------------------------------------------------------------------------------------------------------------------------- : -

bact_WP_050505446.1 : ------------------------------------------------------------------------------------------------------------------------- : -

bact_WP_055538636.1 : ------------------------------------------------------------------------------------------------------------------------- : -

bact_WP_062134708.1 : ------------------------------------------------------------------------------------------------------------------------- : -

bact_WP_062799339.1 : ------------------------------------------------------------------------------------------------------------------------- : -

bact_WP_066463897.1 : ------------------------------------------------------------------------------------------------------------------------- : -

bird_XP_002194798.1 : -----------------------------PAQGTASG------------------------------------------------------------------------------------ : 112

bird_XP_005010984.1 : ------------------------------------S------------------------------------------------------------------------------------ : 125

bird_XP_005505023.1 : ------------------------------------S------------------------------------------------------------------------------------ : 126

bird_XP_008492486.1 : ------------------------------------S------------------------------------------------------------------------------------ : 126

bird_XP_008628214.1 : ------------------------------------S------------------------------------------------------------------------------------ : 126

bird_XP_009068034.1 : -----------------------------LAQSTAGG------------------------------------------------------------------------------------ : 110

bird_XP_009080343.1 : ------------------------------------S------------------------------------------------------------------------------------ : 126

bird_XP_009486154.1 : ------------------------------------------------------------------------------------------------------------------------- : -

bird_XP_009640122.1 : ------------------------------------------------------------------------------------------------------------------------- : -

bird_XP_009672623.1 : ------------------------------------S------------------------------------------------------------------------------------ : 114

bird_XP_009695665.1 : -----------------------------PAQKTSRG------------------------------------------------------------------------------------ : 258

bird_XP_009886214.1 : ------------------------------------------------------------------------------------------------------------------------- : -

bird_XP_009934438.1 : ------------------------------------------------------------------------------------------------------------------------- : -

bird_XP_009999877.1 : ------------------------------------N------------------------------------------------------------------------------------ : 125

bird_XP_010119033.1 : ------------------------------------S------------------------------------------------------------------------------------ : 124

bird_XP_010144931.1 : ------------------------------------N------------------------------------------------------------------------------------ : 114

bird_XP_010222910.1 : ------------------------------------------------------------------------------------------------------------------------- : -

bird_XP_013160560.1 : ------------------------------------G------------------------------------------------------------------------------------ : 130

bird_XP_014795808.1 : ------------------------------------------------------------------------------------------------------------------------- : -

bird_XP_015156587.1 : ------------------------------------------------------------------------------------------------------------------------- : -

bird_XP_015709021.1 : ------------------------------------S------------------------------------------------------------------------------------ : 131

brachiop_XP_013415988.1 : ------------------------------------------------------------------------------------------------------------------------- : -

Cioin_XP_018668123.1 : ------------------------------------------------------------------------------------------------------------------------- : -

Cnidaria_EDO35430.1 : ------------------------------------------------------------------------------------------------------------------------- : -

Cnidaria_KXJ09690.1 : ------------------------------------------------------------------------------------------------------------------------- : -

Cnidaria_KXJ11435.1 : ------------------------------------------------------------------------------------------------------------------------- : -

Cnidaria_KXJ17955.1 : ------------------------------------------------------------------------------------------------------------------------- : -

Cnidaria_KXJ23466.1 : ------------------------VLDQNPHDLKAKF------------------------------------------------------------------------------------ : 189

Cnidaria_XP_015751626.1 : SPEVALAKKSKKGRAKKGSNRQKEKQTNEGSLEGEQPPSVDDNVEPIYSNTTDTSLSLPVARDSVAEEGARTSLTSCEDVTQGRVRSSMTTRNRITPLSKSGLGVGLEGNDLLEKTRERDP : 507

Cnidaria_XP_015751629.1 : SPEVALAKDKDVLDKSQPSVSTSLNNISTDSIPGGKV------------------------------------------------------------------------------------ : 423

Cnidaria_XP_015758975.1 : ----------------------KDVLDKSQASGPIST------------------------------------------------------------------------------------ : 389

Cnidaria_XP_015766106.1 : ------------------------------------------------------------------------------------------------------------------------- : -

Cnidaria_XP_015766118.1 : ------------------------------------------------------------------------------------------------------------------------- : -

Cnidaria_XP_015766124.1 : ------------------------------------------------------------------------------------------------------------------------- : -

coelacanth_XP_006000513.2 : ------------------------------------E------------------------------------------------------------------------------------ : 60

fish_CDQ87765.1 : ------------------------------------------------------------------------------------------------------------------------- : -

fish_CDQ92295.1 : -----------------------------KVSRSSEK------------------------------------------------------------------------------------ : 120

fish_KKF13953.1 : ------------------------LATTSESPPTQTT------------------------------------------------------------------------------------ : 113

fish_XP_004085798.2 : ------------------------------------------------------------------------------------------------------------------------- : -

fish_XP_004552746.3 : ------------------------------------------------------------------------------------------------------------------------- : -

fish_XP_004558390.1 : ------------------------LSTVSDGPPAQTT------------------------------------------------------------------------------------ : 127

fish_XP_004576250.1 : ------------------------------------------------------------------------------------------------------------------------- : -

fish_XP_005161888.1 : ----------------------------------NKV------------------------------------------------------------------------------------ : 100

fish_XP_005478813.2 : ------------------------------------------------------------------------------------------------------------------------- : -

fish_XP_005806405.2 : --------------------------------SEGTT------------------------------------------------------------------------------------ : 119

fish_XP_006625674.1 : EKLQEVNQLSQDENTKHNTSQKVANQSTQNTSETVNE------------------------------------------------------------------------------------ : 296

fish_XP_006635547.1 : ------------------------FRSLARLWVRGSP------------------------------------------------------------------------------------ : 187

fish_XP_006635548.1 : -----------------------------KAGGKGPP------------------------------------------------------------------------------------ : 111

fish_XP_006791552.1 : ------------------------LRTVSDGSPAQTI------------------------------------------------------------------------------------ : 120

fish_XP_006791631.1 : -----------------------------QL-SPFHH------------------------------------------------------------------------------------ : 118

fish_XP_007241837.1 : ----------------------------------DST------------------------------------------------------------------------------------ : 109

fish_XP_007242299.1 : ------NTTTQTTESQAPKPLSNVDEGQPKERPTVMP------------------------------------------------------------------------------------ : 204

fish_XP_007557414.1 : ------------------------SQTTEEGS--EDP------------------------------------------------------------------------------------ : 109

fish_XP_008284284.1 : ------------------------------------------------------------------------------------------------------------------------- : -

fish_XP_008284762.1 : -----------------------------KLSSQEAS------------------------------------------------------------------------------------ : 87

fish_XP_008285761.1 : ---------------------------TSEDPPAQTP------------------------------------------------------------------------------------ : 115

fish_XP_008336142.1 : ------------------------------------------------------------------------------------------------------------------------- : -

fish_XP_008417602.1 : ------------------------------------------------------------------------------------------------------------------------- : -

fish_XP_009510531.1 : ------------------------------------------------------------------------------------------------------------------------- : -

fish_XP_010752845.1 : ------------------------------------------------------------------------------------------------------------------------- : -

fish_XP_010755274.1 : ------------------------------------------------------------------------------------------------------------------------- : -

fish_XP_010767524.1 : ------------------------------------------------------------------------------------------------------------------------- : -

fish_XP_010781233.1 : ------------------------------------------------------------------------------------------------------------------------- : -

fish_XP_010791966.1 : ------------------------------GHPAQTS------------------------------------------------------------------------------------ : 54

fish_XP_012687177.1 : ------------------------------------------------------------------------------------------------------------------------- : -

fish_XP_012708385.1 : ----------------------------------QKA------------------------------------------------------------------------------------ : 108

fish_XP_012713253.1 : ------------------------------------------------------------------------------------------------------------------------- : -

fish_XP_012727798.1 : --------------------------------SEEHL------------------------------------------------------------------------------------ : 103

fish_XP_012990558.1 : ------------------------------------------------------------------------------------------------------------------------- : -

fish_XP_013126906.1 : ------------------------------------------------------------------------------------------------------------------------- : -

fish_XP_013796981.1 : ------------------------------------S------------------------------------------------------------------------------------ : 126

fish_XP_014005049.1 : ------------------------------------------------------------------------------------------------------------------------- : -

fish_XP_014049585.1 : ------------------------DQG---------------------------------------------------------------------------------------------- : 110

fish_XP_014325953.1 : ------------------------------------------------------------------------------------------------------------------------- : -

fish_XP_015195770.1 : ------------------------------------------------------------------------------------------------------------------------- : -

fish_XP_015197335.1 : ------------------------------------------------------------------------------------------------------------------------- : -

fish_XP_015232478.1 : ------------------------------------------------------------------------------------------------------------------------- : -

fish_XP_015252023.1 : ------------------------------------------------------------------------------------------------------------------------- : -

fish_XP_015264177.1 : ------------------------------------------------------------------------------------------------------------------------- : -

fish_XP_015801149.1 : ------------------------LAAVGVAP--QAP------------------------------------------------------------------------------------ : 116

fish_XP_015810486.1 : ------------------------------------------------------------------------------------------------------------------------- : -

fish_XP_016115105.1 : ------------------------------------------------------------------------------------------------------------------------- : -

fish_XP_016117315.1 : ------------------------------------------------------------------------------------------------------------------------- : -

fish_XP_016395637.1 : ----------------------------------IKR------------------------------------------------------------------------------------ : 101

fish_XP_017207716.1 : ------------------------------------------------------------------------------------------------------------------------- : -

fish_XP_017277259.1 : -----------------------------KPHTQKSC------------------------------------------------------------------------------------ : 120

fish_XP_017277301.1 : ------------------------SSTAGNTP--QLP------------------------------------------------------------------------------------ : 116

fish_XP_017325460.1 : ------------------------------------------------------------------------------------------------------------------------- : -

fish_XP_017346953.1 : ------------------------TKT-------ISI------------------------------------------------------------------------------------ : 107

fish_XP_017550536.1 : ------------------------------------------------------------------------------------------------------------------------- : -

fish_XP_017550824.1 : ------------------------------------------------------------------------------------------------------------------------- : -

fish_XP_017554653.1 : ------------------------------------------------------------------------------------------------------------------------- : -

fish_XP_017569575.1 : ------------------------VKG-------GST------------------------------------------------------------------------------------ : 112

fish_XP_018413083.1 : ------------------------------------A------------------------------------------------------------------------------------ : 115

fish_XP_018429299.1 : ------------------------------------------------------------------------------------------------------------------------- : -

fish_XP_018521334.1 : ------------------------------------------------------------------------------------------------------------------------- : -

fish_XP_018526467.1 : ------------------------PPATSDSPSGQVS------------------------------------------------------------------------------------ : 118

fish_XP_018541041.1 : ------------------------------------------------------------------------------------------------------------------------- : -

fish_XP_018558293.1 : ------------------------------------------------------------------------------------------------------------------------- : -

fish_XP_018595329.1 : ------------------------------------------------------------------------------------------------------------------------- : -

fish_XP_018611696.1 : ------------------------DAGA------AQT------------------------------------------------------------------------------------ : 121

fish_XP_018956725.1 : ------------------------------------------------------------------------------------------------------------------------- : -

frog_XP_018091031.1 : QVPSTLASSTNQEPIEKGKASAQTHTDSSSSLEITTP-----------------------------------TDAVQVP------------------------------------------ : 213

Hemichordata_XP_006825609.1 : KNDDVVFFNFDENKRYATSFDCTKNMTITFPQRRTRA------------------------------------------------------------------------------------ : 608

lance_EEN48978.1 : ------------------------------------------------------------------------------------------------------------------------- : -

lance_EEN55742.1 : ------------------------------------------------------------------------------------------------------------------------- : -

mammal_XP_001378512.1 : ------------------------------------------------------------------------------------------------------------------------- : -

mammal_XP_003771993.1 : ------------------------------------------------------------------------------------------------------------------------- : -

mammal_XP_003771994.1 : --------------------------SQDI------T------------------------------------------------------------------------------------ : 121

mammal_XP_003782717.1 : --------------------------ATEN------T------------------------------------------------------------------------------------ : 116

mammal_XP_003982796.1 : ------------------------------------------------------------------------------------------------------------------------- : -

mammal_XP_004389713.1 : --------------------------DHDL------T------------------------------------------------------------------------------------ : 122

mammal_XP_004582357.1 : --------------------------KAES------T------------------------------------------------------------------------------------ : 106

mammal_XP_004582358.1 : --------------------------DHVL------Q------------------------------------------------------------------------------------ : 124

mammal_XP_004622892.1 : --------------------------DHDL------K------------------------------------------------------------------------------------ : 122

mammal_XP_004646313.1 : --------------------------DQDL------R------------------------------------------------------------------------------------ : 124

mammal_XP_004702866.1 : --------------------------DHDL------T------------------------------------------------------------------------------------ : 122

mammal_XP_005340592.1 : --------------------------DHDL------R------------------------------------------------------------------------------------ : 123

mammal_XP_005388084.1 : --------------------------DQDF------R------------------------------------------------------------------------------------ : 124

mammal_XP_006163552.1 : --------------------------NYDL------G------------------------------------------------------------------------------------ : 124

mammal_XP_006779273.1 : ------------------------------------------------------------------------------------------------------------------------- : -

mammal_XP_006834357.1 : --------------------------DHDL------T------------------------------------------------------------------------------------ : 122

mammal_XP_006891533.1 : --------------------------EHDL------T------------------------------------------------------------------------------------ : 122

mammal_XP_007522555.1 : --------------------------DHDV------R------------------------------------------------------------------------------------ : 119

mammal_XP_007938002.1 : --------------------------NDDL------T------------------------------------------------------------------------------------ : 122

mammal_XP_008062458.1 : --------------------------DKGN------P------------------------------------------------------------------------------------ : 121

mammal_XP_008259950.1 : --------------------------DHDL------R------------------------------------------------------------------------------------ : 124

mammal_XP_008524371.1 : ------------------------------------------------------------------------------------------------------------------------- : -

mammal_XP_008820771.1 : --------------------------DHDL------R------------------------------------------------------------------------------------ : 123

mammal_XP_010601767.1 : --------------------------DGNL------T------------------------------------------------------------------------------------ : 124

mammal_XP_011853657.1 : --------------------------DYDP------R------------------------------------------------------------------------------------ : 124

mammal_XP_012791283.1 : --------------------------DKEK------S------------------------------------------------------------------------------------ : 120

mammal_XP_012881542.1 : --------------------------EHDL------R------------------------------------------------------------------------------------ : 123

mammal_XP_012934202.1 : --------------------------DKEI------S------------------------------------------------------------------------------------ : 126

mammal_XP_013013639.1 : --------------------------DQDL------S------------------------------------------------------------------------------------ : 120

mammal_XP_016016798.1 : ------------------------------------------------------------------------------------------------------------------------- : -

mammal_XP_016281899.1 : ------------------------------------------------------------------------------------------------------------------------- : -

mammal_XP_017199853.1 : ------------------------------------------------------------------------------------------------------------------------- : -

mammal_XP_017508280.1 : --------------------------DHDL------G------------------------------------------------------------------------------------ : 133

mollusk_EKC42356.1 : ------------------------------------------------------------------------------------------------------------------------- : -

mollusk_XP_011436570.1 : ------------------------------------------------------------------------------------------------------------------------- : -

mollusk_XP_011440496.1 : ------------------------------------------------------------------------------------------------------------------------- : -

mollusk_XP_013061423.1 : ------------------------------------------------------------------------------------------------------------------------- : -

mollusk_XP_013061425.1 : ------------------------------------------------------------------------------------------------------------------------- : -

Musmu_NP_034286.2 : --------------------------DHGL------R------------------------------------------------------------------------------------ : 120

rept_XP_008110733.2 : --------------------------QTGFAR----S------------------------------------------------------------------------------------ : 149

rept_XP_008112638.1 : ------------------------------------------------------------------------------------------------------------------------- : -

rept_XP_015264172.1 : --------------------------YES-PE----P------------------------------------------------------------------------------------ : 158

shark_XP_007886940.1 : ------------------------------------------------------------------------------------------------------------------------- : -

Strpu_XP_003727489.1 : ------------------------------------------------------------------------------------------------------------------------- : -

Strpu_XP_011667260.1 : ------------------------------------------------------------------------------------------------------------------------- : -

turtle_XP_006139744.1 : --------------------------DTSESQELRVS------------------------------------------------------------------------------------ : 144

turtle_XP_007060369.1 : --------------------------DASESQELRLS------------------------------------------------------------------------------------ : 144

turtle_XP_014430084.1 : ------------------------------------------------------------------------------------------------------------------------- : -

Xenla_OCT63184.1 : ------------------------------------------------------------------------------------------------------------------------- : -

Xenla_XP_018122800.1 : ------------------------------------K------------------------------------------------------------------------------------ : 110

Xentr_XP_002943568.3 : QTPLADLCSTPGPPLVQQTPLADLCSTTGPPLVPQTP------------------------------------------------------------------------------------ : 268

Xentr_XP_012813408.1 : -----------------------------AQGSRSES------------------------------------------------------------------------------------ : 113

* 860 * 880 * 900 * 920 * 940 * 960

Homsa_NP_060124.2 : -------------------------------------DMAN----------------PSAMSTTAKGSKSLKVELI--EDKIDYTKERQP----------------------SIDLTC--- : 161

Homsa_NP_689916.2 : -------------------------------------EIRD----------------IKQEESILMKENVL-DEVA---NAKHKKKGKLK----------------------PEQLTC--- : 163

fish_XP_015241820.1 : ------------------------------------------------------------------------------------------------------------------------- : -

fish_XP_018543893.1 : ------------------------------------------------------------------------------------------------------------------------- : -

fish_CDQ83481.1 : ------------------------------------------------------------------------------------------------------------------------- : -

fish_CDQ89582.1 : ------------------------------------------------------------------------------------------------------------------------- : -

fish_XP_005809248.1 : ------------------------------------------------------------------------------------------------------------------------- : -

fish_XP_007231320.1 : ------------------------------------------------------------------------------------------------------------------------- : -

fish_XP_007254667.1 : ------------------------------------------------------------------------------------------------------------------------- : -

fish_XP_008277954.1 : ------------------------------------------------------------------------------------------------------------------------- : -

fish_XP_010746900.1 : ------------------------------------------------------------------------------------------------------------------------- : -

fish_XP_012685053.1 : ------------------------------------------------------------------------------------------------------------------------- : -

fish_XP_013123332.1 : ------------------------------------------------------------------------------------------------------------------------- : -

fish_XP_014011897.1 : ------------------------------------------------------------------------------------------------------------------------- : -

fish_XP_014011904.1 : ------------------------------------------------------------------------------------------------------------------------- : -

fish_XP_014264868.1 : ------------------------------------------------------------------------------------------------------------------------- : -

fish_XP_014264875.1 : ------------------------------------------------------------------------------------------------------------------------- : -

fish_XP_014854842.1 : ------------------------------------------------------------------------------------------------------------------------- : -

fish_XP_017578465.1 : ------------------------------------------------------------------------------------------------------------------------- : -

fish_XP_012713132.1 : ------------------------------------------------------------------------------------------------------------------------- : -

fish_XP_010787190.1 : ------------------------------------------------------------------------------------------------------------------------- : -

fish_XP_017319342.1 : -----------------------------------------------------------------------------------------T----------------------IRKRDS--- : 108

Alligator_KYO30992.1 : ---------------------------------------------------------SEIDASSPGGSISKKDTEKTEATLDRAGGRKSR----------------------SLHENTAEA : 141

Alligator_XP_006025954.1 : -------------------------------------AWTK----------------PLKSVLVDGTENKLVEAEG---KTNKLLRINQL----------------------PTGQTC--- : 183

amphibia_XP_018413082.1 : -------------------------------------TSAT----------------PQEKNNSTEESEDTYKESG-----------NLN----------------------TTPAECSYS : 147

bact_AGA70736.1 : ------------------------------------------------------------------------------------------------------------------------- : -

bact_CUJ86040.1 : --------------------------------------------------------------WLDYKLDPSNGRIE------------------------------------KHIRAYFGD : 100

bact_ESW58583.1 : ------------------------------------------------------------------------------------------------------------------------- : -

bact_GAC67715.1 : ---------------------------------------------------------SLDAVRTAESVSDHLDLDEVHSKLASELLRAQL----------------------ASGSAC--- : 144

bact_KMO66808.1 : ---------------------------------------------------------LIEVKDGVSVELYDLAGFSTKTTRTFREVRNQN----------------------DKQHILSEL : 140

bact_KUO79116.1 : ---------------------------------------------------------QFASRIAHTITSKEIDGKLVQIIHYALHS--------------------------RINSVCAEK : 124

bact_KYG06363.1 : ---------------------------------------------------------HTQNGLYHLLLMGLAGGSRLEWRPLVDSLRLFG----------------------SAYANLVDV : 132

bact_ODA70488.1 : ---------------------------------------------------------TSVDVYDIAGFSLDHLRSAEHVDAEDREETCAR----------------------ASRELLNAQ : 154

bact_OGP49286.1 : ----------------------------------------------------------------------------------------------------------------PGNHDIDLS : 102

bact_SCF05533.1 : -------------------------------------G-------------------DGPEGLVASLHEVAGGSLARFTPPGRESTNLLR----------------------VVRHLSREV : 147

bact_SCX17622.1 : ------------------------------------------------------------------------------------------------------------------------- : -

bact_SFJ80976.1 : ----------------------------------------------------------------------------------------------------------------EDGRLV--- : 112

bact_WP_006128369.1 : ---------------------------------------------------HMLRIRTCGATDGSHPLVVSLQQFAGGGTHRYVSPRSPS----------------------TALQLTAEL : 172

bact_WP_006365791.1 : ------------------------------------------------------------------------------------------------------------------------- : -

bact_WP_006849212.1 : ------------------------------------------------------------------------------------------------------------------------- : -

bact_WP_007897046.1 : ------------------------------------------------------------------------------------------------------------------------- : -

bact_WP_030194308.1 : -------------------------------------AGASVDVYDIAGFSLDLLRSADHVDAEDREESCGRAARELLGAQLDAAGAPDYRG--------------------TVGTVL--- : 171

bact_WP_030198971.1 : ---------------------------------------------------------RRVPPLELSGHDEQLRVDIYKIAGETRGVR-------------------------TARVAT--- : 134

bact_WP_030898614.1 : ---------------------------------------------------------PGGIKVMFQEIAGGSIRDVRPLSATLRGRELPE----------------------LVGEVARSI : 156

bact_WP_041219555.1 : ------------------------------------------------------------------------------------------------------------------------- : -

bact_WP_050505446.1 : ---------------------------------------------------------EVLSAWAAPQDVE--------------------------------------------------- : 113

bact_WP_055538636.1 : ---------------------------------------------------------RRVPPLELSGHDEQLRVDIYKVAGETRGVR-------------------------PARVAT--- : 134

bact_WP_062134708.1 : ------------------------------------------------------------------------------------------------------------------------- : -

bact_WP_062799339.1 : ----------------------------------------------------------------------------------------------------------------TISAMA--- : 67

bact_WP_066463897.1 : ------------------------------------------------------------------------------------------------------------------------- : -

bird_XP_002194798.1 : -------------------------------------GSSG----------------AVGTTSSENTAPTSG---KKQKRNKKSQLLSAD----------------------EVLDIR--- : 152

bird_XP_005010984.1 : -------------------------------------ILQD----------------SELEPVENKTAIKSADEEN---VSEKSPSSSQH----------------------PARNIC--- : 165

bird_XP_005505023.1 : -------------------------------------ILQD----------------SKTEPTEDKKAMESADKEN---GSDTPLLSSQH----------------------PTGKMC--- : 166

bird_XP_008492486.1 : -------------------------------------TLQD----------------SKTEPTVREKAFKSIDKEN---GSEDTLSSSRD----------------------PTGKMC--- : 166

bird_XP_008628214.1 : -------------------------------------ILKD----------------SKMKPIEDTKASK-AGIKN---ETDEPLSSSQQ----------------------PAGKMC--- : 165

bird_XP_009068034.1 : -------------------------------------DSCG----------------AVDKTSSSNTAPDSG---KRQRRSNKSQLKSSE----------------------EVLELT--- : 150

bird_XP_009080343.1 : -------------------------------------ILQD----------------SKTEPMEDTKTVKSTGKEI---VTEEPLSSSQH----------------------PTGNMS--- : 166

bird_XP_009486154.1 : ---------------------------------------------------------LQDSKAEPIEDKNAMKSADKENVSEKPLSSSQH----------------------PTGKMC--- : 166

bird_XP_009640122.1 : ---------------------------------------------------------LQDSNMEPIKDEKAMKSADKENVSEKPPSSSQH----------------------PTGEMC--- : 164

bird_XP_009672623.1 : -------------------------------------ISQN----------------SKTETLEDKGAEKSDGKED---ISEKSLSSNQH----------------------PNRKTC--- : 154

bird_XP_009695665.1 : -------------------------------------DSCD----------------PVDETSRDHTASTSG---KKQRRSNNSQRKSSD----------------------EVLELS--- : 298

bird_XP_009886214.1 : ---------------------------------------------------------LQYSKTESIEDEKAMKSADKENLLEKVLSSSQH----------------------PTGRMC--- : 166

bird_XP_009934438.1 : ---------------------------------------------------------LQDAKAEPTEDKKAMNSADKQNVSEKPLSSSQH----------------------PAGNMC--- : 160

bird_XP_009999877.1 : -------------------------------------ILQD----------------STTEPIEDQKAIKSSDKEN---VSEEALSSIQH----------------------PTGKMC--- : 165

bird_XP_010119033.1 : -------------------------------------ILQD----------------SKMEPIEDKKATESADKEN---VSEKAPSNSQH----------------------PTGNTC--- : 164

bird_XP_010144931.1 : -------------------------------------ILQD----------------SKMEPIAYKKAMKSADKEN---VSEEPLSNSQH----------------------AAGKTC--- : 154

bird_XP_010222910.1 : ----------------------------------------N----------------CKTESLEDKAEKTTDAKED---ASEKALSSNQY----------------------PTRKTC--- : 155

bird_XP_013160560.1 : -------------------------------------ILQD----------------SKMEPIEDKNPEKSDDKEN---VSEKPMSSSQH----------------------PDGKMC--- : 170

bird_XP_014795808.1 : ---------------------------------------------------------PQDFKTEPNEEEKAMKSADKENISEKPLSSSQH----------------------PTGNMC--- : 166

bird_XP_015156587.1 : ---------------------------------------------------------ILQDSEMEPIENKREVKTADENVPEQSLSSSQH----------------------PTRNMC--- : 158

bird_XP_015709021.1 : -------------------------------------ILQD----------------SEREPIENKREVKTAD-ES---VPDQSLSSSQH----------------------PTRNMC--- : 170

brachiop_XP_013415988.1 : -------------------------------------TTVSSSMRVGDSMIPPLAAERTSTLITGSTPSKEKCVTNVIKITNSSVNVVIGDQNQQNIVHPAKQSSRKFISIDEKAKTKEQK : 260

Cioin_XP_018668123.1 : ----------------------------------------------------------------------------------------------------------------TTPVPC--- : 63

Cnidaria_EDO35430.1 : -------------------------------------LSDKWQAWGESIPVINREMSCALAQTGHQVACLVLKATAVEIEEARSHNVNLFTCDDDNNIFKGKIRSMYHHETFPFEPDLVVG : 225

Cnidaria_KXJ09690.1 : -------------------------------------ERKEVKVNAGGAKPKPSDEKSSSPSTDRKKKKKKKAGGFLLSDVNLSEPQNDSDKEKKDLK--------------SSEKDTMKL : 241

Cnidaria_KXJ11435.1 : -------------------------------------ERVKSWEESCSAAKKPPEKSSSSPASDRKKKRKKKAGFLLSDVDLTEPQNDSDKEK-------------------KDLAKSSSE : 223

Cnidaria_KXJ17955.1 : -------------------------------------AYGQGKTIGETYPSKILVVCDKWSGWHHSHYTVNRHLCKTLAKQPGVQVACIV----------------------LDAKET--- : 316

Cnidaria_KXJ23466.1 : -------------------------------------FLARSYEGLREYSKAYEELKLLKVSNPKDPDVLILLEKVARQVDKERREESHS----------------------TTCQAS--- : 248

Cnidaria_XP_015751626.1 : LKAKVEYHLSQVQDKDVLDKSQPSVSTSLNNISTDSIPGGK--VSGEAYPRRILLISEGWSSWQRSVCTVNRQLSRSFARNQDIYVACLVLHASEQETIAATRDGV------NLIKATIQP : 620

Cnidaria_XP_015751629.1 : --------------------------------------------SGEAYPRRILLISEGWSSWQRSVCTVNRQLSRSFARNQDIYVACLVLHASEQETIAATRDGVNLIK--ATIQPGLSE : 498

Cnidaria_XP_015758975.1 : -------------------------------------ESTPVSGDSAAYPRRILLISERWSSWQRSVYTVNRQLSRSFARNEDIYVACLVLHASEQETIAATRDGV------NLIKATIQP : 467

Cnidaria_XP_015766106.1 : ----------------------------------------------------------------------------------------------------------------FNRGDI--- : 56

Cnidaria_XP_015766118.1 : ---------------------------------------------------------GDEHSCEEPSGTSTGKKEKEKENKGVTSLDC------------------------NSGDDC--- : 149

Cnidaria_XP_015766124.1 : ---------------------------------------------------------SLGSSSRSVPLQRSAFDIKSEKSESIRQDDGID----------------------THGEPCKAT : 139

coelacanth_XP_006000513.2 : -------------------------------------QTTQ----------------SPTEPTLGTEPTS-----------------GAH----------------------QSKRTC--- : 86

fish_CDQ87765.1 : -------------------------------------EELRHSGSDVASPILLPMVESEYIPAYGNMEDNTRQSETSISSFDHLRETVPN----------------------LEKRIC--- : 403

fish_CDQ92295.1 : -------------------------------------SESR----------------PVSIPNECEPKSGGA-----------------S------------------------LPLC--- : 144

fish_KKF13953.1 : -------------------------------------DRDQ----------------DV---LKERQTTQ---------------EQCVL----------------------TSKQDC--- : 138

fish_XP_004085798.2 : -----------------------------------FMETETVSSDSGIQSLSSAMQMLEMPQYKMKLGQDESSNYAHSMIEIPDSETAVQ----------------------PQKSFS--- : 198

fish_XP_004552746.3 : ---------------------------------------------------------EQVSQWLVQHVNV------------------------------------------YSKYAE--- : 114

fish_XP_004558390.1 : -------------------------------------ETEQ----------------DV---FEENHASQ---------------EQCVL----------------------ISKEDC--- : 152

fish_XP_004576250.1 : ---------------------------------------------------------DTLQTGRDRISSVTDQNTRADTQLHKGNTDNTTE---------------------LQRRVC--- : 214

fish_XP_005161888.1 : -------------------------------------ADTR----------------AD---IKEVDEED---------------PVPAV----------------------LRNRDT--- : 125

fish_XP_005478813.2 : ---------------------------------------------------------KYAERLQEEDVSGDCLVCFRKQDLADLGVKGGP----------------------AIRIISALK : 152

fish_XP_005806405.2 : -------------------------------------DRKV----------------QDDSTNACNDIAKSG-----------------V-------------------------GSC--- : 142

fish_XP_006625674.1 : -------------------------------------SASANKKCAEEKRNPEGNKTSPGIKADIELSEQKSADLKLKATTNENGNDNKEAKGLLHAQKQSESKAESSPKYPQQKNTC--- : 377

fish_XP_006635547.1 : -------------------------------------VYGR----------------AVQSGGRERREAIRTAASALKSRDGLGGPACRA----------------------PARRDC--- : 230

fish_XP_006635548.1 : -------------------------------------EVGA----------------AKEPSPVKEKKARKE-----------------K----------------------SDLSLC--- : 137

fish_XP_006791552.1 : -------------------------------------QSDQ-----------------------EKETAQ---------------EQCVL----------------------ISKEDC--- : 141

fish_XP_006791631.1 : -------------------------------------KPEH----------------KKNPTDSSENTSSTE-----------------V-----------------------TLSFC--- : 143

fish_XP_007241837.1 : -------------------------------------ISSP----------------QK---GELKQDSV-----------------MIQ----------------------KTRRDS--- : 132

fish_XP_007242299.1 : -------------------------------------SPTHKKRKNKTVVSSQNVPPDITETEPAETTDLPKMQVSNAEEPEIKSESKQP----------------------QSKHLC--- : 263

fish_XP_007557414.1 : -------------------------------------VRNR----------------EV---SKERQNVQ---------------KECVL----------------------SSKEDC--- : 134

fish_XP_008284284.1 : ----------------------------------------------------------------------------------------------------------------QPGKPC--- : 101

fish_XP_008284762.1 : -------------------------------------DEAE----------------QSSIPTACSDSWTSS-----------------V----------------------EALSFC--- : 113

fish_XP_008285761.1 : -------------------------------------VSDQ----------------EV---SEEK--------------------QCML----------------------ISKQDC--- : 135

fish_XP_008336142.1 : ---------------------------------------------------------EQVCQWLLQHVIV------------------------------------------YRKYAD--- : 110

fish_XP_008417602.1 : -------------------------------------SAENETVSSDSGIQSLSSSVQMLQMTSDLLRVYPNIAERQFDIQTHDNLKAPQ----------------------RQRPVC--- : 243

fish_XP_009510531.1 : ---------------------------------------------------------LHDSKTEPIEDKKAMKPADEQNASEKPLSSSQH----------------------PTGKMC--- : 165

fish_XP_010752845.1 : ---------------------------------------------------------EQVAQWLLQHVNV------------------------------------------YSRYAE--- : 113

fish_XP_010755274.1 : ----------------------------------------------------------------------------------------------------------------TNQPVC--- : 99

fish_XP_010767524.1 : ---------------------------------------------------------QQVTQWLLQHVKV------------------------------------------YDKYAE--- : 112

fish_XP_010781233.1 : ----------------------------------------------------------------------------------------------------------------QCGRPC--- : 121

fish_XP_010791966.1 : -------------------------------------VRNT----------------ED---SVERQTAQ---------------EQCVL----------------------TSKEDC--- : 79

fish_XP_012687177.1 : ----------------------------------------------------------------------------------------------------------------LEQRIS--- : 82

fish_XP_012708385.1 : -------------------------------------KEEE----------------NV---LQEKQPVV---------------QQCVL-----------------------TKEDC--- : 132

fish_XP_012713253.1 : ----------------------------------------------------------------------------------------------------------------QPGKPS--- : 99

fish_XP_012727798.1 : -------------------------------------DKDV----------------QKDSS-TCSDIEKKS-----------------V-------------------------AFC--- : 125

fish_XP_012990558.1 : ---------------------------------------------------------EQVCQWLKQVNANHKAAELLLEE--------------------------------EVSGDCLFC : 122

fish_XP_013126906.1 : ----------------------------------------------------------------------------------------------------------------QPGKPC--- : 101

fish_XP_013796981.1 : -------------------------------------TSQN----------------SNIEPLEDKAAKKSDGKED---VSEKSLSSNQH----------------------PTKKTC--- : 166

fish_XP_014005049.1 : ------------------------------------------------------------------------------------------------------------------------- : -

fish_XP_014049585.1 : -------------------------------------LSEE----------------EK---QTHQGQSV---------------EDNVL----------------------TTKRDC--- : 135

fish_XP_014325953.1 : ----------------------------------------------------------------------------------------------------------------KQQVSC--- : 94

fish_XP_015195770.1 : ----------------------------------------------------------------------------------------------------------------PSSRPC--- : 113

fish_XP_015197335.1 : -------------------------------------ISNEEKVVKTNISLASESDKSPADAMCLKNVDISLGTDIKTELLPSREKTYPESTIFKE----------------PELEEK--- : 178

fish_XP_015232478.1 : ----------------------------------------------------------------------------------------------------------------STGEPT--- : 30

fish_XP_015252023.1 : ----------------------------------------------------------------------------------------------------------------KPGKPS--- : 93

fish_XP_015264177.1 : -------------------------------------------------------TQFNVIPVSLQHGASIQQQSGKKNISTFSKTESI-----------------------TNTDNPGES : 168

fish_XP_015801149.1 : -------------------------------------VRER----------------DV---SEERPT-L---------------EQSSS----------------------SSKEDC--- : 140

fish_XP_015810486.1 : ----------------------------------------------------------------------------------------------------------------QPGKPC--- : 99

fish_XP_016115105.1 : ------------------------------------------------------------------------------------------------------------------------- : -

fish_XP_016117315.1 : -------------------------------------TDCQTKEVLKLAPSQEKMLEETSSHSTSKESELREAAGLKTVVIDADKTDKEKIKRK------------------PGQHSC--- : 213

fish_XP_016395637.1 : -------------------------------------VDTR----------------AD---IREEHKED---------------SVSVI----------------------TTKRDS--- : 126

fish_XP_017207716.1 : -------------------------------------DSLDIKTNPQTAHSQPKGPLASTFTKETELGKSPAFKTAVINEENIISKENSTRK--------------------SEQHSC--- : 198

fish_XP_017277259.1 : -------------------------------------EREE----------------QGNIPNVCNDTLNSD-----------------V-------------------------AFC--- : 143

fish_XP_017277301.1 : -------------------------------------VTDQ----------------NE---SEERQTPQ---------------EEFTL----------------------SSKDDC--- : 141

fish_XP_017325460.1 : ----------------------------------------------------------------------------------------------------------------LPCTSC--- : 120

fish_XP_017346953.1 : -------------------------------------TSTP----------------QK---GE-KEDSL---------------IVP------------------------TTKRDS--- : 129

fish_XP_017550536.1 : ------------------------------------------------------------------------------------------------------------------------- : -

fish_XP_017550824.1 : ----------------------------------------------------------------------------------------------------------------QSSKSC--- : 105

fish_XP_017554653.1 : -------------------------------------SSQKGGGEGKPKGSSMNVPDQVTRRESEEPEDLKTQDTNREKVPEMKKTEMQT----------------------KCKHSC--- : 245

fish_XP_017569575.1 : -------------------------------------TSSG----------------QK---GEIKQDSV---------------AVR------------------------TTKSDA--- : 135

fish_XP_018413083.1 : -------------------------------------TLNE----------------DEPREISNVTEEQPCCSEV-----------DVK----------------------CRKVTC--- : 147

fish_XP_018429299.1 : -------------------------------------ESKEFPGDIQQEAAETSADQELTQPMQHLSLENQTQGENLSNTTKSDKNKCIQHT--------------------EPGTTA--- : 195

fish_XP_018521334.1 : ---------------------------------------------------------EQVNQWLLQHVKI------------------------------------------RGKYAE--- : 114

fish_XP_018526467.1 : -------------------------------------VSNQ----------------GV---LEERQTAQ---------------KQSVL----------------------ASKEDC--- : 143

fish_XP_018541041.1 : --------------------------------------MQTMSSDSGIQSLSSVQMLQIARNKIRSVIDQRTTNDIQLHTEMTDKATCLH----------------------LKKPVC--- : 272

fish_XP_018558293.1 : ----------------------------------------------------------------------------------------------------------------QPGRPC--- : 113

fish_XP_018595329.1 : --------------------------------------------------DTLQVPGNTEKLMSEDNVHDENKSLLSFNNTKKRNDEVTK----------------------AEIRIC--- : 190

fish_XP_018611696.1 : -------------------------------------LPEP----------------KT---KTSQRKSL---------------GGT------------------------SAKTDC--- : 144

fish_XP_018956725.1 : -------------------------------------NSTDKLIQLGKDSVDNISKTTPKEDPQINEPLRKRPFSGTSTSSKETEPTESAEPKTSSINADKVVEKEKHMRSRSVLHSC--- : 218

frog_XP_018091031.1 : ---------------------------------SSLTNSCS----------------MPELPLQSETPKEQHNIDLLRACNPLGSTKDLA----------------------ISLSLS--- : 260

Hemichordata_XP_006825609.1 : -------------------------------------AKIEEQRMSDKICVGSLQSSLKHLPDRSSPGNMVKEDLFTMKSQTQETETRVAKEGLDTQDIN------------STQSHQVKA : 680

lance_EEN48978.1 : ------------------------------------------------------------------------------------------------------------------------- : -

lance_EEN55742.1 : -----------------------------------------------------------RTRSSKSTSSSSSHGTITPDRPPSPTPTSQG----------------------MGCKNCTPC : 128

mammal_XP_001378512.1 : ---------------------------------------------------------PKNDTTTQDEEVYESPKTNPSKGTESNAEETKDNLP-------------------FRGQTC--- : 155

mammal_XP_003771993.1 : -------------------------------------PKID----------------TNTKSKEVYESSETEPSKETKENHPQETEDDLP----------------------YTGRPC--- : 154

mammal_XP_003771994.1 : -------------------------------------DSES----------------T--QESKPTREDIL-DVEE---VTMDEAKPKLE----------------------PKRLPC--- : 158

mammal_XP_003782717.1 : -------------------------------------DMAN----------------AFAISTVAEGSKSQKNELM--KDEIDDTNEKQP----------------------SPEVTC--- : 157

mammal_XP_003982796.1 : ---------------------------------------------------------TKKQKSILSKEDTLNGGRTTEDQNQDKLE--------------------------TEQLTC--- : 163

mammal_XP_004389713.1 : -------------------------------------ETGD----------------TKEEELILMKENAT-NEVG---ATKGKKKNKLK----------------------TEQLTC--- : 161

mammal_XP_004582357.1 : -------------------------------------DGAK----------------ASAMSPVEKDSKSLTNELM--EEQRGDTKKTPP----------------------PIEPSC--- : 147

mammal_XP_004582358.1 : -------------------------------------ESGG----------------PEEQESVLTKEKTL-DEVA---SAKDKKKNTQQ----------------------AGQLTC--- : 163

mammal_XP_004622892.1 : -------------------------------------ESVN----------------TKEQESVPIEKDSL-DYQA---ITED-QKGKLK----------------------KEQSTC--- : 160

mammal_XP_004646313.1 : -------------------------------------ENRK----------------TEGQESILMKRNPV-SKDT---NIKDKETNQPK----------------------TEKLPC--- : 163

mammal_XP_004702866.1 : -------------------------------------ESGG----------------TKEQESIPMKEKAT-NVVA---GTDGKTKNEVK----------------------IEQVTC--- : 161

mammal_XP_005340592.1 : -------------------------------------EIGD----------------TTEQQSILTKESAL-SERT---NTKDEKKNELQ----------------------TEQLTC--- : 162

mammal_XP_005388084.1 : -------------------------------------EIRN----------------TKGQESILVTENAV-SEVS---DTTDKKKNKPK----------------------TENFTC--- : 163

mammal_XP_006163552.1 : -------------------------------------ENGD----------------NKEQESTLMKEDAL-TEVV---NTKDKKKNKLK----------------------TEQLTC--- : 163

mammal_XP_006779273.1 : ---------------------------------------------------------TKEQESVLIKENALNEVVTTKDKKKKKLK--------------------------TEQLTC--- : 162

mammal_XP_006834357.1 : -------------------------------------EIRN----------------AKEQESILTKVNAT--------IEQSRKKNELK----------------------TQPVTC--- : 157

mammal_XP_006891533.1 : -------------------------------------ETRD----------------TEKQELSLLKENAT-TEEA---AMKGKENKALA----------------------PEKLTC--- : 161

mammal_XP_007522555.1 : -------------------------------------EIQN----------------AKAQELAATGKNAQ-DEVG---ITEEKKKKR-K----------------------IVQSTC--- : 157

mammal_XP_007938002.1 : -------------------------------------NIRD----------------AKEQESIVKKENAT-NEAA---VTEGRKKNKGK----------------------IEQLTC--- : 161

mammal_XP_008062458.1 : -------------------------------------GMAT----------------ASAMSTVAKGPDSLNKELT--DDEIKDTKEKPP----------------------FMEITC--- : 162

mammal_XP_008259950.1 : -------------------------------------ETTE----------------IEVQESIPLKEKAL-DETV---NAAD-KENAIQ----------------------TERLTC--- : 162

mammal_XP_008524371.1 : ---------------------------------------------------------RETRDTKEQESILMKENALNEVVTKDKQKNKLQ----------------------AEQLTC--- : 162

mammal_XP_008820771.1 : -------------------------------------ETEH----------------TKGQEASLMKENAP--REA---VTEDMKDSNLE----------------------TEPMSC--- : 161

mammal_XP_010601767.1 : -------------------------------------EIRD----------------TKDQESILKKENAL-SEVS---NAIDKKKNEPK----------------------TEYLTC--- : 163

mammal_XP_011853657.1 : -------------------------------------EVRD----------------IKERESILVKENVLEEEVA---NAKDKKKGELK----------------------PEQLTC--- : 164

mammal_XP_012791283.1 : -------------------------------------DTVD----------------TSTTHTVAKSSKSVENEFI--ED---DIQEKQL----------------------STEQTC--- : 158

mammal_XP_012881542.1 : -------------------------------------ETNN----------------TKENT----------VSEV---EIKDKEKDKPE----------------------AEQFTC--- : 153

mammal_XP_012934202.1 : -------------------------------------DMDN----------------ASKVSPAAKQSKSLKNEPM--ENPPDD----------------------------MPQPTC--- : 161

mammal_XP_013013639.1 : -------------------------------------ESRN----------------IKDQDSIPMEENAA-NEVS---NTIDKKKNKLK----------------------TENLTC--- : 159

mammal_XP_016016798.1 : ---------------------------------------------------------TKEQDSVLMKENELNEVATTKNTKKNKLK--------------------------TEQLTC--- : 163

mammal_XP_016281899.1 : ---------------------------------------------------------DEVISIINSQESKLSRKNILDDAEGTKKKTNPKVE--------------------PKRLFC--- : 149

mammal_XP_017199853.1 : ---------------------------------------------------------KQPLVQKEKRDTSKQKQKNKENSDPADGSAASP----------------------GEKEPK--- : 136

mammal_XP_017508280.1 : -------------------------------------ETRE----------------SKEQESNLKEENAL-NEVA---TVKDKNKINLK----------------------TEQLTC--- : 172

mollusk_EKC42356.1 : ----------------------------------------------------------------------------------------------------------------PICATC--- : 86

mollusk_XP_011436570.1 : -------------------------------------SDMPYGDKKRLFNIKTSILKEESLNGPIFENASIGYKSLEDNENTNIDHAGQS----------------------VGDQTHNET : 340

mollusk_XP_011440496.1 : ------------------------------------------------------ISFEDFAGHQIQHFVDRNTDWYVKFKDKGKIMVRLLPK--------------------KIKEKMPLK : 159

mollusk_XP_013061423.1 : -------------------------------------LEKIQISEGVSTECKEQKELNELKELKEQKEVRDMSNTELRDFLTSRLEQEGQ----------------------PTEDLC--- : 269

mollusk_XP_013061425.1 : ----------------------------------------------------------------------------------------------------------------LRNFDC--- : 104

Musmu_NP_034286.2 : -------------------------------------ETGQ----------------NEEQEPSLTKENML--GDV---VTKDMEDNKPK----------------------PEQMSC--- : 158

rept_XP_008110733.2 : -------------------------------------ECTE----------------SPEFVLQES---------------------NHF----------------------PTRQTC--- : 171

rept_XP_008112638.1 : -------------------------------------NQEENKHKPASDLKPASLPRGSLKPQCSNEKNTTHSSDTHSEMESTVNISVPL----------------------DPVRLS--- : 171

rept_XP_015264172.1 : -------------------------------------ATSE----------------QPQPPIQETMDNVIGPKDG---E-------KPL----------------------PDRQTC--- : 191

shark_XP_007886940.1 : -------------------------------------------------------------------------------------------------------------------DQC--- : 18

Strpu_XP_003727489.1 : ----------------------------------------------------------------------------------------------------------------PLRRIC--- : 69

Strpu_XP_011667260.1 : -------------------------------------KSSSSLPMGNIHVDSGGVEAPAQLNVTHPNQLVREEAQGNVQALLDASSSQIS----------------------LDQSTGVSA : 182

turtle_XP_006139744.1 : -------------------------------------ECTE----------------SIKSVLTDAIENKVDEAKG---KSEKLLENNQH----------------------PTRQNC--- : 184

turtle_XP_007060369.1 : -------------------------------------ECTE----------------SMESVLTDRTENKLGEAKD---KSKKLLENNEH----------------------PTRQNC--- : 184

turtle_XP_014430084.1 : -------------------------------------VASCQATSHQNTASEIDISGTVEESRREETEKSLSALIKSENGTSHSVHKKRA----------------------QALTLS--- : 200

Xenla_OCT63184.1 : ----------------------------------------------------------------------------------------------------------------QQGQKS--- : 79

Xenla_XP_018122800.1 : -------------------------------------AVAT----------------PEKEKTENQEKNTSVNADT-----------EST----------------------SAKVTC--- : 142

Xentr_XP_002943568.3 : -------------------------LADLCSTTGPPLVPQTPLADLCSTPGPPLVPQTPLADLCSTPGPPLVPQTPKGQHNTNLSGACKPL---------------------GSTKDSPVL : 343

Xentr_XP_012813408.1 : -------------------------------------QETV----------------SQETPPQPSSQKKKKQKGKNVSVGGTSSEGNAK----------------------HPLTMS--- : 156

* 980 * 1000 * 1020 * 1040 * 1060 * 1080

Homsa_NP_060124.2 : ----VSYPFDEFSNPYRYKL-DF-SLQ--PETGPGN------LI-----DP----IHEF------KAFT-NT-ATAT-EEDVKMK-----------------------------------F : 215

Homsa_NP_689916.2 : ----MPYPFDQFHDSHRYIE-HY-TLQ--PETGALN------LI-----DP----IHEF------KALT-NT-ETAT-EVDIKMK-----------------------------------F : 217

fish_XP_015241820.1 : ------------------------------------------------------------------------------------------------------------------------- : -

fish_XP_018543893.1 : ------------------------------------------------------------------------------------------------------------------------- : -

fish_CDQ83481.1 : ------------------------------------------------------------------------------------------------------------------------- : -

fish_CDQ89582.1 : ------------------------------------------------------------------------------------------------------------------------- : -

fish_XP_005809248.1 : ------------------------------------------------------------------------------------------------------------------------- : -

fish_XP_007231320.1 : -------------------------------------------------------MSNF-------------------------------------------------------------- : 4

fish_XP_007254667.1 : ------------------------------------------------------------------------------------------------------------------------- : -

fish_XP_008277954.1 : ------------------------------------------------------------------------------------------------------------------------- : -

fish_XP_010746900.1 : ------------------------------------------------------------------------------------------------------------------------- : -

fish_XP_012685053.1 : ------------------------------------------------------------------------------------------------------------------------- : -

fish_XP_013123332.1 : ------------------------------------------------------------------------------------------------------------------------- : -

fish_XP_014011897.1 : ------------------------------------------------------------------------------------------------------------------------- : -

fish_XP_014011904.1 : ------------------------------------------------------------------------------------------------------------------------- : -

fish_XP_014264868.1 : ------------------------------------------------------------------------------------------------------------------------- : -

fish_XP_014264875.1 : ------------------------------------------------------------------------------------------------------------------------- : -

fish_XP_014854842.1 : ------------------------------------------------------------------------------------------------------------------------- : -

fish_XP_017578465.1 : ------------------------------------------------------------------------------------------------------------------------- : -

fish_XP_012713132.1 : ------------------------------------------ML-----TA---------------------------------------------------------------------- : 4

fish_XP_010787190.1 : ------------------------------------------MI-----DP----CHEY------KSLE-NS-HKLN-SNSLKLK-----------------------------------V : 26

fish_XP_017319342.1 : ----KPRPFGKPGIDHTYVK-HD-VLQ--PETGGID------LI-----TP----CHEY------KSFC-TA-ATLD-HQRLQAK-----------------------------------L : 162

Alligator_KYO30992.1 : ITLSKFRPFRSDNINFKYVK-NN-VLG--PETGITD------LI-----TP----CHEY------KSLA-IA-AELD-RQRLQAK-----------------------------------L : 199

Alligator_XP_006025954.1 : ----LPYPFDEFHASHRYMQ-GH-ILS-VPETGPLN------LI-----DP----AHEF------KKLR-NT-ENAT-EENIKLK-----------------------------------F : 238

amphibia_XP_018413082.1 : TPKTSPCPFDCSDARKCYTQ-HH-YLP--PESGTSN------YI-----DP----VHEY------KEFT-NT-ETAT-QEDKKMK-----------------------------------F : 205

bact_AGA70736.1 : ----LFNAYSRRDSEDASYK----TLSLLNQAGTSL----------------------Y------QVFE----AALKRYLVLINK------------------------------------ : 65

bact_CUJ86040.1 : ELVSSDYFLCRGALYFNPLKFSRQSHIINKKMRTLY-------------GPFHGDCHAG------NVLT---------KPTIDGK-----------------------------------V : 158

bact_ESW58583.1 : ----EAKTNGDRILQGISVHEDDNTLQ----QKVES------LV-----YP----HPKF------RYVE-VA------------------------------------------------Y : 59

bact_GAC67715.1 : ----SPSSVGSVLEKWLGGS----WLE-DHRGGRLNTESQ--AL-----NP----QQD-------DLFR-HG-AELLPDPVL--------------------------------------V : 198

bact_KMO66808.1 : VLGLISDQFDVESRRASYDISPIDILQ-E-------------WL-----GEKFWTTERY------KKLS-SGTSIGFADEAKVFL-----------------------------------F : 200

bact_KUO79116.1 : LKHEALLPVLEMVSEELLTKWNDDYMS--CEAQSEN------VL-----AKWLSYRLKSESTGQGSVFFNRICRLLERPQEPAFH-----------------------------------Y : 197

bact_KYG06363.1 : LWSSTQFNWTNGVRPSTEIARVLGYRL-EREQGGRI------LD-----NVSKFLLPDLITSARFVYRG----DVLP-NPIHFVK-----------------------------------Y : 201

bact_ODA70488.1 : LAVTGPPDYQGTVGSVLREWLGS-TFP-DNQRGSRVRE----VL-----GP----VQGA------RHVF-RHEGELLPNPLVLLD-----------------------------------P : 218

bact_OGP49286.1 : TTIDAHKKLGECIDKLSFSEIDK-ILS-EPDINKML------YI-----SNKIFSTEDA-----YKAIKEKFTNYLEFLRLFGHR-----------------------------------T : 170

bact_SCF05533.1 : MAAWSDPSSVTLMTPYEMLA-TVVGVD-KAEQSLSV------VA-----DL----FGAN------DVLD-DHGRAFANPRRLLDP-----------------------------------H : 209

bact_SCX17622.1 : ----SIRLFKRFQELKLAGDNELARMK-AIEAGTS-------LY-----Q-----CCEW---------T---------------------------------------------------F : 71

bact_SFJ80976.1 : -----ASLHQLAGGSHRYAP-PRSQSE-SLLRGVEA------IS-----RK----ILDA------WADP-HDVEPMRPRELLLAV-------------------------------AGERR : 173

bact_WP_006128369.1 : LSRELLTAWSLPHDIREQTP-HELLIDLIGTDRAAA------CL-----DGVESLFPAS------RPVVSGGQTFLDPRKILGPG------------------------------------ : 239

bact_WP_006365791.1 : ------------------------IIS-RAESESLD------FK-----AE----GYDL------KGSR-NA------------------------------------------------F : 34

bact_WP_006849212.1 : ----IPQPREPEGSFFITLAKSTMYYA-FPT-----------LF-----DP----------------------GQFPKSKRIKVK------------------------------------ : 121

bact_WP_007897046.1 : ------------------------------------------------------------------------------------------------------------------------- : -

bact_WP_030194308.1 : ----REWLGQDFPDDRRGARVREVLGR-LQRTGHVF-------------------RHEG------ELLP-DPLVLLHPTLGVADR------------------------------------ : 225

bact_WP_030198971.1 : ----TSVSIDALSGVSRELL-EAQFMN-HAGGDRMT------VR-----QT----LEAW---MGERFLD-GKRGEALRETRRLAG-----------------------------------I : 195

bact_WP_030898614.1 : LSDWNPTPTDIRRMAVRDFVLDHVGTR-TDKDGPVAKLAEQLAY-----DPVDDQVPLW--------ITFATGGVIPNVVAWLER-----------------------------------P : 228

bact_WP_041219555.1 : ----LFNAYSRRDSEDASYK----TLSLLNQAGTSL----------------------Y------QVFE----AALKRYLVLINK------------------------------------ : 70

bact_WP_050505446.1 : ----LMRPHELLLAVVGEAK-VDACLA--ATSSLFQ-------------TE----VHQE-----------DGHAFLDPRTVLGGT------------------------------------ : 163

bact_WP_055538636.1 : ----TAVSIDALSEVSRELL-KAQLGH-HAGGGPMT------VR-----QT----LEAW---MGAGFFG-GQKGEALREARRLAG-----------------------------------S : 195

bact_WP_062134708.1 : -----------------------------DEAASAE------------------------------------------------------------------------------------- : 12

bact_WP_062799339.1 : ----TLKAVETYQPALVVFVGIAGGIK-DVRIGDVV------VV-----DK----VYPY---EIGKVAD----GQFQARPDTRT------------------------------------I : 125

bact_WP_066463897.1 : ----GTLLYNVMEWAYKHLL-HETYRK-EREKGKIN-------S-----NK----CKDL-------------------QGKLKKS------------------------------------ : 105

bird_XP_002194798.1 : ----NCRPFRSQDTDFRYVK-DT-VLA--PESGVSD------LI-----IP----CHEY------KSCN-TA-AELN-KHQLQSK-----------------------------------F : 206

bird_XP_005010984.1 : ----MPHPFDNFSDGTRYIQ-HH-ILN-VPETGPLN------LI-----DP----AHEF------KLLC-NT-ENAL-EEDIKMK-----------------------------------F : 220

bird_XP_005505023.1 : ----MPYPFDNFSDGSRYTQ-HN-ILN-VPETGPLN------LI-----DP----AHEF------KLFT-NT-DKAQ-EEDIMMK-----------------------------------F : 221

bird_XP_008492486.1 : ----MPYPFDSFSDGTRYIQ-YN-ILK-VPETGPSN------LI-----DP----AHEF------KLLT-NT-DKAA-EDDILKK-----------------------------------F : 221

bird_XP_008628214.1 : ----MPHPFDNFSDGSRYKQ-YN-ILN-VPETGASN------LM-----DP----AHEF------KLFT-NT-ENAQ-EDEILMK-----------------------------------F : 220

bird_XP_009068034.1 : ----GFRPFRSQDTNFKYVK-NT-VLA--PETGVND------LI-----TP----CHEY------KSLD-TA-SGLN-RNQLQSK-----------------------------------F : 204

bird_XP_009080343.1 : ----MPYPFDNFSDCTRYIR-YN-ILN-APETGPRN------LM-----DP----VHEY------KLLT-NT-ENTL-EDDIMRK-----------------------------------F : 221

bird_XP_009486154.1 : ----MPYPFDNYTDGTRYTQ-YN-ILN-VPETGPLN------LI-----DP----AHEF------KLLT-NVEKAL--EEDIMMK-----------------------------------F : 221

bird_XP_009640122.1 : ----MPHRFDNFSDGTRYTQ-YN-ILN-VPETGPLN------LI-----DP----AHEF------KLLT-NTENSL--EKDVMMK-----------------------------------F : 219

bird_XP_009672623.1 : ----MPHPFDNFSDGVRYTH-HN-ILN-IPETGPLN------LI-----DP----AHEF------KLFT-NT-KKAQ-EEDIMMK-----------------------------------F : 209

bird_XP_009695665.1 : ----NCRPFRSRNTDFKYVK-DK-VLA--PESGVNG------LI-----TP----CHEY------KSFA-IA-AELN-KNQLQSK-----------------------------------F : 352

bird_XP_009886214.1 : ----MPYPFDNFSDGTRYIQ-YS-ILN-VPETGPLN------LI-----DP----AHEF------KLLT-NTEATA--DEDIMMK-----------------------------------F : 221

bird_XP_009934438.1 : ----MPHAFDNFRDGTRYIQ-YN-ILN-VPETGPLN------LI-----DP----VHEF------KLFT-NREKAL--EEDIMMK-----------------------------------F : 215

bird_XP_009999877.1 : ----MPYPFDNFRDGTRYIQ-HN-ILN-IPETGPLN------LI-----DP----AHEF------KSLT-NT-AKAS-EEDIMMK-----------------------------------F : 220

bird_XP_010119033.1 : ----MPYPFDNFSDATRYIE-HN-ILN-VPETGSLN------LI-----DP----AHEF------KLLT-NT-DKAL-EEDVMMK-----------------------------------F : 219

bird_XP_010144931.1 : ----MPYPFDNFSDGTRYTQ-YN-ILN-VPETGTLN------FI-----DP----AHEF------KLLT-NT-NKAL-EDNIMMK-----------------------------------F : 209

bird_XP_010222910.1 : ----MPHPFDNFRDGARYIY-HN-ILN-FPETGPLN------LI-----DP----AHEF------KLLT-NT-DNAK-EEDIMMK-----------------------------------F : 210

bird_XP_013160560.1 : ----MPYPFDNFSDGTRYTQ-YN-IIN-VPETGPFN------LI-----DP----AHEF------KLFT-NT-QNA--NEDPMMK-----------------------------------F : 224

bird_XP_014795808.1 : ----MPYPFDNFTDSTRYIQ-YN-ILN-VPETGPLN------FI-----DP----AHEF------KLLT-NTDKAV--EEDIMMK-----------------------------------F : 221

bird_XP_015156587.1 : ----MPHPFDNFSDGIRYTQ-HN-SLN-IPETGPLN------LI-----DP----VHEF------KLLT-NT-EGAS-EENIKMK-----------------------------------F : 213

bird_XP_015709021.1 : ----MPHPFDNFSDGTRYIQ-HN-ILN-VPETGPLN------LI-----DP----AHEF------KLLT-NT-ENAL-EEDIKMK-----------------------------------F : 225

brachiop_XP_013415988.1 : AVEFLNGLFNPFNYNITYSKPYEKIVDAVRQKGGFKQFGVGPLQRFLDKSP----CFDLRKNSKGKVEQVVSLPISDTKNEDLKE----------------------ESCFDDAALCSDSD : 355

Cioin_XP_018668123.1 : ----GRLSLSNFASIANHGTYKLFLFA-FSTEQTKN-----------------------------ENYG----KMVHVVTSMKQN-----------------------------------K : 111

Cnidaria_EDO35430.1 : HGTLTGPVADHFASRFSCYRVHV-FHC-VPDEGET-------FD-----DQSMEECDEV---TLAKSADFTAAIGVYLKDKWNSV-----------------------------------L : 294

Cnidaria_KXJ09690.1 : CDTALSADSAERESDFKTFSKDTSLLE-SETREQNN------LF-----KI----LIIY---NGLGLDT-LRKLFMKIHPSWSNK------------------------------------ : 306

Cnidaria_KXJ11435.1 : KDAKKLRDSVERESDFKTFNKDTSLLQ-TEIKEKNN------LS-----KI----LIVV---NGLGLDT-LRKLFMEIHPTWSNK------------------------------------ : 288

Cnidaria_KXJ17955.1 : ----DERKATRDGILLIKAA-NRTGIP-VGDMRLLL------MH-----DSFPFDEPDI---VIGHAMV-TGAEAAEIARRFKCR-----------------------------------F : 381

Cnidaria_KXJ23466.1 : ----GISPTANFVADGNYPHKVLFVCD-KWSGWHLD------IY-----SVNRHLCRHF---AKFDCLR-VACLVLEATEKDIVL-----------------------------------A : 314

Cnidaria_XP_015751626.1 : GLSEGDEHLLQLHCDFPFKV-DLIVGH-GIISGTAA------AI-----QANRLDCKRL---HVFHHLP-GEEQQRERDLGLNAD-------------------FVAGIGPLVVEQWEAIL : 705

Cnidaria_XP_015751629.1 : GDEHLLQLHCDFPFKVDLIV-GHGIIS-GTAAAIQA------NR-----LD----CKRL------HVFH-HLPGEEQQRERDLGLNA------DFVAGIGPLVVEQWEAI----------L : 579

Cnidaria_XP_015758975.1 : GLCEGDEHLLQLHSDFPFKV-DLIVGH-GILSGTAA------AI-----QANRLDCKRL---HVFHNLP-EEEQERERDLGLNAD-------------------FVAGIGPLVVEQWEAIL : 552

Cnidaria_XP_015766106.1 : ----KEWDFSLMTTVLLYSQSCKLQMNQRPDQPLAL-----------------------------RELR-NHRNKIIGHPSTEK------------------------------------M : 107

Cnidaria_XP_015766118.1 : -ETAELDIASKHVAGMSSAISGDNILK-EPSDGQAT------LR-----PEKDGPQTDF---IKEDNVD-SREAETMSKIDIDKE-----------------------CDKELSESKPQDI : 230

Cnidaria_XP_015766124.1 : NVATISEPSNNQSDTDSGINFQRNRLT-RKERNILC------VF-----KL----MYGI-------GLRALRNLFMEIHPAWSNQ------------------------------------ : 201

coelacanth_XP_006000513.2 : ----QPHPFDMQQGPHRYIV-NC-ILV--PETGPSN------LI-----DP----CHEY------KSFR-NT-GSIT-EK--MKK-----------------------------------F : 138

fish_CDQ87765.1 : ----PPRPFDKNDLSFTYIQ-ND-TLP--PESGPSN------LI-----DP----VHEY------KLLP-STEEAS--EIDILEK-----------------------------------F : 457

fish_CDQ92295.1 : ----DFRKFDDIEKEFRYVK-NT-VLP--PETGIED------II-----VP----CHEY------KSLE-NA-HKLE-PQKLKVK-----------------------------------V : 198

fish_KKF13953.1 : ----RPRPFDQEAIDFIYVK-HR-VLQ--PESGAFD------LI-----SP----CHEF------KSFA-VA-AALE-RTRLQAK-----------------------------------F : 192

fish_XP_004085798.2 : ----QLRPFDKSNSSMFYME--NAILP--PMAGPSN------LI-----DP----VHEY------VLFPNDDEAS---EKEILYE-----------------------------------F : 252

fish_XP_004552746.3 : ----RLQEEDVSGDCLVCFR-KQDLVD-LNVKGGPA-------------------VRIF------SALK-QLNQKPEPTLQPITD-----------------------------------Q : 168

fish_XP_004558390.1 : ----KPRPFDQEGIDFLYVK-HR-ILQ--PESGAFN------LI-----SP----CHEF------KSFA-VA-ATLD-RTRLQAK-----------------------------------F : 206

fish_XP_004576250.1 : ----KMRPFDKNNFSTFYTE-NY-IFP--PATGPSN------LL-----DP----IHEY------QLLP-NA-TEAD-EREILCE-----------------------------------F : 268

fish_XP_005161888.1 : ----KPRPFEMKGGDFKYVK-NS-VLL--PESGVID------MI-----SP----CHEY------KSFA-IA-ATLD-RQRLQAK-----------------------------------L : 179

fish_XP_005478813.2 : QLNQKPEPTLQPITDKQKEA-TK--PT-EPGNAQAK------EI-----NR----PESY------ENTE-SKRDGTVEKDSKQIQLP------FHKTS----------------------E : 219

fish_XP_005806405.2 : ----EYRKFDQRPTNFRYVK-HN-SLP--PETGPEN------MI-----VP----CHEY------KSLE-TA-HKLD-SKRLKAK-----------------------------------V : 196

fish_XP_006625674.1 : ----SFYLFDSQHQTKRYIQ-NY-ILS--PETGPGN------RI-----EP----VHEY------KLLG-NVENAS--ETDILKK-----------------------------------F : 431

fish_XP_006635547.1 : ----KPRPLNKPGQDFTYAK-HN-VLQ--PESGVID------LM-----TP----CHEY------KAFT-TA-AGLD-RPRLQAK-----------------------------------F : 284

fish_XP_006635548.1 : ----EPRPFDTEDTVFKYVK-NR-VLP--PETGIKN------LI-----AP----CREY------KSLD-KA-VTLD-QTRLQAK-----------------------------------F : 191

fish_XP_006791552.1 : ----QPRPFDQEGTNFLYVK-HS-ILQ--PESGAFN------LI-----SP----CHEF------KSFA-VA-ATLD-RTRLQAK-----------------------------------F : 195

fish_XP_006791631.1 : ----DYRKFDQDEKDFRYVK-HH-VLP--PETGTEN------MM-----VP----CHEY------KSLE-IA-YKLE-SKRLQVK-----------------------------------V : 197

fish_XP_007241837.1 : ----KPRPFGTSSVDFTYVQ-HN-VLQ--PESGPID------LI-----TP----CHEY------KAFT-DA-ATLD-QQRLQAK-----------------------------------L : 186

fish_XP_007242299.1 : ----SFYPFDQQSAPHRYIE-NY-ILP--PELGPSN------LI-----DP----VHEY------KFLG-RAEDIHIMKKK---------------------------------------I : 315

fish_XP_007557414.1 : ----KPRPFDEDDIDFMYLK-HR-VLQ--PESGAFN------LI-----SP----CHEY------KSFA-IA-ATLD-RTRLQAK-----------------------------------F : 188

fish_XP_008284284.1 : ----KPYPFCRYHDTFRYLE-SS-ILD-ITESGASD------LI-----EP----CHEY------KGFY-NTP-----DDDKMSK-----------------------------------F : 153

fish_XP_008284762.1 : ----EYRKFDQNEKDCRYVK-HK-VLP--PETGTDN------ML-----VP----CHEY------KSLE-NA-HKLD-SKLLKVK-----------------------------------V : 167

fish_XP_008285761.1 : ----KPRPFDQEGIDFIYVK-HR-VLQ--PESGAFN------LT-----SP----CHEF------KSFS-VA-ASLD-RTRLQTK-----------------------------------F : 189

fish_XP_008336142.1 : ----RLKAEEVSGDCLVCFS-KQDLQA-LEIKGGPS-------------------VKIL------KELK-DLNSKPEPVLQPDPE-----------------------------------D : 164

fish_XP_008417602.1 : ----QIRPFDKSNSSIFYME-ND-ILP--PMAGPSN------LI-----DP----VHEY------HLVPNVNQAS---EKEILYE-----------------------------------F : 297

fish_XP_009510531.1 : ----MPYPFDNFSDGTRYIQ-YN-ILN-VPETGPLN------LI-----DP----AHEF------KLLT-NTEKAL--EEDIMMK-----------------------------------F : 220

fish_XP_010752845.1 : ----RLQEEDVSGDCLVWFK-KQDFLDLDIKSGPAV------KI-----------LAEL------RQLNKKPEPTLQPILHTGTD------------------------------------ : 170

fish_XP_010755274.1 : ---RKPYPFCRYHDTYRYME-NS-ILD-ITESGASD------LI-----EP----CHEY------KAFI-NTT-----DETKMSK-----------------------------------F : 152

fish_XP_010767524.1 : ----MLREEDVTGDCLVCFKKQDFLDL-KVKSGPAV-----------------------------KILA-EL-HKLNNKPEPQLQ-----------------------------------P : 162

fish_XP_010781233.1 : ----KPYPFCRQHDTHRYIE-SN-ILD-IPESGALD------LI-----EP----CHEY------KGFTNTT------DETKMSK-----------------------------------F : 173

fish_XP_010791966.1 : ----RPRPFDQEEIDFIYVK-NR-VLQ--PESGAFN------LT-----SP----CHEF------KSLS-KA-STLD-RTRLQAK-----------------------------------F : 133

fish_XP_012687177.1 : ----QPRPFDQNSPSFMYTQ-NE-LLP--EETGQTN------LM-----DP----IHEF------KVMV-NTENAT--EKEILHK-----------------------------------F : 136

fish_XP_012708385.1 : ----RARPFDKDP-DFIYVK-HR-VLQ--PESGAFN------LI-----NP----CREF------KSLA-NA-SQLD-NTRLRTK-----------------------------------F : 185

fish_XP_012713253.1 : ----KPYPFCRYHDTFRYV--HGSILD-VTESGASD------MI-----QP----CHEY------KAFINTP------DEAKMNK-----------------------------------F : 151

fish_XP_012727798.1 : ----EYRKFDQRPTNFRYVK-HN-LLP--PETGPEN------MV-----VP----CHEY------KSLE-TA-HKLD-SIRLKTK-----------------------------------V : 179

fish_XP_012990558.1 : FQKNDLLELGIKHGPAVKII---GMLE-KLNNEPEP------VL-----QP----ASSI---KVDQGQP-PLKQPLTKSLKTTSK-----------------------------------T : 185

fish_XP_013126906.1 : ----KPYPFCRYHDTFRYME--SSILD-VTESGASD------LI-----EP----CHEY------KAFTSTT------EETKMKK-----------------------------------F : 153

fish_XP_013796981.1 : ----MPHPFDNFRDGSRYTH-HN-ILN-IPETGPLN------LI-----DP----AHEF------KLLT-NT-EEAQ-EENIMMK-----------------------------------F : 221

fish_XP_014005049.1 : ----EPKPQAMGAE----------MLP-SKMPSSLT------LL-----Q--------------------NTLDDLQ-------------------------------------------- : 56

fish_XP_014049585.1 : ----KPRPFGKEGIDFTYVK-HS-VLQ--PESGVFD------LI-----SP----CHEY------KSLD-NA-AKLD-RTRLQAK-----------------------------------F : 189

fish_XP_014325953.1 : ------WLLDHLKIYIKYVE----LLL---------------------------------------------------DQDVSGD------------------------------------ : 118

fish_XP_015195770.1 : ----KPYPFQCFHDAYRYTE-NR-TLD-IPETGPSN------LI-----DP----CHEF------KAFI-HT-QEGS-TEDQMQK-----------------------------------F : 168

fish_XP_015197335.1 : ----LPYPFDKSHEPYTYSQ-HD-ILP--AEKGPSN------LI-----EP----VHEY------KLLG-NTEHAS--EAEVLKK-----------------------------------F : 232

fish_XP_015232478.1 : ----KPYPFGRYHDAFRYLK-DN-ILA-VPESGSFN------FI-----EP----CHEF------KGFY-----NTP-EDKKMEK-----------------------------------F : 82

fish_XP_015252023.1 : ----KPYPFCRYHDTFRYVE-GS-ILD-VTESGASD------LI-----EP----CHEY------KAFI-NAPD----DETRMTK-----------------------------------F : 146

fish_XP_015264177.1 : LRWTKCRAFNSNDINFKYVK-NT-VLP--PETGVLN------LI-----TP----CHEY------KSFT-TA-ASLD-RTRLQAK-----------------------------------F : 226

fish_XP_015801149.1 : ----KPRPFDEEGIDFIYLK-HR-VLQ--PESGAFN------LI-----SP----CHEY------KSFS-IA-AALD-PTRLRAK-----------------------------------F : 194

fish_XP_015810486.1 : ----KPYPFSRYQDTFRYIE-DS-ILD-VPESGASN------FI-----EP----CHEY------KGFI-NTP-----DEHKLNK-----------------------------------F : 151

fish_XP_016115105.1 : ----KPYPFSRFNAAQRYRE-NS-ILD-VTETGPKD------LI-----QP----CHEF------KAFV-NTA-----EEDRMKK-----------------------------------Y : 157

fish_XP_016117315.1 : ----SLYPFDHNAASHRYIQ-NY-TLP--PETGPDN------LI-----DP----VHEY------KFMG-RTDDIHTIKKK---------------------------------------F : 265

fish_XP_016395637.1 : ----KLRPFDMKGVDFTYVK-NS-VLL--PESGVVD------FI-----SP----CHEY------KSFA-IA-ATLD-RQRLQAK-----------------------------------F : 180

fish_XP_017207716.1 : ----SFYKFDQSSASHRYIQ-NY-VLP--PETGPDN------LI-----DP----VHEY-----------KFMGRTDDMTVMKKK-----------------------------------F : 250

fish_XP_017277259.1 : ----DYRKFDTREKNFRYVK-HN-LLP--PETGTDN------MI-----VP----CHEY------KSLE-VA-HKLD-SKRLKSK-----------------------------------V : 197

fish_XP_017277301.1 : ----KPRPFDEE-IDFIYVK-HR-VLQ--PESGAFN------LI-----SP----CHEY------KSLA-VA-ATLD-RTRLQAK-----------------------------------F : 194

fish_XP_017325460.1 : ----KPYPFNRFHAAYRYKE-NS-ILD-VIETGALN------LI-----EP----CHEY------KAFI-NF-QNVTTEEKLKK------------------------------------F : 175

fish_XP_017346953.1 : ----KPRPFGKTGIDYTYVK-HN-VLH--PETGIID------LI-----TP----CHEY------KAFT-TA-ATLD-RQRLQAK-----------------------------------L : 183

fish_XP_017550536.1 : ----PQRPFQRAERNNQMPSIFTLLLH----------------------------CLED---LTNEDFK-KF------RRYLKEP-----------------------------------I : 148

fish_XP_017550824.1 : ----KPYPFSQYHAAYRYQE-NS-ILD-VIETGALN------LI-----EP----CHEY------KAFI-NF-QNVTTEEKLKK------------------------------------F : 160

fish_XP_017554653.1 : ----SFCPFDQKSVTYRYIENYR--LP--PESGPSN------LV-----DP----VHEY-----------KFSGRAEDIDILKKK-----------------------------------I : 297

fish_XP_017569575.1 : ----KPRPFGKRGIDYTYVK-HG-VLQ--PESGVID------LI-----TP----CHEY------KAFT-IA-AALD-CQRVQAK-----------------------------------L : 189

fish_XP_018413083.1 : ----TPYPFDSTHESKRYTQ-HH-FLL--PEAGTSN------YI-----DP----VHEY------KEFT-NT-GKAT-EEDKKMK-----------------------------------F : 201

fish_XP_018429299.1 : ----HFRPFDKQVEGFKYFK-DQ-VLP--PETGVDD------LI-----TP----CHEY------KSL--VTAAKLD-RVRLQTK-----------------------------------F : 249

fish_XP_018521334.1 : ----QLHDEDVSGDCLVYFS-KQDFLDLDIKSGPAV------KI-----------LAEL------HQLNKKPEPTLHPTLHTTTD------------------------------------ : 171

fish_XP_018526467.1 : ----KPRPFDQEGIDYIYVK-HR-VLQ--PESGAFN------LI-----SP----CHEF------KSFA-IA-AALD-RGRLQAK-----------------------------------F : 197

fish_XP_018541041.1 : ----QIRPFDNRNSNIFYIK-NN-TLP--PVAGPSN------LL-----DP----VHEY------QLLP---SADETSEREILYE-----------------------------------F : 326

fish_XP_018558293.1 : ----KPYPFCRYHDADRYME-NS-ILD-ITESGASD------LI-----EP----CHEY------KAFINTT------DETKMSK-----------------------------------F : 165

fish_XP_018595329.1 : ----VPRSFDKDGRLLTYRE-ND-ILP--PETGPSN------LL-----DP----VHEY------KLLA-NTENAG--ETEILKK-----------------------------------F : 244

fish_XP_018611696.1 : ----KPRPFGNNGVDFTYVK-HN-VLF--PESGIID------LI-----SP----CHEY------KSFS-TA-ATLD-RQRLQAK-----------------------------------F : 198

fish_XP_018956725.1 : ----SLYPFDQNSASHRYIQ-NY-TLP--PETGPGN------LT-----DP----VHEY-----------KFMGRTDDIDVIKKK-----------------------------------F : 270

frog_XP_018091031.1 : ----SFRPFDKEVDNFKYVK-NN-VLS--PETGAID------LI-----EP----CHEY------KSLS-TA-AKLD-RLRLGTK-----------------------------------F : 314

Hemichordata_XP_006825609.1 : SLMQKPRLFDREVENCSYTK--GWILD-IIETGVID------IL-----QP----VHEY------KQFT--SYARSNPKERIWK------------------------------------F : 739

lance_EEN48978.1 : ------RPKGKIFVCGTRVK----LFR-TPDPVLVQ----------------------------------NTEKRQHGREMYFTK------------------------------------ : 91

lance_EEN55742.1 : GN--TSMPGSDNGGAAHITVYNFGNIQ-GSQIGHGGTLHNIQAR-----QT----SSKADSRLPSTPYF-NTNISADLLDKLLHK-----------------------------------P : 201

mammal_XP_001378512.1 : ----QTYPFDKFHKSFRYRE-QT-IIQ--PETGSFN------LI-----DP----IHEF------KALT-NT-ERAT-EEDILMK-----------------------------------F : 209

mammal_XP_003771993.1 : ----SPYPFDNFHKSFRYKE-RDVIIQ--PETGSLN------LI-----DP----AHEF------KALT-ET-EGAT-RKDIYMK-----------------------------------F : 209

mammal_XP_003771994.1 : ----LPYPFNSFHDTCRYIQ-HY-ILQ--PETGPLN------LI-----DP----VHEF------KLLT-NT-EVAT-EEDIKMK-----------------------------------F : 212

mammal_XP_003782717.1 : ----VSYPFDEFSNPYRYKF-HF-ILQ--PESGPGN------LI-----DP----VHEF------KAFT-NT-ETAS-VEDIKMK-----------------------------------F : 211

mammal_XP_003982796.1 : ----MPYPFDQFHNSQRYIE-HS-ILQ--PETGPLN------LI-----DP----IHEF------KALT-NT-EAAT-EKDIKMK-----------------------------------F : 217

mammal_XP_004389713.1 : ----MPYPFDQFHDGRRYIE-HY-MLQ--PETGPLN------LI-----DP----IHEF------KALT-NT-DMAT-EKDIQMK-----------------------------------F : 215

mammal_XP_004582357.1 : ----RTHPFNRFDELWRYKS-NF-ILQ--PETGPHN------LI-----DP----IHEF------KALT-NT-EKAT-EEDVKMK-----------------------------------F : 201

mammal_XP_004582358.1 : ----MPYPFDQFHASHRYIE-RH-LLQ--PETGPLN------LI-----DP----IHEF------KALT-NT-GTAT-EEDIKMK-----------------------------------F : 217

mammal_XP_004622892.1 : ----VPYPFDQFHDSRRYVE-HQ-ILQ--PETGPRN------LI-----DP----IHEY------KALT-NT-ESVE-EKDIKMK-----------------------------------F : 214

mammal_XP_004646313.1 : ----IPYPFDNFHDSQRYIE-HY-ILQ--PETGPLN------LI-----DP----IHEF------KALT-NT-ENAT-EKDIKMK-----------------------------------F : 217

mammal_XP_004702866.1 : ----MPYPFDQFHDSQRYTV-HC-ILP--PETGPLN------LI-----DP----VHEY------KALT-NT-DTAT-EKDMLMK-----------------------------------F : 215

mammal_XP_005340592.1 : ----MSYPFDHFHDSHRYIE-HY-ILQ--PETGPLN------LI-----DP----IHEF------KALT-NT-DTAT-EEDIKMK-----------------------------------F : 216

mammal_XP_005388084.1 : ----LPYPFDNFHDSKRYIE-HY-ILQ--PETGPLN------LI-----DP----IHEF------KALT-DT-EYAT-EEGLKMK-----------------------------------F : 217

mammal_XP_006163552.1 : ----MPYPFDQFHDSKRYIE-HY-ILQ--PETGPLN------LI-----DP----IHEF------KALT-NT-ETAT-EESIKMK-----------------------------------F : 217

mammal_XP_006779273.1 : ----MPYPFDQFHSSHRYIE-HY-LLI-QPETGPLN------LI-----DP----IHEF------KALI-NT-DTAT-EKDIKMK-----------------------------------F : 217

mammal_XP_006834357.1 : ----MPYPFDQFHAGQRYTE-HC-VLP--PETGPLN------LI-----DP----IHEY------KEFT-NT-DNAT-EKDSLMK-----------------------------------F : 211

mammal_XP_006891533.1 : ----LPYPFDKFHDSRRYIE-HY-ILQ--PETGPLN------LI-----DP----IHEF------KAFT-NT-STAT-AKDIQMK-----------------------------------F : 215

mammal_XP_007522555.1 : ----MPYPFDQFHASHHYTE-HY-ILQ--PETGPLN------LI-----DP----IHEF------KALT-NT-ETAT-EKDIKMK-----------------------------------F : 211

mammal_XP_007938002.1 : ----MPCPFDQFHDSQRYIE-HY-ILQ--PETGPLN------LI-----DP----IHEF------KLLT-NT-ETAT-EKDIHMK-----------------------------------F : 215

mammal_XP_008062458.1 : ----APYPFDESNNQYCYKL-GF-SLP--PETGPGN------LI-----DP----VREF------KSFT-NT-ETAT-EEDIKMK-----------------------------------F : 216

mammal_XP_008259950.1 : ----MPYPFDQFHDSQRYIE-HY-ILQ--PETGPLN------LI-----DP----IHEF------KALT-NT-AAAT-EEDIKMK-----------------------------------F : 216

mammal_XP_008524371.1 : ----MPYPFDQFHDSQRYIE-HY-ILQ--PETGPLN------LI-----DP----IHEF------KALT-NT-ETAT-EKDIKMK-----------------------------------F : 216

mammal_XP_008820771.1 : ----MPYPFDCFHASKHYIE-HS-ILQ-VAETGPLN------LI-----DP----IHEF------KALT-NT-ETAT-EQDIKMK-----------------------------------F : 216

mammal_XP_010601767.1 : ----MPYPFDNFHDSQRYIE-HY-ILQ--PETGPLN------LI-----DP----IHEF------KALT-NT-ENAT-EEDLKMK-----------------------------------F : 217

mammal_XP_011853657.1 : ----MPYPFDQFHDSHRYIE-HY-TLQ--PETGALN------LI-----DP----IHEF------KALT-NT-ETAT-EADIKMK-----------------------------------F : 218

mammal_XP_012791283.1 : ----IPYPFDEFSDPYRYKE-NF-VLQ--PETGPLN------LI-----DP----IHEF------KAFK-NI-EKGT-KEDIKIK-----------------------------------F : 212

mammal_XP_012881542.1 : ----MPYPFDQFHDSQRYIE-HY-ILQ--PETGPLN------LI-----DP----IHEF------KALT-NI-EMAT-EEDVKMK-----------------------------------F : 207

mammal_XP_012934202.1 : ----TPYPFNTFSDPYRYKL-HF-ILQ--PETGPQN------LI-----DP----VHEF------KALK-NT-ETAT-EEDIKMK-----------------------------------F : 215

mammal_XP_013013639.1 : ----PPYPFDNFHDGQRYIE-HY-ILQ--PETGPLN------LI-----DP----IHEF------KALT-NT-ENAT-EEDLKMK-----------------------------------F : 213

mammal_XP_016016798.1 : ----MPYPFDQFHDSQRYIE-RY-FIQ--PETGPLN------LI-----DP----IHEF------KALT-NT-ETAT-EKDIKMK-----------------------------------F : 217

mammal_XP_016281899.1 : ----MPYPFDSFHDSCRYIQ-HY-ILQ--PETGPLN------LI-----DP----VHEF------KALI-NT-ETAT-EEDIKMK-----------------------------------F : 203

mammal_XP_017199853.1 : ----SPKTELTDNEDERGDT----KEK-LPAKEPSC------RS-----HP----FNKF-------------------DEQWRYK------------------------------------ : 178

mammal_XP_017508280.1 : ----MPYPFDKFHESKRYIE-HY-ILQ--PETGPLN------LI-----DP----VHEF------KALT-NT-EAAS-DKDIRMK-----------------------------------F : 226

mollusk_EKC42356.1 : -------ASSEEHQGHKFIEILKSLES-KKEEMQGD------LQ-----ELEKSIHPKY------QEIASNISVQKDEIKENSQE-----------------------------------L : 147

mollusk_XP_011436570.1 : KFTEIFRNFDTLAKATDAYRKHA-VFR-TSSLFISN------LI-----EP----LHCF-------------RHRADHDPSSLKW-----------------------------------I : 396

mollusk_XP_011440496.1 : VQGKPPLVSDQLSVSANEASESDLGVT-DYETASSDFENIQQVL-----N-----VSEFSSLASNERCSVDEPDEIPWTEKIRTK-----------------------------------H : 234

mollusk_XP_013061423.1 : ----VLEMQNISGKIFLTMKEEDFDKV-LPKTSTYGVKKTLALL-----IT--ELSHKT---NVYRAFL-RRFDTEVTHTDKYTKGHCTDVSINIRGRKTLPVRNFYLIEDTDQENALIFI : 374

mollusk_XP_013061425.1 : ----------EVTHTDKYTKGH--CAD-VSVNIRSR------KT-----HP----VRNF------HLID-ESDKNMALEY----------------------------------------V : 150

Musmu_NP_034286.2 : ----TPYPFDSFCDVKQYIE-HS-ILR-VAETGPLN------LI-----DP----IHEF------KAFT-NT-KKAT-EEDIKMK-----------------------------------F : 213

rept_XP_008110733.2 : ----LPYPFDEFHDSHRYIE-HY-VLH-IPETGPLN------LI-----DP----VHEF------KLFT-NT-DNAT-EENLKMK-----------------------------------F : 226

rept_XP_008112638.1 : ----PFRPFKSTDINFKYVK-NS-VFA--PETGSHD------LI-----IP----CHEY------KSFD-TA-AMLD-RPRLQVK-----------------------------------F : 225

rept_XP_015264172.1 : ----LPYPFDDFHSGHRYIQ-QH-VLS-IPETGPLN------LI-----DP----AHEF------KLFT-DAIQKAT-KENLKFK-----------------------------------F : 247

shark_XP_007886940.1 : ----NPHPFDTEDAEFKYIR-NT-VLP--PETGSKD------LI-----TP----CHEY------KSFQ-KM-CLKD-KNQQRLK-----------------------------------F : 72

Strpu_XP_003727489.1 : ------FNSVHFFVRVKVGPDHDAFLR-ACADGTLSRGLTKLLV-----TP--------------ELEE-AAGVKLRVRLDVKEK------------------------------------ : 127

Strpu_XP_011667260.1 : DSLERLKTFSESCTPQLNLV-KDLKRS-IGRDGLYN------LN-----GM----THDS------IHFH-VKVYTIEGLHRLQSD-----------------------------------V : 244

turtle_XP_006139744.1 : ----LPYPFDEFHDSRRYIQ-HN-LLN-VPETGPLN------LI-----EP----AHEF------KLLT-NT-ENAT-EEEIKMK-----------------------------------F : 239

turtle_XP_007060369.1 : ----LPYPFDEFHDSRRYIQ-HY-ILN-VPETGPLN------LI-----EP----AHEF------KLFT-NT-ENAT-EEDIKLK-----------------------------------F : 239

turtle_XP_014430084.1 : ----RFRPFGTQDINFKYVK-NT-VLS--PETGVID------LI-----TP----CHEY------KSFV-IA-AELD-QPRLQAK-----------------------------------F : 254

Xenla_OCT63184.1 : ----------------------KTLLQ-ITVASESN------------------------------------------------------------------------------------V : 93

Xenla_XP_018122800.1 : ----TPYPFDCTHASKIYVQ-NH-FLP--PESGTSN------YI-----DP----VHEY------KEFT-KT-ENAT-ENDKKMK-----------------------------------F : 196

Xentr_XP_002943568.3 : LSSSIFRPFDKEVGNFKYVK-NN-VLS--PETGAID------LI-----QP----CHEY------KSFS-TA-AKLD-RLRIGTK-----------------------------------F : 401

Xentr_XP_012813408.1 : ----SFREFGMEDKTFTYMK-NQ-RLS--PETGVID------LI-----RP----CHEY------KSFA-TA-ATLD-KMRLQAK-----------------------------------F : 210

* 1100 * 1120 * 1140 * 1160 * 1180 * 1200 *

Homsa_NP_060124.2 : SNEVFRFAS------A-CM-------------------------------------NSRTNGTIH-----FGVKDK----P------------------------------------HGKI : 247

Homsa_NP_689916.2 : SNEVFRFAS------A-CM-------------------------------------NSRTNGTIH-----FGVKDK----P------------------------------------HGEI : 249

fish_XP_015241820.1 : ------------------------------------------------------------------------------------------------------------------------- : -

fish_XP_018543893.1 : ------------------------------------------------------------------------------------------------------------------------- : -

fish_CDQ83481.1 : ------------------------------------------------------------------------------------------------------------------------- : -

fish_CDQ89582.1 : ------------------------------------------------------------------------------------------------------------------------- : -

fish_XP_005809248.1 : ------------------------------------------------------------------------------------------------------------------------- : -

fish_XP_007231320.1 : ---------------------------------------------------------TRNDDSTT-------------------------------------------------------- : 12

fish_XP_007254667.1 : -----------------ML------------------------------------------------------------------------------------------------------ : 2

fish_XP_008277954.1 : ------------------------------------------------------------------------------------------------------------------------- : -

fish_XP_010746900.1 : ------------------------------------------------------------------------------------------------------------------------- : -

fish_XP_012685053.1 : ------------------------------------------------------------------------------------------------------------------------- : -

fish_XP_013123332.1 : ------------------------------------------------------------------------------------------------------------------------- : -

fish_XP_014011897.1 : ------------------------------------------------------------------------------------------------------------------------- : -

fish_XP_014011904.1 : ------------------------------------------------------------------------------------------------------------------------- : -

fish_XP_014264868.1 : ------------------------------------------------------------------------------------------------------------------------- : -

fish_XP_014264875.1 : ------------------------------------------------------------------------------------------------------------------------- : -

fish_XP_014854842.1 : ------------------------------------------------------------------------------------------------------------------------- : -

fish_XP_017578465.1 : ------------------------------------------------------------------------------------------------------------------------- : -

fish_XP_012713132.1 : ------------------------------------------------------------------------------------------------------------------------- : -

fish_XP_010787190.1 : AFEVLRFAC------A-CM-------------------------------------NVRSNGTIH-----FGITDK--NSG----------------K-----------------HRHGEI : 63

fish_XP_017319342.1 : ANEVLKFAS------G-CM-------------------------------------NMRSNGTIH-----FGVMDS-RDGS----------------G-----------------HVHGEI : 200

Alligator_KYO30992.1 : AYEVIRFAS------A-CM-------------------------------------NVRTNGTIH-----FGVTDRDEDKG----------------------------------WKHGQI : 237

Alligator_XP_006025954.1 : TNEVFRFAS------A-CM-------------------------------------NSRTNGTIH-----FGVLDN----P------------------------------------HGEI : 270

amphibia_XP_018413082.1 : CNEVFRFAA------A-CM-------------------------------------NSRTNGTIH-----FGVRDK----P------------------------------------HGEI : 237

bact_AGA70736.1 : --------------------------------------------------------EAYNNGQIN-------------------------------------------------------- : 74

bact_CUJ86040.1 : EDAIIIDFS-----------------------------------------------SYRKSEPFF-----FDVLYFELAAL----------------------------------LEQFKG : 193

bact_ESW58583.1 : KNKQFGVIE------I-PV-------------------------------------DSFVQGPFI-----VTLHPN---------------------------------------MAAGNI : 92

bact_GAC67715.1 : AHYFLKVPL-----------------------------------------------DGTLDGICH-----GDLHERNI-------------------------------------LVHGSR : 230

bact_KMO66808.1 : AAELLPNPI------SIIS-------------------------------------ELAKQDHAR-----VGVIAGLNHGD----------------------------LHTANILVQGST : 245

bact_KUO79116.1 : DGQFYPNPF-----------------------------------------------YYVENYKIL-----QGLLDGEFLTG----------------------------------KLHADL : 232

bact_KYG06363.1 : ADSYKGHVV------S-VL-------------------------------------RGPVHGDCH-----SGNVFVRA-------------------------------------SMNAEL : 236

bact_ODA70488.1 : HVGLSGRDL------A-CF-------------------------------------RGPAHGDLH--------------------------------------------------LRNVLV : 245

bact_OGP49286.1 : YKDSFYFSDIYLSESGLAI-------------------------------------GVVGLNSAF-----MSEGKGASEYG-------------------------------------HML : 212

bact_SCF05533.1 : GEDRLPVMA------G-LA-----------------------------------------HGDLH-----VGNLLV----------------------------------------ERSEI : 237

bact_SCX17622.1 : KNYLFRRYI-----------------------------------------------ELVADGNMT-------MTERVEKTN----------------------------------ALDSKR : 104

bact_SFJ80976.1 : AEECLRSAA------G-LF----------------------------------GGELHREDGHVF-----LDPRTVFERDA----------------GAAFP-------------VVHGTC : 219

bact_WP_006128369.1 : QDERLPLMY------GYCH-------------------------------------GDLHTGNLLVPAAELAGADSGYWIV-------------------------------DLDRAREGA : 286

bact_WP_006365791.1 : VKDVLAMAN------T----------------------------------------PRDVTAYIV-----FGVRWTPESGS--------------------------------------TV : 66

bact_WP_006849212.1 : IDTCLDYMI------E----------------------------------------YYNTSSEVV-----MHTADS--------------------------------------------- : 146

bact_WP_007897046.1 : ------------------------------------------------------------------------------------------------------------------------- : -

bact_WP_030194308.1 : TMPAFRGPA---------------------------------------------------HGDLH--------------------------------------------------LRNVLV : 245

bact_WP_030198971.1 : SGSAFAFST------Q-ML----------------------------PDPVSLLERDNEFSDRAE-----FVMLGYNHGDL----------------------------------HTRNIL : 242

bact_WP_030898614.1 : TWDGLDSAH------VLAI-------------------------------------LGRAHGDLHADNILLPMSPVADATA----------------------------------YRLIDL : 272

bact_WP_041219555.1 : --------------------------------------------------------EAYNNGQIN-------------------------------------------------------- : 79

bact_WP_050505446.1 : EDTHLPVLH------GICH-------------------------------------GDLHTGNLLVPRDEIEAADDAFWIV-------------------------------DMDQARRSV : 210

bact_WP_055538636.1 : AGTMFAFAT------Q-VL----------------------------PDPVSVLEQDNELSHEVE-----FVMRGYNHGDL----------------H-----------------TRNILL : 243

bact_WP_062134708.1 : ------------------------------------------------------------------------------------------------------------------------- : -

bact_WP_062799339.1 : PSKLLSIAR-----------------------------------------------KLRANFSPSEYRLFIGALAS------------------------------------------GDK : 157

bact_WP_066463897.1 : ---------------GTKN-------------------------------------NFLVSQIKH--------------------------------------------------FYQNEL : 124

bird_XP_002194798.1 : ASEVIRFAS------A-CM-------------------------------------NIRTNGTIH-----FGVMDS-VEDK----------------G-----------------WKHGQI : 244

bird_XP_005010984.1 : SNEVFRFAA------A-CM-------------------------------------NSRTNGTIH-----FGIHDN----P------------------------------------HGEI : 252

bird_XP_005505023.1 : SNEVFKFAA------A-CM-------------------------------------NSRTNGTIH-----FGVCDN----P------------------------------------HGEI : 253

bird_XP_008492486.1 : TNDTFKFAA------A-CM-------------------------------------NSRTNGTIH-----FGVRDN----P------------------------------------HGQI : 253

bird_XP_008628214.1 : SNEAFRFAA------A-CM-------------------------------------NSRTNGTIH-----FGVHDN----P------------------------------------HGKI : 252

bird_XP_009068034.1 : ASEVIRFAS------A-CM-------------------------------------NIRTNGTIH-----FGVMDS-VEDK----------------G-----------------WKHGQI : 242

bird_XP_009080343.1 : CNETFRFAA------A-CM-------------------------------------NSRTNGTIH-----FGVWDN----P------------------------------------QGKI : 253

bird_XP_009486154.1 : STEVFRFAA------A-CM-------------------------------------NSRTNGTIH-----FGVRDK----------------------------------------PHGEI : 253

bird_XP_009640122.1 : STETFRFAA------A-CM-------------------------------------NSRTNGTIH-----FGVHDK----------------------------------------PHGKI : 251

bird_XP_009672623.1 : SNEVFRFAA------A-CM-------------------------------------NSRTNGTIH-----FGVSNK----P------------------------------------HGEI : 241

bird_XP_009695665.1 : ACEVIRFAS------A-CM-------------------------------------NIRTNGTIH-----FGVMDR-VEDR----------------G-----------------WKHGQI : 390

bird_XP_009886214.1 : SNEVFRFAA------A-CM-------------------------------------NSRTNGTIH-----FGVRDN----------------------------------------PHGEI : 253

bird_XP_009934438.1 : SNEVFRFAA------A-CM-------------------------------------NCRTNGTIH-----FGVRDN----------------------------------------PHGEI : 247

bird_XP_009999877.1 : SNEVFRFAA------A-CM-------------------------------------NSRTNGTIH-----FGVHDN----P------------------------------------HGQI : 252

bird_XP_010119033.1 : SNEAFRFAA------A-CM-------------------------------------NSRTNGTIH-----FGVCDN----P------------------------------------HGEI : 251

bird_XP_010144931.1 : SNEVFRFAA------A-CM-------------------------------------NSRTNGTIH-----FGVRDK----P------------------------------------HGEI : 241

bird_XP_010222910.1 : SNEVFRFAA------A-CM-------------------------------------NSRTNGTIH-----FGVHDN----P------------------------------------HGQI : 242

bird_XP_013160560.1 : SNEVFRFAA------A-CM-------------------------------------NSRTNGTIH-----FGVHDN----P------------------------------------HGKI : 256

bird_XP_014795808.1 : SNDVFKFAA------A-CM-------------------------------------NSRTNGTIH-----FGVRDT----------------------------------------PHGQI : 253

bird_XP_015156587.1 : SNEVFRFAS------A-CM-------------------------------------NSRTNGTIH-----FGVRDN----------------------------------------PHGEI : 245

bird_XP_015709021.1 : SNEVFRFAS------A-CM-------------------------------------NSRTNGTIH-----FGVHDN----P------------------------------------HGEI : 257

brachiop_XP_013415988.1 : LSELESFVT--------CP----------------------------------SDVTSRDNTPLHFLSSSKGFPQRIDSEI--------------------MLVGSENWQKIQRKKRDNKT : 414

Cioin_XP_018668123.1 : SSNVLVLNQ-----------------------------------------------NLQEKHLKD--------------------------------------------------FKQKQV : 135

Cnidaria_EDO35430.1 : NKEVFEIIPGLPSYATARN-------------------------------------SIASPQCRC-----FVATNPNAPDS------------------------------------SGLD : 337

Cnidaria_KXJ09690.1 : PDDAKNLDK------G-IM-------------------------------------RLKHHELSR-----FNNGNINDWDV----------------------------------SLMTTV : 344

Cnidaria_KXJ11435.1 : PDDAKNLER------G-KM-------------------------------------KLKHHELAR-----FNTGNIYDWDV----------------------------------SLMTTV : 326

Cnidaria_KXJ17955.1 : MQILYDPPE------E-CT------------------------------------HNMNPESVSK-----MEKMAELGRDA-------------------------------DFVAAIGPI : 423

Cnidaria_KXJ23466.1 : NKEGISLKR------C-SV-----------------------------------SSGIPDGDNRL-----YAMHGDFPFIP----------------DVV---------------FGHGLV : 357

Cnidaria_XP_015751626.1 : NREVITIIP------G-LP-------------------------------EVAPRNKIMTPQTRC-----LVLGDFNNRNT----------------NGLKLVAAALANCRRRKHCIHLTV : 767

Cnidaria_XP_015751629.1 : NREVITIIP------G-LP-------------------------------EVAPRNKIMTPQTRC-----LVLGDFNNRNT----------------NGLKLVAAALANCRRRKHCIHLTV : 641

Cnidaria_XP_015758975.1 : NREVITIIP------GLPE--------------------------------VSPRNKIMTPQTRC-----LILGDFNNPNT----------------NGLKLVTAALAYCRQRELYIHLTV : 614

Cnidaria_XP_015766106.1 : PEEEFNTMW-----------------------------------------------PILSKNFVT-----LGVDEG--------------------------------------------- : 131

Cnidaria_XP_015766118.1 : VHEQVKRNCNRPDLEGARM-------------------------------------GNELSNTLA-----SGRLGETGDND-----------CTELSDSEDNTNVEMHQEGRNILCIFKLV : 298

Cnidaria_XP_015766124.1 : KSDAAAFDK------GDMN-------------------------------------RLHKDQEAV-----FNRGDINKWDF----------------------------------SLMTTV : 240

coelacanth_XP_006000513.2 : TDEVFRFAS------A-CM-------------------------------------NCRTNGTIH-----FGVEDTMEGFA------------------------------------HGEI : 174

fish_CDQ87765.1 : SNEVFRFSA------A-CM-------------------------------------NSRTNGTIH-----FGVTGGPR-------------------------------------HMHGQV : 492

fish_CDQ92295.1 : ASEVLRFAC------A-CM-------------------------------------NMRSNGTIH-----FGVMDK--VKG----------------I-----------------YKHGEI : 235

fish_KKF13953.1 : AKEVLKFAT------G-CM-------------------------------------NVRTNGTIH-----FGVMDS-KEDA----------------R-----------------HVHGEI : 230

fish_XP_004085798.2 : KKEVFCFSA------S-CM-------------------------------------NSRTNGTIH-----FGVDRLSDREE------------------------------------IGQV : 288

fish_XP_004552746.3 : KEAAKPTEP------G----------------------------------------NAQAKEINR-----PESYENTESKR---------------------------------------- : 198

fish_XP_004558390.1 : AKEVLKFAA------G-CM-------------------------------------NIRSNGTIH-----FGVMDS-KEDG----------------E-----------------YVHGEI : 244

fish_XP_004576250.1 : TREVFSFAA------S-CM-------------------------------------NSRTNGTIH-----FGINNQQG-------------------------------------PQHGQV : 303

fish_XP_005161888.1 : AREVLKFAT------G-CM-------------------------------------NVRTNGTIH-----FGVMDS-RGDA----------------G-----------------HVHGEI : 217

fish_XP_005478813.2 : KEEIQQPKP------Q-NT-------------------------------------RVRNKGAVV-----QTTVSP-----------------------------------------DGSR : 250

fish_XP_005806405.2 : ASEVLRFAC------A-CM-------------------------------------NMRANGTIH-----FGIMDN--VKS----------------T-----------------HKHGEI : 233

fish_XP_006625674.1 : SNEVFRFSA------A-CM-------------------------------------NSRTNGTIH-----FGVGDRPH-------------------------------------FHDGEI : 466

fish_XP_006635547.1 : AKEVLKFAC------G-CL-------------------------------------NVRSNGIIH-----FGVVDS-KDDN----------------D-----------------HVHGEI : 322

fish_XP_006635548.1 : AYEVIRFAA------G-CM-------------------------------------NIRTNGTIH-----FGVKDN--IRT----------------D-----------------YEHGEI : 228

fish_XP_006791552.1 : AKEVLKFAT------G-CM-------------------------------------NIRSNGTIH-----FGVMDS-KEDG----------------G-----------------YVHGEI : 233

fish_XP_006791631.1 : ASEVLRFAC------A-CM-------------------------------------NMRANGTIH-----FGIMDK--VKG----------------T-----------------HKHGEI : 234

fish_XP_007241837.1 : AKEVFKFAT------G-CM-------------------------------------NARSNGTIH-----FGVMDG-QDSS----------------C-----------------YVHGEI : 224

fish_XP_007242299.1 : NKEAFRFAA------G-CM-------------------------------------NARTNGTIH-----FGVADSKDSE-----------------------------------FSHGEI : 352

fish_XP_007557414.1 : AREVFKFAT------G-CM-------------------------------------NIRSNGTIH-----FGVMDS-KENE----------------G-----------------YVHGEI : 226

fish_XP_008284284.1 : TAEVIRFAA------A-CM-------------------------------------NSRTNGTIH-----FGIGDTPD-------------------------------------FSHGQV : 188

fish_XP_008284762.1 : AMEVLRFAC------A-CM-------------------------------------NMRANGTIH-----FGIMDK--VKG----------------S-----------------HKHGEI : 204

fish_XP_008285761.1 : AKEVLKFAT------G-CM-------------------------------------NIRSNGTIH-----FGVMDS-KEDA----------------G-----------------YVHGEI : 227

fish_XP_008336142.1 : NTDLQDLND------V-SV-------------------------------------TPKPNVSQH-----LATKKSDSMNQ---------------------------------------- : 196

fish_XP_008417602.1 : TREVFSFAA------S-CM-------------------------------------NSRTNGTIH-----FGVKGQQQEQQ------------------------------------QCHV : 333

fish_XP_009510531.1 : SNEVFRFAA------A-CM-------------------------------------NSRTNGTIH-----FGVRDN----------------------------------------PHGEI : 252

fish_XP_010752845.1 : QKEDPKPTQ--------CE-------------------------------------QSLMTATIQ-------------------------------------------------------- : 190

fish_XP_010755274.1 : TAEVIRFAA------A-CM-------------------------------------NSRTNGTIH-----FGIGDTPD-------------------------------------FIHGEV : 187

fish_XP_010767524.1 : ISYITDITE------K-PN-------------------------------------PTQPELSLP-----QAITTNQPESQ---------------------------------NKIESET : 201

fish_XP_010781233.1 : TAEVIRFAA------A-CM-------------------------------------NSRTNGTIH-----FGIGDEPD-------------------------------------FSHGQV : 208

fish_XP_010791966.1 : AKEVLKFGT------G-CM-------------------------------------NIRTNGTIH-----FGVMDS-KEDA----------------G-----------------YVHGEI : 171

fish_XP_012687177.1 : SFEVFRFAA------A-CM-------------------------------------NVRTNGTIH-----FGVGDTPH-------------------------------------YRHGQV : 171

fish_XP_012708385.1 : AREVLKFAT------G-CM-------------------------------------NVRTNGTIH-----FGVMDS-REDA----------------D-----------------YVHGEI : 223

fish_XP_012713253.1 : TSEVVRFAA------A-CM-------------------------------------NSRTNGTIH-----FGVGDKPD-------------------------------------FVHGQV : 186

fish_XP_012727798.1 : ASEVLRFAC------A-CM-------------------------------------NMRTNGTIH-----FGVMDN--VKG----------------T-----------------HRHGEI : 216

fish_XP_012990558.1 : SQPNIRELT------GTEV-----------------------------------TPNIEEPTQHL-----SAQGDN---------------------------------------ILLSKI : 221

fish_XP_013126906.1 : TSEVIRFAA------A-CM-------------------------------------NSRTNGTIH-----FGIGDKPD-------------------------------------FTHGQV : 188

fish_XP_013796981.1 : SNEVFRFAA------A-CM-------------------------------------NSRTNGTIH-----FGVRDN----P------------------------------------HGEI : 253

fish_XP_014005049.1 : QDEFKRFKS--------CL------------------------------------KDFKLNGCRS-------------------------------------------------------- : 77

fish_XP_014049585.1 : AKEVLKFGS------G-CM-------------------------------------NIRSNGTIH-----FGVMDS-RDDT----------------G-----------------YVHGEI : 227

fish_XP_014325953.1 : -----------------CL------------------------------------------------------------------------------------------------------ : 120

fish_XP_015195770.1 : TEETVRFSA------A-CM-------------------------------------NSRTNGTIH-----FGVGDR----P----------------E-----------------FAHGQI : 203

fish_XP_015197335.1 : SNEVFRFAA------A-CM-------------------------------------NSRTNGTIH-----FGVGDEPH-------------------------------------FTHGEV : 267

fish_XP_015232478.1 : ITEVIRFAA------A-CM-------------------------------------NSRTNGTIH-----FGIGDK----P----------------E-----------------FVHGQV : 117

fish_XP_015252023.1 : TSEVIRFAA------A-CM-------------------------------------NSRTNGTIH-----FGIGDKPD-------------------------------------FVHGQV : 181

fish_XP_015264177.1 : ACEVMKFAS------A-CM-------------------------------------NIRTNGTIH-----FGVKDTVEKKD----------------------------------WQHGQI : 264

fish_XP_015801149.1 : AREVLKFAT------G-CM-------------------------------------NIRSNGTIH-----FGVMDS-KEDA----------------G-----------------YVHGEI : 232

fish_XP_015810486.1 : TFEVIRFSA------A-CM-------------------------------------NSRTNGTIH-----FGIGDKPD-------------------------------------FVHGQV : 186

fish_XP_016115105.1 : TYEVIRFAA------A-CM-------------------------------------NSRTNGTIH-----FGVADN----------------------------------------PHGQI : 189

fish_XP_016117315.1 : NKEVFRFAA------G-CM-------------------------------------NSRTNGTIH-----FGVADSKDSQ-----------------------------------YTHAEI : 302

fish_XP_016395637.1 : AKEVLKFAT------G-CM-------------------------------------NVRTNGTIH-----FGVMDS-RGDT----------------G-----------------YVHGEI : 218

fish_XP_017207716.1 : NKEVFRFAA------G-CM-------------------------------------NSRTNGTIH-----FGVADSKDSH-----------------------------------YVHGEI : 287

fish_XP_017277259.1 : ASEVLRFAC------A-CM-------------------------------------NMRANGTIH-----FGVMDK--VKG----------------T-----------------HKHGEI : 234

fish_XP_017277301.1 : AREVLKFAT------G-CM-------------------------------------NIRSNGTIH-----FGVMDS-KEDA----------------E-----------------YVHGEI : 232

fish_XP_017325460.1 : TDEVIRFAC------A-CM-------------------------------------NSRTNGTIH-----FGVGDLQK-------------------------------------FSHGQI : 210

fish_XP_017346953.1 : AKEVLKFAM------G-CI-------------------------------------NIRSNGTIH-----FGIMDS-RENS----------------G-----------------YVHGEI : 221

fish_XP_017550536.1 : LNECNTIPT------C--------------------------------------------------------------------------------------------------------- : 158

fish_XP_017550824.1 : TEEVIRFAA------A-CM-------------------------------------NSRTNGTIH-----FGVGDRPD-------------------------------------FTHGQI : 195

fish_XP_017554653.1 : NKEVFRFAA------G-CM-------------------------------------NSRTNGTIH-----FGVADSKNSE-----------------------------------YCHGEI : 334

fish_XP_017569575.1 : AKEIFKFAT------G-CM-------------------------------------NMRSNGTIH-----FGVMDG-KDNS----------------G-----------------YVHGEI : 227

fish_XP_018413083.1 : CNEVFRFAA------A-CM-------------------------------------NARTNGTIH-----FGVRDK----P------------------------------------HGEI : 233

fish_XP_018429299.1 : ASEVLRFAC------A-CM-------------------------------------NMRTNGTIH-----FGVVDSVEDKA----------------------------------YRHGQI : 287

fish_XP_018521334.1 : QKEPKPIQP------ELSL--------------------------------------NQAKETKQ-------------------------------------------------------- : 192

fish_XP_018526467.1 : AKEVLKFAT------G-CM-------------------------------------NIRSNGTIH-----FGVMDS-KEDA----------------G-----------------YVHGEV : 235

fish_XP_018541041.1 : TKEVFSFAA------S-CM-------------------------------------NSRTNGTIH-----FGVHSQPG-------------------------------------QGHGEV : 361

fish_XP_018558293.1 : TDEVIRFAA------A-CM-------------------------------------NSRTNGTIH-----FGIGDEPD-------------------------------------FTHGQV : 200

fish_XP_018595329.1 : TDEAFRFGA------A-CM-------------------------------------NSRTNGTVH-----FGVGDKPQ-------------------------------------YEHGQI : 279

fish_XP_018611696.1 : AREVLKFGT------G-CM-------------------------------------NIRSNGTIH-----FGVMDS-REDA----------------G-----------------FVHGEI : 236

fish_XP_018956725.1 : NKEAFRFAA------G-CM-------------------------------------NSRTNGTIH-----FGVADSKDSQ-----------------------------------YAHAEI : 307

frog_XP_018091031.1 : AYEVLRFGC------A-CL-------------------------------------NTRTNGTIH-----FGIVDS-KEDK----------------S-----------------YQHGQI : 352

Hemichordata_XP_006825609.1 : VTETIRFAA------A-CL-------------------------------------NDRTNGTIH-----FGIADSKHGYA-----------------------------------QHGQI : 776

lance_EEN48978.1 : ---------------AEQQ-------------------------------------NARQETTES------SLCDD--------------------------------------------- : 109

lance_EEN55742.1 : AGPNILFRE------SEVV-------------------------------------YQGHEGQAAFMLDVVSMWNTPKRKE-----------------------------------RAFIV : 244

mammal_XP_001378512.1 : SNEVFRFAS------A-CM-------------------------------------NCRTNGTIH-----FGVKDS----------------------------------------PHGEI : 241

mammal_XP_003771993.1 : SNEVFRFAS------A-CM-------------------------------------NRRTNGTIH-----FGVKDK----------------------------------------PHGEI : 241

mammal_XP_003771994.1 : SNEVFRFAS------A-CM-------------------------------------NSRTNGTIH-----FGVKDK----P------------------------------------HGEI : 244

mammal_XP_003782717.1 : SNEVFRFAS------A-CM-------------------------------------NSRTNGTIH-----FGVKDK----P------------------------------------QGKI : 243

mammal_XP_003982796.1 : SNEVFRFAA------A-CI-------------------------------------NSRTNGTIH-----FGVKNK----------------------------------------PHGQI : 249

mammal_XP_004389713.1 : SNEVFRFAS------A-CM-------------------------------------NSRTNGTIH-----FGVKDK----P------------------------------------HGEI : 247

mammal_XP_004582357.1 : SNEVFEFAS------A-CM-------------------------------------NSRTNGTIH-----FGVKDK----P------------------------------------HGQI : 233

mammal_XP_004582358.1 : SNEVFRFAS------A-CM-------------------------------------NSRTNGTIH-----FGVRDN----P------------------------------------HGEI : 249

mammal_XP_004622892.1 : SNEVFRFAS------A-CM-------------------------------------NSRTNGTIH-----FGVLDK----P------------------------------------QGEI : 246

mammal_XP_004646313.1 : SNEVFKFAS------A-CM-------------------------------------NSRSNGTIH-----FGVKDK----P----------------H-----------------AEHGEI : 252

mammal_XP_004702866.1 : SNEVFRFAS------A-CM-------------------------------------NSRTNGTIH-----FGVQDK----P------------------------------------HGEI : 247

mammal_XP_005340592.1 : SNEVFRFAS------A-CM-------------------------------------NSRTNGTIH-----FGVKDK----P------------------------------------HGEI : 248

mammal_XP_005388084.1 : SNEAFRFAS------A-CM-------------------------------------NTRTNGTIH-----FGVKDK----P------------------------------------HGEI : 249

mammal_XP_006163552.1 : SNEVFRFAS------A-CM-------------------------------------NSRTNGTIH-----FGVLDK----P------------------------------------HGEI : 249

mammal_XP_006779273.1 : SNEVFRFAS------A-CM-------------------------------------NSRTNGTIH-----FGILDK----------------------------------------PHGEI : 249

mammal_XP_006834357.1 : SNEVFKFAS------A-CM-------------------------------------NSRTNGTIH-----FGVMDK----P------------------------------------HGEI : 243

mammal_XP_006891533.1 : SNEVFRFAS------A-CM-------------------------------------NVRTNGTIH-----LGVKDK----P------------------------------------HGEI : 247

mammal_XP_007522555.1 : SNEVFRFAS------A-CM-------------------------------------NSRTNGTIH-----FGVLDK----P------------------------------------HGEI : 243

mammal_XP_007938002.1 : SNEAFRFAS------A-CM-------------------------------------NSRTNGTIH-----FGVKDK----P------------------------------------HGEI : 247

mammal_XP_008062458.1 : SNEIFRFAS------A-CM-------------------------------------NSRTNGTIH-----LGVIDK----P------------------------------------HGKI : 248

mammal_XP_008259950.1 : SNEVFRFAS------A-CM-------------------------------------NSRTNGTIH-----FGVKDK----P------------------------------------HGEI : 248

mammal_XP_008524371.1 : SNEAFRFAS------A-CM-------------------------------------NSRTNGTIH-----FGVKNK----------------------------------------PHGEI : 248

mammal_XP_008820771.1 : SNEVFRFAS------A-CM-------------------------------------NSRTNGTIH-----FGVKDK----P------------------------------------HGEI : 248

mammal_XP_010601767.1 : SNEAFRFAS------A-CM-------------------------------------NSRTNGTIH-----FGVKDK----P------------------------------------HGEI : 249

mammal_XP_011853657.1 : SNEVFRFAS------A-CM-------------------------------------NSRTNGTIH-----FGVKDK----P------------------------------------HGEI : 250

mammal_XP_012791283.1 : SNEVFKFAS------A-CM-------------------------------------NSRTNGTIH-----FGVEDK----P------------------------------------HGKI : 244

mammal_XP_012881542.1 : TNEVFRFAS------A-CM-------------------------------------NSRTNGTIH-----FGVKDK----P------------------------------------HGEI : 239

mammal_XP_012934202.1 : TNETFRFAS------A-CM-------------------------------------NSRTNGTIH-----FGVEDK----P------------------------------------HGKI : 247

mammal_XP_013013639.1 : SNETFRFAS------A-CM-------------------------------------NSRTNGTIH-----FGVKDK----P------------------------------------HGEI : 245

mammal_XP_016016798.1 : SNEVFRFTS------A-CM-------------------------------------NSRTNGTIH-----FGVKDK----------------------------------------PHGEI : 249

mammal_XP_016281899.1 : SNEVFRFAS------A-CM-------------------------------------NSRTNGTIH-----FGVKDK----------------------------------------PHGEI : 235

mammal_XP_017199853.1 : LHFILQPET--------CM-------------------------------------NSRTNGTIH-----LGVKDK----------------------------------------PXGQI : 209

mammal_XP_017508280.1 : SNEVFRFAS------A-CM-------------------------------------NSRTNGTIH-----FGVLDK----P------------------------------------HGEI : 258

mollusk_EKC42356.1 : ASAIDKHGE--------DM-------------------------------------HREIDIIIAKLKSDLGEIESKHLTV---------------------------------LNEHEQE : 190

mollusk_XP_011436570.1 : AEDTVRFAC------A-CF-------------------------------------SDRTNGTIH-----FGISENLNKDG----------------------------------DLEGKV : 434

mollusk_XP_011440496.1 : HKKLLKRTS------S----------------------------------------DGDSIPTFYQNSSSQKIILKEKKKQ----------------------------------FTRSS- : 274

mollusk_XP_013061423.1 : SEEVVPFVS------A-CL-------------------------------------NDRRNGTIY-----FGISPAENEK-----------------------------------FSQGEI : 411

mollusk_XP_013061425.1 : AVETIPFVS------A-CL-------------------------------------NDRRNGTIY-----FGISPNSQNT-----------------------------------LKEGEI : 187

Musmu_NP_034286.2 : SNETFRFAA------A-CM-------------------------------------NSRTNGTIH-----FGVKDK----P------------------------------------HGEI : 245

rept_XP_008110733.2 : CNEVFRFAA------A-CM-------------------------------------NSRTNGTIH-----LGIRDN----P------------------------------------HGEI : 258

rept_XP_008112638.1 : AREVIKFAS------A-CL-------------------------------------NIRTNGTIH-----FGVMDRDKEKN----------------------------------RTHGQI : 263

rept_XP_015264172.1 : CNEVFRFAA------A-CM-------------------------------------NSRSNGTIH-----LGVLDK----P------------------------------------HGEI : 279

shark_XP_007886940.1 : GTDVMKFAA------A-CM-------------------------------------NTRTNGTIH-----FGVEDG---DG----------------D-----------------YVHGEI : 108

Strpu_XP_003727489.1 : PAEHSLIAV------K-SL-------------------------------------REMSPTDIQ-----IWLIEEVHVSI----------------------------------SIAAKF : 165

Strpu_XP_011667260.1 : QSGHIGHQL------G-KVLVSEKTRQQFLEDIILDVVLDGSGAWDLLSHAVIDPIPSSTSLSTD-----FEKREALKKDQTSSSDLMAQQGHPVVQAATVRKQVVNQEEGTSGLPAGSSP : 353

turtle_XP_006139744.1 : SKETFRFAA------A-CM-------------------------------------NSRTNGTIH-----FGVSNK----P------------------------------------HGEI : 271

turtle_XP_007060369.1 : SNETFRFAA------A-CM-------------------------------------NSRTNGTIH-----FGVSNK----P------------------------------------HGEI : 271

turtle_XP_014430084.1 : AFEVIRFAS------A-CM-------------------------------------NIRSNGTIH-----FGVMDRFEGDK---------------------------------DYEHGQI : 293

Xenla_OCT63184.1 : PNELLASKN----------------------------------------------------------------------------------------------------------IQQGES : 108

Xenla_XP_018122800.1 : CNEVFRFAA------A-CM-------------------------------------NVRTNGTIH-----FGVCDK----P------------------------------------HGKI : 228

Xentr_XP_002943568.3 : AYEVLRFGC------A-CL-------------------------------------NTRTNGTIH-----FGIMDSKEDKS----------------------------------YQHGQI : 439

Xentr_XP_012813408.1 : SAEVFKFAT------A-CL-------------------------------------NVRSNGTIH-----FGIMDS-VEDK----------------G-----------------YEHGQI : 248

1220 * 1240 * 1260 * 1280 * 1300 * 1320 *

Homsa_NP_060124.2 : VGI--------------------------------------------KVTNDTK-------------------------------------EALINHFNLM-----INKYFED-HQVQQAK : 281

Homsa_NP_689916.2 : VGV--------------------------------------------KITS--K-------------------------------------AAFIDHFNVM-----IKKYFEE-SEINEAK : 281

fish_XP_015241820.1 : ------------------------------------------------------------------------------------------------------------------------- : -

fish_XP_018543893.1 : ------------------------------------------------------------------------------------------------------------------------- : -

fish_CDQ83481.1 : ------------------------------------------------------------------------------------------------------------------------- : -

fish_CDQ89582.1 : ------------------------------------------------------------------------------------------------------------------------- : -

fish_XP_005809248.1 : ------------------------------------------------------------------------------------------------------------------------- : -

fish_XP_007231320.1 : ------------------------------------------------------------------------------------------------------------------------- : -

fish_XP_007254667.1 : ------------------------------------------------------------------------------------------------------------------------- : -

fish_XP_008277954.1 : ------------------------------------------------------------------------------------------------------------------------- : -

fish_XP_010746900.1 : ------------------------------------------------------------------------------------------------------------------------- : -

fish_XP_012685053.1 : ------------------------------------------------------------------------------------------------------------------------- : -

fish_XP_013123332.1 : ------------------------------------------------------------------------------------------------------------------------- : -

fish_XP_014011897.1 : ------------------------------------------------------------------------------------------------------------------------- : -

fish_XP_014011904.1 : ------------------------------------------------------------------------------------------------------------------------- : -

fish_XP_014264868.1 : ------------------------------------------------------------------------------------------------------------------------- : -

fish_XP_014264875.1 : ------------------------------------------------------------------------------------------------------------------------- : -

fish_XP_014854842.1 : ------------------------------------------------------------------------------------------------------------------------- : -

fish_XP_017578465.1 : ------------------------------------------------------------------------------------------------------------------------- : -

fish_XP_012713132.1 : ------------------------------------------------------------------------------------------------------------------------- : -

fish_XP_010787190.1 : IGF--------------------------------------------PVSS--P-------------------------------------EDFVDALD-Y-----IEKSFKGSDQQCDAR : 95

fish_XP_017319342.1 : IGI--------------------------------------------PIRE--K-------------------------------------DMYTDALD-H-----IEKCFK-SDSEL-VR : 230

Alligator_KYO30992.1 : AGV--------------------------------------------KVRD--R-------------------------------------DAYVDALD-Y-----IERCFDQSEQEA-AR : 268

Alligator_XP_006025954.1 : IGV--------------------------------------------EVRS--K-------------------------------------DTFIDYFNQA-----IKKYFGE-KYISEAK : 302

amphibia_XP_018413082.1 : IGV--------------------------------------------HIEN--V-------------------------------------DTYVNYIDQM-----ISKCFEE-KQLNLAK : 269

bact_AGA70736.1 : --------------------------------------------------------------------------------------------------------------WSEFQNNKKSI : 85

bact_CUJ86040.1 : VGI----------------------------------------------------------------------------------------EIWTDLVESL-----VEEVPDSTGRLQLER : 221

bact_ESW58583.1 : L---------------------------------------------------------------------------------------------------------IDKALYWRRSSKNSI : 108

bact_GAC67715.1 : QTS--------------------------------------------NLT-----------------------------------------YWLIDFNWDHRAPLLYDHAYLECSLILGGL : 266

bact_KMO66808.1 : LST--------------------------------------------QVH-----------------------------------------HWIIDANWADESPLLYDYSYLEVSLILDSL : 281

bact_KUO79116.1 : HLK----------------------------------------------------------------------------------------NLFISQFRNT-----TDFCLIDFDTYQDCN : 260

bact_KYG06363.1 : VAI--------------------------------------------SLIDL---------------------------------------GCFVADSMVF-----FDHAYFELSTLLHKL : 269

bact_ODA70488.1 : RGS--------------------------------------------RLTKDLA-------------------------------------YWLIDVNWDAPTPLLYDHAYLELSALLFGL : 285

bact_OGP49286.1 : LGI----------------------------------------------------------------------------------------RPLLKVFEQ------LENQASEKNRALDLT : 239

bact_SCF05533.1 : EGA--------------------------------------------GDDS----------------------------------------FWIVDVDQARISVAGIDLAYLELSVLINFY : 274

bact_SCX17622.1 : TRL----------------------------------------------------------------------------------------SDLIASFKTY-------------------- : 117

bact_SFJ80976.1 : HGD--------------------------------------------LHTDNLLLPRDELVASDDA-------------------------FWIVDVDQARR----SVAGFDLAYLEVSVL : 267

bact_WP_006128369.1 : IGF--------------------------------------------DLAYLEV-------------------------------------SVLVNLLPDLDPVV-LSRCLA--SAEGFSR : 323

bact_WP_006365791.1 : VGM----------------------------------------------------------------------------------------SCQLDDVE-------LQDAFGNGRVQPNPR : 92

bact_WP_006849212.1 : -----------------------------------------------SLDE----------------------------------------KTQADSCATF-----FVKAFNDLKTQVSEE : 175

bact_WP_007897046.1 : ------------------------------------------------------------------------------------------------------------------------- : -

bact_WP_030194308.1 : RGS--------------------------------------------RLTKDLA-------------------------------------YWLIDVNWDVPTPLLYDHAYLELSALLFGL : 285

bact_WP_030198971.1 : LDG--------------------------------------------ASADGVA-------------------------------------YWLIDIDWEHASPLLYDHAYLETATLLVGQ : 282

bact_WP_030898614.1 : SGY--------------------------------------------RADAPLS-------------------------------------RDLTHLTLAL-----ILRELPELTERQRSV : 307

bact_WP_041219555.1 : --------------------------------------------------------------------------------------------------------------WSEFQNNKKSI : 90

bact_WP_050505446.1 : VGF--------------------------------------------DLAYLEV-------------------------------------SVLVNILPT------IRRPVLARCLEAAED : 244

bact_WP_055538636.1 : DGV--------------------------------------------SADEVG--------------------------------------YWLIDIAWDGEAPLLYDHAYLETATLLVGQ : 282

bact_WP_062134708.1 : ------------------------------------------------------------------------------------------------------------------------- : -

bact_WP_062799339.1 : VVA----------------------------------------------------------------------------------------DINSIELERI-------RALGGDSLAVEME : 183

bact_WP_066463897.1 : DAN----------------------------------------------------------------------------------------DINLDTVAQY-------SYILNNGPKH--- : 147

bird_XP_002194798.1 : VGI--------------------------------------------KIKN--R-------------------------------------EDFVDALDYY-----IEKCFCNNLQEI-AK : 276

bird_XP_005010984.1 : KGI--------------------------------------------KVTK--K-------------------------------------EAYIDHFNRH-----IEKYFDK-QYIGAAK : 284

bird_XP_005505023.1 : EGV--------------------------------------------EVTK--K-------------------------------------EAYIDHFKKS-----IKKYFSE-PYTSIAK : 285

bird_XP_008492486.1 : EGI--------------------------------------------KVTS--R-------------------------------------NSYVDQLDKS-----FKDYFNK-DYVNIAR : 285

bird_XP_008628214.1 : EGI--------------------------------------------KVTN--K-------------------------------------EVYIKHFNIH-----IKKYFND-PYTIIAE : 284

bird_XP_009068034.1 : VGI--------------------------------------------KVER--R-------------------------------------EDYVDALD-Y-----IEKCFDDNCQEI-AR : 273

bird_XP_009080343.1 : EGI--------------------------------------------EVTN--Q-------------------------------------DAYIEHFNKD-----LGKYFDK-RFVSIAK : 285

bird_XP_009486154.1 : EGI--------------------------------------------KVTE--K-------------------------------------EAYVNHLNKL-----IGKYFNE-PYTSIAK : 285

bird_XP_009640122.1 : EGI--------------------------------------------EVTT--K-------------------------------------ETYVDHLNKM-----IGKYFHE-QYIDIAK : 283

bird_XP_009672623.1 : EGI--------------------------------------------KVTN--K-------------------------------------EAYIDHFNKY-----IGKYFSG-KYAEIAK : 273

bird_XP_009695665.1 : IGI--------------------------------------------KVKE--P-------------------------------------EYYVDALD-Y-----IEKCFCKNVQEI-AR : 421

bird_XP_009886214.1 : EGM--------------------------------------------KVTN--T-------------------------------------AAYVDHLNKY-----IGKYFHG-QYISIAK : 285

bird_XP_009934438.1 : EGI--------------------------------------------KITK--K-------------------------------------EAYIDHFNKS-----IRNYFSE-PYTSIAK : 279

bird_XP_009999877.1 : EGI--------------------------------------------QVTK--K-------------------------------------DAYVDHLNKS-----LRKYFNE-QHIDIAK : 284

bird_XP_010119033.1 : RGI--------------------------------------------KLTK--K-------------------------------------EAYVDHFNKF-----IDIYFKG-QYTGIAK : 283

bird_XP_010144931.1 : EGI--------------------------------------------KVTR--K-------------------------------------EAYVDHLNKL-----IGKYFNE-PYTSIAK : 273

bird_XP_010222910.1 : EGI--------------------------------------------KVTN--K-------------------------------------EVYIDHFNKY-----IGKYFGG--MSQIAK : 273

bird_XP_013160560.1 : EGI--------------------------------------------NVTK--K-------------------------------------EEYVDHLDKN-----IGKYFRK-EYITIAK : 288

bird_XP_014795808.1 : EGI--------------------------------------------KVTK--K-------------------------------------EAYINHFNTQ-----IGKYFHG-QYITIAK : 285

bird_XP_015156587.1 : EGI--------------------------------------------KVTK--Q-------------------------------------EAYIDHLNQY-----IEKYFVG-VYIRTAK : 277

bird_XP_015709021.1 : EGI--------------------------------------------KVTK--K-------------------------------------EAYIEHFNKY-----IQKYFVG-EYIGTAK : 289

brachiop_XP_013415988.1 : LGT--------------------------------------------QFPTLKQ-------------------------------------STENDQVYETEM---SNKIFESLCLNSSAS : 451

Cioin_XP_018668123.1 : QVV----------------------------------------------------------------------------------------DVKLDTIEGL-----IRKIFQ--------- : 154

Cnidaria_EDO35430.1 : IAV--------------------------------------------WALNNCR-------------------------------------RSKLKYKATL-----IVRNTNPSRTGEVEK : 372

Cnidaria_KXJ09690.1 : LLY-------------------------------------------------------------------------------------------------------SKECAKEIRLKPGYE : 362

Cnidaria_KXJ11435.1 : LLY--------------------------------------------SKEC----------------------------------------AKEIRLKPGY-----ENAIRTIKDQKNQLI : 358

Cnidaria_KXJ17955.1 : LTL-------------------------------------------EWINKLGR-------------------------------------DDVMELIPG------IPEGIQRNSIPSPSN : 458

Cnidaria_KXJ23466.1 : TGA--------------------------------------------EASGLAKKFNCKHFHICYDVNFTKEELQVGNDADFVAAIGPQLAKEWSNKLGRE-----VIEIIPGIPSDSRQR : 429

Cnidaria_XP_015751626.1 : VGT--------------------------------------------------D-------------------------------------REMVRELKEE-----ISNDLPYSRVQVEVK : 796

Cnidaria_XP_015751629.1 : VGT-----------------------------------------------D----------------------------------------REMVRELKEE-----ISNDLPYSRVQVEVK : 670

Cnidaria_XP_015758975.1 : VGT--------------------------------------------------D-------------------------------------REMVQELKEK-----ISNDLRHSRVNVEVK : 643

Cnidaria_XP_015766106.1 : -----------------------------------------------DVAD----------------------------------------ILHQSAVHIM-----FLLRYKPSSSLFALK : 160

Cnidaria_XP_015766118.1 : YGV--------------------------------------------GLQALR--------------------------------------DLFMQIHPKWTNQPSDAAALDKGRMRLDKN : 337

Cnidaria_XP_015766124.1 : LLY-----------------------------------------------------------------------------------------SQSCKLEIY-----KRPGYALALHELKNR : 267

coelacanth_XP_006000513.2 : LGV--------------------------------------------EVGD--P-------------------------------------SKYVDIIKQE-----ISKYFLE-KYDEDAK : 206

fish_CDQ87765.1 : IGQ--------------------------------------------NLPS--F-------------------------------------NMYTDRFDLH-----LKEHFG--EETNIAR : 523

fish_CDQ92295.1 : IGI--------------------------------------------PVEN--Q-------------------------------------DLFVDALD-Y-----IEKSFRGSTHQCDAR : 267

fish_KKF13953.1 : IGV--------------------------------------------PVKD--K-------------------------------------DIYVDALD-Y-----IERSFS-SDSDH-VR : 260

fish_XP_004085798.2 : VGQ--------------------------------------------KIRSF---------------------------------------RDYNQAFESS-----FGEFFEE-QHVDTAR : 320

fish_XP_004552746.3 : -------------------------------------------------------------------------------------------DGIVEKDSKQ-----IQLPFDKTSGKKEIQ : 223

fish_XP_004558390.1 : TGI--------------------------------------------PVTD--K-------------------------------------DIYVDALD-H-----IERSFS-SDKEH-IR : 274

fish_XP_004576250.1 : IGH--------------------------------------------TITSF---------------------------------------NKYIEAFELS-----VSKYFEE-QHVNTAR : 335

fish_XP_005161888.1 : VGF--------------------------------------------PVKE--K-------------------------------------DIYYDALD-Y-----IEKGFSESDSEL-VR : 248

fish_XP_005478813.2 : ITV--------------------------------------------EIKNILD-------------------------------------DLLKVDLKTF--------IFHLRSYTESKR : 282

fish_XP_005806405.2 : LGI--------------------------------------------PVQS--Q-------------------------------------EDFVDALD-Y-----IEKCFKGSNHQSDAR : 265

fish_XP_006625674.1 : IGI--------------------------------------------DVKD--R-------------------------------------NKYVDILNKS-----VQNYFG--ENAEDAK : 497

fish_XP_006635547.1 : IGV--------------------------------------------PVRE--R-------------------------------------DVYTDALD-H-----IEKCFPDSDSES-AR : 353

fish_XP_006635548.1 : IGL--------------------------------------------PVKD--R-------------------------------------DVFVDALD-Y-----TERCFANPSHKEDAR : 260

fish_XP_006791552.1 : TGI--------------------------------------------PVTD--K-------------------------------------DIYVDALD-Y-----IERSFS-SDTEH-IR : 263

fish_XP_006791631.1 : IGI--------------------------------------------PANN--R-------------------------------------EDFVDALD-Y-----IERCFKGSNQQSDAR : 266

fish_XP_007241837.1 : VGV--------------------------------------------PVRK--K-------------------------------------NLYADVLN-L-----IDRSFSSTDTEL-VQ : 255

fish_XP_007242299.1 : VGV--------------------------------------------SVEK--T-------------------------------------DTIIDHFNQG-----IKQYFE--DSADDAK : 383

fish_XP_007557414.1 : IGI--------------------------------------------PIKE--K-------------------------------------DIYVDALD-L-----SEKSFR-SDKEQ-VL : 256

fish_XP_008284284.1 : LGV--------------------------------------------VVQD--K-------------------------------------EAYAKELKKA-----IDGHFEH-KHKEAAQ : 220

fish_XP_008284762.1 : IGI--------------------------------------------PIRN--Q-------------------------------------EDFVDALD-Y-----IEKCFKGSNQQTDAR : 236

fish_XP_008285761.1 : VGV--------------------------------------------PVTE--K-------------------------------------DIYVDALD-Y-----IERSFS-SDKEH-VR : 257

fish_XP_008336142.1 : -----------------------------------------------PELN------------------------------------------TVPDKSSL-----SSKNFSSKAKEQQEE : 223

fish_XP_008417602.1 : VGQ--------------------------------------------RISSL---------------------------------------NKYNEAFESS-----LGDFFEE-EYVKVAR : 365

fish_XP_009510531.1 : EGI--------------------------------------------EVTK--K-------------------------------------EAYVDHFNKF-----IGKYFIS-NYTSIAK : 284

fish_XP_010752845.1 : -----------------------------------------------PESKT---------------------------------------EKKVENESEK-----VLQPFDKGGKEEIQP : 220

fish_XP_010755274.1 : LGV--------------------------------------------AVGD--K-------------------------------------EAYAKELKSA-----IDSHFEH-KHKQAAQ : 219

fish_XP_010767524.1 : DNV--------------------------------------------------A-------------------------------------EDKSENIQQQ-----FNKASEKDGIQHTNQ : 230

fish_XP_010781233.1 : LGV--------------------------------------------VVKD--K-------------------------------------EAYLKELKSA-----IDGYFEY-KHKQSAQ : 240

fish_XP_010791966.1 : IGI--------------------------------------------AVQE--K-------------------------------------DIYVDALD-Y-----IERSIS-CDKEH-VR : 201

fish_XP_012687177.1 : VGL--------------------------------------------KLTS--R-------------------------------------EKYIDEFDLQ-----LKKHFE--ENQSVAR : 202

fish_XP_012708385.1 : IGI--------------------------------------------PIKD--K-------------------------------------DVFVDALD-F-----IERSFS-SDKEH-IR : 253

fish_XP_012713253.1 : LGV--------------------------------------------AVDD--K-------------------------------------EAYANGLKSA-----IDGYFEH-KHKHAAQ : 218

fish_XP_012727798.1 : IGI--------------------------------------------PVQS--Q-------------------------------------ECFVDALD-Y-----IEKCFKGSTHQNDAR : 248

fish_XP_012990558.1 : SSM--------------------------------------------PSSL----------------------------------------TLLQNTLDDL-----QQKEFKR------FK : 247

fish_XP_013126906.1 : LGV--------------------------------------------VVED--R-------------------------------------EAFANELKSA-----IDEYFEY-KHKQAAQ : 220

fish_XP_013796981.1 : EGI--------------------------------------------KVTN--K-------------------------------------EAYIHHFNKY-----IGKYFPE-KYAEIAK : 285

fish_XP_014005049.1 : ----------------------------------------------------------------------------------------------------------IARCHLEDNKSRTDV : 92

fish_XP_014049585.1 : VGV--------------------------------------------PVSE--K-------------------------------------DVYVDALD-H-----IERSFSRSDSEH-VR : 258

fish_XP_014325953.1 : ------------------------------------------------------------------------------------------------------------------------- : -

fish_XP_015195770.1 : LGV--------------------------------------------RVPD--K-------------------------------------EAYVNELPRA-----IEGHFEH-KHVETAK : 235

fish_XP_015197335.1 : IGI--------------------------------------------SVPS--P-------------------------------------NKFVDHFNKS-----LKDYFT--EHTNIAK : 298

fish_XP_015232478.1 : LGV--------------------------------------------AVDD--K-------------------------------------EAYAKSLKEA-----IDGYFEH-KHKDDAQ : 149

fish_XP_015252023.1 : LGV--------------------------------------------AVGD--K-------------------------------------EAYANGQKSA-----IDAFFEH-KHKHTAE : 213

fish_XP_015264177.1 : VGI--------------------------------------------PVTD--R-------------------------------------DMYVDALD-Y-----IERCFEPYVQEV-AR : 295

fish_XP_015801149.1 : IGI--------------------------------------------HVEE--K-------------------------------------DIYVDALD-Y-----IERSFS-SDKEH-VR : 262

fish_XP_015810486.1 : LGV--------------------------------------------SVKD--K-------------------------------------EDYTTKLNSA-----ITAHFHF--NQDYAK : 217

fish_XP_016115105.1 : LGI--------------------------------------------CVQK--N-------------------------------------DDFDVQQSHA-----IEKNFKLERSVQMAK : 222

fish_XP_016117315.1 : IGV--------------------------------------------SVDD--K-------------------------------------DTIIDHFNQG-----IKSYFD--EHTDEAK : 333

fish_XP_016395637.1 : IGI--------------------------------------------PVKE--K-------------------------------------DVYCDALD-Y-----IERSFSSSEGEL-VR : 249

fish_XP_017207716.1 : IGI--------------------------------------------SVDK--K-------------------------------------DAIIDHFHQG-----IKQYFE--EQTQEAK : 318

fish_XP_017277259.1 : TGI--------------------------------------------PLKN--Q-------------------------------------EDFVDALD-Y-----IERCFKGSAHQIDAR : 266

fish_XP_017277301.1 : IGI--------------------------------------------PVND--R-------------------------------------AIYVDALD-Y-----IERSFS-TDREH-VR : 262

fish_XP_017325460.1 : LGA--------------------------------------------SVED--K-------------------------------------EAFAKKLAEV-----IKVRFEH-KHTDVAK : 242

fish_XP_017346953.1 : IGI--------------------------------------------PIKE--K-------------------------------------DMYVDALD-H-----IERSFSTSDSEL-VR : 252

fish_XP_017550536.1 : -----------------------------------------------QLEN----------------------------------------KERTDIAN-------LILQYHGEGNALKIT : 185

fish_XP_017550824.1 : LGT--------------------------------------------KIED--K-------------------------------------ETYNQKLADV-----IEGRFEY-KHINVVK : 227

fish_XP_017554653.1 : IGV--------------------------------------------SIDKT---------------------------------------DIIIDHFHQG-----IKSYFE--DNFDDAK : 365

fish_XP_017569575.1 : IGF--------------------------------------------PIQN--K-------------------------------------DTYADALN-L-----IDRCFSSANVEL-VQ : 258

fish_XP_018413083.1 : IGV--------------------------------------------HIAE--R-------------------------------------ELYGMYLYQM-----INKYFEE-KQFSIAK : 265

fish_XP_018429299.1 : TGI--------------------------------------------PVND--Q-------------------------------------DWYVDALDC------IEKCFTKVTSDV-AR : 318

fish_XP_018521334.1 : -----------------------------------------------QESN----------------------------------------DKTDSQTDEM-----ETQPFEKASERKIIQ : 221

fish_XP_018526467.1 : IGI--------------------------------------------PVKE--R-------------------------------------DIYVDALD-F-----IERSFS-SNKEQ-IR : 265

fish_XP_018541041.1 : VGH--------------------------------------------EITSF---------------------------------------SKYNEAFESC-----LSEYFEE-QHVNTAR : 393

fish_XP_018558293.1 : LGV--------------------------------------------VVKD--K-------------------------------------EAYANKLKSA-----IDQCFEY-KHKQTAQ : 232

fish_XP_018595329.1 : VGL--------------------------------------------RVPE--P-------------------------------------QKFVDRFYQQ-----LEKHFQ--KEAEDAR : 310

fish_XP_018611696.1 : IGL--------------------------------------------EVKD--K-------------------------------------DIYVDALD-Y-----TERCFSSSDSEH-VR : 267

fish_XP_018956725.1 : TGV--------------------------------------------SVDK--K-------------------------------------DTIIDHFNQG-----IKSYFD--EHTDEAK : 338

frog_XP_018091031.1 : VGI--------------------------------------------PVHD--K-------------------------------------DWFVDALD-C-----IEKVFTQND--A-AR : 381

Hemichordata_XP_006825609.1 : YGI--------------------------------------------EVLD--K-------------------------------------PEYEEGLDQF-----IGKCFQKNENIECAR : 809

lance_EEN48978.1 : -----------------------------------------------PPET----------------------------------------DSY------------MYLPFHKHPY----- : 126

lance_EEN55742.1 : MGV--------------------------------------------QCKCPPP-------------------------------------HDVIGLQDTG-----LDAYY---QNLFDTA : 276

mammal_XP_001378512.1 : VGL--------------------------------------------KLTEEDK-------------------------------------EKFVDHFYKK-----IEVYFEK-DDVSQAK : 275

mammal_XP_003771993.1 : VGV--------------------------------------------KVIEEDK-------------------------------------NKFVEHFYKK-----IEDYFSK-EEVEEAK : 275

mammal_XP_003771994.1 : VGM--------------------------------------------KITN--T-------------------------------------DAFIDHFNVK-----IKQYFRD-DQVNLAR : 276

mammal_XP_003782717.1 : VGV--------------------------------------------KFTSVTK-------------------------------------EALIDHFNLM-----IHQYFED-HQIQKAK : 277

mammal_XP_003982796.1 : VGV--------------------------------------------KVTS--K-------------------------------------DAFIDHFNIT-----IRQYFED--EVNEAK : 280

mammal_XP_004389713.1 : VGV--------------------------------------------KVTS--K-------------------------------------DAFIDHFNLM-----IKQYFEE-SHIKEAK : 279

mammal_XP_004582357.1 : VGV--------------------------------------------KFANITK-------------------------------------NALIDHFDRM-----IGKYFEE-HQVQQAK : 267

mammal_XP_004582358.1 : VGV--------------------------------------------NISS--K-------------------------------------DALIDHFNLM-----IKKYFED-NEVTIAK : 281

mammal_XP_004622892.1 : VGV--------------------------------------------KVTR--K-------------------------------------DSFIDHFNKM-----IKHYFED-SEIKEAR : 278

mammal_XP_004646313.1 : VGV--------------------------------------------KVTN--K-------------------------------------AAFIDHFNQM-----IKKYFEE-SDISEAK : 284

mammal_XP_004702866.1 : VGV--------------------------------------------RVTR--K-------------------------------------DAFIDHFNGM-----IKQYFEE-SHIKEAK : 279

mammal_XP_005340592.1 : TGV--------------------------------------------KVTS--K-------------------------------------DAFINHFNLM-----IKKYFEE-SEINEAK : 280

mammal_XP_005388084.1 : VGV--------------------------------------------KVTN--K-------------------------------------GAFIDHFNKM-----IKKYFEE-SEIHEAK : 281

mammal_XP_006163552.1 : VGV--------------------------------------------KVAN--K-------------------------------------NVFIDHFNLM-----IKKYFEE-SEINEAK : 281

mammal_XP_006779273.1 : VGM--------------------------------------------EVTS--K-------------------------------------DAFIDHFNKM-----IKQYFEE-SEINEAK : 281

mammal_XP_006834357.1 : VGV--------------------------------------------KITD--K-------------------------------------DAFINHFNLM-----IQKYFEK-SHIKEAK : 275

mammal_XP_006891533.1 : VGV--------------------------------------------SVTS--K-------------------------------------DAFIDHFNLM-----INQYFED-SHVKEAK : 279

mammal_XP_007522555.1 : VGV--------------------------------------------KVRS--K-------------------------------------DAFIDHFNLM-----IKQYFEE-SEIKEAK : 275

mammal_XP_007938002.1 : VGL--------------------------------------------KVTS--K-------------------------------------DGFIDHFNKI-----IKQYFEE-SDIKEAR : 279

mammal_XP_008062458.1 : VGV--------------------------------------------TLTRVTK-------------------------------------EALIDHFNQM-----THQYFEV-HQVQQAK : 282

mammal_XP_008259950.1 : VGV--------------------------------------------KISS--K-------------------------------------DVLINHFNVM-----IKKYFED-SDINEAK : 280

mammal_XP_008524371.1 : VGV--------------------------------------------KVTS--K-------------------------------------DSFIDHFNVM-----IKQYFEE-SEIKVAK : 280

mammal_XP_008820771.1 : VGV--------------------------------------------EITR--K-------------------------------------ESFIEHFNVM-----MKKYFEK-DDINEAK : 280

mammal_XP_010601767.1 : VGV--------------------------------------------KVTI--K-------------------------------------EAFIDHFNLM-----IKKYFKE-SEISKAK : 281

mammal_XP_011853657.1 : VGV--------------------------------------------KITS--K-------------------------------------DAFIDHFNLM-----IKKYFEE-SEINQAK : 282

mammal_XP_012791283.1 : IGV--------------------------------------------RFIAITK-------------------------------------EVLIDHFNQI-----IHQYFEE-GQVQKAK : 278

mammal_XP_012881542.1 : VGV--------------------------------------------KVAS--K-------------------------------------DVFIDHFNVM-----IKKYFEE-SEIQEAK : 271

mammal_XP_012934202.1 : VGI--------------------------------------------KVTSVTK-------------------------------------EALIDHFNWM-----IHQYFEA-HQVQQAK : 281

mammal_XP_013013639.1 : VGV--------------------------------------------KVTS--K-------------------------------------DAFIDHFNLM-----IKKYFEE-SEISEAQ : 277

mammal_XP_016016798.1 : VGV--------------------------------------------EVPS--K-------------------------------------DAFIDHFNLM-----IKQYFEE-SDINKAK : 281

mammal_XP_016281899.1 : IGM--------------------------------------------RITN--K-------------------------------------DAFVDHFNKM-----LRHYFDD-NHVNKAR : 267

mammal_XP_017199853.1 : VGV--------------------------------------------KLASITK-------------------------------------NALIDHFDEM-----IPKYFEEHQVQQ-AK : 243

mammal_XP_017508280.1 : IGV--------------------------------------------KVTS--K-------------------------------------DAFIDHFNIM-----IKQYFEE-SEINEVK : 290

mollusk_EKC42356.1 : ITH--------------------------------------------TIS-----------------------------------------EITQSILD-------LNKLLNSNDVSIISA : 219

mollusk_XP_011436570.1 : VGL--------------------------------------------QINQ----------------------------------------TKLLEIFYEE-----IRQSFYK-DQLSIVL : 465

mollusk_XP_011440496.1 : -------------------------------------------------------------------------------------------EKYFVQL--------LERGESKTVLIRNIH : 296

mollusk_XP_013061423.1 : VGV--------------------------------------------CLPK----------------------------------------EEVEAEIREA-----LSCSFTENQKEV-VL : 442

mollusk_XP_013061425.1 : VGV--------------------------------------------SIAK----------------------------------------EEVQARIRDA-----IKSSFTEIQKNI-LQ : 218

Musmu_NP_034286.2 : VGV--------------------------------------------QVTS--K-------------------------------------DIFVNHFNTM-----ITKYFED-SEISEAR : 277

rept_XP_008110733.2 : IGV--------------------------------------------KVQN--K-------------------------------------EKYINYFDLM-----INQYFV--NAVGVAK : 289

rept_XP_008112638.1 : IGI--------------------------------------------PIRE--T-------------------------------------EMYVDALD-Y-----IEKCFESSVHAT-AR : 294

rept_XP_015264172.1 : VGV--------------------------------------------KVES--K-------------------------------------DIYISFFNTM-----IQHYFG--RIVDVAK : 310

shark_XP_007886940.1 : VGV--------------------------------------------PVRD--P-------------------------------------ALFVQARDVY-----IQKCFMP-KYCFHAE : 140

Strpu_XP_003727489.1 : AGA--------------------------------------------GVDG----------------------------------------ELLIRYEEQH-----LQRDFQLPTGVMRKI : 197

Strpu_XP_011667260.1 : LATSGALASFAMIQDKCGPEDAVDKEKESLTSNIEFDLKEICDLLKGEASKEMK-------------------------------------DWTDKYVTFW-----LKNYVRLSDELVSKF : 432

turtle_XP_006139744.1 : IGV--------------------------------------------KLTN--K-------------------------------------DEFIDEFNNK-----IEKYFEK-SFVSIAK : 303

turtle_XP_007060369.1 : IGV--------------------------------------------KLSN--K-------------------------------------DEFIDDFNEK-----IEKYFDR-RFVRVAK : 303

turtle_XP_014430084.1 : IGV--------------------------------------------PVSN--R-------------------------------------DIYVDALDKN-----IAKCFSASDQEA-AR : 325

Xenla_OCT63184.1 : EGL---------------------------------------------MPHEGQ-------------------------------------DVPPAEKEHF-----ECSRFNSQNQEGPVG : 142

Xenla_XP_018122800.1 : IGI--------------------------------------------NIDN--R-------------------------------------EAYVSYIGHM-----MNKYFEE-KQLKTAK : 260

Xentr_XP_002943568.3 : VGI--------------------------------------------PVHD--K-------------------------------------DWFVDALDC------MEKAFAQNDKAV-AR : 470

Xentr_XP_012813408.1 : VGI--------------------------------------------AITQ--L-------------------------------------DWYVDALN-N-----MRECFKNPSEFAAAR : 280

1340 * 1360 * 1380 * 1400 * 1420 * 1440 *

Homsa_NP_060124.2 : KCI--REPR-----FVE-------VLLP-------NSTLSDRFVIE---VDIIPQ---FSECQY---DYFQ-IKMQNY--NNK-----------IWEQSK----KFSLFVRD--------- : 345

Homsa_NP_689916.2 : KCI--REPR-----FVE-------VLLQ-------NNTPSDRFVIE---VDTIPK---HSICND---KYFY-IQMQIC--KDK-----------IWKQNQ----NLSLFVRE--------- : 345

fish_XP_015241820.1 : ------------------------------------------------------------------------------------------------------------------------- : -

fish_XP_018543893.1 : ------------------------------------------------------------------------------------------------------------------------- : -

fish_CDQ83481.1 : ------------------------------------------------------------------------------------------------------------------------- : -

fish_CDQ89582.1 : ------------------------------------------------------------------------------------------------------------------------- : -

fish_XP_005809248.1 : ------------------------------------------------------------------------------------------------------------------------- : -

fish_XP_007231320.1 : ------------------------------------------------------------------------------------------------------------------------- : -

fish_XP_007254667.1 : ------------------------------------------------------------------------------------------------------------------------- : -

fish_XP_008277954.1 : ------------------------------------------------------------------------------------------------------------------------- : -

fish_XP_010746900.1 : ------------------------------------------------------------------------------------------------------------------------- : -

fish_XP_012685053.1 : ------------------------------------------------------------------------------------------------------------------------- : -

fish_XP_013123332.1 : ------------------------------------------------------------------------------------------------------------------------- : -

fish_XP_014011897.1 : ------------------------------------------------------------------------------------------------------------------------- : -

fish_XP_014011904.1 : ------------------------------------------------------------------------------------------------------------------------- : -

fish_XP_014264868.1 : ------------------------------------------------------------------------------------------------------------------------- : -

fish_XP_014264875.1 : ------------------------------------------------------------------------------------------------------------------------- : -

fish_XP_014854842.1 : ------------------------------------------------------------------------------------------------------------------------- : -

fish_XP_017578465.1 : ------------------------------------------------------------------------------------------------------------------------- : -

fish_XP_012713132.1 : ------------------------------------------------------------------------------------------------------------------------- : -

fish_XP_010787190.1 : SCI--RNPR-----FIE-------VLDKETSEETSEETSEKTWVIE---YDVVLK---ASIVKD---KLFY-VGLPKL--ADN-----------DKKNKL--E-KKVPFQRV--------- : 167

fish_XP_017319342.1 : QCI--RPPE-----FIL-------VIEP--------NRKEEHYVVE---FDVEPS---VNIVRN---KVFS-SSLPKF--SEE-----------SNKTAR--E-KPTIYHRV--------- : 294

Alligator_KYO30992.1 : LCI--HPPI-----FVE-------VIQK--------DSQEQTFVVE---VDIEPS---SSIANG---RVFQ-VRLPRF--NQD-----------NNKVII--EKHPVFYQRV--------- : 333

Alligator_XP_006025954.1 : ACI--REPR-----FVE-------VLLK-------NNTPSNTFVIE---VDIVPR---HSICEA---KFFQ-VQQYLF--KTE-----------TWEP--------ALFVRD--------- : 362

amphibia_XP_018413082.1 : QCI--RPPR-----FVC-------VLCQ-------DNTKSDLFVIE---VDVVPA---HAFCHS---QIFY-TYEQVF--KDN-----------SWKKSK----DKCCFVRD--------- : 333

bact_AGA70736.1 : ENM--QRPD-----LIE-------MLGK-------QDPHLHNISID---YEIINR------------NAFSITNRHKHELSNT---------------------QENSFRLV--------- : 140

bact_CUJ86040.1 : GWI--ADIAEARRFLIR--------------------KVSDGYPHD---QDALRL---QFLLGGVAAGLSFINKIPNNGEGSL--------GLDKNQYLQAYVWAAIFLKKY--------- : 297

bact_ESW58583.1 : ASH--EEMRGIYEWFLT-------------------------------------------------------------------------------------------------------- : 123

bact_GAC67715.1 : ERM--SNPV-----LAT-------VLGK-------IDGVEPAAPAP---LDLIAS---VLVHRI---KTIRAATEQELLRTEP--------------RRRDLWRRQIVLARI--------- : 334

bact_KMO66808.1 : SSS--THLE-----ILS-------VLRS------INKQNGNEHQFDLGHTDLISN---ICTIRDIVCGYLE-EHQQRRADVWQ---------------------QQLLLSRI--------- : 348

bact_KUO79116.1 : YLF--FDHA-----YLE-----FSVLQR-----LYEKNPIDDWILLLRHGEKLSP---LDMVRNEEYECLRGEQVRNSIAKGI------AVWSKSERSSTDAVQLQFLLAKV--------- : 346

bact_KYG06363.1 : ETI--GEER-----WGA-------LASA--------LTDDEVKLET---LDQIERGWAEDILRGRD-RLFA-RAFESWPDRRD-----------DLKIQFYSAHVSAGLAFL--------- : 343

bact_ODA70488.1 : GHS--TSGR-----VLP-------LLAR-------LDEQPLTVPVE---LDLNDS-GLITLLRRIRTETRS-VLATREQKRED-----------VWQ-------QQMLMARV--------- : 353

bact_OGP49286.1 : VALCHHPFE-----WLY---------------------EAERTVVN----QIVPKKTNILLEGHQNNPQHYFYSISPS--------------------------EKTLILQE--------- : 295

bact_SCF05533.1 : ADI--TLPV-----LSS-------CLDAAEAPTTQVIPDGHRWLVD---LLRSGRAGIGDWVNTQPGRSDQ-LRQQVMLTRMV---------------------AALQWARR--------- : 347

bact_SCX17622.1 : -----ASPS-----YLS-------------------------FEID---TDIL-----LNNSQNVNNDPKHSARIPDP----------------NKMRESIGELRKIIRNYI--------- : 170

bact_SFJ80976.1 : VNI--LPPK-----RLP-------VLAR--CLEAAEDPAVKSVPDD---CHWLVG---LLQRSRAGIQEWV-VRQPGRLDALY---------------------RQFMLARM--------- : 335

bact_WP_006128369.1 : RQI--PDGTDWLMAFLE-------ASRR---------GIEQWTSVQQGRRDHLER---QFMLIRMITGLLWAKRFEPGSPKARLCLAYAGCYAFHYDRVYGPGCLAPATESS--------- : 414

bact_WP_006365791.1 : FVY--TPIE-----FDG----------------------KQIGILE---IPVEKD------------GPYTPVKDYDG------------------------LQAGVVYYRR--------- : 136

bact_WP_006849212.1 : AAV-----------FFA-------SLDM---------------PIK---NEIKPD---INI-------------------------------------------EKAIHVSN--------- : 205

bact_WP_007897046.1 : ------------------------------------------------------------------------------------------------------------------------- : -

bact_WP_030194308.1 : GGS--TSGR-----VLP-------LLAR-------LEGEKLTVPVE---LDFNDS-GLVSLLRSIRGQTDD-VLMSREHKRVD-----------VWR-------RQLLMARV--------- : 353

bact_WP_030198971.1 : ERL--RKEL-----PLE-------ILRE-------CDDHDPRRAGP---LEWEGNVRLVESIRRGKDEAVE-ALQSARKGSLR---------------------HQYLVARL--------- : 348

bact_WP_030898614.1 : LAEFLVDPEADTDAVLH-------VVGL-GNLSNDIAQAGEVYARQLSMPDDWQDQYLLSLAGN---ALLFATRDTTVGLQER-----------AWCFQLACRSLGRFFSMH--------- : 397

bact_WP_041219555.1 : ENM--QRPD-----LIE-------MLGK-------QDPHLHNISID---YEIINR------------NAFSITNRHKHELSNT---------------------QENSFRLV--------- : 145

bact_WP_050505446.1 : TAV--RAVPDDCLWLVE-------FLQR-------TRAGIHEWIVEQP-GRMDPLYQQFMLARMAAALLWA-RRFPETDDRSR---------------------ICVAYAG---------- : 316

bact_WP_055538636.1 : ELL--RQQL-----PLE-------ILQD-------CDLRDPRRAVP---LVWEGNVRLVESIRRGKDETIE-ALQPARKGSLR---------------------HQYLVARL--------- : 348

bact_WP_062134708.1 : --------------LVQ-------------------------------------------------------------------------------------------------------- : 15

bact_WP_062799339.1 : GAA-----------VLS-------AIDR-------ADVTADHFVVR---------------------GISD-LISNKSETDAE-------------------------------------- : 219

bact_WP_066463897.1 : --------------YAE-------------------DPDKEKFEVI---AEIVKR-------------------------------------------------FILVFIDN--------- : 174

bird_XP_002194798.1 : KCI--HPPV-----FVE-------VISK--------DSQEQRFVVE---VDIEPT---FSLVKN---NCFE-VYLPKY--NED-----------SQKVTLT-K-EPALYQRL--------- : 341

bird_XP_005010984.1 : ACI--REPR-----FVE-------VLLP-------NETPSDTFVIE---IDVVPR---HSICNT---KYFC-TNIYDF--KSK-----------CFRK--------AVFIRN--------- : 344

bird_XP_005505023.1 : ACI--REPR-----FVE-------VLLQ-------NGSPSHRFVIE---IDVVPK---HCLCDT---KYFC-TNTYEY--MSK-----------SEKK--------AVFIRD--------- : 345

bird_XP_008492486.1 : DCI--RQPR-----FVE-------VLLQ-------NGDPSHTFVIE---VDVVPK---YHKCDT---KYFS-TRTYSY--KKK-----------CWQK--------TVFIRD--------- : 345

bird_XP_008628214.1 : YCI--RPPR-----FVE-------VLLQ-------NGTSADLFVIE---IDVVPQ---HSFCDT---KYFC-TNTYEY--KSK-----------TWKK--------AIFIRD--------- : 344

bird_XP_009068034.1 : KCI--HPPV-----FVE-------VISK--------GSKEQRFVVE---VDIEPT---SSLVRN---CCFE-VFLPKY--NEG-----------SQKVTLT-K-DLVMYQRL--------- : 338

bird_XP_009080343.1 : ACI--REPR-----FVE-------VLSP-------NGTSSHRFVIE---VDVVPQ---HCICDT---KYFI-TFTYKF--KSK-----------TWEK--------AVFIRD--------- : 345

bird_XP_009486154.1 : ACI--REPR-----FVE-------VLLP-------NGIPSHRFVIE---IDVVPK---HCICDT---KYFC-TNTYEYKSKGW---------------------KKAVFIRD--------- : 345

bird_XP_009640122.1 : ACI--KGPR-----FVE-------VLLP-------NGMQSHKFVIE---VDVIPK---HCKCNA---KYFC-TKTYEYKTKSW---------------------KKAVFIRD--------- : 343

bird_XP_009672623.1 : ACI--REPR-----FVE-------ILSQ-------NGTLSDTFIIE---IDVVPK---HSLCEA---KYFL-INTYEY--SSK-----------TWKQ--------AFFIRD--------- : 333

bird_XP_009695665.1 : KCI--HPPV-----FVE-------VISK--------DSQEQRFVVE---VDIEPT---SNLVKN---RVFE-VYLPKY--NEG-----------SQKVTLT-K-DRALYQRL--------- : 486

bird_XP_009886214.1 : DCI--REPR-----FVE-------VLLE-------NSTPSHKFVIE---IDVVPK---HSICEA---KYFC-TNTYEYKTKSW---------------------KKAVFIRD--------- : 345

bird_XP_009934438.1 : ACI--REPR-----FVE-------VLLQ-------NGHPSQMFVIE---IDVVPK---HCICDT---KYFC-TNTYEYKSKSW---------------------KKAVFVRD--------- : 339

bird_XP_009999877.1 : DCI--RQPR-----FVE-------VLLQ-------DGTSSHKFVIE---IDVVPK---YCKCDA---KYFI-TNTYEI--KSK-----------SWKK--------AVYIRD--------- : 344

bird_XP_010119033.1 : ACI--REPR-----FVE-------VLLQ-------DGSPSHKFVIE---IDVVPK---HCICST---KYFC-TNTYEY--SRK-----------SWEK--------AVFIRH--------- : 343

bird_XP_010144931.1 : SCI--REPR-----FVE-------VLLQ-------NGTPSHRFVIE---IDVVPQ---HCFCDT---KYFC-TNTYDY--ESK-----------TWTK--------AVFIRD--------- : 333

bird_XP_010222910.1 : ACI--RQPR-----FVE-------VLLQ-------NGTLSDTFVIE---IDIVPE---HCLCDK---KYFV-TDTYVY--KSK-----------DWNK--------AFFIRD--------- : 333

bird_XP_013160560.1 : ACI--RKPR-----FVE-------VLSQ-------NGASSHLFVIE---VDVVPR---HDICNA---KYFC-TNTYVF--KSD-----------TWKK--------AVYIRD--------- : 348

bird_XP_014795808.1 : ACI--REPR-----FVE-------VLLR-------NGAPSHLFVIE---IDVIPQ---HHICGT---NYFS-TKTYDFKTKSW---------------------EKAVFIRD--------- : 345

bird_XP_015156587.1 : ACI--KGPR-----FVE-------VLLP-------NGTPAGTFVIE---VDVIPH---SSICDT--------------------------------------------------------- : 314

bird_XP_015709021.1 : ACI--RDPR-----FVE-------VLLP-------NGTPSDMFVIE---VDVVPQ---YSICHT---KYFS-TNIYDF--KSN-----------GWKK--------AVFIRD--------- : 349

brachiop_XP_013415988.1 : EHLEFKPSDEV---YFCRDKGKASFMEDIVSFWNTPGREGNAYIIIGIEKCSHPPHKLIGIGDGRIDADYQNLFLDEFFTSRP-----------KFRYIQHEYDKMLFGIVI--------- : 549

Cioin_XP_018668123.1 : --------------FLE-----------------------------------------TTVNQN--------------------------------------------------------- : 163

Cnidaria_EDO35430.1 : KVL--K--------ILE-------QHDKEGVELNFKKASESRQVL----VDIQGSTMFLLPARR---SPFGVAALDAIATGIPVLISSQSGLAYLIRQYFDEKYHSCILDVK--------- : 460

Cnidaria_KXJ09690.1 : NAI--RTVKEQKNQLIS-------HMST-------EQMTDDEFNTA---WEKITKSLETIGASEGDIDEIL-TGDNLESAKFY-----------RDMLLQEMALKDAVLHDI--------- : 443

Cnidaria_KXJ11435.1 : SHV--RSER-----MTDDDFNAAWKKVQTSLITLKANAKHIEEIFE---DDVLES---AKYYRD---MFLQ-ELATNGAVQSE---------------------VRSVYTTV--------- : 432

Cnidaria_KXJ17955.1 : RCL-----------VVD-------SMTK--------ERDSGLQLSKKALSDCKKRVRPIKVTVQGTTRALKDELKQQFKGEAARSXFDIHVTSSEASLESVRGEISAASVLIMPEXSEMFG : 553

Cnidaria_KXJ23466.1 : NSI--PTPHNRALIVDSTASTDNDVLDVAIKALLDCKQRDTSMEIN---IQGTPRAKKDNLKTKLERELGT-KKFKIHITSDDASIELVDGDMRANSLLLIPSLSQTFGMRSLESISSGVP : 544

Cnidaria_XP_015751626.1 : RFD--LSQE-----AIG-------VEIRGVSLVLIPSSADGFGVLS---LEAMAAKVPVLIASSSGVATFL-HKEWEQQFLKL-----------LCSTDSDPDCEEAWKNAI--------- : 879

Cnidaria_XP_015751629.1 : RFD--LSQE-----AIG-------VEIRGVSLVLIPSSADGFGVLS---LEAMAAKVPVLIASSSGVATFL-HKEWEQQFLKL-----------LCSTDSDPDCEEAWKNAI--------- : 753

Cnidaria_XP_015758975.1 : RFD--LSQE-----AIG-------VEIRGVSLVLMPSLSDGFGVLS---LPAVAAKVPILIASSSGVAKFL-RKEFGQQFLKL-----------LCSTGSDTDCEEAWKNAI--------- : 726

Cnidaria_XP_015766106.1 : ICL--RPPS---------------------------------------------------------------------------------------------------------------- : 167

Cnidaria_XP_015766118.1 : EEVIFNKGDINQWDFSL-------ITST--------LLFSKICAIE---ISKKPG--CDTALQELKKTRNK-LLGHPCTDRMS-----------NTDFNIFWPQLCNNFVAL--------- : 417

Cnidaria_XP_015766124.1 : RNELLGHPS------TE-------------------EMSDDDFEIL---WPILSQ--SLVKLGADEGDIANILHYSDEVLSSG-----------EYKKLFLTDRKNQPFVMK--------- : 338

coelacanth_XP_006000513.2 : KCI--RPPR-----FVE-------VLNH-------DQTSLERHVIE---VDVVPD---YSLCLE---KVYY-IQTK----VKR-----------NRKMII----DRCLFIRD--------- : 268

fish_CDQ87765.1 : SCI--RPPK-----FFQ-------VQRL-------DGATSDKWVIE---VDVVPS---YSQTQE---KLFY-TSIISEQNGEE-------------------YKTECLFIRQ--------- : 585

fish_CDQ92295.1 : KCI--RNPK-----FIE-------VIDK--------EASETTWIIE---YDVVPK---AKMVKD---KLYS-VTIPKF--SEK-----------SNKVIY--E-EKATYHRV--------- : 331

fish_KKF13953.1 : LCV--RPPR-----FIE-------VMDQ--------DSTEKRYVVE---VDIVPS---ISIVKS---KVYA-VRLPNF--KES-----------TNKVEF--E-KEAILRRV--------- : 324

fish_XP_004085798.2 : MCI--RVPH-----FIQ-------VCHR-------DGTTSDRWVIE---VDVVPV---YSQTLD---TIFY-AQLGIMAADES-----------------QHCKTECLFIRD--------- : 384

fish_XP_004552746.3 : Q----PKPQ-----NTG-------VWNKGTVVQTTVSPDASRITVE---IKNVLD---DLLKVDLKTFIFH-LRLHTESKRKP---------ICQSKLEDKDSRDIATLMTD--------- : 303

fish_XP_004558390.1 : QCV--RPPR-----FIE-------VIDR--------QSTEKRYVVE---VDIVPL---ISIVKN---KVYA-VRLPNF--KES-----------ANKVEY--E-KEMILRRV--------- : 338

fish_XP_004576250.1 : MCV--RPPN-----FIQ-------VCYQ-------DGTTSDKWVVE---VDVVPT---YSETQH---ILFY-TQLSITTAEAS-------------QQCK----TECLFVRD--------- : 399

fish_XP_005161888.1 : LCI--GEPQ-----FVQ-------VICS--------DSSEEHFIVE---VDIKPS---VRIVKN---QVFS-VRLPNF--SEE-----------ANKVQL--E-KKTVYRRV--------- : 312

fish_XP_005478813.2 : KPI--SQSK-----LEDKDSMDIAKLMINHYGNEEAPQVAIQILKE---INQLPL---ALQLEE---RIRG-CREPQPCPPGHEAG--------MEEIQAPDIQRPQSVRAQ--------- : 369

fish_XP_005806405.2 : KCI--RNPC-----FIE-------VLDK--------ENAEKTWVVE---YDIVPK---ASIVKD---KLYS-VGVPKF--SEK-----------DGKVKC--E-EQIPYHRV--------- : 329

fish_XP_006625674.1 : MCI--RQPR-----FVQ-------VLSP-------DNTLLNKYVIE---VDVVPS---YSVCKG---KEYY-INMMLL--EDN-----------RWMKSK----EIYLFVRD--------- : 561

fish_XP_006635547.1 : ECI--RPPQ-----FVE-------VIDM--------SGTEKLYVVE---VDIVPT---ASIVKG---KVYT-VCLPNF--NEK-----------ANKVQK--E-KSTIYRRV--------- : 417

fish_XP_006635548.1 : QCI--RPPR-----FIE-------VIDK--------GSTETSWVIE---VDVVPM---AKTVKG---QVYT-VRLPKF--NEK-----------TNKVEF--E-KKTVYQRV--------- : 324

fish_XP_006791552.1 : LCV--RPPR-----FIE-------VMDR--------QSTEKRYVVE---VDIVPL---ISIVKN---KVYA-VRLPNF--KES-----------TNKVEF--E-KEVILRRV--------- : 327

fish_XP_006791631.1 : SCI--RHPR-----FIE-------VLDK--------ESSGNTWVIE---YDIVPK---ASIVKD---KLYY-VGVPKF--SEK-----------DNKVKC--E-EKVPYYRV--------- : 330

fish_XP_007241837.1 : QCI--RPPE-----FIP-------LTHT--------DNEEERYVVE---FDVVPS---VSIVRN---KMFG-VRLPNF--REE-----------SNKLEF--E-KETIFCRQ--------- : 319

fish_XP_007242299.1 : KCI--RQPR-----FVQ-------VLCC-------DNTSSSKYVIE---VDVVPS---HIVVHE---KIFY-TQTLD---EGN-----------QWKKCK----GKSLFIRE--------- : 446

fish_XP_007557414.1 : QCV--RPPR-----FIE-------VMDR--------ESTEKRYVVE---VDIVPL---LTIVKN---KVYS-VRLPNF--KES-----------SNKLEF--E-KEVIFRRV--------- : 320

fish_XP_008284284.1 : KCI--KPPR-----FVE-------VLNK-------NMTSSDKFVIE---VDIVPE---STICEENLYHTYT-VDTRKA--RKK-----------SKVKEMESQPSKCFLVRD--------- : 291

fish_XP_008284762.1 : SCI--RNPR-----FIE-------VLDK--------ETPEKTWVIE---YDVVPK---ATTVKG---KIYS-VGVPRP--TDK-----------D-KVKC--E-EKVPYYRA--------- : 299

fish_XP_008285761.1 : QCV--RPPR-----FIE-------VMDQ--------QSTERRYVVE---VDIVPS---ISIVRS---KVYA-VRLPNF--KES-----------TNKVEF--E-KEAILRRV--------- : 321

fish_XP_008336142.1 : QMS--RPQA-----LGA-------RPKE--------RVETEPQLIS---KNIIQDHLDHLISSDF--EKFK-FRLQSYKKSNQ---------------------KPIPKGKL--------- : 286

fish_XP_008417602.1 : LCI--RPPN-----FIP-------VLHE-------NGTASDKWVIE---VDVVPA-----ISQTQDYLFYTELGITSSEEKHQ-------------------CKTLCLFVRE--------- : 429

fish_XP_009510531.1 : ACI--RQPR-----FVE-------VLLQ-------NGTPSHRFVIE---IDVVPS---HCHCGT---KYFC-TNTYEYDSKCW---------------------IKTVFIRD--------- : 344

fish_XP_010752845.1 : AKTQNLGAR-----RKE-------TVRL-------RTVPPDESRIT---VEIQKT---LEDLSKEDLKRFH-FNLKAYTESKY---------------------GPISLSRL--------- : 285

fish_XP_010755274.1 : ICI--KPPR-----FVE-------VLKK-------IMTSTDKCVIE---VDIVPD---SSICEE---NVYH-TLNTKKNKKKN-----------KGQEETETQPLKQFFVRD--------- : 289

fish_XP_010767524.1 : KDL--GERR------KE-------MIGT--------TVQEDKQNIT---LEIKKI---LDDLSKDGLKEFN-FYEFTKSTNQS-------------------IPRGKLEGKD--------- : 293

fish_XP_010781233.1 : NCI--KPPR-----FVE-------VLNK-------NMTSSDKCVIE---VDIVPD---CMVCEESIYHTFN-RNIKKAKKKGK----------GKETDENETKPYKQLFVRD--------- : 314

fish_XP_010791966.1 : QCV--RPPR-----FIE-------VLDR--------ESTEKRYVVE---VDIVPS---ISIVKS---KVYA-VRLPNF--KES-----------TNKVEF--E-KEAILRRV--------- : 265

fish_XP_012687177.1 : ACI--RPPK-----FIP-------VQCL-------DGTSSKLWVIE---VDIVPN---YSETQE---KLFY-TSVQKEVEGKK--------------------CKKCLFVRQ--------- : 263

fish_XP_012708385.1 : QCV--RPPR-----FIE-------VMDH--------TSVEKRYVVE---VDIVPS---ISIVRG---KIYK-VRLPNF--KES-----------ANKVEF--E-KETVLRRV--------- : 317

fish_XP_012713253.1 : MCI--KPPR-----FVE-------VLNR-------NETSSEKCVIE---VDIEPQ---SAICEE---NVYHTYNTRKGKKKTK---------------ETESHPSKSFYIRD--------- : 285

fish_XP_012727798.1 : KCI--RNPC-----FIE-------VIDK--------ENAEKTWVVE---YDIVPK---ASIVKG---KLYS-VGVPKF--SEK-----------DSKVKC--E-DKIPYNRV--------- : 312

fish_XP_012990558.1 : SCL--KDSK-----VNT-------------------YKPIAQSHLE------------DHKCRT---DIAD-LMIKHYGEEDA---------------------LNVTLQIL--------- : 296

fish_XP_013126906.1 : TCI--KPPR-----FVG-------VLNK-------NMTSSDKCVIE---VDVVPE---TTICEE---NGYH-TSYTIKKGKKK-----------GKSKETESEPSKCFFIRD--------- : 290

fish_XP_013796981.1 : ACI--REPR-----FVE-------ILLQ-------NGTLSDTFVIE---IDIVPK---HSLCDT---KYFS-TYTYEY--KSK-----------GWNK--------ALFIRD--------- : 345

fish_XP_014005049.1 : ADS--MTKH-----YEE------------------ETLNVTRQILERIGHNELAG------------QLKSDMGLQMMTAQDG----------------------KNYLRRE--------- : 145

fish_XP_014049585.1 : QCI--RPPR-----FIE-------VMDM--------YSTEKRYVVE---VDIVPT---ISIVKN---RVYS-VRLPNF--KES-----------SNKIEH--E-KETIYRRV--------- : 322

fish_XP_014325953.1 : VCF--RKQD-----FLD--------------------------------LNVKSG------------------------------------------------------------------ : 136

fish_XP_015195770.1 : KCI--KPPR-----FVE-------VLNP-------DMTSSDKYVIE---VDIVPA---YLMCQE---NVYH-VYNVE--RKKGKKKI-------KGKDEM----GKPFYVRD--------- : 303

fish_XP_015197335.1 : NCI--RQPR-----FVQ-------VLSL-------DTTMSNRYVIE---VDVVPS---FSLTRG---EVFN-ITMITCDKQGK------------------KHKEDFLFVRD--------- : 361

fish_XP_015232478.1 : KCI--KPPR-----FVE-------VLNK-------NTTLSYEYVIE---VDIEPQ---FSICGE---KTYK-TYT----IKKAKKMTN------ETKSKQ----LKVFYIRD--------- : 216

fish_XP_015252023.1 : MCI--KPPR-----FVE-------VLNR-------NATSADKCVIE---VDIEPQ---FAICEE---NVFH-TYNTK-----------------KGKKKSKETESKSFYIRD--------- : 277

fish_XP_015264177.1 : LCI--HPPV-----FIE-------VINK--------DSQEQHFVVE---VDIEPI---SSTVKD---KIFQ-VRLPKF--NEE-----------SNKVNI--EKQPVIFQRV--------- : 360

fish_XP_015801149.1 : QCV--RPPR-----FIE-------VMDR--------ESTEKRFVVE---VDIVPS---LNIVKN---KVYA-VRLPNF--KKS-----------SNKVEF--E-KETILRRV--------- : 326

fish_XP_015810486.1 : KCI--RPPR-----FVD-------VLNK-------NTTSSDKCVIE---VDIVPE---FDVCKE---MYFH-TYNTK---KGK---------------------EKSFYVRD--------- : 274

fish_XP_016115105.1 : RCI--KPPR-----FVE-------VLNA-------DMTSTGKCVIE---VDIEPS---SIICEDIYFNTYE-VEKNTKKLTDK-------DKMQELKATGKKSDGKSFFIRD--------- : 299

fish_XP_016117315.1 : QCI--RQPR-----FVE-------VLCP-------DSTLSGKYVIE---VDVVPS---YSIVHG---KLFQ-IQTLD---EDS-----------QWKKSK----GKSLFIRD--------- : 396

fish_XP_016395637.1 : FCI--GEPQ-----FVQ-------VVCS--------NSTEELYIVE---VDIKPT---LSIVKN---KVFS-VSLPNF--NEK-----------ANKVQL--E-KNTAFRRV--------- : 313

fish_XP_017207716.1 : ACI--RQPR-----FVE-------VLCP-------DSTLSGKYIIE---VDVVPS---HIIVHG---KQFEIQTLDEEN---------------QWKKSK----GKSLFIRD--------- : 381

fish_XP_017277259.1 : SCI--RNPC-----FIE-------VLDK--------ETTEKTWVVE---YDVIPK---SSIVKD---KLYY-VAVPKF--NEK-----------DGKVEVKRE-EQVSYYRV--------- : 332

fish_XP_017277301.1 : QCV--RPPT-----FIE-------VMDR--------GSTEKRYVVE---VDIVPS---LRIVKN---KVYL-VKLPNF--KES-----------ANKVEF--EKKELILRRV--------- : 327

fish_XP_017325460.1 : KCI--KPPR-----FVE-------VLKA-------DMTFSGKYVIE---VDVEPS---YLVCKD---SFFH-IYNLDSRKAKR-----------SKITTDEKDSGKVFYIRD--------- : 312

fish_XP_017346953.1 : QCI--RPPE-----FIL-------VVDP--------NSTDRHYVVE---VDIVPS---VSIVRN---RVFS-VRLPNF--KEK-----------SNKVEY--E-KGTIYRRV--------- : 316

fish_XP_017550536.1 : ICILKKLPR---------------------------NDLVESLTEN---VDQLQV------------------------------------------------------------------ : 210

fish_XP_017550824.1 : KCI--KPPR-----FVE-------VLEA-------DMTVSRKYVIE---VDIEPT---SLLCEDSFFHVYSVDTRKSKKHSKT-----------RDLKTDEKDSAKLFYIRD--------- : 301

fish_XP_017554653.1 : RCI--REPR-----FVE-------VLNS-------DSTSSSKYVIE---VDVVPS---HSVVYG---KIYYTQTLDEENQWKK-------------------SKGKSLFIRD--------- : 428

fish_XP_017569575.1 : QCI--RPPE-----FIL-------VIDP--------NSTEEHYVVE---VDIVPS---ISIVRN---RVFP-VLLPNF--KEK-----------KNKIEF--E-IETIYRRL--------- : 322

fish_XP_018413083.1 : QCI--RPPR-----FVD-------VLHQ-------ENTQSDLVVIE---VDVVPE---HAYCSS---QIFH-TYQYSC--TDQ-----------KWMKNK----DLCCFVRD--------- : 329

fish_XP_018429299.1 : NCI--RSPK-----FIE-------VVDR--------GSKEQKFVVE---VDIVPA---AQKVKD---KVFE-VTLPKF----------------NDKSNKVIHDKKAIYQRI--------- : 382

fish_XP_018521334.1 : QPI--KEIK-----RKE-------TVVT--------TALLNTSKIS---VEIQKT---LDNLIKGDLKMFH-FYLHEHTKSGH---------------------KPIPRGRL--------- : 283

fish_XP_018526467.1 : KCV--RPPR-----FVE-------VMDR--------DSTEKMYVVE---VDIVPS---ISIVKS---KVFA-VRLPNF--KES-----------SNKIEF--E-KEATLRRV--------- : 329

fish_XP_018541041.1 : MCI--RPPH-----FIQ-------VHSE-------DGTTSNKWVIE---VDVVPT---YSETQE---NVFYTLLSIESAEKKQ------------------QCKTECLFVRD--------- : 457

fish_XP_018558293.1 : TCI--KPPR-----FVG-------VLNK-------NMTSSDKCVIE---VDIVPD---STICEE---NIYHTLNMGTKKAKKK--------VKYKETVENETKQSKQFFVRD--------- : 306

fish_XP_018595329.1 : GCI--RPPQ-----FIR-------VLHR-------NDTTSDKFVIE---IDVVPD---RLFTRG---QHFY-TCNDAVDEKGK------------------PCRQECLFVRE--------- : 373

fish_XP_018611696.1 : WCI--RPPV-----FIEVVDINSTVVDI--------NSTEKRYIVE---VDIVPA---VSIVRN---RVYS-VRLPNF--REK-----------SNKVEF--E-KDTIYRRV--------- : 338

fish_XP_018956725.1 : QCI--RQPR-----FVE-------VLCP-------DGTLSGKYVIE---VDVVPS---YSIVQG---KLFYTQTLDEDS---------------QWKKNK----EKSLFIRE--------- : 401

frog_XP_018091031.1 : DCI--RPPK-----FIE-------VIER--------DCEEQRFVIE---VDIVPD---FNLVKG---KAFQ-VGLPKF--NGK-----------SNKVLL--E-KKAMYRRV--------- : 445

Hemichordata_XP_006825609.1 : KCI--RPPR-----YVP-------VSAP--------GITRSKYVIE---VDIVPH---STICKN---KAFF-VRLPANPEKQR--------------LQNISKTKETLFRRI--------- : 875

lance_EEN48978.1 : -----RPPH-----ISE---------------------------------DICSR-----------------MVVPKSKRSHR---------------------KSLFSSKM--------- : 157

lance_EEN55742.1 : KCFSYKPPF-----RYE-------------------EFKHRDVLLG---IVVIPP-------------CFGSRYTEPCIAEDS--------------LEHGMWHQDELWYRD--------- : 334

mammal_XP_001378512.1 : QCI--RPPR-----FVE-------VLLK-------NNIVSDRFVIE---IDIVPK---HSICEN---KYFQ-VKMQIF--KEN-----------KWGKNA----ELSLYVRD--------- : 339

mammal_XP_003771993.1 : QCI--RPPR-----FVE-------VLLK-------NNIPSDRFVIE---VDIIPK---HSICVK---KYFP-IKMQTF--ENN-----------KWEKNA----EFSLFVRD--------- : 339

mammal_XP_003771994.1 : ECI--REPR-----FVE-------VLLQ-------DNTLSDRFVIE---VDVIPK---YSICKN---TLFY-IKMQVL--KEE-----------KWMEKK----EYSLFVRE--------- : 340

mammal_XP_003782717.1 : KCI--REPR-----FVE-------VLLP-------NSNLSDRFVIE---VDVIPQ---YSECEC---DYFQ-IKMQIY---NK-----------TWEQSP----KFSVFVRD--------- : 340

mammal_XP_003982796.1 : KCI--REPR-----FVE-------VLLQ-------NNTPSDRFVIE---VDVIPK---HSVCEE---KYFL-IRMQNC--KNE-----------TWKPNQ----DLSLFVRD--------- : 344

mammal_XP_004389713.1 : KCI--REPR-----FVE-------VLLQ-------NNTPSDKFVIE---VDIIPK---HSVCKD---KYFY-INMQNC--KDG-----------SWRQDK----DASLFVRE--------- : 343

mammal_XP_004582357.1 : TCI--QGPR-----FVE-------VLLP-------DDTVSDRFVIE---VDVIPH---YSVCGQ---DYFR-IKKQIC--NNK-----------TWKQSS----EFSVFVRD--------- : 331

mammal_XP_004582358.1 : KCV--REPR-----FVE-------VLLQ-------NNMPSDRVVIE---VDIIPK---HSICKK---RYFY-VKMQSC--KDK-----------VWKQNQ----ECSLFVRE--------- : 345

mammal_XP_004622892.1 : KCI--REPR-----FVE-------VLLQ-------NNTSSDRFVIE---VDVIPK---HSICQE---KYFY-TKMQEL--KDG-----------IWKQTE----NTSLFVRE--------- : 342

mammal_XP_004646313.1 : KCI--RQPR-----FVE-------VLLQ-------NNTLSDRFVIE---VDIIPK---HSVCKQ---KYFY-IKMQIQ--TDN-----------IWKQSQ----DHSLFVRD--------- : 348

mammal_XP_004702866.1 : KCI--REPR-----FVE-------VLQQ-------NNTLSDKFVIE---VDVIPR---HSVCKE---KYFY-ITMQNC--KDG-----------PWRPNK----EHSLFVRE--------- : 343

mammal_XP_005340592.1 : RCI--REPR-----FVE-------VLLQ-------NNTLSDRFVIE---VDIIPK---YSICKE---KYFY-IKMQCC--TDK-----------MWKQHQ----DHSLFIRE--------- : 344

mammal_XP_005388084.1 : QCI--REPR-----FVE-------VLLQ-------NNTLSDRFVIE---VDVIPK---HSVCKE---KYFY-VKLQSE--TDK-----------IWKQNE----NCSLFVRD--------- : 345

mammal_XP_006163552.1 : KCI--RQPR-----FVE-------VLLQ-------NNMPSDRCVIE---VDIIPK---HSVCKR---KYFY-IKMQSC--QNE-----------TWKQNP----NHSLFIRE--------- : 345

mammal_XP_006779273.1 : KCI--REPR-----FVE-------VLLQ-------NNTPSDRFVIE---VDVIPK---HSVCKE---KYFY-IKMQNC--KAE-----------IWKQNQ----DASLFVRD--------- : 345

mammal_XP_006834357.1 : KCI--REPR-----FVE-------VLQQ-------NNTLSDKFVIE---VDVIPS---HYVCKE---KHFC-VTMQNC--KDG-----------SWRPNK----EHSLFVRD--------- : 339

mammal_XP_006891533.1 : QCI--REPR-----FVE-------VLQQ-------NNTLSDKFVIE---VDVIPK---SSVCKM---KSFY-ISMQND--KDG-----------TWRQNK----DHSLFVRD--------- : 343

mammal_XP_007522555.1 : KCI--REPR-----FVE-------VLLQ-------NYTPSDRFVIE---VDVIPK---HSICKE---KYFY-TNMQSY--KND-----------TWKQNQ----ESSLFVRD--------- : 339

mammal_XP_007938002.1 : KCI--REPR-----FVQ-------VLQQ-------NSTLSDRFVIE---VDVIPR---HSVCKK---KYFY-IKMQIC--KDS-----------VWRQDK----EYSLFVRK--------- : 343

mammal_XP_008062458.1 : KCI--RAPR-----FVK-------VLLP-------DSTLSDRFVIE---VDVIPQ---YAECED---HYFQ-IKMQIY--KNR-----------IWKQSP----EFSVFVRD--------- : 346

mammal_XP_008259950.1 : KCI--REPR-----FVE-------VLLQ-------NSMPSDRFVIE---VDVIPK---HSVCKE---KYFY-IKMQSC--KDN-----------VWKQNQ----EYSLFIRE--------- : 344

mammal_XP_008524371.1 : KCI--REPR-----FVE-------VLLQ-------NNTPSDRFVIE---VDVIPK---HSICKE---KYFY-IKMQNC--KNE-----------KWKQNE----DHSLFVRD--------- : 344

mammal_XP_008820771.1 : ECI--REPR-----FVE-------VLLQ-------NNTLSNRFVIE---VDVIPK---HSICKE---KYFQ-VNMQGY--TAK-----------TQKQDQ----NPSLFVRE--------- : 344

mammal_XP_010601767.1 : KCI--REPR-----FVE-------VLLQ-------NSTPSDRFVIE---VDIIPK---YSVCKE---TYFY-IKMYSL--IDK-----------TWKQNQ----GHSLFVRD--------- : 345

mammal_XP_011853657.1 : KCI--REPR-----FVE-------VLLQ-------NNMPSDRFVIE---VDIIPK---HSICKD---KYFY-IQMQIC--KDK-----------IWKQNQ----NTSLFVRE--------- : 346

mammal_XP_012791283.1 : NCI--REPR-----FVE-------VLLT-------NGIPSDRYVIE---VDIIPK---YSECEH---DYFQ-IKMQNF--NNK-----------TWNKSS----RFSVFVRD--------- : 342

mammal_XP_012881542.1 : KCI--REPR-----FVE-------VLLQ-------NNTPSDRFVIE---VDIIPK---HSVCKE---KYFY-IKMQNN--ADK-----------TWKQDQ----DHSLYVRE--------- : 335

mammal_XP_012934202.1 : KCI--REPR-----FVE-------VLLP-------NSTVSDRFVVE---VDVVPQ---FSECKH---DYFQ-IKMQNC--NNK-----------AWQQSS----KYSVFVRD--------- : 345

mammal_XP_013013639.1 : KCI--RQPR-----FID-------VLLQ-------NNTLSDRFVIE---VDIIPK---YSVCKE---KYFY-IKLQSL--TDK-----------TWKQNQ----NLSLFVRD--------- : 341

mammal_XP_016016798.1 : KCI--REPR-----FVE-------VLLQ-------DNTSSGRFVIE---VDVIPK---QSVCKE---NYFY-IKMQNC--KNE-----------TWKQNQ----DHSLFVRE--------- : 345

mammal_XP_016281899.1 : KCI--REPR-----FVE-------VLLQ-------DNTSSDRCVIE---VDVIPL---YSICKE---FYFS-IKQQVL--KDG-----------KWKLND----EYGLFVRE--------- : 331

mammal_XP_017199853.1 : SCI--RVPR-----FVE-------VLLP-------DSTVSDRFVIE---VDVIPH---YSVCGH---DYFQ-IKKQIYDNNNK-----------KWEQSS----KFSVLVRD--------- : 309

mammal_XP_017508280.1 : KCV--REPR-----FVD-------VLLQ-------NNTPSDRFVIE---VDVIPK---YSVCKE---KYFY-IKMQNC--KNE-----------TWKPNQ----DHSLFVRD--------- : 354

mollusk_EKC42356.1 : YES--RNAE-----FRT---------------------LPPKITLS---LPMFTP---FGIDKEQLRLQFGSLSASSIKTEED-----------EYTMDSPVAEXXXXXXXX--------- : 286

mollusk_XP_011436570.1 : QCL--RPPQ-----FVR-------VNSR----GSTDQEETQLFVVE---VDVVPS---YSLTGH---DTFY-LREKNEKHENV-----------PRIYKFDKNIPKVLFDDD--------- : 538

mollusk_XP_011440496.1 : GCI--KKSSDFALDMVS-------VWNT-------PREESGFIIIQDDHLESEIDDDDISKLKNALEKQSS--SFSYFPCCEYQVVTFQKRMFISIEIAYLHDNRMPILKSD--------- : 390

mollusk_XP_013061423.1 : STV--RDVR-----FVP-------VIDT----------EDHRYVIE---LDIVPA---TVALDKDIVRTKE-KFLPDHFRRNH-----------KGK-------DVVVFKFS--------- : 505

mollusk_XP_013061425.1 : NTV--RDAV-----FVP-------VI----------GAEKNRFVVE---VDVVPS---TVVLENDIIRTKE-HSLPERFLRNH-----------KGKDSVVFKFSEDGFPKI--------- : 288

Musmu_NP_034286.2 : ACI--REPR-----FVE-------VLLQ-------NNTQSNRFVIE---VDVIPR---HSICQE---KYFY-IMMQSS--TGK-----------TWKQSK----DTSLFVRE--------- : 341

rept_XP_008110733.2 : DCI--RKPR-----FVE-------VLHQ-------NGVASETFVIE---VDVIPK---SSSCQS---KSFP-TNTYNF--KSK-----------KWEP--------GLFIRD--------- : 349

rept_XP_008112638.1 : QCI--HPPV-----FIE-------VIST--------DSHEQFFVVE---VDIEPL---FDIVKG---KLFQ-VRIPDF--IEK-----------SNKINF--ERDKIAFQRV--------- : 359

rept_XP_015264172.1 : DCI--REPR-----FVE-------VLQQ-------NNTASETFVIE---VDVVPK---HSCCQS---NYFP-SKIYNY--VER-----------KWES--------CLFIRD--------- : 370

shark_XP_007886940.1 : RSI--RPPR-----FVE-------VTSG-------DPGEQQCFVIE---VDIVPS---LAVVQG---IFYS-VRLPVL----------------KDIKEKNTRSPWVIYKRE--------- : 205

Strpu_XP_003727489.1 : IYA--RDDR-----LKT-------RVNTHQHQPLASGKKSDNLNVDALTLDVLPS------------DSTV-VSQENK--------------------------REEQLTEV--------- : 256

Strpu_XP_011667260.1 : --E--GVNG-----YTL-------KWCS-------KAELIEDFELT---AVHCKS---LMIRRERYLEQER-MGLTTIHVDSR-----------GVDAPGKTQTITAPSLKD--------- : 503

turtle_XP_006139744.1 : ACI--REPR-----FVE-------VLLQ-------NNTPSNTFVIE---VDVVPK---HSICEA---TFFY-TNNYNF--KSK-----------TCER--------SLFVRD--------- : 363

turtle_XP_007060369.1 : ACI--REPR-----FVE-------VLLQ-------NNTPSNTFVIE---VDVVPN---HTICET---KFFH-INNYNF--KTK-----------TWEP--------SLFIRD--------- : 363

turtle_XP_014430084.1 : NCI--RPPV-----FTE-------VIEK--------DTEDQSFVVE---VDIEPS---YSTVKG---KIFE-VCLPRF--NEE-----------SNKTIY--EKKPVLYQRL--------- : 390

Xenla_OCT63184.1 : QSQ--------------------------------GDTEVKEVCIE------------NKVTKD--------------------------------------------------------- : 162

Xenla_XP_018122800.1 : QCI--RPPR-----FVD-------VLYQ-------EKTQSDLVIIE---VDVVPE---HAYCNT---EVFS-TYQQVL--IDN-----------TWKKSK----EKSCFIRD--------- : 324

Xentr_XP_002943568.3 : DCI--RPPK-----FIE-------VIER--------DGEEQRFVVE---VDIVPD---FHLVKG---KAFQ-VGLPKF----------------NEKSNKVSHEKKAMYRRV--------- : 534

Xentr_XP_012813408.1 : LCI--RPPI-----FIE-------VYDK--------DSDTKGFVVE---VDIEAS---LVNVKD---KVFQ-VCLPNI--TD------------NNKIRY--K-EPATYERN--------- : 343

1460 * 1480 * 1500 * 1520 * 1540 * 1560 *

Homsa_NP_060124.2 : -------GTSSK---------DITKN------K-VD---FRA---FK--A-DFK-TL-AESRK---------AAEEKF--RAKTNK--K---EREGPK---LVKLLTGNQDL--LDN---- : 407

Homsa_NP_689916.2 : -------GASSR---------DILANSKQ-R-D-VD---FKA---FL--Q-NLK-SL-VASRK---------EAEEEY--GMKAMK--K---ESEGLK---LVKLLIGNRDS--LDN---- : 411

fish_XP_015241820.1 : ------------------------------------------------------------------------------------------------------------------------- : -

fish_XP_018543893.1 : ------------------------------------------------------------------------------------------------------------------------- : -

fish_CDQ83481.1 : ------------------------------------------------------------------------------------------------------------------------- : -

fish_CDQ89582.1 : ------------------------------------------------------------------------------------------------------------------------- : -

fish_XP_005809248.1 : ------------------------------------------------------------------------------------------------------------------------- : -

fish_XP_007231320.1 : ------------------------------------------------------------------------------------------------------------------------- : -

fish_XP_007254667.1 : ------------------------------------------------------------------------------------------------------------------------- : -

fish_XP_008277954.1 : ------------------------------------------------------------------------------------------------------------------------- : -

fish_XP_010746900.1 : ------------------------------------------------------------------------------------------------------------------------- : -

fish_XP_012685053.1 : ------------------------------------------------------------------------------------------------------------------------- : -

fish_XP_013123332.1 : ------------------------------------------------------------------------------------------------------------------------- : -

fish_XP_014011897.1 : ------------------------------------------------------------------------------------------------------------------------- : -

fish_XP_014011904.1 : ------------------------------------------------------------------------------------------------------------------------- : -

fish_XP_014264868.1 : ------------------------------------------------------------------------------------------------------------------------- : -

fish_XP_014264875.1 : ------------------------------------------------------------------------------------------------------------------------- : -

fish_XP_014854842.1 : ------------------------------------------------------------------------------------------------------------------------- : -

fish_XP_017578465.1 : ------------------------------------------------------------------------------------------------------------------MED---- : 3

fish_XP_012713132.1 : ------------------------------------------------------------------------------------------------------------------------- : -

fish_XP_010787190.1 : -------GANTK---------EIP--------E-DD---LFL---FI--Q-RLR-EK-DQQRE---------DAESSS-NQTTVEC--E---EDQERK---LSILLTCGKTH--MDD---- : 228

fish_XP_017319342.1 : -------GANTK---------PVD--------D-IN-----R---FY--Q-DVR-SR-DIQRE---------TAEKSH-GITAPKP--C---KDLEKK---LIMFITNGKKQ--IEK---- : 353

Alligator_KYO30992.1 : -------GAKSE---------PVKTEELVPFIKGLQ-------------------ER-DARRE---------KAESSSREDHDGIS------QDLGRK---LSILLTDGKNY--IND---- : 394

Alligator_XP_006025954.1 : -------GASSK---------NIIKN-------------LAG---FK--T-KLQ-SL-ADARQ---------AAEKKH--AEKTKK--K---QTEGLK---LRCLLTGNQDL--LDN---- : 421

amphibia_XP_018413082.1 : -------GASSK---------NILANVQH-N-T-AE---WKS---FH--T-EIK-DR-DDARK---------TAEDNQ--KRKLKR--S---QEHGSK---LVRLLTGNRDT--LDN---- : 399

bact_AGA70736.1 : -------LPEVK---------------------------------------RFILAYVDPDAQL--------ELDSSEGSAFSMEV------NNLFEE----TDFFQGNGRW--------- : 188

bact_CUJ86040.1 : -------CSEAD---------------------------------VKLPGEGAVIPNIMAERS-----------------------------NEISDD---AAIFVDGLVKN--------- : 337

bact_ESW58583.1 : -------GSKGS--------------------------------------------------------------------------------IAYGEDTPSWQTLINAADIS--ISG---- : 151

bact_GAC67715.1 : -------AAALN---------WASKAAVDPERARAA---------YLVAAWNLR----SWLRD---------YDSDEWQALTSPAPRTAALRGDIIPEAEILRLWSTFT-----LRN---- : 408

bact_KMO66808.1 : -------AAGLN---------WASKPMDANQRSVGF---LYACWALRDWLTRYLPDYWQSAAT---------IADKGSTAVRVKEADVSVEDGEAKLRHY-WELLQSIPTDT--------- : 431

bact_KUO79116.1 : ---------------------AVGINYLCKKKIEEDTLLKKA---LFYAAIHLN-TLFNLTKY---------DWNKKDGGPLKDEV------GDEGDI---WDACDELRTQY--------- : 415

bact_KYG06363.1 : -------NKVFR---------EGSDGPGLSQAQ------YQQALVWAAFYLRRLLELWPAAVP---------EGVRSVPILSARVPAPSRITEEQWRS---VSAFDGSGLNVLVLSP---- : 426

bact_ODA70488.1 : -------AAGLN---------WAAKPLDDMSLRHAA---------LASAGWAARLLLRDRHAKVWFE-----MAKEDPIGTVGLATSSSATPVTAEKALRLWKPFRQPHSGM--------- : 435

bact_OGP49286.1 : -------GPAYD-----GSHWPNRIQFTRYTKSESKKEVEIRSVVFDPRNIKWVEDTSTFTSN---------GSKEGYGYFSLWERPVPQIPEIHISH---WNEFYAACNKF--------- : 383

bact_SCF05533.1 : -------FPGTD--------------------EARV---CLAYAGWY----TLRYRRMFTVKE---------DQTPTP-PTA----------EQNDAL---WEQGWQAVSRF----S---- : 403

bact_SCX17622.1 : -------DSNAQ----------------------LD------------------------------------LIEDSI--------------YGDGNG---WYQILDDTCDF--------- : 200

bact_SFJ80976.1 : -------AAALL---------WARRFPAGDERA-RT---CLAYAGWY--AMRYDRDFPAGAPE---------FGGASS-AVPATREGARETHESARARDELWETFWDAASGF----S---- : 416

bact_WP_006128369.1 : -------GERTA---------------------------------------DTRAGYRTGLAD---------SADSGYTDDRPRAE------AEEAL----WRSLWDATSGFA-------- : 462

bact_WP_006365791.1 : -------GTQND------------------------------------------------------------RAVGSELRRIWTWFQNGDVGIPEGQSEDTWLRFFDAVHHF--------- : 181

bact_WP_006849212.1 : -----------------------------------------------------------------------------------------------------DFSILDNITHN--LEQ---- : 219

bact_WP_007897046.1 : ------------------------------------------------------------------------------------------------------------------------- : -

bact_WP_030194308.1 : -------AAGLN---------WAAKPLDDMALRRAA---------LASAGWAARLLLREHHQE---------LWVDIAAEIGTSTTGAA---TATGAKPVTAQEALRRWKPF--RQT---- : 431

bact_WP_030198971.1 : -------GAALN---------YAAKRRSPVQQRVTA---------YRMAGWATHLLLAEDHPTLLAQV----LADAQQAHMTAPDGVVATADAKPAVDEAAAAELMTEQAKPFIADT---- : 436

bact_WP_030898614.1 : -------GLHAP---------------------------------------TATAPRCTLTRA---------VEATPQVSAAVDRL------AENCDR---WTALRTTIAV---IDS---- : 447

bact_WP_041219555.1 : -------LPEVK---------------------------------------RFILAYVDPDAQL--------ELDSSEGSAFSMEV------NNLFEE----TDFFQGNGRW--------- : 193

bact_WP_050505446.1 : ---------------------------------------------WYAMRYRRSFPASTPQRQ---------EALSSPTTPDEAG-------RVEQVREDLWSSFWSTVSGFA-------- : 368

bact_WP_055538636.1 : -------GAALN---------YAAKRKSPDQQRVTA---------YRLAGWATHLLLTEYHPT---------LLERVLADAHQPQTLVPRREVSAAGAKAVAEEMVEQVKPFVADTH---- : 431

bact_WP_062134708.1 : -----------------------------------------------------------------------------------------------------RFKLFTAGTS---------- : 25

bact_WP_062799339.1 : -------GGQER---------------------------------------AIEHARETTVRL---------IEAWNAVAQAGVPV------EDL------TSDLLAAGLPLPSPTE---- : 269

bact_WP_066463897.1 : -------NAFLR------------------------------------------------------------PIKELNEFNSYTEL---------------LELFVLENENN--------- : 204

bird_XP_002194798.1 : -------GAKSE---------SVQ--------R-NK---LTA---FI--Q-ATP-DR-DAQRE---------RAELSS-TQESTEI--A---QDLGRK---LSILLNDGKTY--MDD---- : 402

bird_XP_005010984.1 : -------GASSK---------NI-IN------T-DE---FEK---FK--C-NLT-LL-ADSRK---------RAEEEY--NLKQKK--T---MNEGLK---LISLLTGNRDM--LDD---- : 405

bird_XP_005505023.1 : -------GASSR---------NI-LN------T-KD---FET---FK--G-NLT-SL-ADSRK---------RAEEEY--KLKQKR--S---MNEGLK---LVSLLTGNKDT--LDS---- : 406

bird_XP_008492486.1 : -------GASSR---------NI-YG------Q-KE---FET---FK--S-SLS-SL-ATSRK---------KAEEEY--NLKQHR--P---VLEGFK---LSRLLTGNKDS--LDD---- : 406

bird_XP_008628214.1 : -------GASSA---------NI-YN------T-KE---CEK---FK--G-SLT-TL-AERRR---------RAEEEH--NFKPKK--S---PKEGLK---LVSLLTGNRDS--LEN---- : 405

bird_XP_009068034.1 : -------GAKSE---------PVQ--------R-SK---LTA---FI--Q-AMQ-DR-DAQRE---------TAELSS-TKVQTEV--S---QDLGRK---LSILLNDGKSY--MDD---- : 399

bird_XP_009080343.1 : -------GASSR---------NI-YN------M-EE---FAQ---FK--D-KLS-SL-ADRRK---------SAEERH--NLKQNK--P---MNEGLK---LVSLLTGNKDS--LDS---- : 406

bird_XP_009486154.1 : -------GASSK---------NIQ--------NTKE---FKT---FK----GTLTSL-AESRK---------RAEEDY-NLKQKKP------MNEGLK---LVSLLTGNRDS--LDS---- : 406

bird_XP_009640122.1 : -------GASSR---------NIY--------NTEE---FET---FK----GTLTSL-ADSRK---------TAEEEY-NLSQKKP------MDEGLK---LVSLLTGNRGS--LDS---- : 404

bird_XP_009672623.1 : -------GASSR---------NLSQN------S-KE---LES---FK--S-TLS-SL-ADHRK---------NAEEEY--TLKKKK--I---GNEGPK---LVSLLTGNKDL--LDD---- : 395

bird_XP_009695665.1 : -------GAKSE---------TVK--------H-DD---LTA---FI--Q-GLQ-DR-DAQRE---------KAELSS-AEVHTEI--S---QNLGRK---LQILLNDGKSY--MDD---- : 547

bird_XP_009886214.1 : -------RASSK---------NIQ--------NTKE---FDT---FK----GTLTSL-AVSRK---------RAEEEYINLKQRKS------KNEGLK---LVSLLTGNRDS--LDS---- : 407

bird_XP_009934438.1 : -------GASSK---------NIH--------NTKD---FET---FK----GNMTSL-ATFRK---------RQEEDY-NLKPKKS------MNEGLK---LVSLLTGNRDS--LDS---- : 400

bird_XP_009999877.1 : -------GASSK---------NI-HN------T-KE---FET---FK--S-NLT-SL-ATSRK---------SAEEEH--NLKQKK--P---MDEGPK---LASLLTGNKDS--LDS---- : 405

bird_XP_010119033.1 : -------GASSK---------NI-KN------T-KE---FET---FK--S-TLT-LL-ADSRK---------KAEEEY--NLKQKR--S---MNEGLK---LISLLTGNQDS--LDS---- : 404

bird_XP_010144931.1 : -------RASSK---------NI-QN------T-KE---LET---FK--C-TLT-SL-ANSRK---------RAEEDY--NLQQKK--S---MN-GLK---LISLLTGNRDS--LDS---- : 393

bird_XP_010222910.1 : -------GASSK---------NIIKN------S-KE---SEF---FK--S-NLT-SL-ANSRK---------SQEEAF--ILKQKK--S---GNEGRK---LASLLTGNKDL--LDD---- : 395

bird_XP_013160560.1 : -------GASSR---------NI-QN------T-KE---FET---FK--G-TLT-TL-ADSRK---------KAEEDY--NLKLTK--P---MNEGLK---LVSLLTGNWDS--LDS---- : 409

bird_XP_014795808.1 : -------GASSK---------NIC--------NSKE---FES---FK----GTLTSL-ANSRK---------RAEEEY-NLKQKKS------MKEGLK---LISLLTGNRDS--LDS---- : 406

bird_XP_015156587.1 : ----------------------------------------------------------KCTKE--------------------------------------LKTL---------------- : 323

bird_XP_015709021.1 : -------GASSK---------NI-QG------T-KE---FKT---FT--C-NLP-LL-ADSRK---------RAEEEY--NLKQKK--T---TLEGRK---LVSLLTGNRDV--LDD---- : 410

brachiop_XP_013415988.1 : -------ISPSQ---------GMEKPCVAEQWHREASWNIGE---IWVRRGTSNQILPVKHHD---------SVYKWFQKPTPYSEVVKGRGKPHSLRNNALQKFLDKVDMF--------- : 633

Cioin_XP_018668123.1 : -------RERTS----------------------------------------------PDLRN-----------------------------DD-------SMNILSTLDHF--------- : 186

Cnidaria_EDO35430.1 : -------GTEDQ---------EKAEDADSWARAVSDLFEDREHAFLFASDFKSQWEKVFSWKS---------TMEVFMAKLSKSVERRPVSPTSFNKTA--LPRISEEDKQL--VEEMCLF : 552

Cnidaria_KXJ09690.1 : -------QAKMD-------------------------------------MMGEEVAEKVAHRL---------VTKNGSKSEKQPRFQ-----DLSGPRWDDWLRFCDNVQDF----N---- : 498

Cnidaria_KXJ11435.1 : -------QELRT---------EMQDIPAKVAEEVQK---------------GLLYSQSLANSN---------PSHRLQ--------------DASDPKWDEWIRFRDFVRDF----D---- : 491

Cnidaria_KXJ17955.1 : VRALEAVAAGVPMLINTSFGFADVLFKHFHKKFWEFLCFSDDEDAYETWSERIDALFDDRDKYFALAAELKNEWQKMFSWEALVNKLFYKFSMSGYFT---SPQFAPVDXXSSRPSK---- : 667

Cnidaria_KXJ23466.1 : VIVSNSYGITDVLLDHFDHKFWIFLSSAKEEDDYES---WSDRIDVFLDNRDEKFALASELKH---------EWEKKFTWDSATTKIL----HHLSRSGEFPKAAKACNENYPSLPS---- : 645

Cnidaria_XP_015751626.1 : -------DGVLD---------DREDAFGMAESLWDK---WSS---------AFTWDSAADNVL---------KRMSSFGVFSAPSPSADLQITLPSQCSDSARNFMQHEVDL--LNR---- : 957

Cnidaria_XP_015751629.1 : -------DGVLD---------DREDAFGMAESLWDK---WSSAFTWD----SAADNVLKRMSS---------FGVFSA-PSPSADLQIT-LPSQCSDS---ARNFMQHEVDL--LNR---- : 831

Cnidaria_XP_015758975.1 : -------DGVLN---------DREGAFDMAESLWNK---WSSVFTWDSAAYNLL------------------KRMSSSGVFSVPSPSADMQITLPSQCSDSALNFMQHEVDL----P---- : 802

Cnidaria_XP_015766106.1 : -----------------------------------------------------------------------------------------------------VKSFLSGATPP--------- : 178

Cnidaria_XP_015766118.1 : -------GADRA---------KIVEIQSQSDEQLQAGSYYKGLFLAENKRLRVVENKMDKLESKMDT-----VIEIVSQLRDGASKEPLSPKDSSGQKWDDWLSLCDAVGDF----D---- : 509

Cnidaria_XP_015766124.1 : ---------KLE---------------------------------------SIESKLDTVIAN---------QCSEASSNAPSSPK------ILGGPDWDEWIRFRDAVGEFETQQN---- : 392

coelacanth_XP_006000513.2 : -------GASSR---------NIFPQPDDTK-K-KE---LNK---FL--T-GLP-QL-SEARK---------AAEENY---SRGSK--V---MNQGLK---LMDMITGGKNM--LDD---- : 334

fish_CDQ87765.1 : -------GPKSI---------NILADNNPRSLQ-EKMRGLTE---------EVK-CW-SSARK---------SKEESN--CQQPNQ------NHQGQR---LKQLITHGRDT--LEN---- : 652

fish_CDQ92295.1 : -------GANTP---------RIS--------E-DG---LIH---FI--Q-DLR-DK-DQQRE---------EAELSD-NQTASDC--R---EDQKRK---LSILLTCGKKY--MDD---- : 392

fish_KKF13953.1 : -------GSKTE---------PVT--------D-NA---LSD---FY--Q-RVR-DR-DAQRE---------EADKSQ-LFSGQDS--G---QDLGRK---LKVLMTSGKKF--IEK---- : 385

fish_XP_004085798.2 : -------GPQST---------NILEDPNPRVTQ------------NKLKGLTDKVKSWATARK---------SADERCENRIFQ--------SNQGQR---LKQLITRGKDA--FDS---- : 451

fish_XP_004552746.3 : -----HYGSDEA---------PQVAIQTLKEIN----------------QLKLARQLEERISR---------LMQMTLSKESVRKE------ANQGEK---LKTLLSCGGNS--LDN---- : 370

fish_XP_004558390.1 : -------GSKTE---------PVS--------D-KD---LSD---FY--Q-RVK-DR-DAQRE---------EAEKNH-FLIAPEM--C---QDLGRK---LTMLMTSGKKF--IEK---- : 399

fish_XP_004576250.1 : -------GSRSI---------NILADTNPRVVQ-EDMKSLTE-----------KVKHWASARK---------SAEEKDEKLQPQ--------GHQGQR---LKQLITRGRDS--FDN---- : 466

fish_XP_005161888.1 : -------GSKTE---------PVA--------D-LM-----E---FY--Q-HVS-LR-DSQRE---------EAEKMQ-KFTSPEP--S---QNLGKK---LTMLITGGKKV--MDK---- : 371

fish_XP_005478813.2 : -------GGPPE----------------------GQ---LMQ----------------MTLSK---------ESVRKE--------------ANQGEK---LKTLLSCGGNS--LDT---- : 410

fish_XP_005806405.2 : -------GAKTP---------RIS--------D-DE---LIR---FI--Q-DLR-EK-DQQRA---------EGESSA-NQTPLEF--R---EDQKRK---LSILLTGGKTH--MDN---- : 390

fish_XP_006625674.1 : -------GASSK---------DLMGNRNQKEMK------------LELKKFSDSIKGLDSLRK---------SMESKTSQMKRP--------SNQGEK---LKHLITCGKGT--LDN---- : 628

fish_XP_006635547.1 : -------GAKTE---------PVS--------D-LN-----D---FY--S-RVK-DR-DARRE---------EAEASY-FQNWPDL--C---EDLGRK---LTVLITSGKKY--IEK---- : 476

fish_XP_006635548.1 : -------GANTV---------PIS--------D-DD---QVE---FT--L-GMP-ER-DSRRE---------EAELSG-SQSPSDI--R---EDLGRK---LTLLLTGGKKK--MDD---- : 385

fish_XP_006791552.1 : -------GSKTE---------PVS--------D-KD---LSD---FY--Q-RVK-DR-DAQRE---------EAEKNH-FLIAPEM--C---QDLGRK---LTMLMTSGKKF--IEK---- : 388

fish_XP_006791631.1 : -------GANTP---------RVP--------E-DD---LVL---FI--Q-GLR-EK-DQQRE---------EAESTS-NETTLDF--G---EDQKRK---LSILLTCGKKH--MDN---- : 391

fish_XP_007241837.1 : -------GSRTQ---------PTD--------A-NT---------FH--R-KLQ-SI-DSQRE---------AKEQEP-ELANPEV--P---QDLGRK---LIMLVTSGKKI--MEK---- : 377

fish_XP_007242299.1 : -------GAATR---------DMQNIPPKELAKVIA-------------DLNIL----DTKRK---------EAETKPNRKRK---------LNQGEK---LKSLLTDSD----------- : 502

fish_XP_007557414.1 : -------GSKTE---------PVS--------D-KD---LGD---FY--Q-RVK-DR-DTQRD---------EAEKKQ-FLIAPEI--C---QDLGRK---LTMLLTSGKKF--ITK---- : 381

fish_XP_008284284.1 : -------GGSSS---------DLLKPTTSAKPK-TE---YNQ---FV----DSVAQR-SQLRK---------KAEEKHLSVIKS--------STQGSR---LSQMITGGSSS--LDK---- : 358

fish_XP_008284762.1 : -------GANTK---------RVT--------D-DD---LVE---FI--Q-GLR-EK-DQQRE---------EAEASS-HETPLDF--R---EDQKRK---LRILLTCGKTH--MDN---- : 360

fish_XP_008285761.1 : -------GSKTE---------PVS--------D-KD---LSD---FY--Q-RVK-GR-DAQRD---------DAEKNQ-FLFAPEI--C---QDLGRK---LTMLMTSGKKF--IET---- : 382

fish_XP_008336142.1 : -------NDKNR---------IGVAELMTDHYGSKEALCIFK-------DVLEEIDQLDLASR---------FGKTLAESQLNLERSSKEVLQKEGDQGYKLKNLLTCGGNS--LDC---- : 369

fish_XP_008417602.1 : -------GPQSV---------NILADTNPRVGQ------------KKLKDLTEKVKNLVSARK---------AAEENKERCFPQ--------SHQGQR---LKQLMTRGRHV--LES---- : 496

fish_XP_009510531.1 : -------GASSK---------NIK--------KTKE---FET---FK----GTLTSL-ADSRK---------RAEEEH-NLKQKKS------MNEGLK---LVSLLTGNRDS--LDS---- : 405

fish_XP_010752845.1 : -------EGKDT---------MDTAKLMTDHYGSRDALRVTK-------DILKEINQRDLVRQ---------LEKSMGQLELRLPKEVLKKEANQGDK---LKNLLTCGGNS--LDN---- : 365

fish_XP_010755274.1 : -------GGSTR---------DLLKLTTSAKPM-EE---YNQ---FV----ECMAKR-SQLRK---------QAEEKHLSVIKS--------STQGSR---LSEMITGGSLS--LDK---- : 356

fish_XP_010767524.1 : -------TKDTA---------KIMTDHYGTYEAVQVTRDILL-------NINQRPLACDLEKK---------TGQLKQQSLLSKSL--LKKEANQGEK---LKNLLTCGGNS--LDQ---- : 371

fish_XP_010781233.1 : -------GGSSR---------NLLAPTTSTKLM------------EEYNQFVDGMAQRSQLRK---------QAEEKHLNVIKS--------STQGSR---LSQMITGGTHS--LDK---- : 381

fish_XP_010791966.1 : -------GSKTE---------PVN--------D-KD---QSD---FY--Q-RVQ-DR-DAQRE---------EAEQNL-YLSAPDF--C---QDLGRK---LKVLMTSGKKL--IEK---- : 326

fish_XP_012687177.1 : -------GASST---------DVLSEQNPRILQEKI---MAV---------SADVQDWASTRK---------SVEQRRFQKPCP--------SHQGRR---LQQLITLGRDT--LEH---- : 330

fish_XP_012708385.1 : -------GSKTE---------PVG--------D-TD---QLD---FF--Q-RIQ-DR-DAQRE---------EAEK---YPCDPEI--Y---QNLGRK---LTMLMTRGKKF--IEK---- : 376

fish_XP_012713253.1 : -------GGSSR---------DLLASNASSSVE------------FS--RFVDHMTQLSQLRK---------NAEEKHVKTIKG--------TTQGSR---LTRMITGGTLS--LDK---- : 350

fish_XP_012727798.1 : -------GANTP---------RIS--------E-DE---LIS---FI--Q-NLK-EK-DQQRE---------EAESSS-SETALEF--K---EDQKRK---LSILLTGGKAH--MDN---- : 373

fish_XP_012990558.1 : -------GRLQE---------NALAS-------------------------RLRSDMGLQTMT---------LKDAKKDLWIE---------TNQGDK---LKNLLTFGGNM--LEH---- : 349

fish_XP_013126906.1 : -------GGSSR---------DLLAQPNKREYE----------------QFLESMAQRLELRK---------KAEEKHLSVIKN--------STQGSR---LSHMITGGSLS--LDK---- : 353

fish_XP_013796981.1 : -------GASSK---------NIIQN------S-KE---LEL---FK--S-TLT-SL-AKSRK---------KAEEEY--ILRQKK--T---GNEGLK---LVSLLTGNRDL--LDD---- : 407

fish_XP_014005049.1 : --------------------------------------------------------------------------------------------TNQGNK---LKNLLTYGGNM--M------ : 163

fish_XP_014049585.1 : -------GSKTE---------PVS--------D-QN-----N---FY--Q-RVR-DR-DAQRE---------EAEHCR-YVNTPDI--C---QDLGRK---LTMLITSGKKF--IEK---- : 381

fish_XP_014325953.1 : ---------------------P-------------------------------------------------------------------------------AVKILAELKKL--------- : 148

fish_XP_015195770.1 : -------GASSR---------DLLAETSKSKPL-EE---YNR---FI--E-STVKEL-ALMRK---------EAEDKR---LAAVK--S---SVQGSK---LREMITGHTWS--LDK---- : 371

fish_XP_015197335.1 : -------GASSK---------NILANKNIREVL------------SQLSEIKENMKFLVSRRE---------ASELEGPKEPGQ--------SNQGQR---LKRLIGFGKDT--LEN---- : 428

fish_XP_015232478.1 : -------GGSSR---------DLFAPTTSSKPM-VE---YNK---FV--D-GIP-QL-SKLRK---------DAEEKH---SEAIK--S---STQGSK---LMHMITGGTLS--LDK---- : 283

fish_XP_015252023.1 : -------GGSSK---------DLLASDTSSKEY-ER---FVH---------HMANEL-SKLRK---------NAEEKH-FKRIKN-------TTQGSR---LAHMITGGTLS--LDK---- : 342

fish_XP_015264177.1 : -------GANSV---------PVKNDGLVAFIQ------------------NLQ-DR-DARRE---------KAELPNNEHLIDDP------QNLGRI---LSVLLTNGKSY--MND---- : 421

fish_XP_015801149.1 : -------GSKTE---------PVS--------D-KD---LSD---FY--Q-RVR-DR-DAQRQ---------EAEKNQ-FFSAPES--C---QDLGRK---LTMLLTSGKKF--IEK---- : 387

fish_XP_015810486.1 : -------GGSSK---------NLLAQATPKEYD----------------HFLQQIAQISKLRK---------KAEEKHLNGIKS--------SNQGSW---LSQMITGGSKS--LDK---- : 337

fish_XP_016115105.1 : -------GSSSK---------NLITSGSLKEYE----------------KYIKGMPHLSQLRK---------DAEEKHLSTIKI--------SVQGLK---LCEMITGGTQS--LDR---- : 362

fish_XP_016117315.1 : -------GASTR---------DIYKIGNPKVLHDEL------------TRMNVQVNVLDNRRK---------EVEKKTESKGT---------SNQGEK---LKNLLTYGGSR--LGH---- : 462

fish_XP_016395637.1 : -------GSKTE---------PVA--------D-LS-----E---FY--Q-RIS-FR-DARRE---------EAEKRH-NFIAPEL--S---QNLGKK---LIMLITGGKKI--MDK---- : 372

fish_XP_017207716.1 : -------GAATR---------DIYKIGNPKDLH------------YELTRINEQINVLDMRRR---------EAEKRPDSKRT---------SNQGEK---LKNLLTCGGNR--L------ : 445

fish_XP_017277259.1 : -------GASSS---------QIP--------E-DH---FVI---FI--Q-GLK-EK-DQQRE---------EAESSS-NETALEF--K---EDQKRK---LSILLTGGKKL--MDN---- : 393

fish_XP_017277301.1 : -------GSKTE---------PVS--------D-KD---IGD---FY--Q-RVK-DR-DAQRE---------EAEKNQ-FLSAPET--C---QDLGRK---LTMLLTSGKKF--IEK---- : 388

fish_XP_017325460.1 : -------NSSSR---------NLL----------SQTSSLKSLEEYN--KHVESMAHLSMVRL---------EAEEKHLTVVKS--------SVQGSK---LCEMLTGGSQS--LDK---- : 379

fish_XP_017346953.1 : -------GSKTE---------PVV--------E-TN-----E---FY--Q-VVH-VR-DAQRE---------EAEQAH-CFTSPGL--C---QDLGRK---LIMLVTSGKKL--MEK---- : 375

fish_XP_017550536.1 : ---------------------------------------------------SIKDSKREVKRE-----------------------------SNQGEK---LKNLLTCGAST--LGH---- : 242

fish_XP_017550824.1 : -------NSSSR---------NLLVQASSTKSL-EE---------YY--KYVDNIVRLSQLRK---------EAEEKHVTVAKN--------SVQGSR---LCEMITGGSRS--LDK---- : 368

fish_XP_017554653.1 : -------GAATR---------DICKIGNPKDLQ------------VELAKVTTGLNVLDTMRK---------QAEKKPQSKKK---------LNQGEM---LKNLFTCGSGT--LDH---- : 494

fish_XP_017569575.1 : -------GSRTQ---------PVT--------D-RN-----E---FY--H-GVQ-SR-DAQRE---------EKEQEH-LFTIPDL--C---QDLGRK---LIMLVTGGKKI--MEQ---- : 381

fish_XP_018413083.1 : -------GESSK---------DILANVKH-K-D-AD---FKM---FY--S-KMI-ER-DEARK---------KAEDNQ--RRKQNK--M---LEQGNK---LVSLITGNRDT--MDN---- : 395

fish_XP_018429299.1 : -------GAKCV---------PVADEDKVVFIQ------------------GLR--NVDDMRG---------SAEMNVSCDTSVT-------EDLGRK---ISMLVTDGKKY--IND---- : 442

fish_XP_018521334.1 : -------EGKDT---------MDTAKVMTSHYGNEGALWVTIDILKEINQCELACQLEKNMGQ---------LQQRCRPKAVLKKE------ANQGDK---LKNLLTCGGNS--LDS---- : 364

fish_XP_018526467.1 : -------GSKTE---------PVN--------D-KD---LGD---FY--Q-QVK-DR-DAQRE---------EAEKNQ-FLSAPGF--C---QDLGRK---LTMLTTSGKKF--IEK---- : 390

fish_XP_018541041.1 : -------GLRSV---------NYLADTDPRIIQ------------EKMKSLTDQVKCWASARK---------AAEEKDEKQPSQ--------GHQGQR---LKQLITRGRDT--FEN---- : 524

fish_XP_018558293.1 : -------GGSSR---------DLLAPVTFAKHM-EEYSKFID-----------SMKQRSQLRK---------EAEEKHLSVIKS--------STQGSR---LSKMITGGSFS--LDK---- : 373

fish_XP_018595329.1 : -------GASSI---------NVLATKNVRQRDEKL------------KSLRDHINAWESARK-----------DRASCVSHEPKQ------NHQGHR---LKQLIAPGRNT--LDN---- : 440

fish_XP_018611696.1 : -------GSKTE---------PVS--------D-QN-----E---FY--Q-RVK-DR-DVQRE---------EAEQAN-FLTAPDI--S---EDLGRK---LTVLVTSGKKV--IEK---- : 397

fish_XP_018956725.1 : -------GAATQ---------DICKIGNPRDWQ------------AKIAQISTKVNVLDNMRK---------EAEKRPLSKGT---------SNQGEK---LKNLLTYGGNR--LDH---- : 467

frog_XP_018091031.1 : -------GAKSE---------PVL--------E-DD---MVQ---FI--Q-GLQ-DV-DLKRQ---------RAESKR-NCDIM-P--P---ENLGRK---ISVLLTNGKQY--LDD---- : 505

Hemichordata_XP_006825609.1 : -------GSTSS---------CVEGQELHTFLE-------------------THLHVLVKQRK---------EAEQSTLINVQNISMRR---DRLARK---LQWLLCTGGTT--LDS---- : 940

lance_EEN48978.1 : -------TSKCH-------------------------------------------SWKDSSAK---------AAHRKKTSAMQRG----------------HTKAAATTKTVRTLPK---- : 199

lance_EEN55742.1 : -------GPCNA---------AAPSQNHAKVFTWFR------------------------------------DSTSPRIAKAQLKF------PLLGRGEEKWESFFEAVDQF--------- : 388

mammal_XP_001378512.1 : -------GTSSK---------NIYANPKQKDGNIKA---------FL-----AELEKCVKLRK---------EAEDHQPKEIKT--------ENEGTK---LVHLLTGNQD---LDN---- : 403
[truncated: 848,593 more chars]
