# Supplementary material for: The complex domain architecture of SAMD9 family proteins, predicted STAND-like NTPases, suggests new links to inflammation and apoptosis
Source: Biol Direct. 2017 May 25;12:13. doi: 10.1186/s13062-017-0185-2 (PMC5445408; doi:10.1186/s13062-017-0185-2)
Supplement: Supplementary file 4 — Sequence logo of the multiple alignment of 7 amino acid sequences of mammalian SAMD9 orthologs. (PPTX 1792 kb) [file 13062_2017_185_MOESM4_ESM.pptx]

## Slide 1
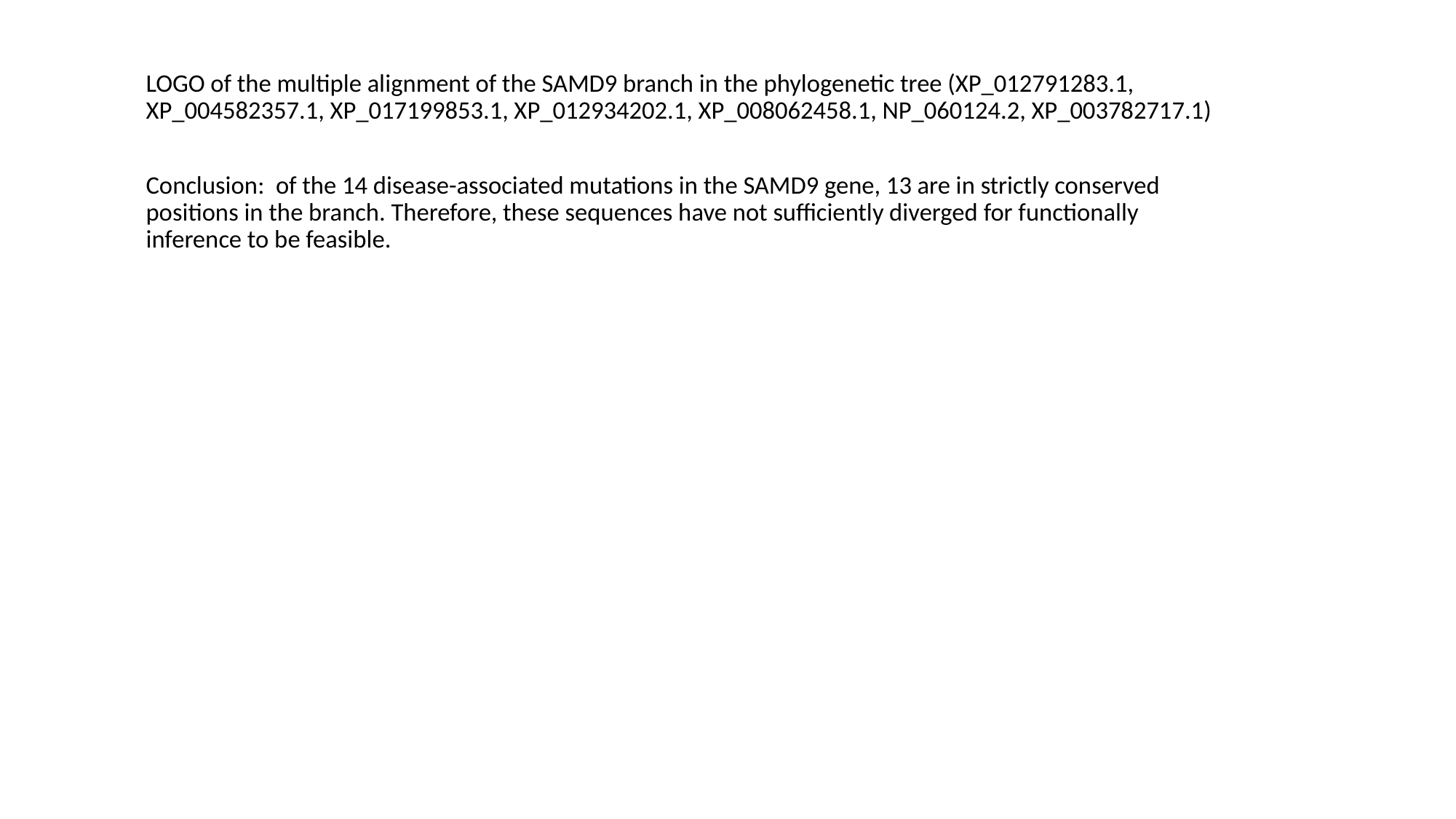

LOGO of the multiple alignment of the SAMD9 branch in the phylogenetic tree (XP_012791283.1, XP_004582357.1, XP_017199853.1, XP_012934202.1, XP_008062458.1, NP_060124.2, XP_003782717.1)
Conclusion: of the 14 disease-associated mutations in the SAMD9 gene, 13 are in strictly conserved positions in the branch. Therefore, these sequences have not sufficiently diverged for functionally inference to be feasible.

## Slide 2
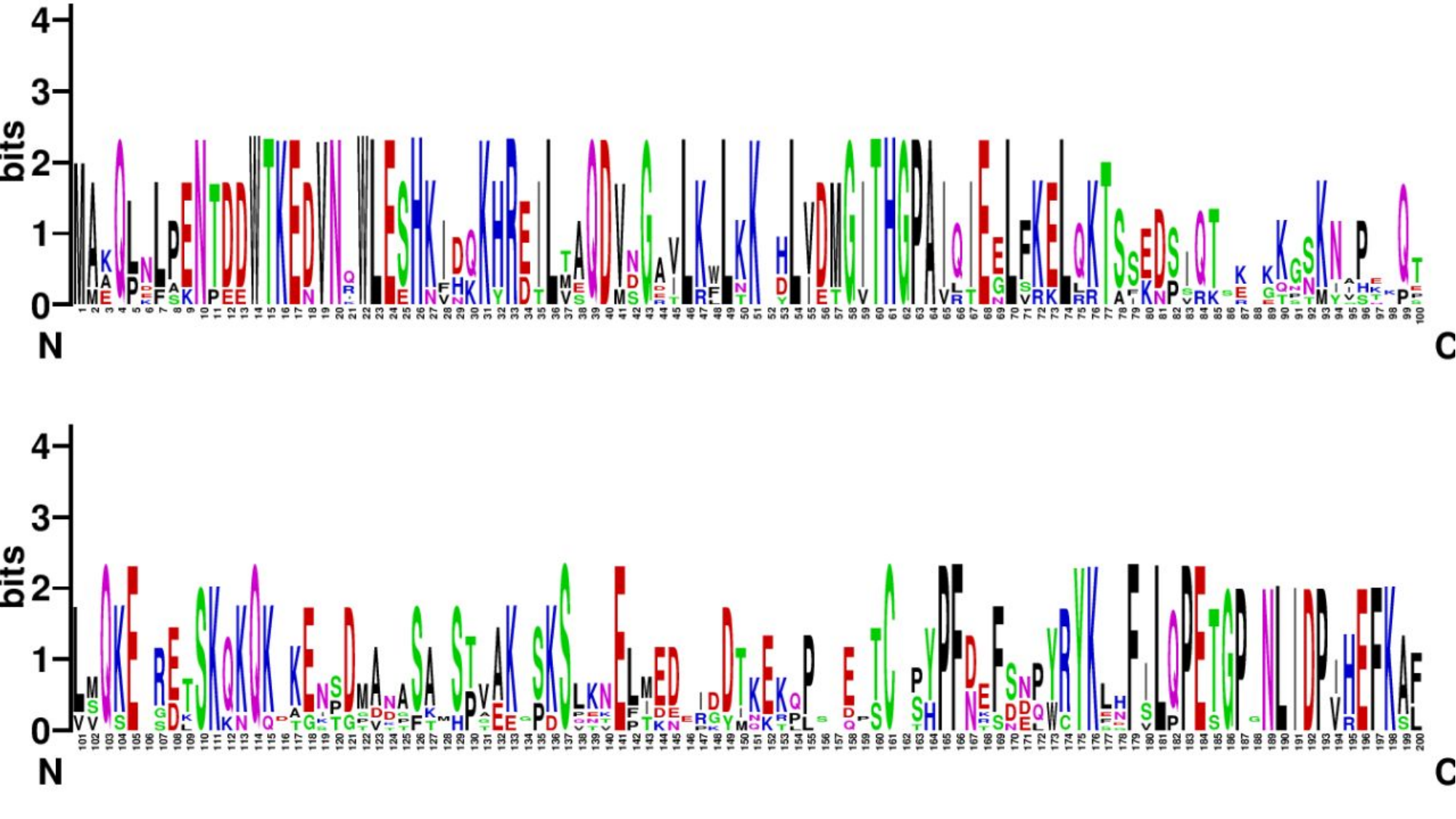

## Slide 3
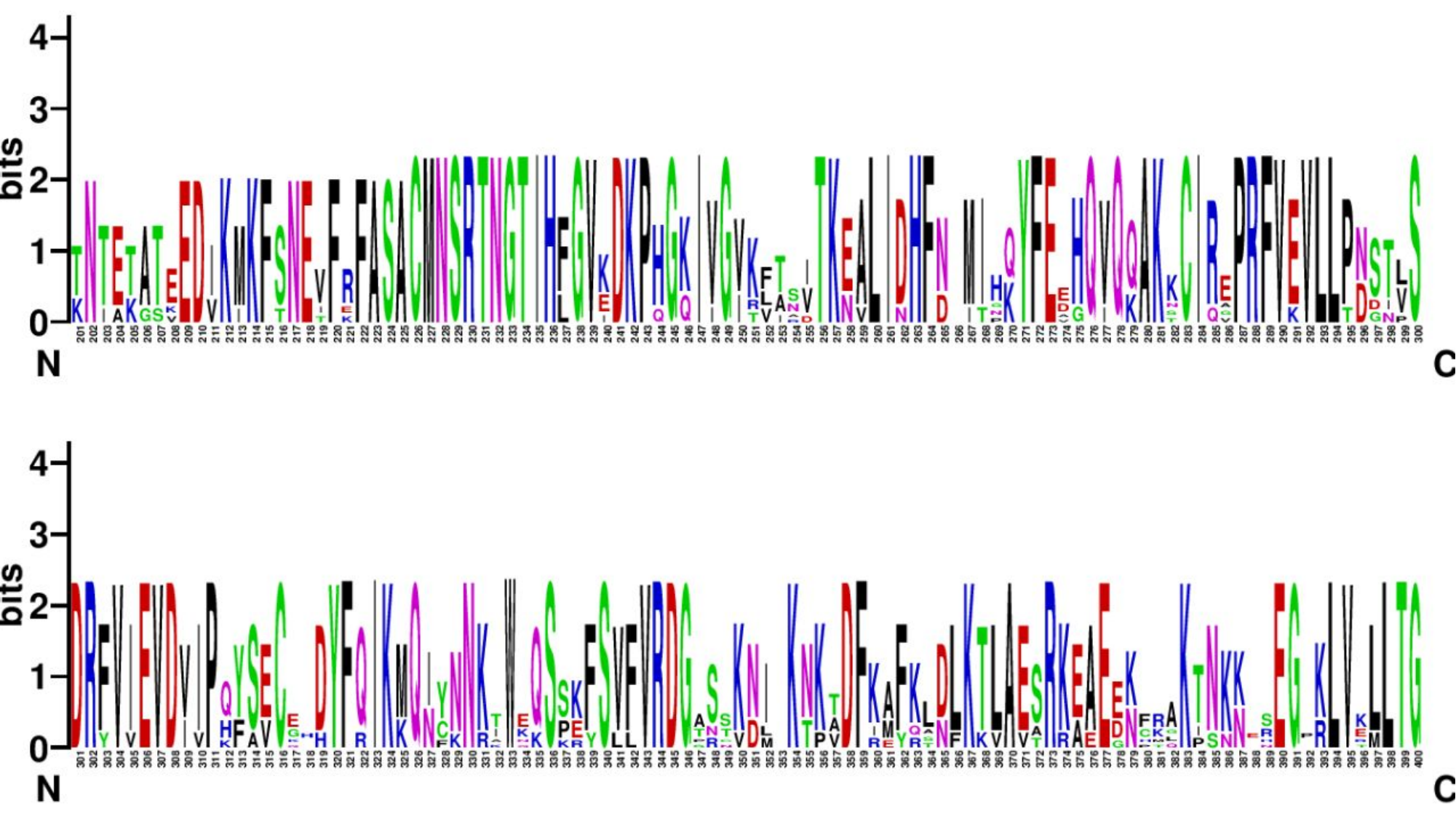

## Slide 4
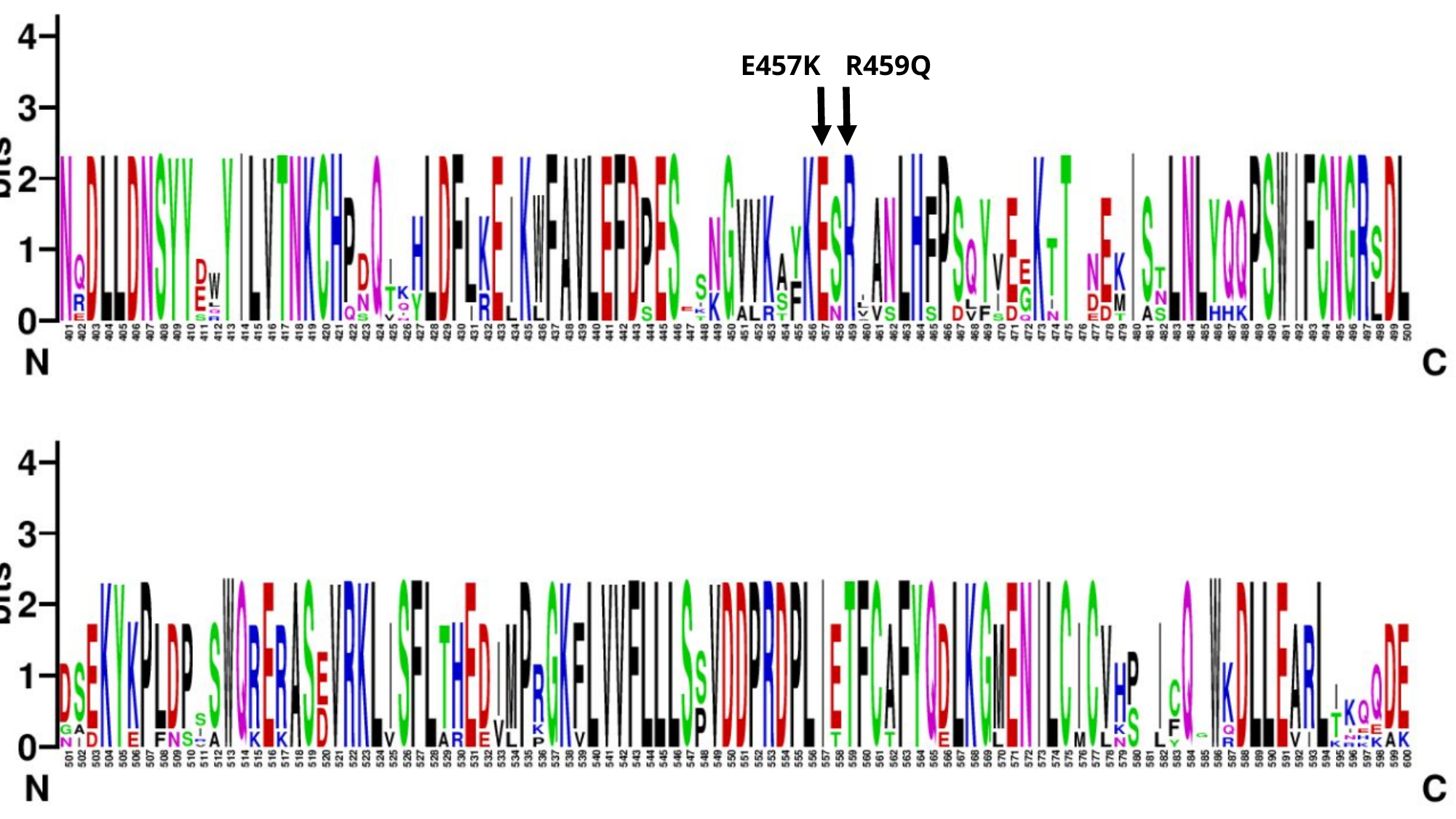

E457K
R459Q

## Slide 5
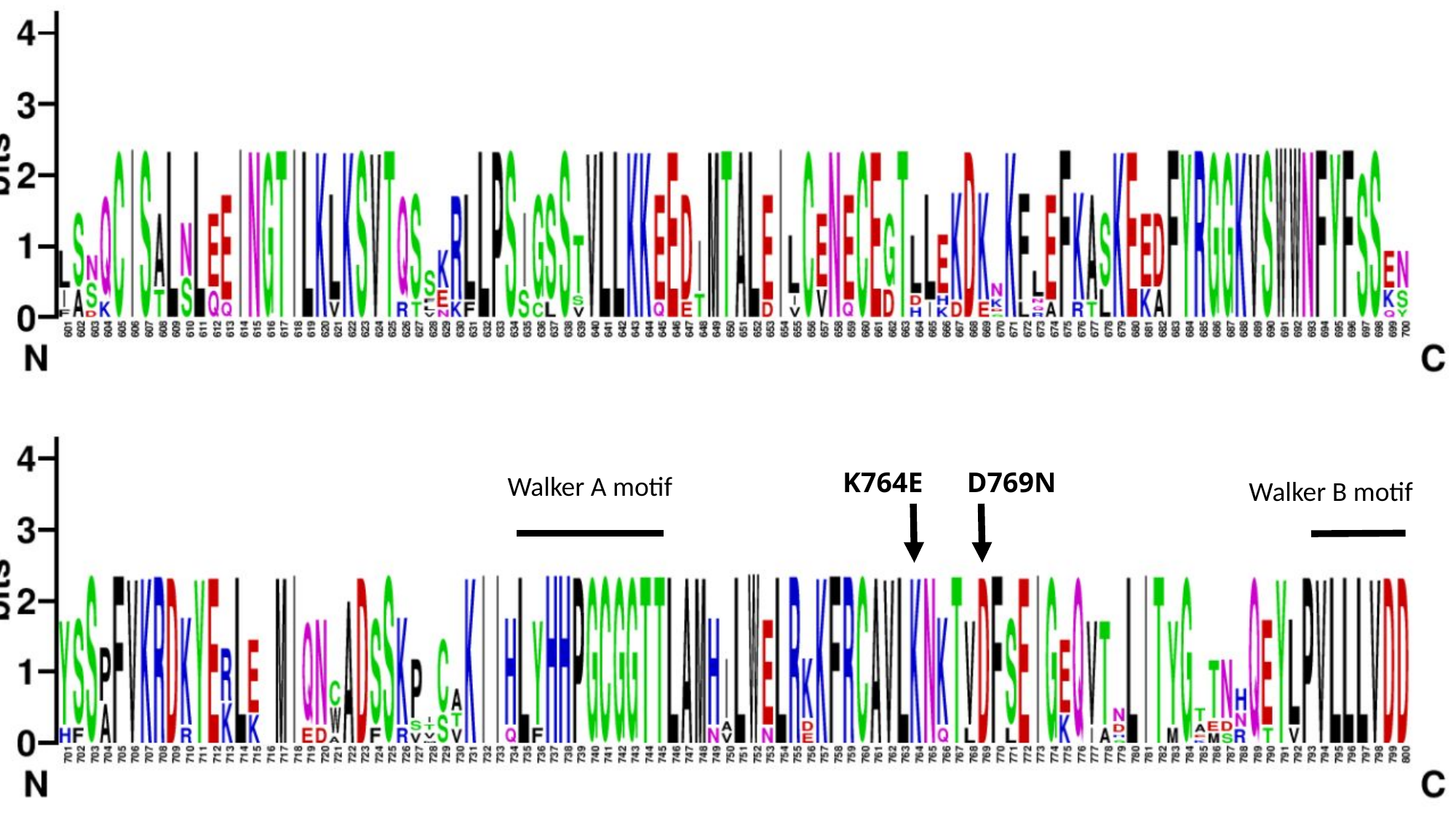

K764E
D769N
Walker A motif
Walker B motif

## Slide 6
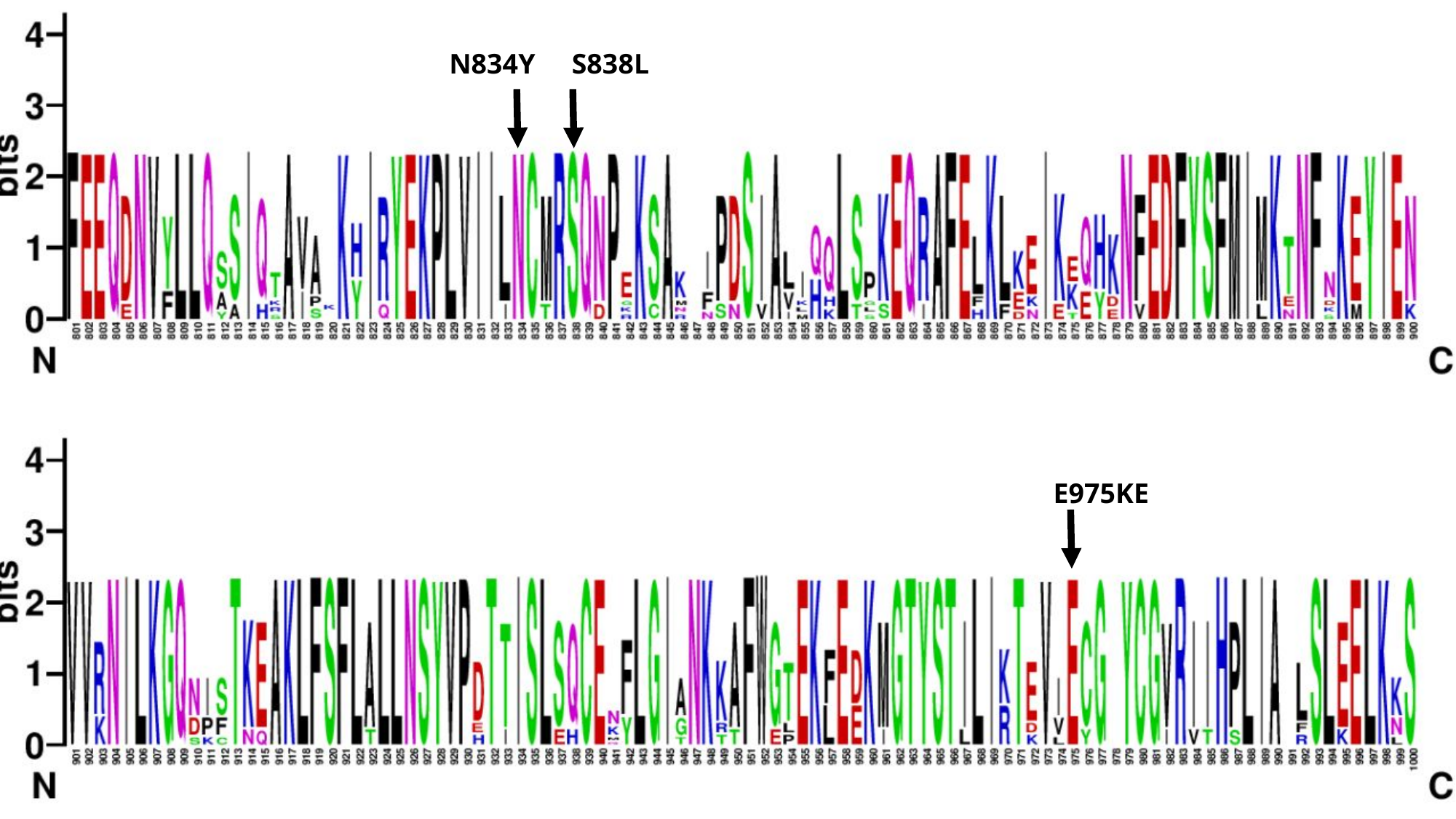

S838L
N834Y
E975KE

## Slide 7
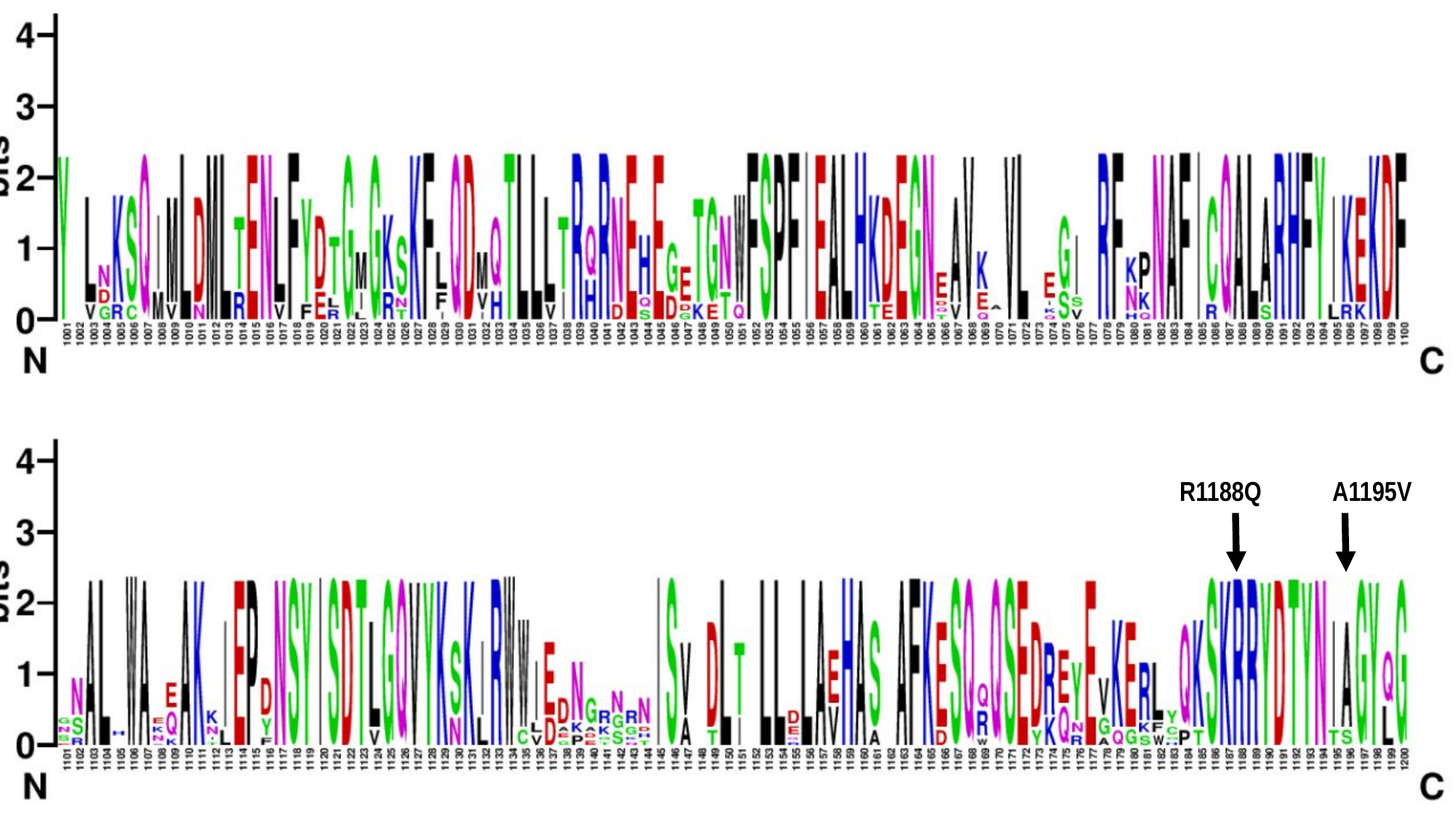

R1188Q
A1195V

## Slide 8
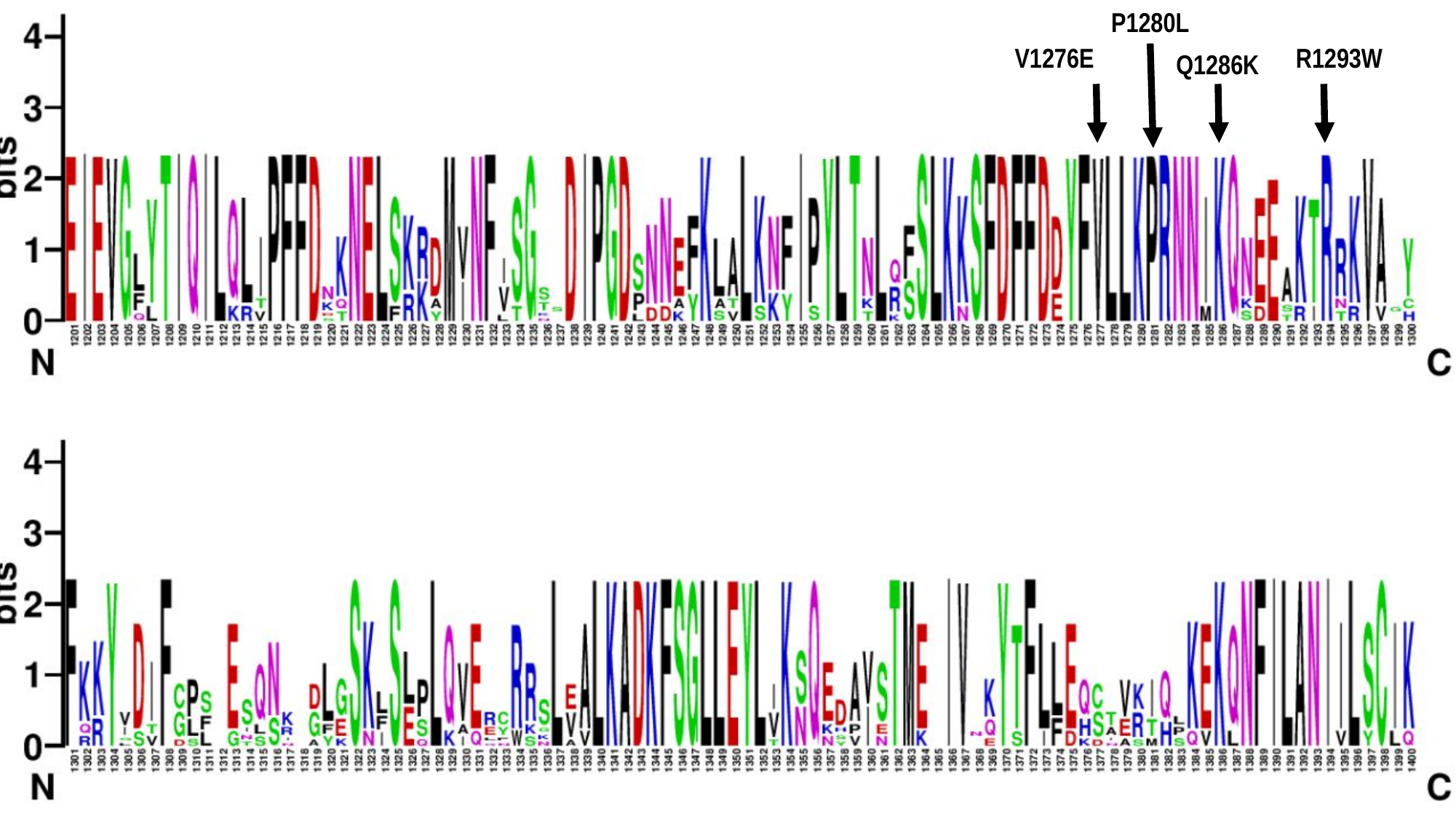

P1280L
V1276E
R1293W
Q1286K

## Slide 9
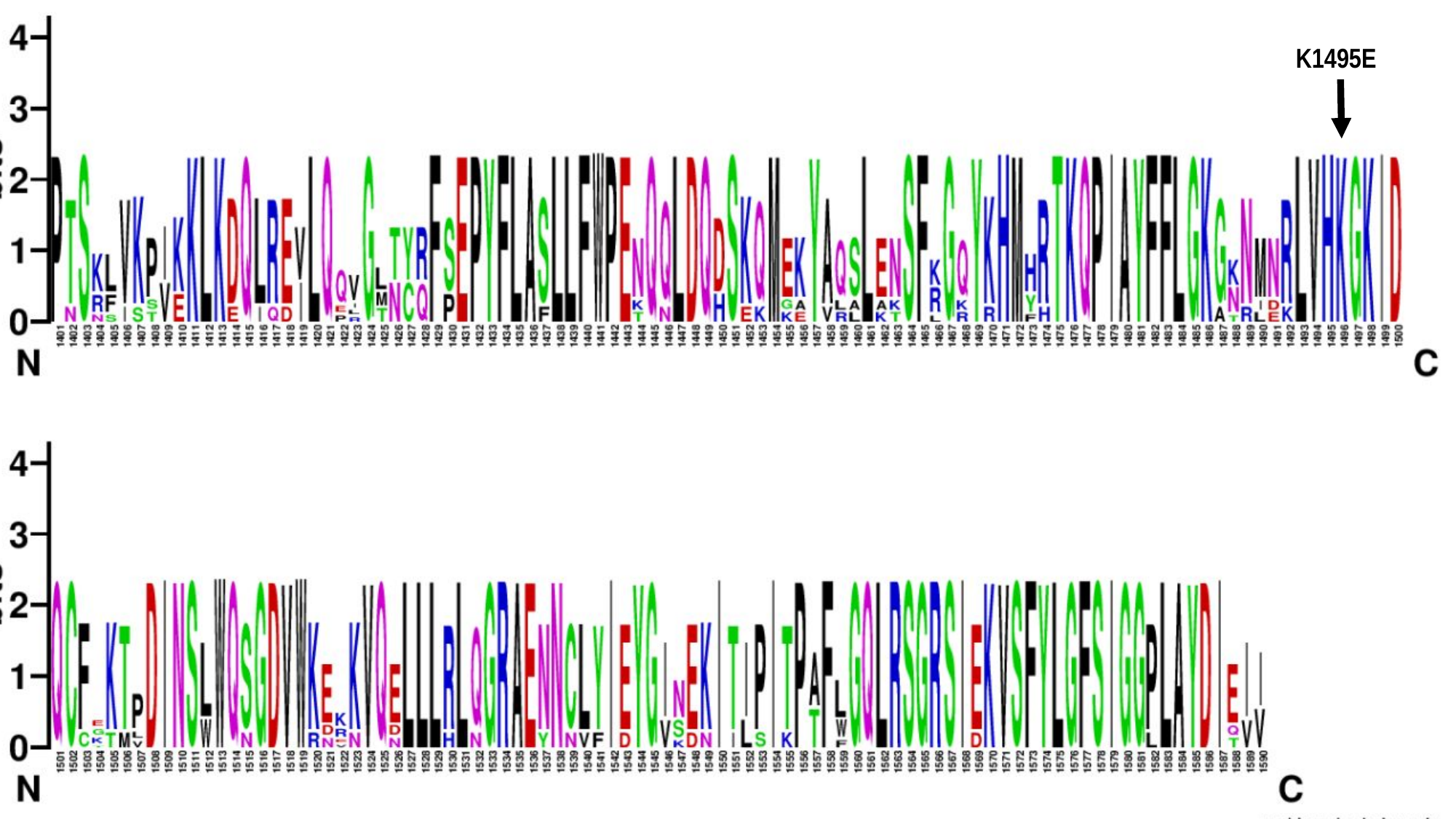

K1495E
